# Hippopotamus optimization algorithm: a novel nature-inspired optimization algorithm

**DOI:** 10.1038/s41598-024-54910-3

**Published:** 2024-02-29

**Authors:** Mohammad Hussein Amiri, Nastaran Mehrabi Hashjin, Mohsen Montazeri, Seyedali Mirjalili, Nima Khodadadi

**Affiliations:** 1 Faculty of Electrical Engineering, Shahid Beheshti University, Tehran, Iran; 2 Centre for Artificial Intelligence Research and Optimization, Torrens University Australia, Adelaide, Australia; 3 Department of Civil and Architectural Engineering, University of Miami, Coral Gables, FL USA; 4 Research and Innovation Center, Obuda University, Budapest, 1034 Hungary

**Keywords:** Engineering, Mathematics and computing

## Abstract

The novelty of this article lies in introducing a novel stochastic technique named the Hippopotamus Optimization (HO) algorithm. The HO is conceived by drawing inspiration from the inherent behaviors observed in hippopotamuses, showcasing an innovative approach in metaheuristic methodology. The HO is conceptually defined using a trinary-phase model that incorporates their position updating in rivers or ponds, defensive strategies against predators, and evasion methods, which are mathematically formulated. It attained the top rank in 115 out of 161 benchmark functions in finding optimal value, encompassing unimodal and high-dimensional multimodal functions, fixed-dimensional multimodal functions, as well as the CEC 2019 test suite and CEC 2014 test suite dimensions of 10, 30, 50, and 100 and Zigzag Pattern benchmark functions, this suggests that the HO demonstrates a noteworthy proficiency in both exploitation and exploration. Moreover, it effectively balances exploration and exploitation, supporting the search process. In light of the results from addressing four distinct engineering design challenges, the HO has effectively achieved the most efficient resolution while concurrently upholding adherence to the designated constraints. The performance evaluation of the HO algorithm encompasses various aspects, including a comparison with WOA, GWO, SSA, PSO, SCA, FA, GOA, TLBO, MFO, and IWO recognized as the most extensively researched metaheuristics, AOA as recently developed algorithms, and CMA-ES as high-performance optimizers acknowledged for their success in the IEEE CEC competition. According to the statistical post hoc analysis, the HO algorithm is determined to be significantly superior to the investigated algorithms. The source codes of the HO algorithm are publicly available at https://www.mathworks.com/matlabcentral/fileexchange/160088-hippopotamus-optimization-algorithm-ho.

## Introduction

Numerous issues and challenges in today's science, industry, and technology can be defined as optimization problems. All optimization problems have three parts: an objective function, constraints, and decision variables^1^. Optimization algorithms can be categorized in diverse manners for addressing such problems. Nonetheless, one prevalent classification method is based on its inherent approach to optimizing problems, distinguishing between stochastic and deterministic algorithms^2^. Unlike stochastic methods, deterministic methods require more extensive information about the problem^3^. However, stochastic methods do not guarantee finding a global optimal solution. In today's context, optimization problems we often encounter are nonlinear, complex, non-differentiable, piecewise functions, non-convex, and involve many decision variables^4^. For such problems, employing stochastic methods for their solution tends to be more straightforward and more suitable, especially when we have limited information about the problem or intend to treat it as a black box^5^.

One of the most important and widely used methods in stochastic approaches is metaheuristic algorithms. In metaheuristic algorithms, feasible initial solution candidates are randomly generated. Then, iteratively, these initial solutions are updated according to the specified relationships in the metaheuristic algorithm. In each step, feasible solutions with better costs are retained based on the number of search agents. This updating continues until the stopping iteration is satisfied, typically achieving a MaxIter such as the Number of Function Evaluations (NFE) or reaching a predefined cost value set by the user for the cost function. Because of the advantages of metaheuristic algorithms, they are used in various applications, and the results show that these algorithms can improve efficiency in these applications. A good optimization algorithm is able to create a balance between exploration and exploitation, in the sense that in exploration, attention is paid to global search, and in exploitation, attention is paid to local search around the obtained answers^6^.

Numerous optimization algorithms have been introduced; however, introducing and developing a new, highly innovative algorithm are still deemed necessary, as per the No Free Lunch (NFL) theorem^7^. The NFL theorem asserts that the superior performance of a metaheuristic algorithm in solving specific optimization problems does not guarantee similar success in solving different problems. Therefore, the need for an algorithm that demonstrates improved speed of convergence and the ability to find the optimal solution compared to other algorithms is highlighted. The broad scope of utilizing metaheuristic optimization algorithms has garnered attention from researchers across multiple disciplines and domains. Metaheuristic optimization algorithms find applications in a wide range of engineering disciplines, including medical engineering problems, such as improving classification accuracy by adjusting hyperparameters using metaheuristic optimization algorithms and adjusting weights in neural networks^8^ or fuzzy systems^9^.

Similarly, these algorithms contribute to intelligent fault diagnosis and tuning controller coefficients^10^ in control and mechanical engineering. In telecommunication engineering, they aid in identifying digital filters^11^, while in energy engineering, they assist in tasks such as modeling solar panels^12^, optimizing their placement, and even wind turbine placement^13^. In civil engineering, metaheuristic optimization algorithms are utilized for structural optimization^14^, while in the field of economics, they enhance stock portfolio optimization^15^. Additionally, metaheuristic optimization algorithms play a role in optimizing thermal systems in chemical engineering^16^, among other applications.

The distinctive contributions of this research lie in developing a novel metaheuristic algorithm termed the HO, rooted in the emulation of Hippopotamuses' behaviors in the natural environment. The primary achievements of this study work can be outlined as follows:

- The design of HO is influenced by the intrinsic behaviors observed in hippopotamuses, such as their position update in the river or pond, defence tactics against predators, and methods of evading predators.
- HO is mathematically formulated through a three-phase model comprising their position update, defence, and evading predators.
- To evaluate the effectiveness of the HO in solving optimization problems, it undergoes testing on a set of 161 standard BFs of various types of UM, MM, ZP benchmark test, the CEC 2019, the CEC 2014 dimensions of 10, 30, 50, and 100 to investigate the effect of the dimensions of the problem on the performance of the HO algorithm
- The performance of the HO is evaluated by comparing it with the performance of twelve widely well-kown metaheuristic algorithms.
- The effectiveness of the HO in real-world applications is tested through its application to tackle four engineering design challenges.

The article is structured into five sections. The “Literature review’’ section focuses on related work, while the “Hippopotamus Optimization Algorithm” section covers the HO approach introduced, modelled, and HO's limitations. The “Simulation results and comparison” section presents simulation results and compares the performance of the different algorithms. The performance of HO in solving classical engineering problems is studied in the “Hippopotamus optimization algorithm for engineering problems” section, and “Conclusions and future works” section provides conclusions based on the article's findings.

## Literature review

As mentioned in the introduction, it should be noted that optimization algorithms are not confined to a singular discipline or specialized research area. This is primarily because numerous real-world problems possess intricate attributes, including nonlinearity, non-differentiability, discontinuity, and non-convexity. Given these complexities and uncertainties, stochastic optimization algorithms demonstrate enhanced versatility and a heightened capacity to address such challenges effectively. Consequently, they exhibit a more remarkable ability to accommodate and navigate the intricacies and uncertainties inherent in these problems. Optimization algorithms often draw inspiration from natural phenomena, aiming to model and simulate natural processes. Physical laws, chemical reactions, animal behavior patterns, social behavior of animals, biological evolution, game theory principles, and human behavior have received significant attention in this regard. These natural phenomena serve as valuable sources of inspiration for developing optimization algorithms, offering insights into efficient and practical problem-solving strategies.

Optimization algorithms can be classified from multiple perspectives. In terms of objectives, they can be grouped into three categories: single-objective, multi-objective, and many-objective algorithms^17^. From the standpoint of decision variables, algorithms can be characterized as either continuous or discrete (or binary). Furthermore, they can be subdivided into constrained and unconstrained optimization algorithms, depending on whether constraints are imposed on the decision variables. Such classifications provide a framework for understanding and categorizing optimization algorithms based on different criteria. From another perspective, optimization algorithms can be categorized based on their sources of inspiration. These sources can be classified into six main categories: evolutionary algorithms, physics or chemistry-based algorithms, swarm-based algorithms, human-inspired algorithms, mathematic-based algorithms, and game theory-inspired algorithms. While the first four categories are well-established and widely recognized, the mathematic-based and game theory-inspired categories may need to be more known.

Optimization algorithms that draw inspiration from swarm-based are commonly utilized to model the collective behavior observed in animals, plants, and insects. For instance, the American Zebra Optimization Algorithm (ZOA)^18^. The inspiration for ZOA comes from the foraging behavior of zebras and their defensive behavior against predators during foraging. Similarly, the inspiration for Northern Goshawk Optimization (NGO)^19^ comes from the hunting behavior of the Northern Goshawk. Among the notable algorithms in this category are Particle Swarm Optimization (PSO)^20^, Ant Colony Optimization (ACO)^21^, and Artificial Bee Colony (ABC) algorithm^22^, Tunicate Swarm Algorithm (TSA)^23^, Beluga Whale Optimization (BWO)^24^, Aphid–Ant Mutualism (AAM)^25^, artificial Jellyfish Search (JS)^26^, Spotted Hyena Optimizer (SHO)^27^, Honey Badger Algorithm (HBA)^28^, Mantis Search Algorithm (MSA)^29^, Nutcraker Optimization Algorithm (NOA)^30^, Manta Ray Foraging Optimization (MRFO)^31^, Orca Predation Algorithm (OPA)^32^, Yellow Saddle Goatfish (YSG)^33^, Hermit Crab Optimization Algorithm (HCOA)^34^, Cheetah Optimizer (CO)^35^, Walrus Optimization Algorithm (WaOA)^36^, Red-Tailed Hawk algorithm (RTH)^37^, Barnacles Mating Optimizer (BMO)^38^, Meerkat Optimization Algorithm (MOA)^39^, Snake Optimizer (SO)^40^, Grasshopper Optimization Algorithm (GOA)^41^, Social Spider Optimization (SSO)^42^, Whale Optimization Algorithm (WOA)^43^, Ant Lion Optimizer (ALO)^44^, Grey Wolf Optimizer (GWO)^45^, Marine Predators Algorithm (MPA)^46^ ,Aquila Optimizer (AO)^47^, Mountain Gazelle Optimizer (MGO)^48^, Artificial Hummingbird Algorithm (AHA)^49^, African Vultures Optimization Algorithm (AVOA)^50^, Bonobo Optimizer (BO)^51^, Salp Swarm Algorithm (SSA)^52^, Harris Hawks Optimizer (HHO)^53^, Colony Predation Algorithm (CPA)^54^, Adaptive Fox Optimization (AFO)^55^, Slime Mould Algorithm (SMA)^3^, Spider Wasp Optimization (SWO)^56^, Artificial Gorilla Troops Optimizer (GTO)^57^, Krill Herd Optimization (KH)^58^, Alpine Skiing Optimization (ASO)^59^, Shuffled Frog-Leaping Algorithm (SFLA)^60^, Firefly Algorithms (FA)^61^, Komodo Mlipir Algorithm (KMA)^62^, Prairie Dog Optimization (PDO)^63^, Tasmanian Devil Optimization (TDO)^64^, Reptile Search Algorithm (RSA)^65^, Border Collie Optimization (BCO)^66^, Cuckoo Optimization Algorithm (COA)^67^ and Moth-flame optimization algorithm (MFO)^68^ are novel optimization algorithm that has been introduced in recent years. They belong to the category of swarm-based optimization algorithms. These algorithms encapsulate the principles of swarm intelligence, offering effective strategies for solving optimization problems by emulating the cooperative and adaptive behaviors found in natural swarms.

Another category of optimization algorithms is based on the origin of inspiration from biological evolution, genetics, and natural selection. The genetic optimization algorithm (GA)^69^ is one of the most well-known algorithms in this category. Among the notable algorithms in this category are Memetic Algorithm (MA)^70^, Differential Evolution (DE)^71^ Evolution Strategies (ES)^72^ Biogeography-Based Optimization (BBO)^73^, Liver Cancer Algorithm (LCA)^74^, Genetic Programming (GP)^75^, Invasive Weed Optimization algorithm (IWO)^76^, Electric Eel Foraging Optimization (EEFO)^77^, Greylag Goose Optimization (GGO)^78^ , and Puma Optimizer (PO)^79^. The Competitive Swarm Optimizer (CSO)^80^ is crafted explicitly for handling large-scale optimization challenges, taking inspiration from PSO while introducing a unique conceptual approach. In CSO, the adjustment of particle positions deviates from the inclusion of personal best positions or global best positions. Instead, it employs a pairwise competition mechanism, allowing the losing particle to learn from the winner and adjust its position accordingly. The Falcon Optimization Algorithm (FOA)^81^ is inspired by the hunting behavior of falcons. The Barnacles Mating Optimizer (BMO)^82^ algorithm takes inspiration from the mating behavior observed in barnacles in their natural habitat. The Pathfinder Algorithm (PFA)^83^ is tailored to address optimization problems with diverse structures. Drawing inspiration from the collective movement observed in animal groups and the hierarchical leadership within swarms, PFA seeks to discover optimal solutions akin to identifying food areas or prey.

Optimization algorithms are based on the origin of physical or chemical laws. As the name of this category suggests, the concepts are inspired by physical laws, chemical reactions, or chemical laws. Some of the algorithms in this category include Simulated Annealing (SA)^84^, Snow Ablation Optimizer (SAO)^85^, Electromagnetic Field Optimization (EFO)^86^, Light Spectrum Optimization (LSO)^87^, String Theory Algorithm (STA)^88^, Harmony Search (HS)^89^, Multi-Verse Optimizer (MVO)^90^, Black Hole Algorithm (BH)^91^, Gravitational Search Algorithm (GSA)^92^, Artificial Electric Field Algorithm (AEFA)^93^ draws inspiration from the principles of Coulomb's law governing electrostatic force. Magnetic Optimization Algorithm (MOA)^94^, Chemical Reaction Optimization (CRO)^95^ , Atom Search Optimization (ASO)^96^, Henry Gas Solubility Optimization (HGSO)^97^, Nuclear Reaction Optimization (NRO)^98^, Chernobyl Disaster Optimizer (CDO)^99^, Thermal Exchange Optimization (TEO)^100^, Turbulent Flow of Water-based Optimization (TFWO)^101^, Water Cycle Algorithm (WCA)^102^, Equilibrium Optimizer (EO)^103^, Lévy Flight Distribution (LFD)^104^, and Crystal Structure Algorithm (CryStAl)^105^ which takes inspiration from the symmetric arrangement of constituents in crystalline minerals like quartz.

Human-inspired algorithms derive inspiration from the social behavior, learning processes, and communication patterns found within human society. Some of the algorithms in this category include Driving Training-Based Optimization (DTBO)^106^, Fans Optimization (FO)^107^, Mother Optimization Algorithm (MOA)^108^, Mountaineering Team-Based Optimization (MTBO)^109^, Human Behavior-Based Optimization (HBBO)^110^, Chef-Based Optimization Algorithm (CBOA)^111^ is the process of acquiring culinary expertise through training programs. Teaching–Learning-Based Optimization (TLBO)^112^, Political Optimizer (PO)^113^, In the War Strategy Optimization (WSO)^114^ optimization algorithm, two human strategies during war, attack and defence, are modelled. EVolutive Election Based Optimization (EVEBO)^115^, Distance-Fitness Learning (DFL)^116^, and Cultural Algorithms (CA)^117^. Supply–Demand-Based Optimization (SDO)^118^ is inspired by the economic supply–demand mechanism and is crafted to emulate the dynamic interplay between consumers' demand and producers' supply. The Search and Rescue Optimization Algorithm (SAR)^119^ takes inspiration from the exploration behavior observed during search and rescue operations conducted by humans. The Student Psychology Based Optimization (SPBO)^120^ algorithm draws inspiration from the psychology of students who aim to enhance their exam performance and achieve the top position in their class. The Poor and Rich Optimization (PRO)^121^ algorithm is inspired by the dynamics between the efforts of poor and rich individuals to improve their economic situations. The algorithm mirrors the behavior of both the rich, who seek to widen the wealth gap, and the poor, who endeavor to accumulate wealth and narrow the gap with the affluent.

Game-based optimization algorithms often model the rules of a game. Some of the algorithms in this category include Squid Game Optimizer (SGO)^122^, Puzzle Optimization Algorithm (POA)^123^, and Darts Game Optimizer (DGO)^124^.

Mathematical theories inspire mathematical algorithms. For example, Arithmetic Optimization Algorithm (AOA)^125^ ,the Chaos Game Optimization (CGO)^126^ is inspired by chaos theory and fractal configuration principles. Another known algorithm in this category are Sine Cosine Algorithm (SCA)^127^, Evolution Strategy with Covariance Matrix Adaptation (CMA-ES)^128^, and Quadratic Interpolation Optimization (QIO).

## Hippopotamus optimization algorithm

In this section, we articulate the foundational inspiration and theoretical underpinnings of the proposed HO Algorithm.

### Hippopotamus

The hippopotamus is one of the fascinating creatures residing in Africa^129^. This animal falls under the classification of vertebrates and specifically belongs to the group of mammals within the vertebrate category^130^. Hippopotamuses are semi-aquatic organisms that predominantly occupy their time in aquatic environments, specifically rivers and ponds, as part of their habitat^131,132^. Hippopotamuses exhibit a social behavior wherein they reside in collective units referred to as pods or bloats, typically comprising a population ranging from 10 to 30 individuals^133^. Determining the gender of hippopotamuses is not easily accomplished as their sexual organs are not external, and the only distinguishing factor lies in the difference in their weight. Adult hippopotamuses can stay submerged underwater for up to 5 min. This species of animal, in terms of appearance, bears resemblance to venomous mammals such as the shrew, but its closest relatives are whales and dolphins, with whom they shared a common ancestor around 55 million years ago^134^.

Despite their herbivorous nature and reliance on a diet consisting mainly of grass, branches, leaves, reeds, flowers, stems, and plant husks^135^, hippopotamuses display inquisitiveness and actively explore alternative food sources. Biologists believe that consuming meat can cause digestive issues in hippopotamuses. These animals possess extremely powerful jaws, aggressive temperament, and territorial behavior, which has classified them as one of the most dangerous mammals in the world^136^. The weight of male hippopotamuses can reach up to 9,920 pounds, while females typically weigh around 3,000 pounds. They consume approximately 75 pounds of food daily. Hippopotamuses engage in frequent conflicts with one another, and occasionally, during these confrontations, one or multiple hippopotamus calves may sustain injuries or even perish. Due to their large size and formidable strength, predators generally do not attempt to hunt or attack adult hippopotamuses. However, young hippopotamuses or weakened adult individuals become vulnerable prey for Nile crocodiles, lions, and spotted hyenas^134^.

When attacked by predators, hippopotamuses exhibit a defensive behavior by rotating towards the assailant and opening their powerful jaws. This is accompanied by emitting a loud vocalization, reaching approximately 115 decibels, which instils fear and intimidation in the predator, often deterring them from pursuing such a risky prey. When the defensive approach of a hippopotamus proves ineffective or when the hippopotamus is not yet sufficiently strong, it retreats rapidly at speeds of approximately 30 km/h to distance itself from the threat. In most cases, it moves towards nearby water bodies such as ponds or rivers^136^.

### Inspiration

The HO draws inspiration from three prominent behavioral patterns observed in the life of hippopotamuses. Hippopotamus groups are comprised of several female hippopotamuses, hippopotamus calves, multiple adult male hippopotamuses, and a dominant male hippopotamus (the leader of the herd)^136^. Due to their inherent curiosity, young and calves hippopotamuses often display a tendency to wander away from the group. As a consequence, they may become isolated and become targets for predators.

**Figure 1:**
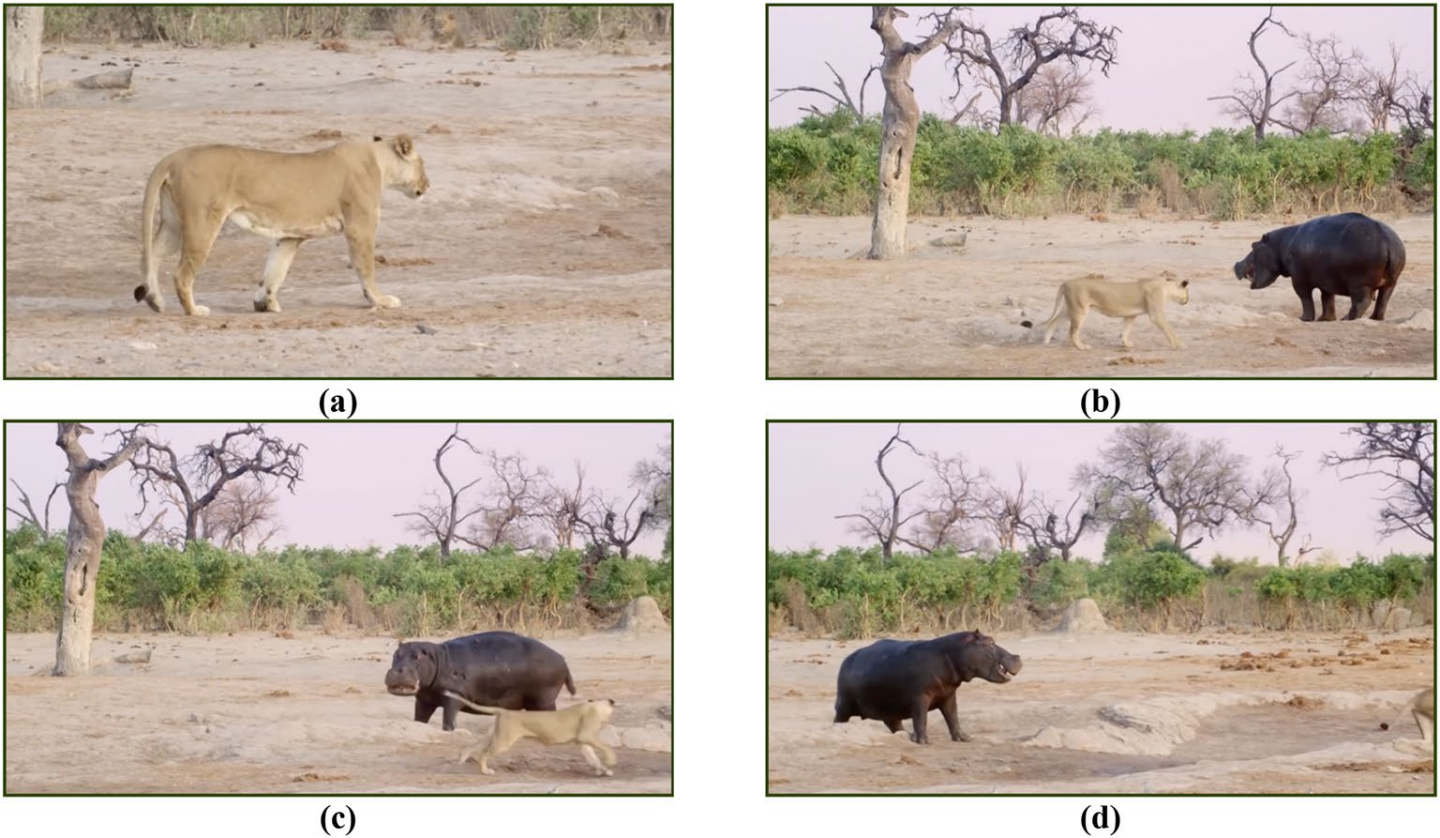
(**a**–**d**) shows the defensive behavior of the hippopotamus against the predator^136^.

The secondary behavioral pattern of hippopotamuses is defensive in nature, triggered when they are under attack by predators or when other creatures intrude into their territory. Hippopotamuses exhibit a defensive response by rotating themselves toward the predator and employing their formidable jaws and vocalizations to deter and repel the attacker (Fig. 1). Predators such as lions and spotted hyenas possess an awareness of this phenomenon and actively seek to avoid direct exposure to the formidable jaws of a hippopotamus as a precautionary measure against potential injuries. The final behavioral pattern encompasses the hippopotamus' instinctual response of fleeing from predators and actively seeking to distance itself from areas of potential danger. In such circumstances, the hippopotamus strives to navigate toward the closest body of water, such as a river or pond, as lions and spotted hyenas frequently exhibit aversion to entering aquatic environments.

#### Mathematical modelling of HO

The HO is a population-based optimization algorithm, in which search agents are hippopotamuses. In the HO algorithm, hippopotamuses are candidate solutions for the optimization problem, meaning that the position update of each hippopotamus in the search space represents values for the decision variables. Thus, each hippopotamus is represented as a vector, and the population of hippopotamuses is mathematically characterized by a matrix. Similar to conventional optimization algorithms, the initialization stage of the HO involves the generation of randomized initial solutions. During this step, the vector of decision variables is generated using the following formula:

1 $${\chi }_{\mathcalligra{i}}: {\mathcalligra{x}}_{\mathcalligra{i},\mathcalligra{j}}={\mathcalligra{l}\mathcalligra{b}}_{\mathcalligra{j}}+r.\left({{\mathcalligra{u}\mathcalligra{b}}_{\mathcalligra{j}}-\mathcalligra{l}\mathcalligra{b}}_{\mathcalligra{j}}\right), \mathcalligra{i}=\mathrm{1,2},\dots ,\mathcal{N} ,\mathcalligra{j}=\mathrm{1,2},\dots ,\mathcalligra{m}$$

where ${\chi }_{\mathcalligra{i}}$ represents the position of the $i$ th candidate solution, $r$ is a random number in the range of 0 to 1, and $\mathcalligra{l}\mathcalligra{b}$ and $\mathcalligra{u}\mathcalligra{b}$ denote the lower and upper bounds of the $j$ th decision variable, respectively. Given that $\mathcal{N}$ denotes the population size of hippopotamuses within the herd, and *m* represents the number of decision variables in the problem, the population matrix is formed by Eq. (2).

2 $$\chi = \left[ {\begin{array}{*{20}c} {\chi _{1} } \\ \vdots \\ {\chi _{i} } \\ \vdots \\ {\chi _{{\mathcal{N}}} } \\ \end{array} } \right]_{{{\mathcal{N}} \times {\mathcalligra{m}}}} = \left[ {\begin{array}{*{20}c} {\mathcalligra{x}_{{1,1}} } & \cdots & {v_{{1,\mathcalligra{j}}} } & \cdots & {\mathcalligra{x}_{{1,\mathcalligra{m}}} } \\ \vdots & \ddots & \vdots & {\mathinner{\mkern2mu\raise1pt\hbox{.}\mkern2mu \raise4pt\hbox{.}\mkern2mu\raise7pt\hbox{.}\mkern1mu}} & \vdots \\ {\mathcalligra{x}_{{\mathcalligra{i},1}} } & \cdots & {\mathcalligra{x}_{{\mathcalligra{i},\mathcalligra{j}}} } & \cdots & {\mathcalligra{x}_{{\mathcalligra{i},\mathcalligra{m}}} } \\ \vdots & {\mathinner{\mkern2mu\raise1pt\hbox{.}\mkern2mu \raise4pt\hbox{.}\mkern2mu\raise7pt\hbox{.}\mkern1mu}} & \vdots & \ddots & \vdots \\ {\mathcalligra{x}_{{{\mathcal{N}},1}} } & \cdots & {\mathcalligra{x}_{{{\mathcal{N}},\mathcalligra{j}}} } & \cdots & {\mathcalligra{x}_{{{\mathcal{N}},\mathcalligra{m}}} } \\ \end{array} } \right]_{{{\mathcal{N}} \times \mathcalligra{m}}}$$

##### Phase 1: The hippopotamuses position update in the river or pond (Exploration)

Hippopotamus herds are composed of several adult female hippopotamuses, calves hippopotamuses, multiple adult male hippopotamuses, and dominant male hippopotamuses (the leader of the herd). The dominant hippopotamus is determined based on the objective function value iteration (The lowest for the minimization problem and the highest for the maximization problem). Typically, hippopotamuses tend to gather in close proximity to one another. Dominant male hippopotamuses protect the herd and territory from potential threats. Multiple female hippopotamuses are positioned around the male hippopotamuses. Upon reaching maturity, male hippopotamuses are ousted from the herd by the dominant male. Subsequently, these expelled male individuals are required to either attract females or engage in dominance contests with other established male members of the herd in order to establish their own dominance. Equation (3) expresses the mathematical representation of the position of male hippopotamus members of the herd in the lake or pond.

3 $${{\chi }_{\mathcalligra{i}}}^{\mathcal{M}\mathcalligra{h}\mathcalligra{i}\mathcalligra{p}\mathcalligra{p}\mathcalligra{o}}:{\mathcalligra{x}}_{\mathcalligra{i},\mathcalligra{j}}^{\mathcal{M}\mathcalligra{h}\mathcalligra{i}\mathcalligra{p}\mathcalligra{p}\mathcalligra{o}}={\mathcalligra{x}}_{\mathcalligra{i},\mathcalligra{j}}+{\mathcalligra{y}}_{1}.\left(\mathcal{D}\mathcalligra{h}\mathcalligra{i}\mathcalligra{p}\mathcalligra{p}\mathcalligra{o}-{I}_{1}{\mathcalligra{x}}_{\mathcalligra{i},\mathcalligra{j}}\right)$$

$$for \mathcalligra{i}=\mathrm{1,2},\dots ,\left[\frac{\mathcal{N}}{2}\right] and \mathcalligra{j}=\mathrm{1,2},\dots ,\mathcalligra{m}$$

In Eq. (3) ${{\chi }_{\mathcalligra{i}}}^{\mathcalligra{m}\mathcalligra{h}\mathcalligra{i}\mathcalligra{p}\mathcalligra{p}\mathcalligra{o}}$ represents male hippopotamus position, $\mathcal{D}\mathcalligra{h}\mathcalligra{i}\mathcalligra{p}\mathcalligra{p}\mathcalligra{o}$ denotes the dominant hippopotamus position (The hippopotamus that has the best cost in the current iteration). ${\overrightarrow{r}}_{1,\dots ,4}$ is a random vector between 0 and 1, ${r}_{5}$ is a random number between 0 and 1 (Eq. 4), ${I}_{1}$ and ${I}_{2}$ is an integer between 1 and 2 (Eqs. 3 and 6). ${\mathcalligra{m}\mathcal{G}}_{\mathcalligra{i}}$ refers to the mean values of some randomly selected hippopotamus with an equal probability of including the current considered hippopotamus (${\chi }_{i}$) and ${\mathcalligra{y}}_{1}$ is a random number between 0 and 1 (Eq. 3). In Eq. (4) ${\varrho }_{1}$ and ${\varrho }_{2}$ are integer random numbers that can be one or zero.

4 $$\mathcalligra{h}=\left\{\begin{array}{c}{I}_{2}\times {\overrightarrow{r}}_{1}+(\sim {\varrho }_{1})\\ 2\times {\overrightarrow{r}}_{2}-1\\ {\overrightarrow{r}}_{3}\\ {I}_{1}\times {\overrightarrow{r}}_{4}+(\sim {\varrho }_{2})\\ {r}_{5}\end{array}\right.$$

5 $$T={\text{exp}}\left(-\frac{\mathcalligra{t}}{\mathcal{T}}\right)$$

6 $${{\chi }_{\mathcalligra{i}}}^{\mathcal{F}\mathcal{B}\mathcalligra{h}\mathcalligra{i}\mathcalligra{p}\mathcalligra{p}\mathcalligra{o}} :{\mathcalligra{x}}_{\mathcalligra{i},\mathcalligra{j}}^{\mathcal{F}\mathcal{B}\mathcalligra{h}\mathcalligra{i}\mathcalligra{p}\mathcalligra{p}\mathcalligra{o}}=\left\{\begin{array}{c}{\mathcalligra{x}}_{\mathcalligra{i},\mathcalligra{j}}+{\mathcalligra{h}}_{1} .\left(\mathcal{D}\mathcalligra{h}\mathcalligra{i}\mathcalligra{p}\mathcalligra{p}\mathcalligra{o}-.{I}_{2}{\mathcal{M}\mathcal{G}}_{\mathcalligra{i}}\right) T>0.6 \\ \Xi \quad\quad\quad\quad\quad\quad\quad\quad\quad\quad\quad else\end{array}\right.$$

7 $$\Xi =\left\{\begin{array}{c}{\mathcalligra{x}}_{\mathcalligra{i},\mathcalligra{j}}+{\mathcalligra{k}}_{2} .\left({\mathcal{M}\mathcal{G}}_{\mathcalligra{i}}-\mathcal{D}\mathcalligra{h}\mathcalligra{i}\mathcalligra{p}\mathcalligra{p}\mathcalligra{o}\right) { r}_{6}>0.5 \\ {\mathcalligra{l}\mathcalligra{b}}_{\mathcalligra{j}}+{r}_{7}.\left({{\mathcalligra{u}\mathcalligra{b}}_{\mathcalligra{j}}-\mathcalligra{l}\mathcalligra{b}}_{\mathcalligra{j}}\right) else\end{array}\right.$$

$$for \mathcalligra{i}=\mathrm{1,2},\dots ,\left[\frac{\mathcal{N}}{2}\right] and \mathcalligra{j}=\mathrm{1,2},\dots ,\mathcalligra{m} .$$

Equations (6) and (7) describe female or immature hippopotamus position (${{\chi }_{\mathcalligra{i}}}^{\mathcal{F}\mathcal{B}\mathcalligra{h}\mathcalligra{i}\mathcalligra{p}\mathcalligra{p}\mathcalligra{o}}$) within the herd. Most immature hippopotamuses are near their mothers, but due to curiosity, sometimes immature hippopotamuses are separated from the herd or away from their mothers. If $T$ is greater than 0.6, it means the immature hippopotamus has distanced itself from its mother (Eq. 5). If ${r}_{6}$, which is a number between 0 and 1 (Eq. 7), is greater than 0.5, it means the immature hippopotamus has distanced itself from its mother but is still within or near the herd, Otherwise, it has separated from the herd. This behavior of immature and female hippopotamuses is modelled according to Eqs. (6) and (7). ${\mathcalligra{h}}_{1}$ and ${\mathcalligra{h}}_{2}$ are numbers or vectors randomly selected from the five scenarios in the $\mathcalligra{h}$ equation. In Eq. (7) ${r}_{7}$ is a random number between zero and one. Equations (8), (9) describe male and female or immature hippopotamus position update within the herd. ${\mathcal{F}}_{\mathcalligra{i}}$ is objective function value.

8 $${\chi }_{\mathcalligra{i}}=\left\{\begin{array}{*{20}l}{{\chi }_{\mathcalligra{i}}}^{\mathcal{M}\mathcalligra{h}\mathcalligra{i}\mathcalligra{p}\mathcalligra{p}\mathcalligra{o}}{\mathcal{F}}_{\mathcalligra{i}}^{\mathcal{M}\mathcalligra{h}\mathcalligra{i}\mathcalligra{p}\mathcalligra{p}\mathcalligra{o}} &\quad <{\mathcal{F}}_{\mathcalligra{i}}\\ {\chi }_{\mathcalligra{i}} & \quad else \end{array}\right.$$

9 $${\chi }_{\mathcalligra{i}}=\left\{\begin{array}{*{20}l}{{\chi }_{\mathcalligra{i}}}^{\mathcal{F}\mathcal{B}\mathcalligra{h}\mathcalligra{i}\mathcalligra{p}\mathcalligra{p}\mathcalligra{o}}{\mathcal{F}}_{\mathcalligra{i}}^{\mathcal{F}\mathcal{B}\mathcalligra{h}\mathcalligra{i}\mathcalligra{p}\mathcalligra{p}\mathcalligra{o}}& \quad <{\mathcal{F}}_{\mathcalligra{i}}\\ {\chi }_{\mathcalligra{i}} & \quad else\end{array}\right.$$

Using $\mathcalligra{h}$ vectors, ${I}_{1}$ and ${I}_{2}$ scenarios enhance the global search and improves exploration in the proposed algorithm. It leads to a better global search and enhances the exploration process in the proposed algorithm.

##### Phase 2: Hippopotamus defence against predators (Exploration)

One of the key reasons for the herd living of hippopotamuses can be attributed to their safety and security. The presence of these large and heavy-weighted herding’s of animals can deter predators from approaching them closely. Nevertheless, due to their inherent curiosity, immature hippopotamuses may occasionally deviate from the herd and become potential targets for Nile crocodiles, lions, and spotted hyenas, given their relatively lesser strength in comparison to adult hippopotamuses. Sick hippopotamuses, similar to immature ones, are also susceptible to being preyed upon by predators.

**Figure 2:**
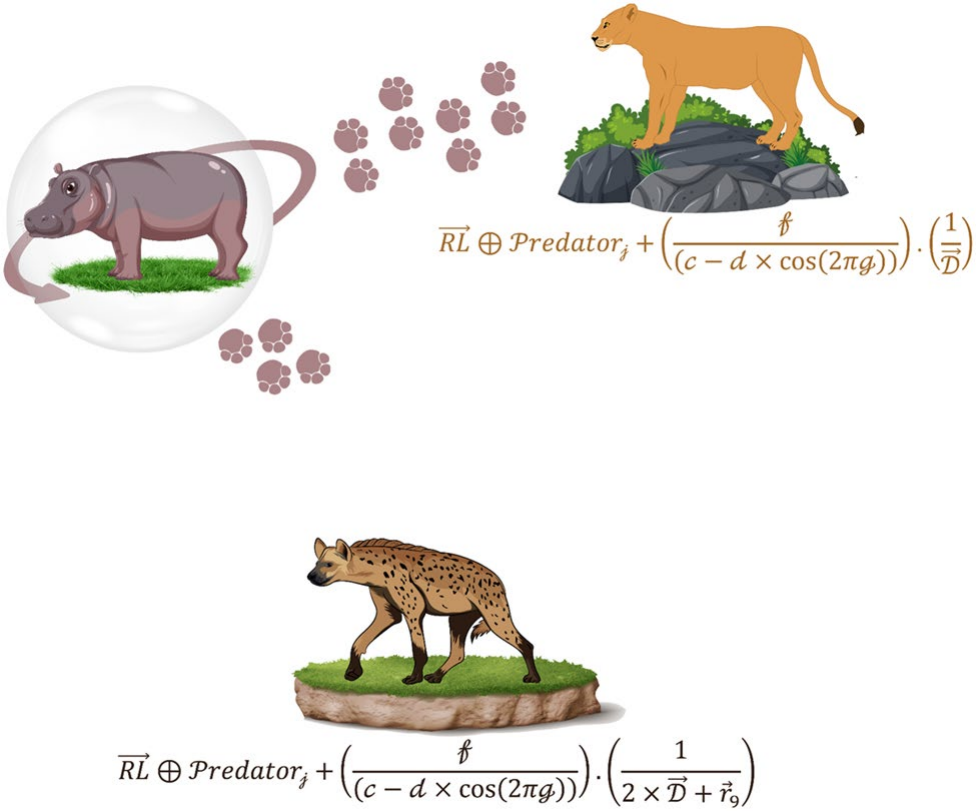
Graphic representation of the phase 2.

The primary defensive tactic employed by hippopotamuses is swiftly turning towards the predator and emitting loud vocalizations to deter the predator from approaching them closely (Fig. 2). During this phase, hippopotamuses may exhibit a behavior of approaching the predator to induce its retreat, thus effectively warding off the potential threat. Equation (10) represents the predator's position in search space.

10 $$\mathcal{P}redator: {\mathcal{P}redator}_{\mathcalligra{j}}={\mathcalligra{l}\mathcalligra{b}}_{\mathcalligra{j}}+{\overrightarrow{r}}_{8}.\left({{\mathcalligra{u}\mathcalligra{b}}_{\mathcalligra{j}}-\mathcalligra{l}\mathcalligra{b}}_{\mathcalligra{j}}\right), \mathcalligra{j}=\mathrm{1,2},\dots ,\mathcalligra{m}.$$

where ${\overrightarrow{r}}_{8}$ represents a random vector ranging from zero to one.

11 $$\overrightarrow{\mathcal{D}}=\left|{\mathcal{P}redator}_{\mathcalligra{j}}-{\mathcalligra{x}}_{\mathcalligra{i},\mathcalligra{j}}\right|$$

Equation (11) indicates the distance of the $\mathcalligra{i}th$ hippopotamus to the predator. During this time, the hippopotamus adopts a defensive behavior based on the factor ${\mathcal{F}}_{{\mathcal{P}redator}_{\mathcalligra{j}}}$ to protect itself against the predator. If ${\mathcal{F}}_{{\mathcal{P}redator}_{\mathcalligra{j}}}$ is less than ${\mathcal{F}}_{\mathcalligra{i}}$, indicating the predator is in very close proximity to the hippopotamus, in such a case, the hippopotamus swiftly turns towards the predator and moves towards it to make it retreat. If ${\mathcal{F}}_{{\mathcal{P}redator}_{\mathcalligra{j}}}$ is greater, it indicates that the predator or intruding entity is at a greater distance from the hippopotamus's territory Eq. (12). In this case, the hippopotamus turns towards the predator but with a more limited range of movement. The intention is to make the predator or intruder aware of its presence within its territory.

12 $${{\chi }_{\mathcalligra{i}}}^{\mathcal{H}\mathcalligra{i}\mathcalligra{p}\mathcalligra{p}\mathcalligra{o}\mathcal{R}}:{\mathcalligra{x}}_{\mathcalligra{i},\mathcalligra{j}}^{\mathcal{H}\mathcalligra{i}\mathcalligra{p}\mathcalligra{p}\mathcalligra{o}\mathcal{R}}=\left\{\begin{array}{c}\overrightarrow{RL}\oplus {\mathcal{P}redator}_{\mathcalligra{j}},+\left(\frac{\mathcalligra{f}}{\left(\mathcalligra{c}-\mathcalligra{d}\times cos\left(2\pi \mathcalligra{g}\right)\right)}\right).\left(\frac{1}{\overrightarrow{\mathcal{D}}}\right) {\mathcal{F}}_{{\mathcal{P}redator}_{\mathcalligra{j}}}<{\mathcal{F}}_{\mathcalligra{i}}\\ \overrightarrow{RL}\oplus {\mathcal{P}redator}_{\mathcalligra{j}},+\left(\frac{\mathcalligra{f}}{\left(\mathcalligra{c}-\mathcalligra{d}\times cos\left(2\pi \mathcalligra{g}\right)\right)}\right).\left(\frac{1}{2\times \overrightarrow{\mathcal{D}}+{\overrightarrow{r}}_{9}.}\right){\mathcal{F}}_{{\mathcal{P}redator}_{\mathcalligra{j}}}\ge {\mathcal{F}}_{\mathcalligra{i}},\end{array}\right.$$

$$for \mathcalligra{i}=\left[\frac{\mathcal{N}}{2}\right]+1,\left[\frac{\mathcal{N}}{2}\right]+2,\dots ,\mathcal{N} and \mathcalligra{j}=\mathrm{1,2},\dots ,\mathcalligra{m}$$

${{\chi }_{\mathcalligra{i}}}^{\mathcal{H}\mathcalligra{i}\mathcalligra{p}\mathcalligra{p}\mathcalligra{o}\mathcal{R}}$ is a hippopotamus position which was faced to predator. $\overrightarrow{RL}$ is a random vector with a Levy distribution, utilized for sudden changes in the predator's position during an attack on the hippopotamus. The mathematical model for the random movement of Lévy movement^46^ is calculated as Eq. (13). $\mathcalligra{w}$ and $\mathcalligra{v}$ are the random numbers in [0,1], respectively; $\vartheta$ is a constant ($\vartheta$ = 1.5), $\Gamma$ is an abbreviation for Gamma function and ${\sigma }_{\mathcalligra{w}}$ can be obtained by Eq. (14).

13 $$\mathcal{L}\mathcalligra{e}\mathcalligra{v}\mathcalligra{y}\left(\vartheta \right)=0.05\times \frac{\mathcalligra{w}\times {\sigma }_{\mathcalligra{w}}}{{\left|\mathcalligra{v}\right|}^{\frac{1}{\vartheta }}}$$

14 $${\sigma }_{\mathcalligra{w}}={\left[\frac{\Gamma \left(1+\vartheta \right){\text{sin}}\left(\frac{\pi \vartheta }{2}\right)}{\Gamma \left(\frac{\left(1+\vartheta \right)}{2}\right)\vartheta {2}^{\frac{\left(\vartheta -1\right)}{2}}}\right]}^{\frac{1}{\vartheta }}$$

In Eq. (12) $\mathcalligra{f}$ is a uniform random number between 2 and 4, $\mathcalligra{c}$ is a uniform random number between 1 and 1.5 and $\mathcal{D}$ is a uniform random number between 2 and 3. $\mathcalligra{g}$ represents a uniform random number between − 1 and 1. ${\overrightarrow{r}}_{9}$ is a random vector with dimensions $1\times \mathcalligra{m}$.

According to the Eq. (15), if ${\mathcal{F}}_{\mathcalligra{i}}^{\mathcal{H}\mathcalligra{i}\mathcalligra{p}\mathcalligra{p}\mathcalligra{o}\mathcal{R}}$ is greater than $\mathcal{F}$, it means that the hippopotamus has been hunted and another hippopotamus will replace it in the herd, otherwise the hunter will escape and this hippopotamus will return to the herd. Significant enhancements were observed in the global search process during the second phase. The first and second phases complement each other and effectively mitigate the risk of getting trapped in local minima.

15 $${\chi }_{\mathcalligra{i}}=\left\{\begin{array}{c}{{\chi }_{\mathcalligra{i}}}^{\mathcal{H}\mathcalligra{i}\mathcalligra{p}\mathcalligra{p}\mathcalligra{o}\mathcal{R}}{\mathcal{F}}_{\mathcalligra{i}}^{\mathcal{H}\mathcalligra{i}\mathcalligra{p}\mathcalligra{p}\mathcalligra{o}\mathcal{R}}<{\mathcal{F}}_{\mathcalligra{i}}\\ {\chi }_{\mathcalligra{i}} {\mathcal{F}}_{\mathcalligra{i}}^{\mathcal{H}\mathcalligra{i}\mathcalligra{p}\mathcalligra{p}\mathcalligra{o}\mathcal{R}}\ge {\mathcal{F}}_{\mathcalligra{i}}\end{array}\right.$$

##### Phase 3: Hippopotamus Escaping from the Predator (Exploitation)

**Figure 3:**
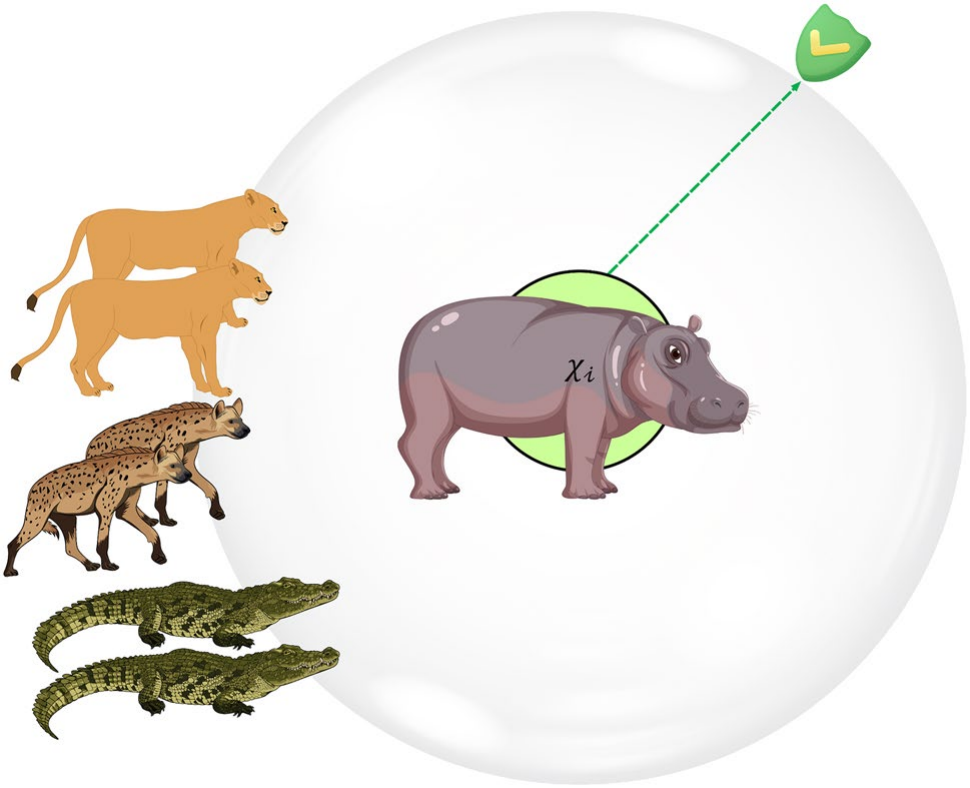
Drawing a Hippopotamus Escaping from the Predator.

Another behavior of a hippopotamus in the face of a predator is when the hippopotamus encounters a group of predators or is unable to repel the predator with its defensive behavior. In this situation, the hippopotamus tries to move away from the area (Fig. 3). Usually, the hippopotamus tries to run to the nearest lake or pond to avoid the harm of predators because spotted lions and hyenas avoid entering the lake or pond. This strategy leads to the hippopotamus finding a safe position close to its current location and modelling this behavior in Phase Three of the HO results in an enhanced ability for exploitation in local search. To simulate this behavior, a random position is generated near the current location of the hippopotamuses. This behavior of the hippopotamuses is modelled according to Eqs. (16–19). When the newly created position improves the cost function value, it indicates that the hippopotamus has found a safer position near its current location and has changed its position accordingly. $\mathcalligra{t}$ denotes the current iteration, while $\mathcal{T}$ represents the MaxIter.

16 $${\mathcalligra{l}\mathcalligra{b}}_{\mathcalligra{j}}^{\mathcalligra{l}\mathcalligra{o}\mathcalligra{c}\mathcalligra{a}\mathcalligra{l}}=\frac{{\mathcalligra{l}\mathcalligra{b}}_{\mathcalligra{j}}}{\mathcalligra{t}}, {\mathcalligra{u}\mathcalligra{b}}_{\mathcalligra{j}}^{\mathcalligra{l}\mathcalligra{o}\mathcalligra{c}\mathcalligra{a}\mathcalligra{l}}=\frac{{\mathcalligra{u}\mathcalligra{b}}_{\mathcalligra{j}}}{\mathcalligra{t}}, \mathcalligra{t}=\mathrm{1,2},\dots ,\mathcal{T}$$

17 $${{\chi }_{\mathcalligra{i}}}^{\mathcal{H}\mathcalligra{i}\mathcalligra{p}\mathcalligra{p}\mathcalligra{o}\mathcal{E}}:{\mathcalligra{x}}_{\mathcalligra{i},\mathcalligra{j}}^{\mathcalligra{H}\mathcalligra{i}\mathcalligra{p}\mathcalligra{p}\mathcalligra{o}\mathcal{E}}={\mathcalligra{x}}_{\mathcalligra{i},\mathcalligra{j}}+{r}_{10.}.\left({\mathcalligra{l}\mathcalligra{b}}_{\mathcalligra{j}}^{\mathcalligra{l}\mathcalligra{o}\mathcalligra{c}\mathcalligra{a}\mathcalligra{l}}+{\mathcalligra{s}}_{1}.\left({\mathcalligra{u}\mathcalligra{b}}_{\mathcalligra{j}}^{\mathcalligra{l}\mathcalligra{o}\mathcalligra{c}\mathcalligra{a}\mathcalligra{l}}-{\mathcalligra{l}\mathcalligra{b}}_{\mathcalligra{j}}^{\mathcalligra{l}\mathcalligra{o}\mathcalligra{c}\mathcalligra{a}\mathcalligra{l}}\right)\right)$$

$$\mathcalligra{i}=\mathrm{1,2},\dots ,\mathcal{N} ,\mathcalligra{j}=\mathrm{1,2},\dots ,\mathcalligra{m}$$

In Eq. (17), ${{\chi }_{\mathcalligra{i}}}^{\mathcalligra{H}\mathcalligra{i}\mathcalligra{p}\mathcalligra{p}\mathcalligra{o}\mathcalligra{E}}$ is the position of hippopotamus which was searched to find the closest safe place. ${\mathcalligra{s}}_{1}$ is a random vector or number that is randomly selected from among three scenarios $\mathcalligra{s}$ Eq. (18). The considered scenarios ($\mathcalligra{s}$) lead to a more suitable local search or, in other words, result in the proposed algorithm having a higher exploitation quality.

18 $$\mathcalligra{s}=\left\{\begin{array}{c}2\times {\overrightarrow{r}}_{11}-1\\ {r}_{12}\\ {r}_{13}\end{array}\right.$$

In Eq. (18) ${\overrightarrow{r}}_{11}$ represents a random vector between 0 and 1, while ${r}_{10}$ (Eq. 17) and ${r}_{13}$ denote random numbers generated within the range of 0 and 1. Additionally, ${r}_{12}$ is a normally distributed random number.

19 $${\chi }_{\mathcalligra{i}}=\left\{\begin{array}{c}{{\chi }_{\mathcalligra{i}}}^{\mathcal{H}\mathcalligra{i}\mathcalligra{p}\mathcalligra{p}\mathcalligra{o}\mathcal{E}}{\mathcal{F}}_{\mathcalligra{i}}^{\mathcal{H}\mathcalligra{i}\mathcalligra{p}\mathcalligra{p}\mathcalligra{o}\mathcal{E}}<{\mathcal{F}}_{\mathcalligra{i}}\\ {\chi }_{\mathcalligra{i}} {\mathcal{F}}_{\mathcalligra{i}}^{\mathcal{H}\mathcalligra{i}\mathcalligra{p}\mathcalligra{p}\mathcalligra{o}\mathcal{E}}\ge {\mathcal{F}}_{\mathcalligra{i}}\end{array}\right.$$

In the HO algorithm to update the population, we did not divide the population into three separate categories of immature, female, and male hippopotamus because although dividing them into separate categories would be better modelling of their nature, it would reduce the performance of the optimization algorithm.

#### Repetition process, and flowchart of HO

After completing each iteration of the HO algorithm, all population members are updated based on Phases 1 to 3 this process of updating the population according to Eqs. (3–19) continues until the final iteration.

**Figure 4:**
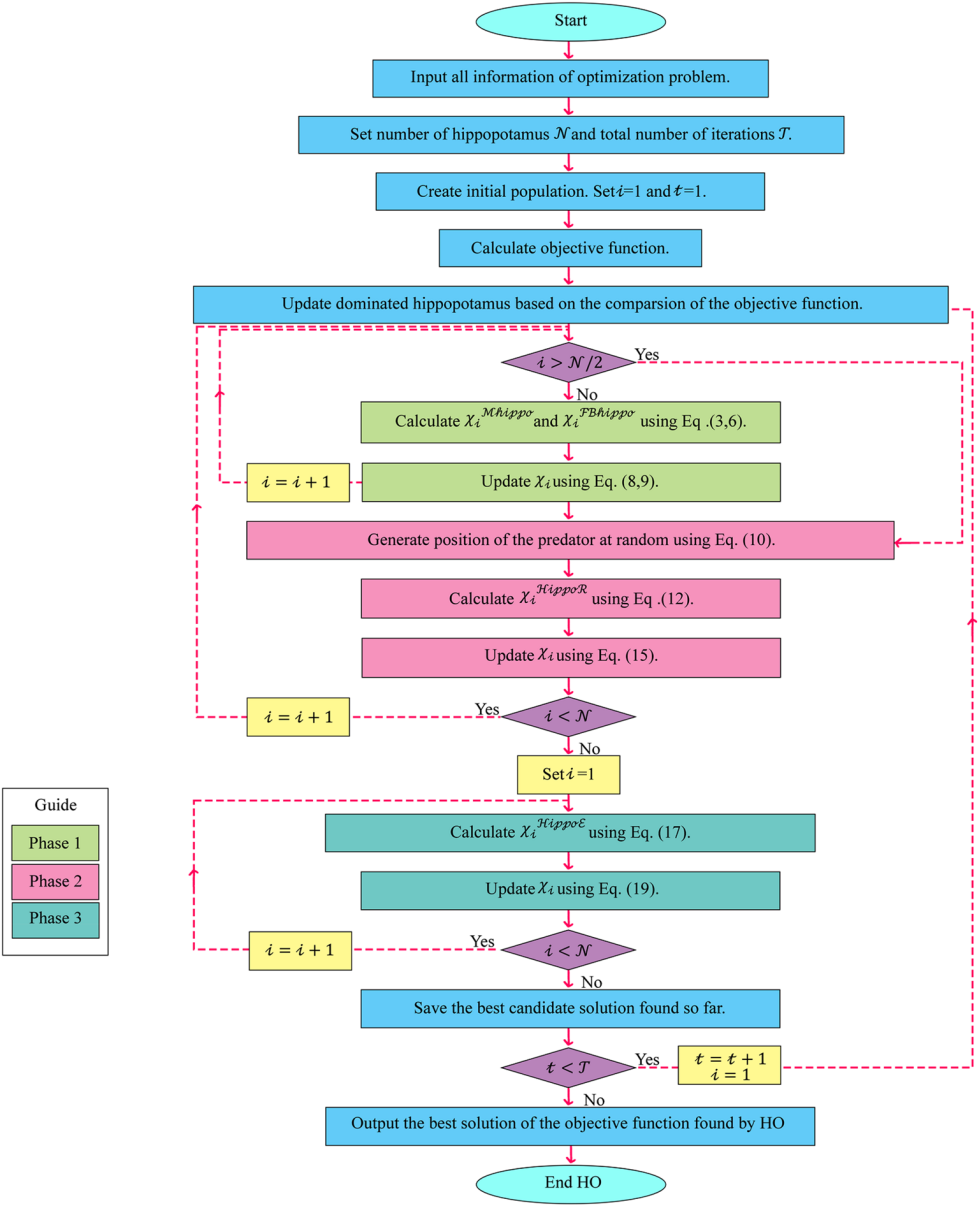
HO's flowchart.

During the execution of the algorithm, the best potential solution is consistently tracked and stored. Upon the completion of the entire algorithm, the best candidate, referred to as the dominant hippopotamus solution, is unveiled as the ultimate solution to the problem. The HO's procedural details are shown in Fig. 4 flowchart and Algorithm 1's pseudocode.

**Algorithm 1:**
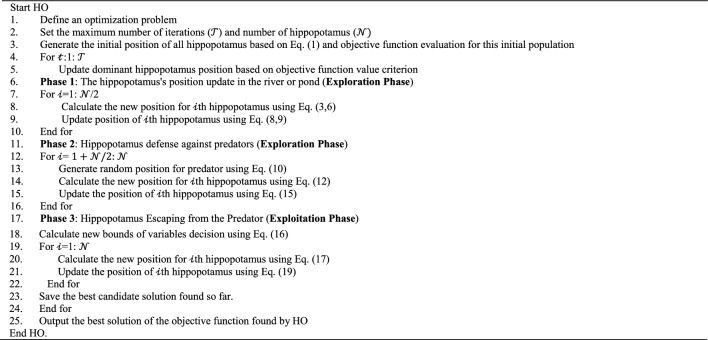
Pseudo-code of HO.

#### Computational complexity of HO

In this subsection, the HO computational complexity analysis is discussed. The total computational complexity of HO is equal to $\mathcal{O}\left(\mathcal{N}\mathcalligra{m}\left(1+\frac{5\times \mathcal{T}}{2}\right)\right).$ The $\mathcal{N}\mathcalligra{m}$ represents the computational complexity of the initial assignment of the algorithm, which is the same for all metaheuristic optimization algorithms. The computational complexity of the initial phase in HO is denoted as $\mathcal{N}\mathcalligra{m}\mathcal{T}$. The computational complexity of the second phase in HO is $\frac{\mathcal{N}\mathcalligra{m}\mathcal{T}}{2}$. Finally, the computational complexity of the third phase is $\mathcal{N}\mathcalligra{m}\mathcal{T}$. Therefore, the total computational complexity of the main loop is $\mathcal{N}\mathcalligra{m}\frac{5\times \mathcal{T}}{2}.$

Regarding competitor algorithms, WOA, GWO, SSA**,** PSO, SCA, FA, GOA, CMA-ES, SSA, MFO, and IWO have a time complexity equal to $\mathcal{O}\left(\mathcal{N}\mathcalligra{m}\left(1+\mathcal{T}\right)\right)$ and TLBO and AOA have a computational complexity equal to $\mathcal{O}\left(\mathcal{N}\mathcalligra{m}\left(1+2\mathcal{T}\right)\right).$ Nevertheless, in order to ensure equitable comparative analysis, we standardized the population size for each algorithm within the simulation study, thereby ensuring uniformity in the total count of function evaluations across all algorithms utilized. Other algorithms with higher time complexity were introduced, for instance, CGO, which exhibits a computational complexity of $\mathcal{O}\left(\mathcal{N}\mathcalligra{m}\left(1+4\mathcal{T}\right)\right)$.

#### Limitation of HO

The initial constraint of the HO, akin to all metaheuristic algorithms, lies in the absence of assurance regarding attaining the global optimum due to the stochastic search procedure. The second constraint stems from the NFL, implying the perpetual potential for newer metaheuristic algorithms to outperform HO. A further constraint involves the inability to assert HO as the preeminent optimizer across all optimization endeavors.

### Simulation results and comparison

In this study, we juxtapose the efficacy of results attained through HO with a dozen established metaheuristic algorithms such as SCA, GWO, WOA, GOA, SSA, FA, TLBO, CMA-ES, IWO, MFO, AOA, and PSO. The adjustment of control parameters is detailed as per the specifications outlined in Table 1. This section presents simulation studies of the HO applied to various challenging optimization problems. The effectiveness of the HO in achieving optimal solutions is evaluated using a comprehensive set of 161 standard BFs. These functions encompass UM, high-dimensional, FM, and the CEC 2014, CEC 2019, ZP, and 4 engineering problems.

**Table 1:** Assigned values to the control parameters of competitor algorithms.

Algorithm	Parameter	Value
GWO	Convergence parameter ($a$)	Linear reduction from 2 to 0
SCA	A	2
WOA	Convergence parameter ($a$)	Linear reduction 2 to 0
	Parameter $r$	A random vector between 0 and 1
	Parameter $l$	A random vector between -1 and 1
PSO	Velocity limit	10% of dimension range
	Cognitive and social constant	(${\text{C}}_{1}\text{,}{\text{C}}_{2}\text{)=(2,2)}$
	Topology	Fully connected
	Inertia weight	Linear reduction from 0.9 to 0.1
GOA	$l$	1.5
	$f$	0.5
	${c}_{min}$	0.00004
	${c}_{max}$	1
SSA	Initial speed (${v}_{0}$)	0
	Leader position update probability	0.5
FA	Alpha ($\alpha$)	0.2
	Beta ($\beta$)	1
	Gamma ($\gamma$)	1
TLBO	Teaching factor (${T}_{F}$)	${\text{round}}\left(\text{1+rand}\right)$
	$Rand$	A random number between 0 and 1
CMA-ES	${\sigma }^{(0)}$	0.5
	$\mu$	$\left\lfloor {\lambda \backslash 2} \right\rfloor$
AOA	$a$	0
	$\mu$	0.5
IWO	Minimum number of seeds (${S}_{min}$)	0
	Maximum number of seeds (${S}_{max}$)	5
	Initial value of standard deviation	1
	Final value of standard deviation	0.001
	Variance reduction exponent	2
MFO	b	1
	r	Linear reduction -1 to -2

To enhance the performance of functions F1 to F23^43^, CEC 2019 test set, ZP, and engineering problems algorithms 30 independent runs encompassing 30,000 NFE and 60,000 NFE for CEC 2014 test set. The HO's population number is maintained at a constant of 24 members for AOA and TLBO set 30 and other algorithms is 60, and the MaxIter is set on 500 and 1000 (CEC 2014). A comprehensive set of six statistical metrics, namely mean, best, worst, Std., median, and rank, are utilized for presenting the optimization outcomes. The mean index is particularly employed as a pivotal ranking parameter for evaluating the efficacy of metaheuristic algorithms across each BF.

The specifications of the software and machines used for simulation are as follows; Core (TM) i3-1005G1 CPU processor with 1.20GHz with 8G for main memory and MacBook Air M1 with 8G for main memory.

#### Evaluation Unimodal benchmark functions

The assessment of functions was conducted, and the outcomes are presented in Table 2. Figure 6, shows convergence of the three most effective algorithms for optimizing F1-F23. This evaluation is to determine the ability of the algorithms to local search on seven separate UM functions, shown as F1-F7. The HO achieved global optimum for F1-F3 and F5-F6 a feat unattained by any of the 12 algorithms subjected to evaluation. Its performance in optimizing the F4 surpassed the others significantly. In a competitive scenario involving the F6, global optimum was achieved alongside four additional algorithms. Lastly, noteworthy superiority in performance was demonstrated by the HO for the F7. HO has consistently converged to zero Std. for F1- F4 and F6. For F7, the Std. is 4.10E-05, while for F5, it stands at 0.36343. The HO has the lowest Std. compared to the investigated algorithms.

**Table 2:** Evaluation outcomes for the objectives specified in the F1-F7.

F	M	Optimization algorithms												
		HO	WOA	GWO	SSA	PSO	SCA	FA	GOA	TLBO	CMA-ES	MFO	AOA	IWO
F1	Mean	0	6.25E-121	5.73E-88	2.71E-07	3.69E-06	14.855	9712.8	3.92E-07	1.24E-89	3.2243e-08	672.36	9.07E-13	1292.5
	Best	0	1.41E-132	1.92E-93	2.22E-08	1.11E-07	0.012079	2686.8	1.33E-08	1.95E-91	1.5314e-08	0.71101	4.77E-160	3.4203
	Worst	0	1.71E-119	9.11E-87	2.09E-06	6.56E-05	77.5	15,976	4.57E-06	7.75E-89	7.6176e-08	10,009	2.72E-11	4731.2
	Std	0	3.11E-120	1.78E-87	4.40E-07	1.19E-05	20.848	2903	8.76E-07	1.55E-89	1.36e-08	2536.9	4.97E-12	1101.6
	Median	0	2.31E-124	6.66E-90	9.95E-08	7.37E-07	4.6546	9605.9	1.47E-07	7.33E-90	3.1564e-08	2.7663	3.04E-81	1019.3
	Rank	1	2	4	7	9	10	13	8	3	6	11	5	12
F2	Mean	0	2.11E-69	3.54E-51	2.011	0.0034028	0.013089	796.13	137.97	4.19E-45	0.00025096	27.865	7.60E-209	0.10835
	Best	0	2.47E-74	1.43E-54	0.13287	6.72E-05	0.00023745	5.1074	62.113	2.42E-46	0.00013828	0.11195	1.88E-259	0.04844
	Worst	0	4.26E-68	2.19E-50	5.6365	0.049467	0.070743	19,630	476.13	1.15E-44	0.0004223	80.013	2.28E-207	0.19507
	Std	0	7.87E-69	6.11E-51	1.7389	0.0091893	0.017935	3561.4	66.627	3.09E-45	7.04e-05	21.195	0	0.033356
	Median	0	2.45E-71	8.41E-52	1.4766	0.000676	0.0048081	118.56	129.11	3.40E-45	0.00025537	25.302	1.36E-233	0.10201
	Rank	1	3	4	10	7	8	13	12	5	6	11	2	9
F3	Mean	0	21,814	9.45E-20	1514	162.1	7903.2	17,097	704.4	5.07E-18	0.023561	19,119	0.0075389	9501.9
	Best	0	1.1848	1.47E-31	312.01	36.17	224.73	7098.1	2.3208	6.80E-21	0.0023696	3189.6	2.59E-126	2067.5
	Worst	0	1.09E + 05	2.24E-18	4288.7	399.71	24,159	28,712	5014.4	9.53E-17	0.090665	42,334	0.047224	25,025
	Std	0	23,342	4.14E-19	949.06	89.026	5848.9	4911.1	1718.9	1.72E-17	0.023315	10,697	0.011884	4795.7
	Median	0	16,593	6.59E-24	1177.9	154.08	6901.6	16,339	18.233	1.20E-18	0.015358	19,243	3.59E-12	8431.1
	Rank	1	13	2	8	6	9	11	7	3	5	12	4	10
F4	Mean	1.43E-217	54.69	1.95E-13	12.346	2.828	35.686	42.732	22.677	1.30E-36	0.0020537	67.677	0.027967	37.301
	Best	9.84E-255	0.69808	2.42E-17	5.8528	0.97132	13.438	28.564	5.5781	1.24E-37	0.0010508	49.754	9.60E-54	26.965
	Worst	3.01E-216	95.783	3.37E-12	20.052	6.9104	64.384	51.977	41.254	5.75E-36	0.0039077	83.333	0.046479	50.889
	Std	0	30.606	6.27E-13	3.2297	1.3593	12.293	6.0607	9.2042	1.35E-36	0.00061183	9.6399	0.019333	5.2665
	Median	4.02E-233	60.163	1.08E-14	11.993	2.4615	33.964	43.936	21.496	8.93E-37	0.0018613	69.183	0.040356	38.063
	Rank	1	12	3	7	6	9	11	8	2	4	13	5	10
F5	Mean	0.12111	27.601	27.591	212.69	43.819	53,121	8585.4	51,037	25.425	56.719	2.68E + 06	28.5	145.47
	Best	0	26.905	26.211	22.487	5.8924	43.934	30.427	0.24725	24.579	20.528	185.69	27.613	23.25
	Worst	1.9637	28.553	28.8	1243	119.87	3.25E + 05	41,425	1.02E + 06	26.293	684.86	8.00E + 07	28.916	1692.8
	Std	0.36343	0.39245	0.72478	285.55	33.794	92,441	11,144	2.05E + 05	0.39027	127.83	1.46E + 07	0.29675	314.02
	Median	0.0070966	27.627	27.202	85.715	25.626	6262.6	3219	87.009	25.42	22.307	880.08	28.522	29.201
	Rank	1	4	3	9	6	12	10	11	2	7	13	5	8
F6	Mean	0	0	0	19.1	4.5	17.067	21,561	1.0333	0	0	1727.8	0	3023.2
	Best	0	0	0	6	0	0	9654	0	0	0	1	0	502
	Worst	0	0	0	32	37	139	28,728	4	0	0	10,225	0	6159
	Std	0	0	0	7.4987	7.2099	31.139	4301	1.3257	0	0	3791.4	0	1649.4
	Median	0	0	0	19	1.5	6	22,142	0.5	0	0	13.5	0	2818.5
	Rank	1	1	1	8	6	7	11	5	2	3	9	4	10
F7	Mean	3.54E-05	0.0046721	0.0016763	0.18146	0.024313	0.1112	0.076687	117.49	0.0011331	0.011562	4.3902	5.80E-05	0.071947
	Best	1.30E-06	0.00013219	0.00016558	0.067404	0.0094839	0.018044	0.035773	63.606	0.0004299	0.005156	0.065606	1.76E-06	0.029085
	Worst	0.00013102	0.015981	0.0083233	0.3183	0.055549	0.89506	0.15281	183.52	0.0023231	0.017513	77.983	0.00033704	0.12335
	Std	4.10E-05	0.0047918	0.0014997	0.070721	0.011822	0.16168	0.029595	30.728	0.00050432	0.0032379	14.338	7.42E-05	0.020096
	Median	1.99E-05	0.002365	0.0013396	0.17233	0.020326	0.064266	0.073503	118.49	0.0009457	0.012149	0.28247	2.67E-05	0.070357
	Rank	1	5	4	11	7	10	9	13	3	6	12	2	8
Sum rank		7	40	21	60	47	65	78	64	20	37	81	27	67
Mean rank		1	5.7143	3	8.5714	6.7143	9.2857	11.1429	9.1429	2.8571	5.2857	11.5714	3.8571	9.5714
Total rank		1	6	3	8	7	10	12	9	2	5	13	4	11

#### Evaluation benchmark function high-dimensional multimodal

The outcomes of F8-F13 which were HM function using algorithms are presented in Table 3. The objective behind choosing these functions was to assess algorithm’s global search capabilities. The HO outperformed all other algorithms in F8 by a significant margin. In F9, it achieved global optimum along with the WOA, which indicates outstanding performance compared to other algorithms. For F10, it outperformed all other algorithms. F11 converged to global optimum alongside the TLBO, demonstrating superior performance compared to other algorithms. In F12, GOA outperformed HO and TLBO and ranked first. In F13, HO obtained the first rank. For F8, the HO's Std. is notably lower than the investigated algorithms. The F13 Std. is 0.012164, the lowest after the CMA-ES algorithm. This suggests that the HO demonstrates resilience in effectively addressing these functions (Fig. 6).

**Table 3:** Evaluation outcomes for the objectives specified in the F8-F13.

F	M	Optimization algorithms												
		HO	WOA	GWO	SSA	PSO	SCA	FA	GOA	TLBO	CMA-ES	MFO	AOA	IWO
F8	Mean	− 12,567	− 10,876	− 5568	− 7033.5	− 6590.1	− 3734.5	− 7463.7	− 9483.9	− 7906.9	− 4363.9	− 8496.8	− 5340.9	-6695.5
	Best	− 12,569	− 12,569	− 6952.5	− 8479.5	− 8325.1	− 4553.8	− 8678.6	− 10,608	− 9427.3	− 5177.9	− 9778.5	− 6242.2	-8233.1
	Worst	− 12,530	− 8345.5	− 3461	− 6099.1	− 4337.3	− 3362.8	− 6488.8	− 8278.2	− 5915.7	− 3860.7	− 6725.5	− 4587.8	-4759.4
	Std	7.3469	1729.4	941.59	640.28	903.25	281.89	615.82	556.59	781.93	320.79	863.55	471.37	677.46
	Median	− 12,569	− 11,715	− 5432.2	− 6845.4	− 6489.3	− 3679.7	− 7454.6	− 9477.9	− 8002.1	− 4301.5	− 8559.5	− 5316.7	-6646.7
	Rank	1	2	10	7	9	13	6	3	5	12	4	11	8
F9	Mean	0	0	4.9095	52.899	45.735	50.849	186.92	202.22	12.924	126.97	155.49	0	65.852
	Best	0	0	0	23.879	22.884	0.03564	117.41	113.44	0	6.9667	84.588	0	43.819
	Worst	0	0	27.59	80.591	78.602	202.58	258.69	280.58	23.007	187.18	228.14	0	95.563
	Std	0	0	8.2235	17.219	14.675	48.636	33.884	37.356	6.0126	71.067	40.991	0	12.894
	Median	0	0	5.68-14	56.713	45.271	38.413	187.05	204.35	13.042	162.64	152.47	0	64.752
	Rank	1	1	2	6	4	5	10	11	3	8	9	1	7
F10	Mean	4.44-16	4.00-15	1.51-14	2.7311	1.1408	14.229	18.997	19.035	9.21-15	5.6832-05	13.321	4.44-16	10.679
	Best	4.44-16	4.44-16	7.55-15	1.6462	6.26-05	0.050121	18.271	18.094	4.00-15	3.4412-05	0.68917	4.44-16	0.0087287
	Worst	4.44-16	7.55-15	2.18-14	5.6781	2.4083	20.402	19.296	19.438	1.03-13	9.5468-05	19.962	4.44-16	19.288
	Std	0	2.47-15	3.20-15	0.81204	0.82655	8.6665	0.21681	0.26647	1.79-14	1.5424-05	7.836	0	9.4921
	Median	4.44-16	4.00-15	1.47-14	2.4519	1.3404	20.204	19.028	19.088	7.55-15	5.4007-05	17.837	4.44-16	18.181
	Rank	1	3	5	8	7	11	12	13	4	6	10	2	9
F11	Mean	0	0.012321	0.014884	0.018205	0.021824	0.91439	163.94	0.027115	0	2.9979-07	6.9724	0.1554	480.41
	Best	0	0	0	0.00077716	4.46-07	0.025341	71.221	7.06-05	0	8.804-08	0.43489	0.00044758	333.51
	Worst	0	0.23384	0.15444	0.0526	0.087692	1.6975	237.77	0.11136	0	7.7197-07	91.085	0.43829	640.31
	Std	0	0.048624	0.033114	0.011469	0.026358	0.42847	36.504	0.028254	0	1.7512-07	22.846	0.11095	71.029
	Median	0	0	0	0.016869	0.0098613	0.99061	163.4	0.01876	0	2.6344-07	1.0093	0.13784	477.65
	Rank	1	3	4	5	6	9	11	7	1	2	10	8	12
F12	Mean	9.30-09	0.020187	0.1456	7.0883	0.11094	40,328	42.51	4.4816	0.0034654	1.9945-09	17.719	0.51896	8.8769
	Best	1.49-09	0.0024683	0.034516	1.4549	6.84-09	0.79446	13.932	1.37-10	6.74-09	7.8685-10	0.70708	0.41734	3.4841
	Worst	7.32-08	0.14187	0.31253	16.042	1.0405	7.11E + 05	76.246	14.181	0.10367	7.2421-09	285.16	0.61102	12.625
	Std	1.62-08	0.025008	0.067403	3.1194	0.23009	1.47E + 05	15.85	3.6815	0.018926	1.3528-09	50.987	0.050388	1.8974
	Median	5.33-09	0.013983	0.14025	6.8009	2.09-05	17.858	44.103	3.5971	1.14-07	1.6197-09	6.8694	0.527	8.9038
	Rank	2	4	6	9	5	13	12	8	3	1	11	7	10
F13	Mean	0.0050467	0.44897	1.4596	18.841	0.021928	6.69E + 05	44,205	0.49606	0.072491	2.128-08	2.73E + 07	2.81	0.0027154
	Best	1.35-32	0.14522	0.80982	0.033744	1.03-08	2.7393	50.302	1.80-10	2.23-06	7.311-09	2.1321	2.6101	5.00E-05
	Worst	0.063492	1.3863	1.8467	62.222	0.28572	1.31E + 07	3.97E + 05	4.9234	0.20724	4.3763-08	4.10E + 08	2.9954	0.011275
	Std	0.012164	0.27245	0.21244	15.746	0.05445	2.56E + 06	86,550	1.2302	0.069667	9.3747-09	1.04E + 08	0.092163	0.0047482
	Median	0.0014522	0.35799	1.4967	18.36	0.010988	1130.3	2772.3	0.010987	0.047853	1.9344-08	29.131	2.7955	0.00014517
	Rank	3	6	8	10	4	12	11	7	5	1	13	9	2
Sum rank		9	19	35	45	35	63	62	49	21	30	57	38	48
Mean rank		1.5	3.1667	5.8333	7.5000	5.8333	10.5	10.3333	8.1667	3.5	5	9.5	6.3333	8
Total rank		1	2	5	8	6	13	12	10	3	4	11	7	9

#### Evaluation fixed-dimension multimodal benchmark function

The objective was to examine the algorithm’s capacity to achieve a harmonious equilibrium between exploration and exploitation while conducting the search procedure on F14-F23. Results are reported in Table 4. HO performed best for F14-F23. The HO achieves a significantly lower Std. especially for F20-F22. The findings suggest that HO, characterized by its strong capability to balance exploration and exploitation, demonstrates superior performance when addressing FM and MM functions.

**Table 4:** Evaluation outcomes for the objectives specified in the F14-F23.

F	M	Optimization algorithms												
		HO	WOA	GWO	SSA	PSO	SCA	FA	GOA	TLBO	CMA-ES	MFO	AOA	IWO
F14	Mean	0.998	3.0928	6.9207	1.1305	5.5195	2.2512	9.502	12.782	0.998	4.7816	2.51	8.0876	11.358
	Best	0.998	0.998	0.998	0.998	0.998	0.998	0.998	0.998	0.998	1.992	0.998	0.998	0.998
	Worst	0.998	10.763	12.671	1.992	12.671	10.763	21.988	23.809	0.998	11.721	10.763	12.671	23.809
	Std	0	3.2723	4.9187	0.34368	3.0682	1.8878	6.2553	6.298	3.86-16	2.4391	2.3156	4.7721	7.3331
	Median	0.998	1.495	7.3657	0.998	5.9288	2.0092	8.3574	12.671	0.998	3.9742	0.998	10.763	10.763
	Rank	1	6	9	3	8	4	11	13	2	7	5	10	12
F15	Mean	0.00030836	0.00088694	0.0083127	0.0027545	0.0024923	0.0010454	0.0058617	0.010286	0.00036839	0.0019	0.0013293	0.0071685	0.0027859
	Best	0.00030749	0.00030755	0.00030749	0.00033385	0.00030749	0.00057375	0.00030749	0.00030749	0.00030749	0.0011	0.00074582	0.00034241	0.00058505
	Worst	0.00031288	0.0035616	0.056621	0.02043	0.020363	0.0016389	0.020363	0.056543	0.0012232	0.0035	0.0083337	0.069975	0.020363
	Std	1.31-06	0.00071966	0.012961	0.0059824	0.0060732	0.00035949	0.0089054	0.012603	0.00018223	7.25-06	0.0013978	0.014639	0.0059637
	Median	0.00030779	0.0005872	0.00045554	0.00077266	0.00030782	0.00087851	0.00030749	0.0029582	0.00030749	0.0016	0.00080207	0.00047392	0.00074539
	Rank	1	3	12	8	7	4	10	13	2	6	5	11	9
F16	Mean	− 1.0316	− 1.0316	− 1.0316	− 1.0316	− 1.0316	− 1.0316	− 0.8956	− 0.79107	− 1.0316	− 1.0316	− 1.0316	− 1.0316	− 1.0316
	Best	− 1.0316	− 1.0316	− 1.0316	− 1.0316	− 1.0316	− 1.0316	− 1.0316	− 1.0316	− 1.0316	− 1.0316	− 1.0316	− 1.0316	− 1.0316
	Worst	− 1.0316	− 1.0316	− 1.0316	− 1.0316	− 1.0316	− 1.0314	− 0.21546	2.1043	− 1.0316	− 1.0316	− 1.0316	− 1.0316	− 1.0316
	Std	5.96-16	3.09-09	9.67-09	2.66-14	6.71-16	5.70-05	0.30937	0.62776	6.65-16	6.78-16	6.78-16	1.22-07	1.44-08
	Median	− 1.0316	− 1.0316	− 1.0316	− 1.0316	− 1.0316	− 1.0316	− 1.0316	− 1.0316	− 1.0316	− 1.0316	− 1.0316	− 1.0316	− 1.0316
	Rank	1	1	1	1	1	1	2	3	1	1	1	1	1
F17	Mean	0.39789	0.3979	0.39789	0.39789	0.39789	0.40008	0.39789	0.39789	0.39789	0.39789	0.39789	0.41197	0.39789
	Best	0.39789	0.39789	0.39789	0.39789	0.39789	0.3979	0.39789	0.39789	0.39789	0.39789	0.39789	0.39813	0.39789
	Worst	0.39789	0.39794	0.39789	0.39789	0.39789	0.40747	0.39789	0.39789	0.39789	0.39789	0.39789	0.4415	0.39789
	Std	0	1.42-05	1.41-06	4.70-14	7.23-16	0.0023394	3.86-11	0	0	0	0	0.01286	5.57-09
	Median	0.39789	0.39789	0.39789	0.39789	0.39789	0.39939	0.39789	0.39789	0.39789	0.39789	0.39789	0.40712	0.39789
	Rank	1	2	1	1	1	3	1	1	1	1	1	4	1
F18	Mean	3	3.0001	3	3	3.9	3.0001	3.9	9.3	3	3	3	7.7674	3
	Best	3	3	3	3	3	3	3	3	3	3	3	3	3
	Worst	3	3.0006	3.0001	3	30	3.0004	30	84	3	3	3	37.986	3
	Std	1.27-15	0.00013473	1.79-05	2.77-13	4.9295	0.00010081	4.9295	16.904	5.53-16	1.35-15	1.62-15	10.92	8.47-07
	Median	3	3	3	3	3	3	3	3	3	3	3	3	3
	Rank	1	2	1	1	3	2	3	5	1	1	1	4	1

F	M	Optimization algorithms												
		HO	WOA	GWO	SSA	PSO	SCA	FA	GOA	TLBO	CMA-ES	MFO	AOA	IWO
F19	Mean	− 3.8628	− 3.8335	− 3.8595	− 3.8628	− 3.8628	− 3.8542	− 3.8628	− 3.8212	− 3.8628	− 3.8628	− 3.8628	− 3.8512	-3.8628
	Best	− 3.8628	− 3.8628	− 3.8628	− 3.8628	− 3.8628	− 3.8621	− 3.8628	− 3.8628	− 3.8628	− 3.8628	− 3.8628	− 3.8605	-3.8628
	Worst	− 3.8628	− 3.0885	− 3.8549	− 3.8628	− 3.8628	− 3.8443	− 3.8628	− 3.0898	− 3.8628	− 3.8628	− 3.8628	− 3.8408	-3.8628
	Std	2.70-15	0.14079	0.0036479	1.51-11	6.42-07	0.0032649	5.72-11	0.14271	2.71-15	2.71-15	2.71-15	0.0038724	6.32-07
	Median	− 3.8628	− 3.8611	− 3.8624	− 3.8628	− 3.8628	− 3.8542	− 3.8628	− 3.8627	− 3.8628	− 3.8628	− 3.8628	− 3.8516	− 3.8628
	Rank	1	5	2	1	1	3	1	6	1	1	1	4	1
F20	Mean	− 3.322	− 3.2406	− 3.1218	− 3.2275	− 3.2784	− 2.8823	− 3.2586	− 2.7966	− 3.2811	− 3.2919	− 3.2422	− 3.0755	− 3.203
	Best	− 3.322	− 3.3219	− 3.322	− 3.322	− 3.322	− 3.1473	− 3.322	− 3.3138	− 3.3206	− 3.322	− 3.322	− 3.1844	− 3.2031
	Worst	− 3.322	− 2.4308	− 2.2671	− 3.1469	− 3.2031	− 1.9133	− 3.2031	− 1.9056	− 3.1059	− 3.2031	− 3.1376	− 2.9507	− 3.2026
	Std	9.78-12	0.17522	0.26562	0.058906	0.058273	0.32598	0.060328	0.36014	0.066162	0.051459	0.063837	0.063759	0.00012908
	Median	− 3.322	− 3.3191	− 3.1676	− 3.1988	− 3.322	− 2.9914	− 3.2031	− 2.9387	− 3.3109	− 3.322	− 3.2031	− 3.0905	− 3.2031
	Rank	1	7	10	8	4	12	5	13	3	2	6	11	9
F21	Mean	− 10.153	− 7.9465	− 8.6325	− 7.5665	− 6.3165	− 2.1516	− 5.6345	− 5.2171	− 9.778	− 7.9121	− 6.3132	− 3.4351	− 6.6391
	Best	− 10.153	− 10.153	− 10.153	− 10.153	− 10.153	− 6.0051	− 10.153	− 10.153	− 10.153	− 10.153	− 10.153	− 5.5613	− 10.153
	Worst	− 10.153	− 2.6294	− 5.0552	− 2.6305	− 2.6305	− 0.35136	− 2.6305	− 2.6305	− 3.9961	− 2.6829	− 2.6305	− 1.9507	− 2.6305
	Std	4.74-06	2.7979	2.3619	3.5062	3.6985	1.872	3.1766	2.9687	1.4345	3.4819	3.5169	0.97125	3.4561
	Median	− 10.153	− 10.137	− 10.152	− 10.153	− 5.078	− 0.88031	− 5.0552	− 5.0552	− 10.153	− 10.153	− 5.078	− 3.2531	− 5.1008
	Rank	1	4	3	6	8	13	10	11	2	5	9	12	6
F22	Mean	− 10.403	− 6.573	− 9.5722	− 8.8874	− 6.7572	− 2.7098	− 5.3848	− 5.5286	− 9.7141	− 10.403	− 8.2382	− 3.7002	− 7.4415
	Best	− 10.403	− 10.401	− 10.403	− 10.403	− 10.403	− 6.3217	− 10.403	− 10.403	− 10.403	− 10.403	− 10.403	− 6.8593	− 10.403
	Worst	− 10.403	− 1.8372	− 2.7659	− 2.7519	− 1.8376	− 0.52104	− 1.8376	− 1.8376	− 5.0265	− 10.403	− 2.7519	− 1.2708	− 1.8376
	Std	6.16-05	3.2924	2.1838	2.8452	3.7466	1.9244	3.194	3.3448	1.7896	1.65-15	3.3738	1.2624	3.7449
	Median	− 10.403	− 5.0875	− 10.402	− 10.403	− 7.7659	− 2.6079	− 3.7243	− 3.7243	− 10.403	− 10.403	− 10.403	− 3.6181	− 10.403
	Rank	1	9	3	5	8	13	11	10	2	1	6	12	7
F23	Mean	− 10.536	− 6.8188	− 9.2737	− 8.2567	− 6.0645	− 4.1564	− 4.7569	− 5.3577	− 10.123	− 10.536	− 7.9819	− 4.6738	− 8.3548
	Best	− 10.536	− 10.534	− 10.536	− 10.536	− 10.536	− 8.3393	− 10.536	− 10.536	− 10.536	− 10.536	− 10.536	− 8.6767	− 10.536
	Worst	− 10.536	− 1.8588	− 2.4217	− 2.4217	− 1.8595	− 0.94428	− 1.6766	− 1.8595	− 3.8354	− 10.536	− 2.4217	− 1.8573	− 2.4217
	Std	2.99-05	3.4247	2.9055	3.3562	3.7424	1.5765	3.0762	3.5273	1.5801	1.78-15	3.6868	1.5405	3.437
	Median	− 10.536	− 5.1284	− 10.536	− 10.536	− 3.8354	− 4.6344	− 3.8354	− 3.8354	− 10.536	− 10.536	− 10.536	− 4.8892	− 10.536
	Rank	1	8	4	6	9	13	11	10	2	1	7	12	5
Sum rank		10	47	46	40	50	68	65	85	17	26	42	81	52
Mean rank		1	4.7000	4.6000	4	5	6.8000	6.5000	8.5000	1.7000	2.6000	4.2000	8.1000	5.2000
Total rank		1	7	6	4	8	11	10	13	2	3	5	12	9

**Figure 5:**
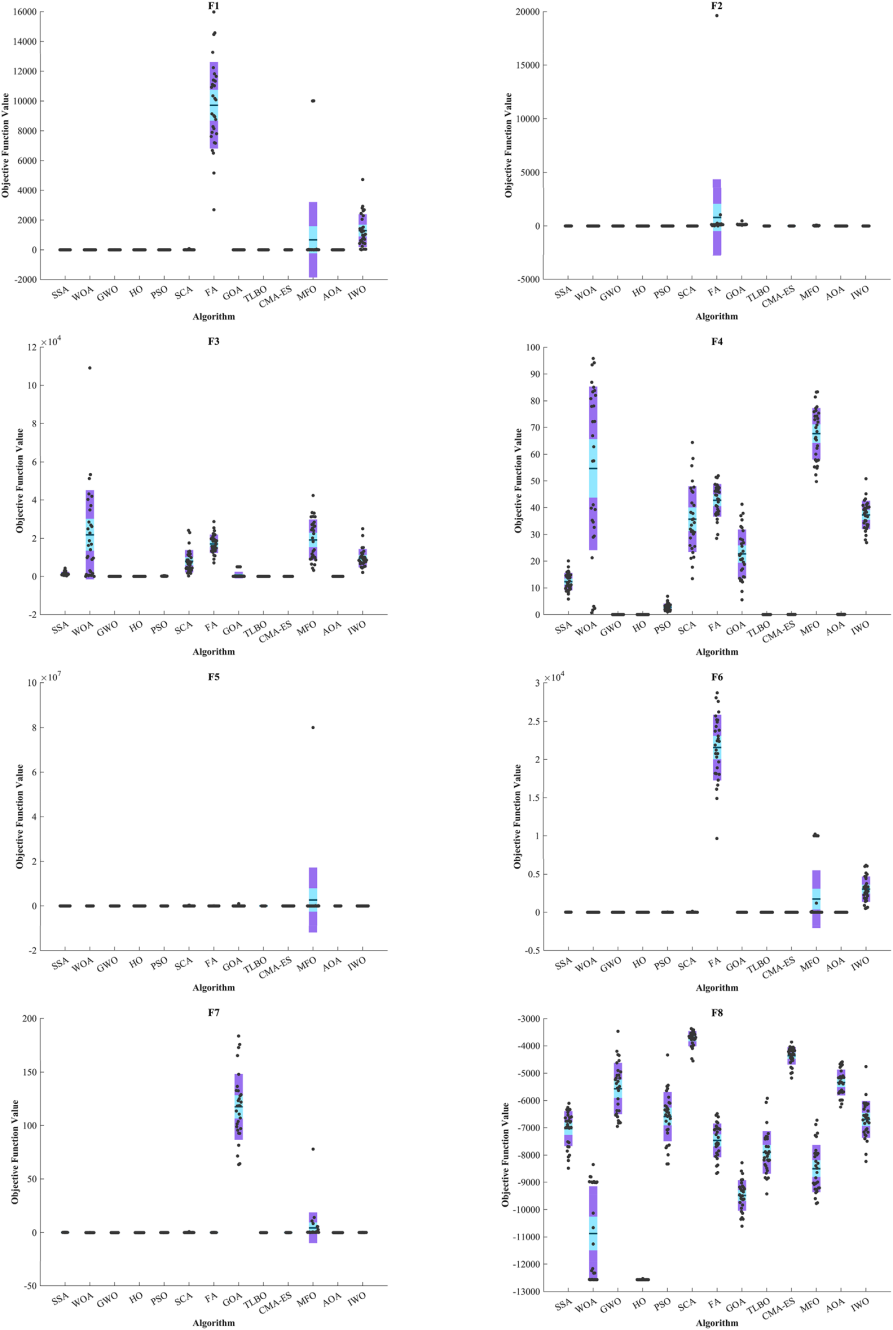

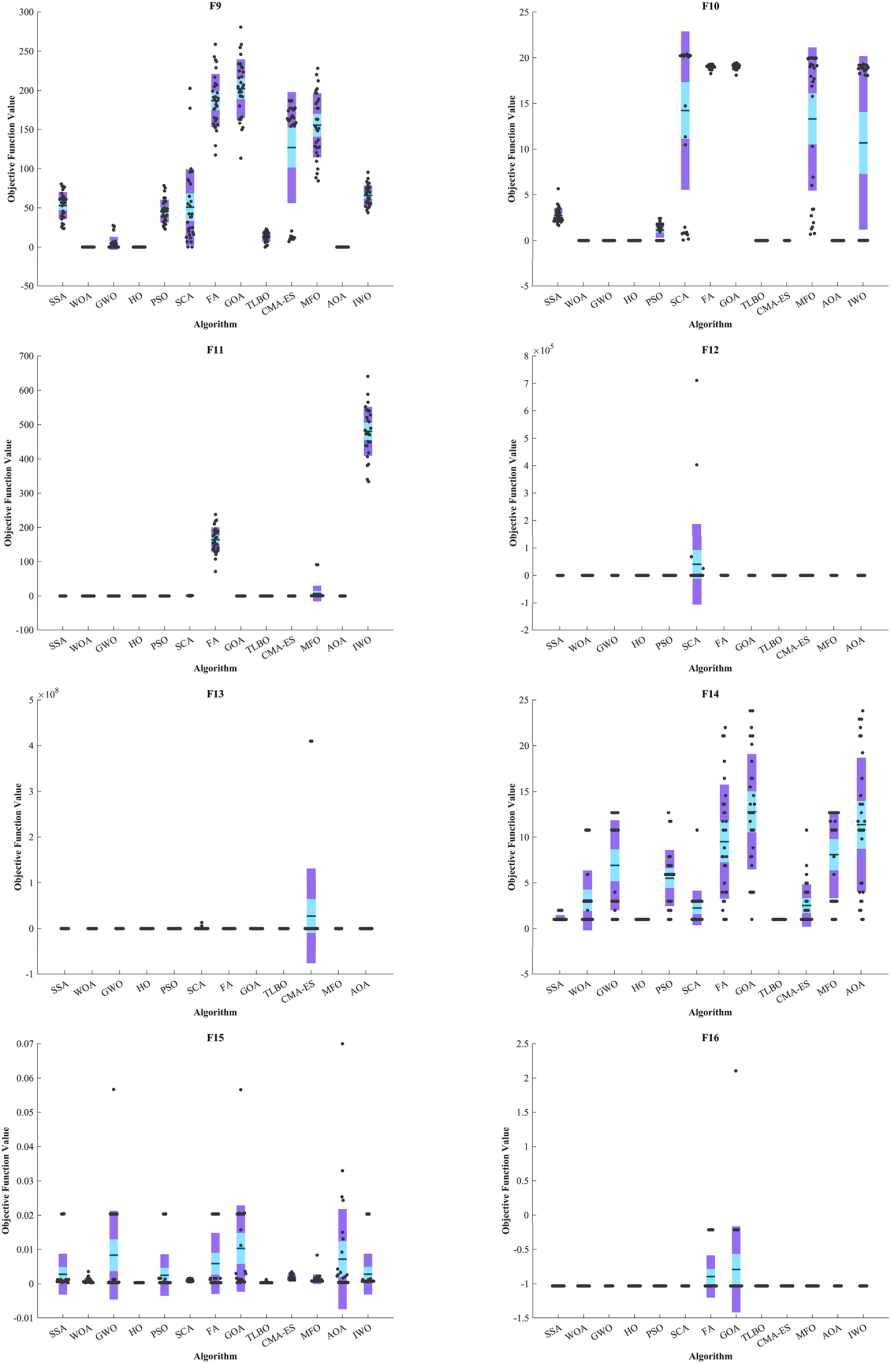

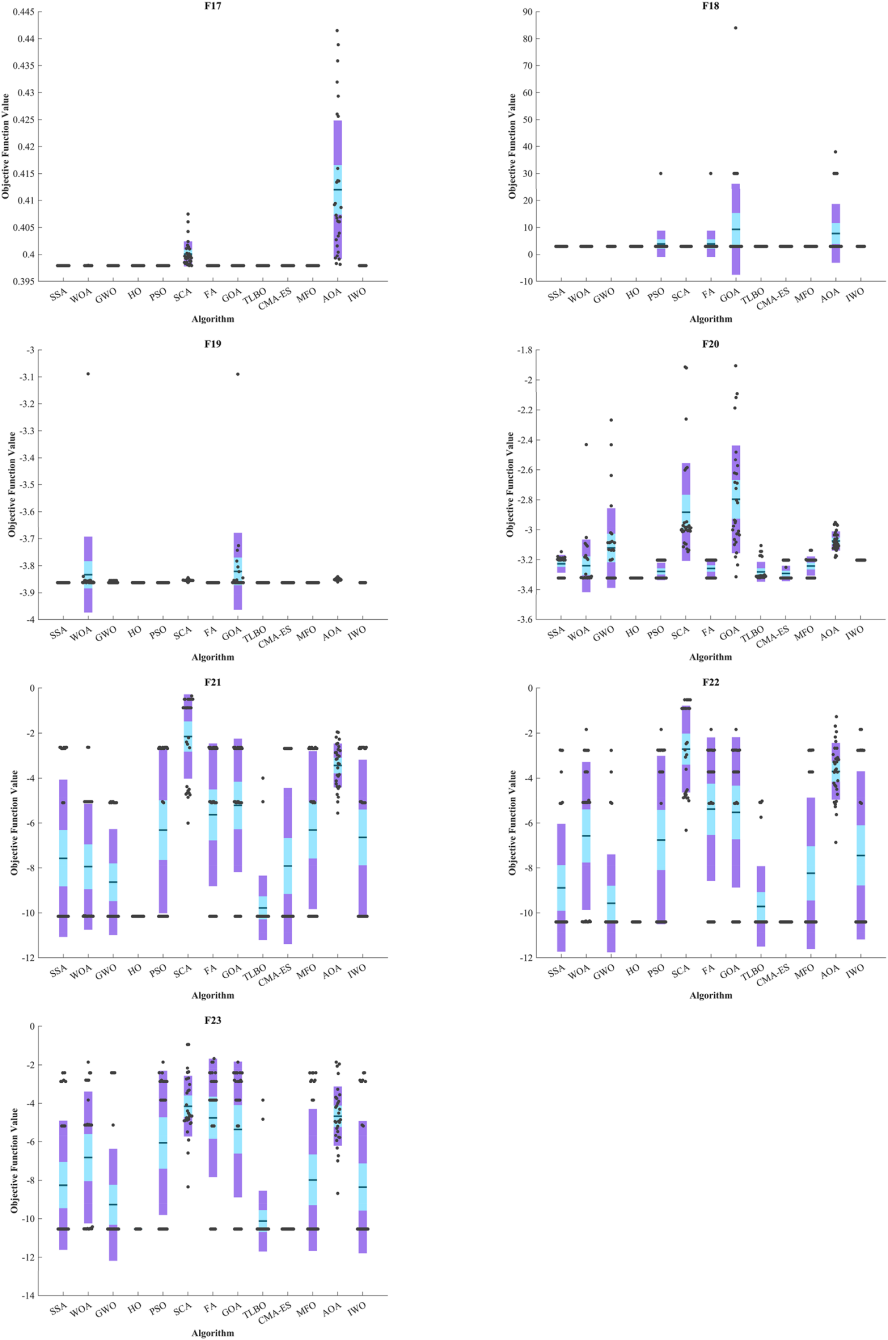
Boxplot illustrating the performance of the HO in comparison to competing algorithms for optimizing BFs (F1-F23).

Figure 5 displays box plot diagrams depicting the optimal values of the objective function obtained from 30 separate runs for F1-F23, utilizing a set of HO and 12 algorithms.

#### Evaluation of the ZP

**Figure 6:**
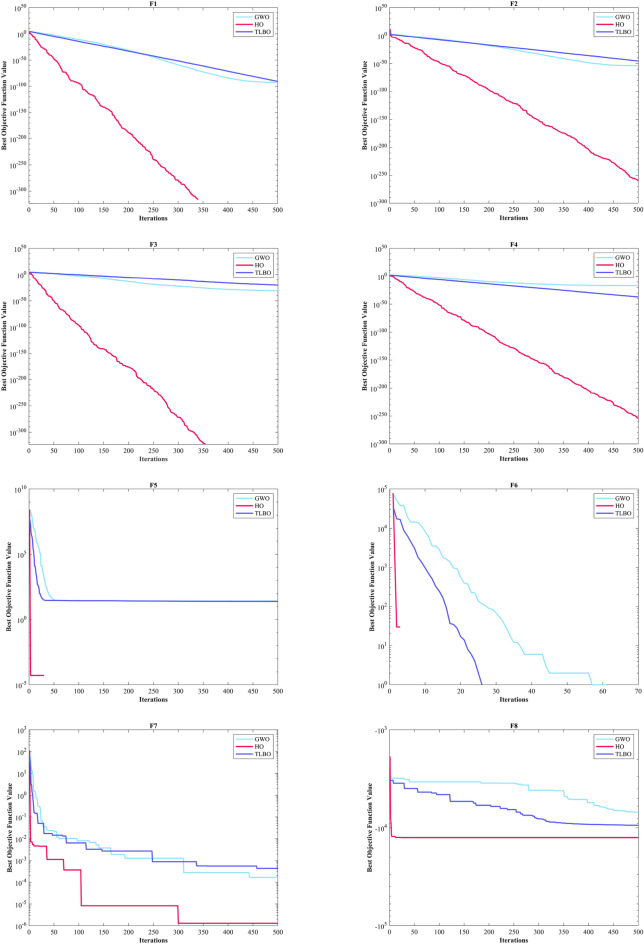

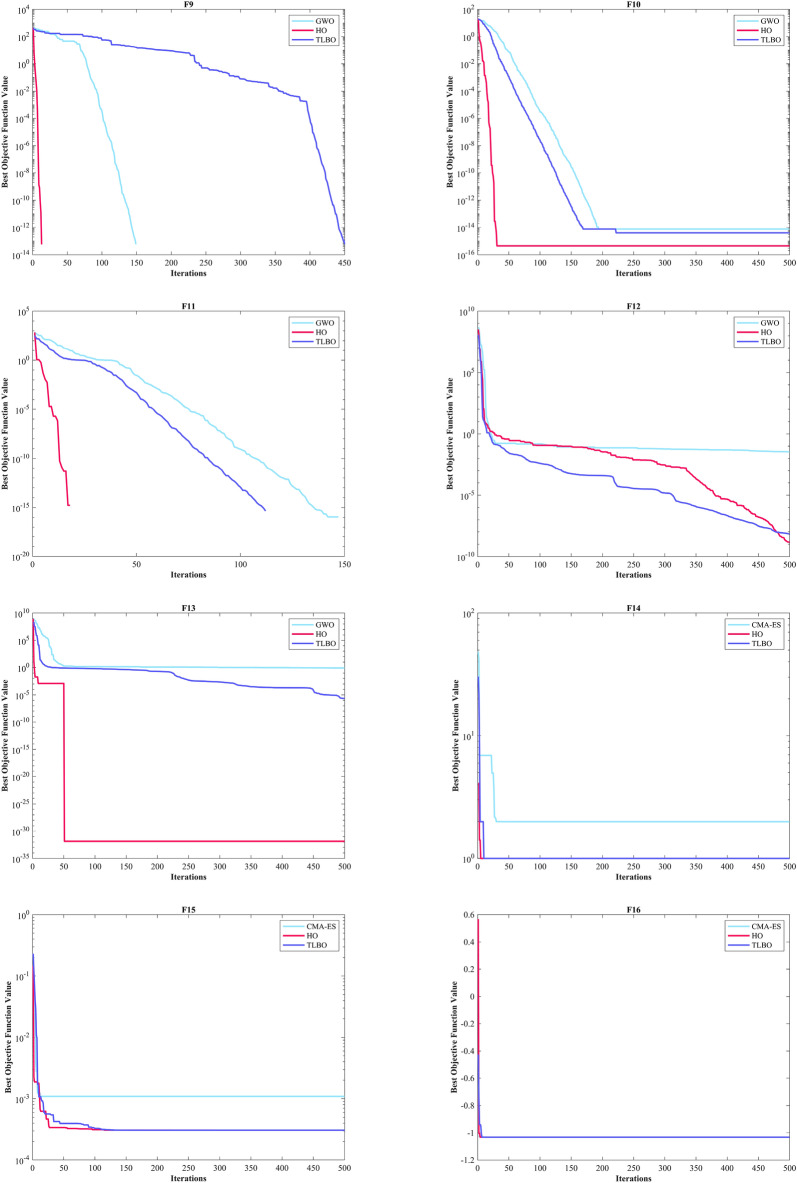

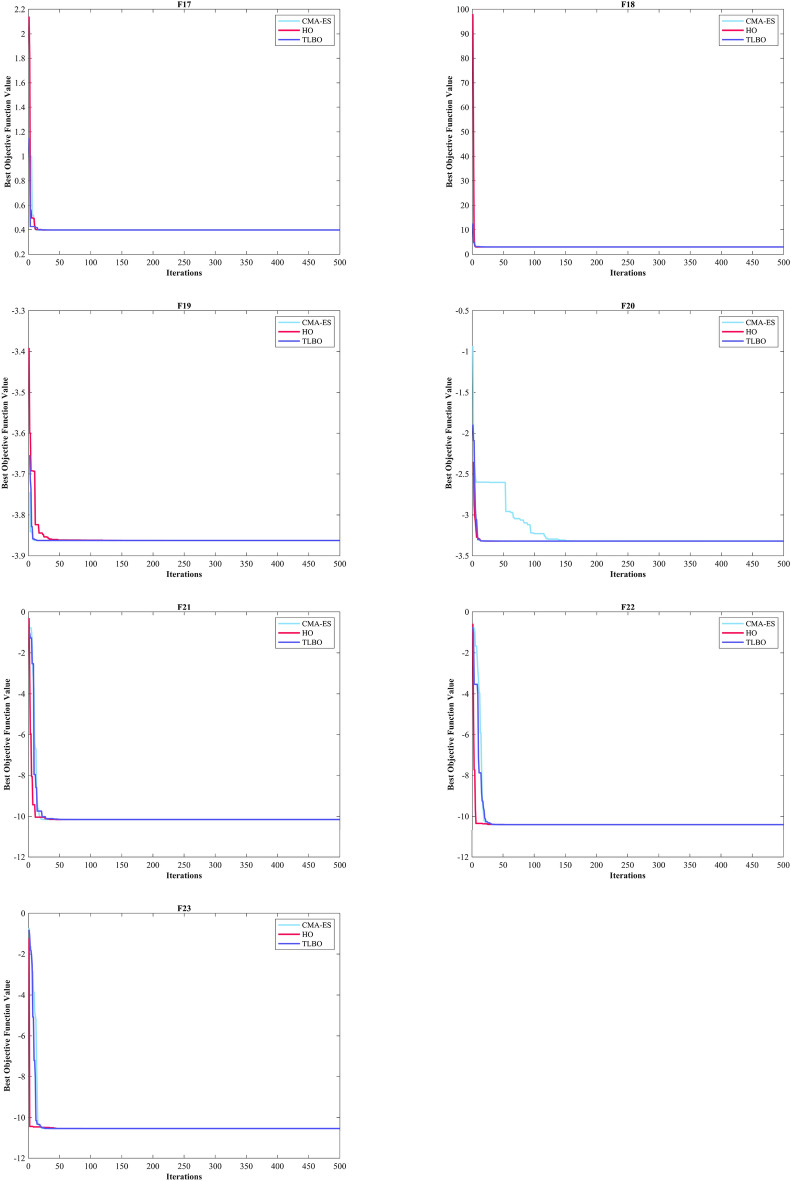
Convergence curves of the top three algorithms in each benchmark functions (F1- F23).

Kudela and Matousek introduced eight novel challenging benchmark functions, presenting a formidable challenge for bound-constrained single-objective optimization. These functions are crafted on the foundation of a ZP characterized by their non-differentiable nature and remarkable multimodality, and introduced functions incorporate three adjustable parameters, allowing for alterations in their behavior and level of difficulty^137^. Table 5 presents the results for eight ZP (ZP-F1 to ZP-F8). In ZP-F1 and ZP-F2, WOA outperformed HO and TLBO and ranked first. The HO exhibited superior performance across ZP-F3 to ZP-F8, achieving global optimum for the objective function in ZP-F3 and ZP-F8. HO outperformed all investigated algorithms for ZP-F3 and ZP-F4. Furthermore, the HO achieved a remarkable result by achieving global optimum for ZP-F5 and ZP-F6 across all criteria. In the case of ZP-F7, HO was in close competition with the GWO algorithm and secured the first rank by achieving global optimum. A similar success was observed for the ZP-F8 function, where HO competed with the AOA algorithm and achieved global optimum (Fig. 6).

**Table 5:** Evaluation outcomes for the objectives specified in the ZP.

F	M	Optimization Algorithms												
		HO	WOA	GWO	SSA	PSO	SCA	FA	GOA	TLBO	CMA-ES	MFO	AOA	IWO
ZP-F1	Mean	5.81E-66	3.70E-63	126.21	401.46	103.82	599.72	499.05	295.46	7.60E-39	570.29	394.48	0.20819	483.88
	Best	5.47E-72	0	8.54E-27	302.31	72.97	390.51	383.14	227.12	5.90E-40	488.94	326.44	5.30E-05	428.22
	Worst	6.08E-65	9.86E-62	686.76	528.15	132.22	821.23	576.72	414.65	7.28E-38	630.04	529.95	0.59081	574.49
	Std	1.51E-65	1.80E-62	194.58	53.315	16.449	111.11	44.155	46.643	1.55E-38	34.272	45.389	0.16611	39.582
	Median	1.17E-67	3.29E-65	20.172	390.45	100.2	613.96	501.48	290.25	3.14E-39	570.84	388.78	0.1819	478.19
	Rank	1	2	6	9	5	13	11	7	3	12	8	4	10
ZP-F2	Mean	2.86E-65	9.29E-64	0.057452	411.24	167.96	464.66	1.36E-39	946.55	694.82	524.24	673.65	0.24434	919.95
	Best	2.50E-71	0	6.23E-27	326.33	118.88	181.41	3.70E-40	843.24	579.84	328.51	518.82	0.00025022	680.28
	Worst	4.92E-64	1.78E-62	1.7235	500.21	244.4	707.21	5.42E-39	1065.3	808.5	640.74	861.43	0.62708	1094.2
	Std	9.69E-65	3.28E-63	0.31468	40.297	27.671	140.48	1.12E-39	58.929	66.872	63.56	70.097	0.17334	82.853
	Median	3.14E-67	3.24E-65	4.47E-26	407.95	164.04	470.97	9.71E-40	943.5	681.74	543.1	672.55	0.23486	914.94
	Rank	1	2	4	7	6	8	3	13	11	9	10	5	12
ZP-F3	Mean	1.66E-60	1.57E-54	55.432	549.6	305.04	548.83	491.76	543.73	493.46	702.89	387.35	536.12	0.17836
	Best	0	2.26E-70	5.29E-27	520.92	223.48	125.47	474.81	461.05	389.78	674.68	356.77	478.31	6.41E-05
	Worst	1.97E-59	4.25E-53	622.76	572.28	405.51	601.45	502.87	609.37	576.5	640.74	444.61	586.16	0.49694
	Std	4.60E-60	7.77E-54	134.96	13.521	46.236	105	7.7342	33.124	43.534	12.79	18.204	22.927	0.13407
	Median	3.78E-64	3.18E-66	14.392	548.5	306.76	577.19	491.54	548.19	491.79	703.89	386.52	535.44	0.18738
	Rank	1	2	4	12	5	11	7	10	8	13	6	9	3
ZP-F4	Mean	8.84E-52	15.063	13.2	580.13	240.08	281.78	565.75	486.58	485.52	659.77	399.81	0.31674	460.65
	Best	0	3.83E-69	9.80E-27	407.46	168.95	20.976	555.51	422.48	404	633.74	351.62	0.0081653	417.49
	Worst	2.65E-50	220.31	47.416	606.17	308.15	596.39	569.78	564.8	543.55	678.56	467.78	0.63772	526.23
	Std	4.84E-51	46.111	11.422	36.235	34.131	220.52	2.9162	30.404	33.984	10.471	28.016	0.17113	26.368
	Median	1.09E-63	5.73E-66	14.42	588.77	235.18	189.01	566.01	483.73	485.59	661.44	396.32	0.35609	458.52
	Rank	1	4	3	12	5	6	11	10	9	13	7	2	8

F	M	Optimization Algorithms												
		HO	WOA	GWO	SSA	PSO	SCA	FA	GOA	TLBO	CMA-ES	MFO	AOA	IWO
ZP-F5	Mean	0	273.38	464.21	1718.6	1297	1467.7	1766.1	1047.9	1496.1	2480.4	1573.9	11.263	1724.3
	Best	0	0	47.775	1583.7	1148.2	607.85	1527	867.71	1298.4	2380.2	1231.4	3.30E-06	1606.1
	Worst	0	1366.9	1747.3	1955.4	1545	1970.3	1939.1	1465.7	1664.3	2531.1	1793.3	41.375	1856
	Std	0	463.47	531.54	94.576	93.791	358.18	99.787	144.98	80.878	35.674	119.7	10.591	73.998
	Median	0	3.55E-15	195.54	1703.1	1276.3	1599.3	1750.6	1009.5	1480.4	2478	1596.1	8.8444	1720.1
	Rank	1	3	4	10	6	7	12	5	8	13	9	2	11
ZP-F6	Mean	0	2.37E-16	28.129	1301.6	962.94	683.03	1413.4	1364.8	1497.9	2480.4	1384.8	0.0099571	1522.5
	Best	0	0	2.84E-14	1118.9	771.93	171.18	1213.9	1229.3	1117.4	2303.3	1250.7	1.44E-11	1353.3
	Worst	0	3.55E-15	141.92	1530.8	1323.8	1889.9	1559.3	1577.3	1952.8	2500	1499.8	0.13786	1672.1
	Std	0	9.01E-16	44.749	101.86	120.91	404.33	74.498	95.834	191.33	47.699	72.837	0.02644	76.638
	Median	0	0	7.99E-14	1289.6	943.67	619.83	1414.6	1353.6	1482	2445.1	1376.6	0.00019612	1530.1
	Rank	1	2	4	7	6	5	10	8	11	13	9	3	12
ZP-F7	Mean	1.28E-12	66.15	2.30E-10	4009.7	3401.1	204.52	4485.6	4362.2	3278.3	4756.4	3913.5	0.047242	4391.5
	Best	0	0	7.67E-11	3885.1	3250.3	0.38829	4382.1	4273.7	0	4464.1	3831.7	0	4293.2
	Worst	1.92E-11	1984.5	3.93E-10	4148.9	3531.1	1016.7	4604.8	4583.2	4209.2	4864.5	4040.4	1.0943	4556
	Std	4.16E-12	362.32	7.44E-11	70.365	68.54	197.33	47.575	83.923	1162.8	103.69	41.426	0.2002	57.296
	Median	0	0	2.40E-10	4012.9	3405	154.3	4482.8	4329.8	3563.4	4798.3	3916	8.64E-05	4388.1
	Rank	1	4	2	9	7	5	12	10	6	13	8	3	11
ZP-F8	Mean	2.2833	304.89	1805	1193	1686.4	1256.1	1780.1	1680.8	549.37	2522.8	533.77	4.5963	1804.4
	Best	0	273.11	1687.5	1102.7	1366.5	1130.3	1578.5	1451.6	537.65	2425.3	507.73	1.55E-08	1643
	Worst	68.5	429.47	1961.5	1279.8	2049.9	1369.1	2012.8	1824.7	552.32	2574.5	702.13	31.634	2006.8
	Std	12.506	46.077	68.209	53.834	182.4	59.606	103.17	84.747	3.1924	103.69	39.594	8.7473	79.751
	Median	0	277.4	1794.4	1198.8	1680.1	1249	1795	1661.2	549.77	2527.3	523.8	0.0024046	1800.5
	Rank	1	3	12	6	9	7	10	8	5	13	4	2	11
Sum rank		8	22	39	72	49	62	76	71	61	99	61	30	78
Mean rank		1	2.7500	4.875	9	6.1200	7.7500	9.5000	8.8750	7.6250	12.375	7.6250	3.7500	9.7500
Total rank		1	2	4	10	5	8	11	9	6	13	7	3	12

**Figure 7:**
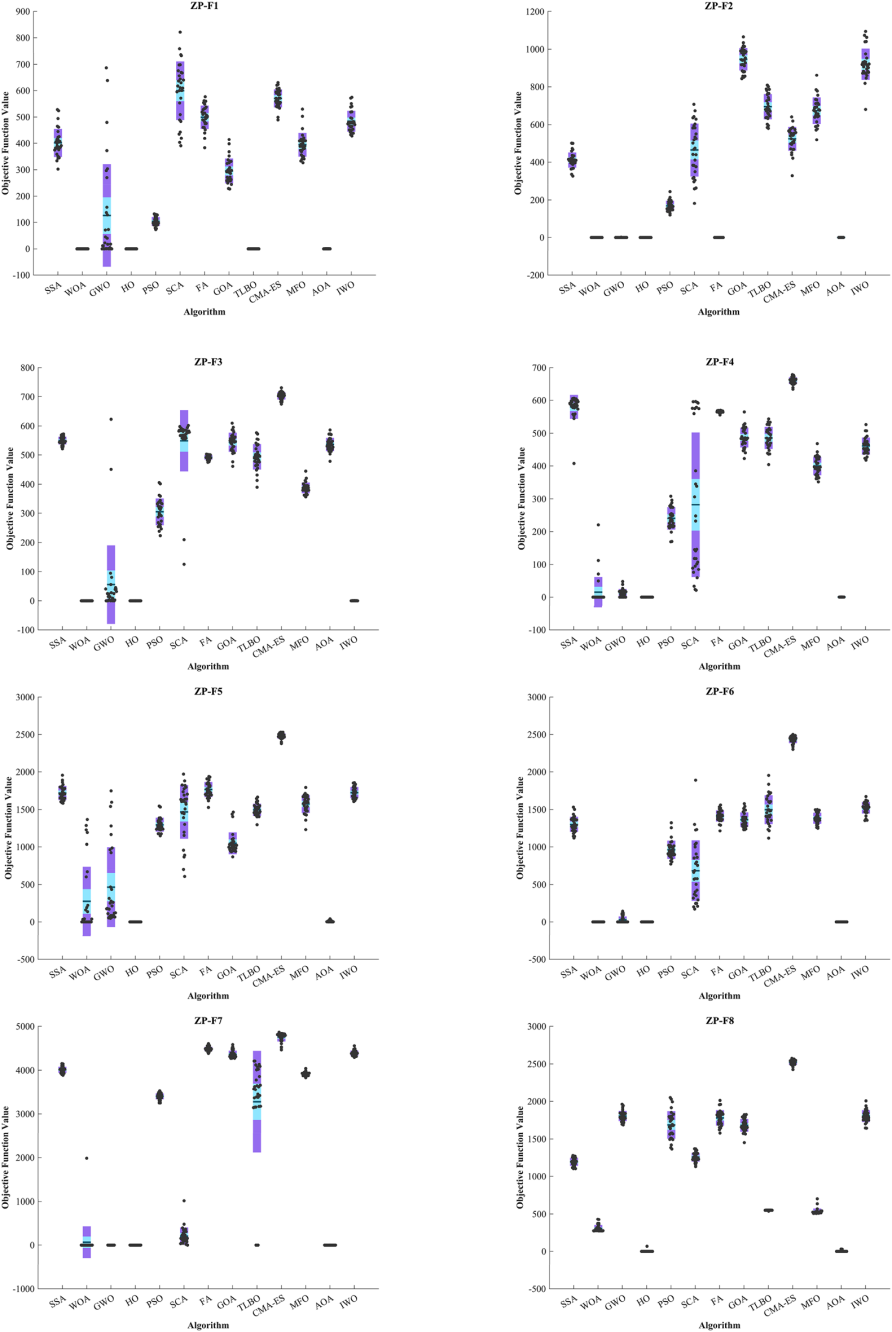
Boxplot illustrating the performance of the HO in comparison to competing algorithms for ZP.

**Figure 8:**
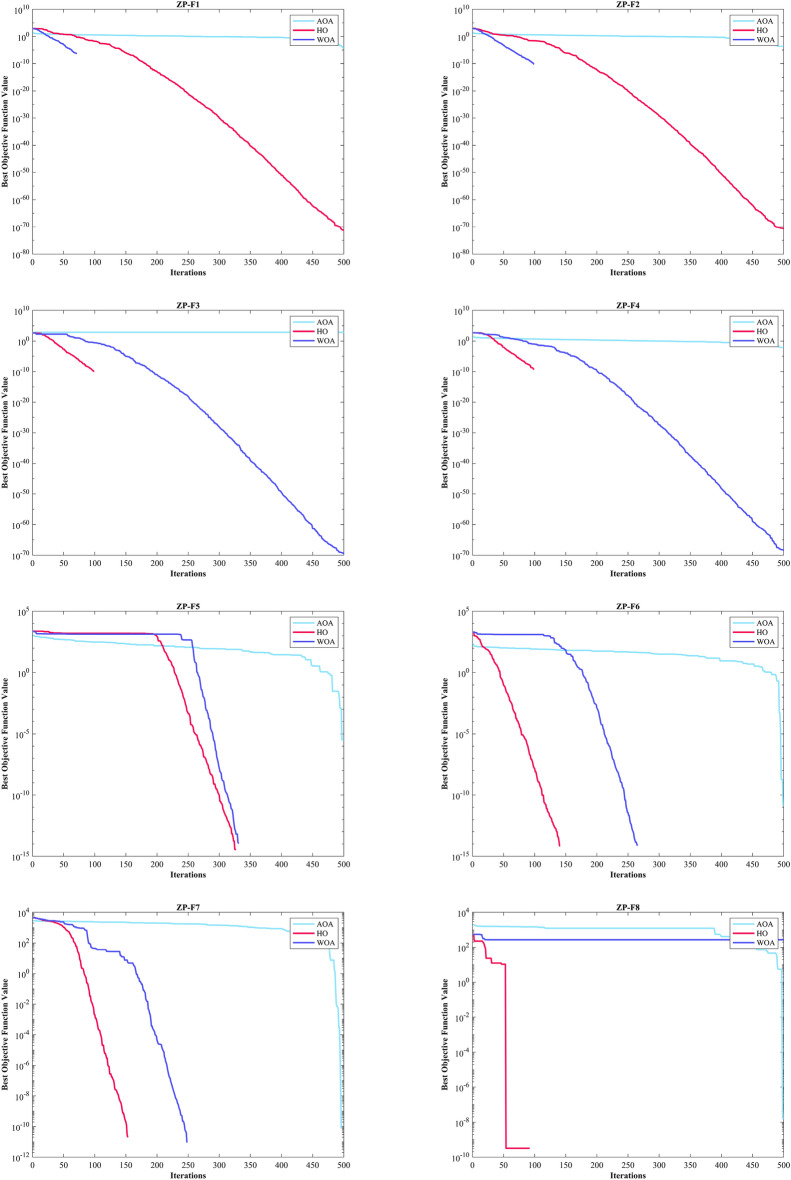
Convergence curves of the top three algorithms in each function in ZP.

In addition, when examining the boxplot diagrams in Fig. 7, it is evident that the HO consistently demonstrated a lower Std. than other algorithms. Figure 8, covering ZP-F1 to ZP-F8, demonstrates that the HO performs much faster than its competitors and reaches an unattainable optimal solution for other investigated algorithms.

#### Evaluation of the CEC 2019 test suite

CEC 2019 test BFs include ten complex functions described in^138^. The details of optimization are reported in Table 6. C19–F1 and C19–F10 functions from the CEC 2019 test designed for single-objective real parameter optimization. They aim to find the best possible outcome globally. These functions are ideal for assessing how well algorithms can perform in a thorough search for the best solution. The HO achieved the top rank in C19-F2–C19-F4 and C19-F7 functions. In C19-F1, it notably outperformed other algorithms across all criteria except the Best criterion Similar outcomes were observed in C19-F2, which ranked first with 3 top algorithms in converges (HO, PSO and SSA). The GWO achieved the top rank in C19-F1. In the case of C19-F3, HO secured the first position with a Std. better than that of the SSA algorithm. For C19-F4, both the Best and Mean criteria demonstrated significantly superior values compared to other algorithms. In C19-F5 CMA-ES surpassed of all algorithms.

**Table 6:** Evaluation outcomes for the objectives specified in the CEC 2019.

F	M	Optimization Algorithms												
		HO	WOA	GWO	SSA	PSO	SCA	FA	GOA	TLBO	CMA-ES	MFO	AOA	IWO
C19-F1	Mean	47,252	1.79E + 10	3.52E + 07	1.06E + 10	5.21E + 08	8.36E + 09	3.87E + 10	3.71E + 10	1.19E + 08	3.5331e + 09	1.75E + 10	2.45E + 09	9.13E + 12
	Best	39,398	8.47E + 05	38,806	5.95E + 08	3.31E + 07	3.43E + 08	1.38E + 09	1.83E + 09	8.13E + 06	4.5975e + 08	6.17E + 08	7.78E + 05	7.70E + 10
	Worst	55,445	1.81E + 11	2.53E + 08	3.42E + 10	2.15E + 09	4.01E + 10	2.76E + 11	1.05E + 11	7.17E + 08	1.875e + 10	1.11E + 11	4.64E + 10	5.93E + 13
	Std	4020	3.93E + 10	7.03E + 07	1.01E + 10	4.90E + 08	9.63E + 09	7.23E + 10	3.20E + 10	1.40E + 08	3.3022e + 09	2.50E + 10	8.61E + 09	1.25E + 13
	Median	46,563	1.79E + 08	1.19E + 06	7.07E + 09	4.16E + 08	4.32E + 09	1.16E + 10	3.22E + 10	8.72E + 07	2.9352e + 09	8.52E + 09	3.49E + 06	5.30E + 12
	Rank	1	10	2	8	4	7	12	11	3	6	9	5	13
C19-F2	Mean	17.343	17.351	17.389	17.345	17.343	17.479	122.78	17.348	17.402	147.07	17.343	19.262	22,426
	Best	17.343	17.344	17.343	17.343	17.343	17.379	17.383	17.343	17.363	43.863	17.343	18.031	11,666
	Worst	17.343	17.37	17.688	17.364	17.343	17.708	1677.2	17.36	17.524	338.8	17.343	19.848	35,735
	Std	7.23E-15	0.0069313	0.11702	0.0046934	9.02E-15	0.068685	401.52	0.004165	0.039382	69.624	7.23E-15	0.46456	6515.5
	Median	17.343	17.349	17.343	17.343	17.343	17.475	17.417	17.347	17.389	142.68	17.343	19.298	23,181
	Rank	1	4	5	2	1	7	9	3	6	10	1	8	11
C19-F3	Mean	12.702	12.702	12.702	12.702	12.702	12.703	12.702	12.703	12.702	12.703	12.702	12.703	12.702
	Best	12.702	12.702	12.702	12.702	12.702	12.702	12.702	12.702	12.702	12.702	12.702	12.702	12.702
	Worst	12.702	12.702	12.702	12.702	12.702	12.703	12.702	12.706	12.702	12.705	12.702	12.705	12.702
	Std	3.61E-15	7.67E-07	1.11E-05	2.90E-13	1.04E-05	0.00010154	6.15E-12	0.001076	8.35E-11	0.00061215	4.23E-06	0.00084308	4.13E-10
	Median	12.702	12.702	12.702	12.702	12.702	12.702	12.702	12.703	12.702	12.702	12.702	12.703	12.702
	Rank	1	1	1	1	1	2	1	2	1	2	1	2	1
C19-F4	Mean	18.374	287.53	535.29	37.543	1845	1462.5	1568.9	18.971	34.367	33.409	329.8	13,356	368.92
	Best	3.9798	141.9	33.824	5.9697	631.72	660.86	398.99	8.9546	9.0757	24.308	7.9597	3823.3	11.94
	Worst	36.813	559.47	3847.9	68.652	7044.6	2675.7	2697.5	35.818	137.3	12.705	2088.2	27,247	1412.2
	Std	8.7939	88.897	954.39	16.365	1384.3	556.95	592.77	7.001	26.315	4.1181	550.46	6225.9	410.04
	Median	18.904	282.78	86.52	36.813	1343.5	1338.1	1642.7	17.909	24.989	33.864	47.26	11,582	193.01
	Rank	1	6	9	5	12	10	11	2	4	3	7	13	8

F	M	Optimization Algorithms												
		HO	WOA	GWO	SSA	PSO	SCA	FA	GOA	TLBO	CMA-ES	MFO	AOA	IWO
C19-F5	Mean	1.0678	1.8316	1.4958	1.2335	1.1233	2.1568	2.2312	4.136	1.2173	1.0014	1.2473	3.673	3.6919
	Best	1.0074	1.193	1.1329	1.0541	1.0246	1.5777	2.0687	1.9503	1.0762	1	1.0394	2.3812	2.0239
	Worst	1.1378	3.2677	1.9381	1.4576	1.3961	3.0567	2.4053	6.1124	1.4825	1.0123	1.8616	5.3353	5.802
	Std	0.0334	0.43639	0.18817	0.10615	0.089297	0.34352	0.10319	0.95669	0.10143	0.0036999	0.1959	0.89047	1.0129
	Median	1.0628	1.7593	1.5061	1.2264	1.096	2.0913	2.2217	4.064	1.1919	1	1.2028	3.627	3.6302
	Rank	2	8	7	5	3	9	10	13	4	1	6	11	12
C19-F6	Mean	1.5425	9.6629	9.0261	4.5374	8.1181	11.021	2.3478	2.9376	10.682	10.892	6.8227	8.991	9.6056
	Best	1.4035	7.3861	5.9608	1.2839	3.9993	9.4881	2.0652	1.0389	9.1723	9.1742	1.1632	7.1545	6.9297
	Worst	1.9837	12.218	12.314	8.7245	11.058	12.525	3.6176	5.5079	12.024	12.219	11.479	10.338	11.957
	Std	0.10476	1.0186	1.5481	1.9199	1.9926	0.60782	0.28102	1.1605	0.73218	0.7937	2.3334	0.83323	1.1387
	Median	1.539	9.7241	9.2959	4.1175	8.6361	11.024	2.2967	2.8617	10.72	11.011	7.4596	9.0147	9.6308
	Rank	1	10	8	4	6	13	2	3	11	12	5	7	9
C19-F7	Mean	29.325	678.73	314.32	351.98	160.5	826.8	474.14	335.56	610.19	214.12	430.55	196.58	309.18
	Best	− 290.8	172.32	87.12	− 136.95	− 139.82	599.1	253.63	− 145.88	125.46	− 72.575	60.249	− 62.577	-56.337
	Worst	198.88	1274	717.83	811.77	412.05	1134.9	915.29	695.39	871.36	1366.4	762.97	711.89	757.85
	Std	93.354	280.85	157.53	241.63	140.99	147.45	181.68	195.98	193.01	339.79	182.54	153.41	191.64
	Median	40.238	784.52	281.51	320.15	191.46	794.08	403.98	337.45	627.49	0.62922	445.9	182.31	301.34
	Rank	1	12	6	8	2	13	10	7	11	4	9	3	5
C19-F8	Mean	4.1966	6.1509	5.2985	5.3343	4.9815	6.1024	5.1356	5.5623	4.3096	6.4078	5.3638	5.6254	5.3825
	Best	2.5485	5.0662	3.4801	3.8021	3.8615	5.3519	4.1766	4.1813	1.8903	2.5595	3.7784	4.3116	4.044
	Worst	5.0156	6.9192	6.5897	6.3892	5.9536	6.7471	5.7968	6.0347	6.0951	7.2601	6.6349	6.544	5.9473
	Std	0.54905	0.5087	0.71219	0.63787	0.6016	0.36842	0.41055	0.4124	1.2012	1.2979	0.71428	0.51051	0.45738
	Median	4.3309	6.204	5.2345	5.3686	5.0262	6.1525	5.2306	5.6443	4.8078	6.7501	5.4298	5.6781	5.4244
	Rank	1	12	5	6	3	11	4	9	2	13	7	10	8
C19-F9	Mean	2.3573	4.8835	28.981	2.6582	2.3639	94.872	2.4128	2.369	65.188	2.4427	2.8046	880.35	2.3694
	Best	2.3429	3.5788	3.3457	2.3651	2.3458	11.304	2.338	2.3452	5.03	2.3787	2.4807	245.71	2.3404
	Worst	2.397	7.0006	367.77	3.1746	2.4212	318.35	3.2355	2.439	446.83	2.5646	3.4282	1758.6	2.4811
	Std	0.012081	0.87027	91.026	0.23252	0.014965	58.664	0.20086	0.018266	102.38	0.041856	0.24161	422.38	0.036775
	Median	2.3548	4.9213	5.0003	2.5526	2.3612	78.066	2.3431	2.3667	28.7	2.4367	2.7476	814.21	2.3554
	Rank	1	9	10	7	2	12	5	3	11	6	8	13	4
C19-F10	Mean	20.299	20.279	20.186	20.065	19.507	20.332	20.001	20.008	19.58	20.49	20.12	20.135	20
	Best	20.029	20.049	20.091	19.999	3.00E− 10	16.849	20	19.999	1.6469	20.164	19.993	20.05	19.999
	Worst	20.533	20.528	20.397	20.307	20.506	20.677	20.001	20.212	20.555	20.682	20.485	20.209	20
	Std	0.11987	0.13818	0.071573	0.095288	3.6879	0.66502	0.00021144	0.038596	3.615	0.11251	0.12782	0.033027	0.00024029
	Median	20.307	20.296	20.17	20.008	20.154	20.449	20.001	20	20.446	20.508	20.084	20.139	20
	Rank	11	10	9	6	1	12	4	5	2	13	7	8	3
Sum rank		21	82	62	52	35	96	68	58	55	70	60	80	74
Mean rank		2.1	8.2000	6.2000	5.2000	3.5000	9.6	6.8	5.8	5.5	7	6	8	7.4
Total rank		1	12	7	3	2	13	8	5	4	9	6	11	10

**Figure 9:**
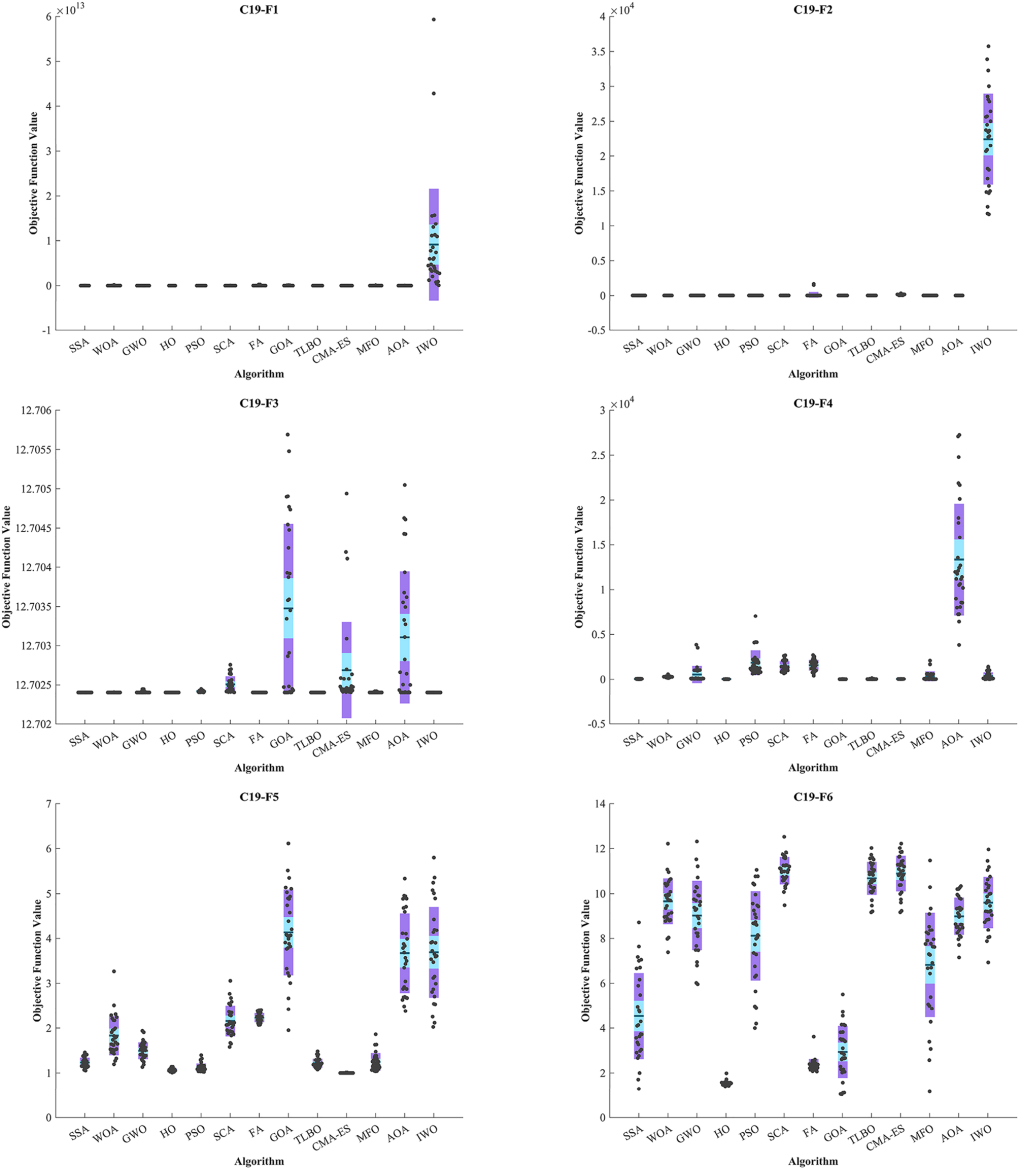

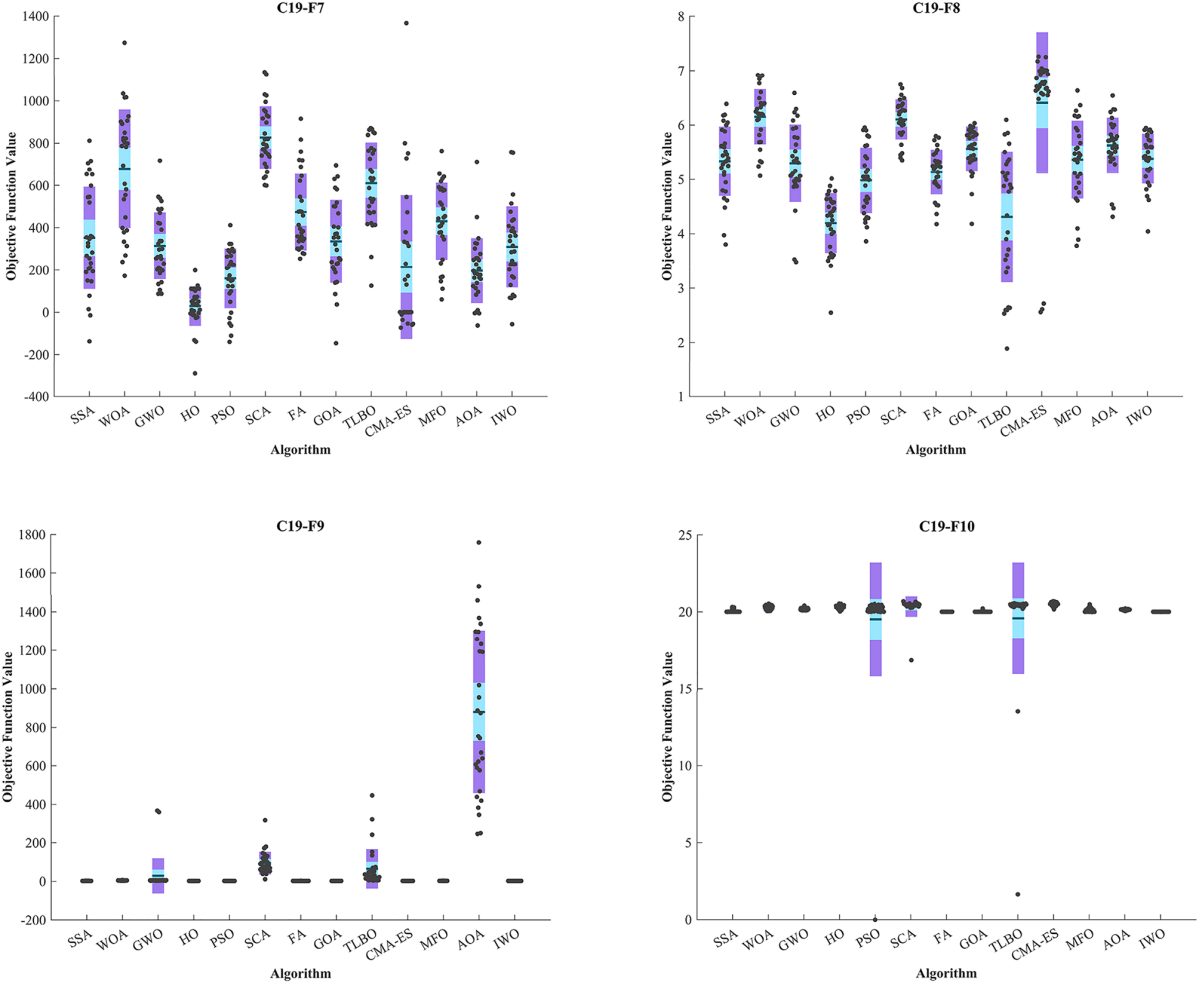
Boxplot illustrating the performance of the HO in comparison to competing algorithms for optimizing CEC 2019.

**Figure 10:**
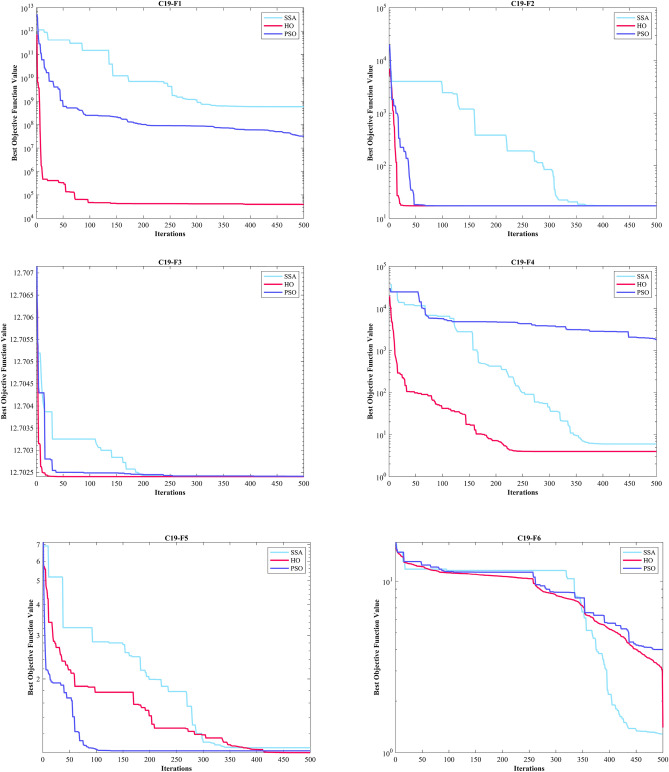

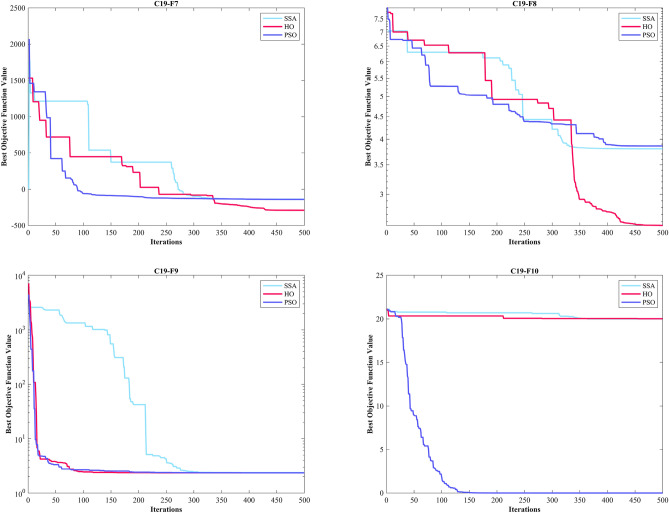
Convergence curves of the top three algorithms in each function in CEC 2019.

The GOA achieved the top rank in C19-F6. In C19-F7 and C19-F9, it surpassed PSO by a slight margin, and in C19-F8 and C19-F10, it had a slight edge over the TLBO, respectively. Notably, in C19-F7, it outperformed PSO by a considerable margin. Finally, in C19-F8, HO emerged as the best across all criteria except the Best criterion while the TLBO found optimal value of C19-F8. In the box plots of Fig. 9, it is obvious that the HO has a dispersion of almost 0 in C19-F1 to C19-F4. Additionally, C19-F5 and C19-F6 have a much lower Std. than investigated algorithms. In the convergence plots of Fig. 10, we observe the excellent performance of the HO in achieving the optimal solution.

#### Evaluation of the CEC 2014 test suite

The CEC 2014 test suite encompasses a total of 30 standard BFs. These functions are categorized into UM functions (C14–F1 to C14–F3), MM functions with subcategories (C14–F4 to C14–F16), hybrid functions (C14–F17 to C14–F22), and composition functions (C14–F23 to C14–F30)^139^. The assessment of the HO is documented for CEC 2014 across varying dimensions (10, 30, 50, and 100) by employing 12 different algorithms. The results of this evaluation are presented in Table S1-S3 within the supplementary, accompanied by graphical representations depicted in Fig. S2-S9, illustrating the boxplots and convergence (The top 3 algorithms) diagrams HO has achieved the first rank in 83 out of 120 functions in finding optimal value. In the function (D = 30), C14-F13 had Std. worse than the first rank algorithm with a difference of 0.1 but better than the known GWO, GOA, and CMA-ES algorithms. The same happened in the functions (D = 50) C14-F13 and (D = 100) C14-F5.

In functions (D = 30) C14-F13, (D = 50) C14-F13, and (D = 100) C14-F5 had a slight difference with the first ranking algorithm only in the Std. value. In the function (D = 50), C14-F29 ranked second compared to the PSO algorithm and was not good in Std. and Best compared to the top 3 algorithms. C14–F4 and C14–F30 present ideal choices for assessing the proficiency of metaheuristic algorithms in local search and exploitation due to their absence of local optima. These functions possess a single extremum, prompting a focal objective of assessing the metaheuristic algorithms' efficacy in converging towards the global optimum during optimization endeavours.

#### Statistical analysis

To thoroughly evaluate the efficacy of the HO, we conduct a comprehensive statistical analysis by comparing it with the reviewed algorithms. The Wilcoxon nonparametric statistical signed-rank test^140^ checks if there's a big difference between pairs of data (See Table 7) It ranks the differences in size (ignoring whether they are positive or negative) and calculates a number based on those ranks. This number helps determine if the differences are likely due to chance or if they're significant. A small p-value means there's likely a big difference between the paired data. A big p-value means we can't be sure there's a significant difference.

**Table 7:** Wilcoxon signed-rank test results.

Algorithms	Functions								
	Unimodal	High-multimodal	Fixed	CEC 2019	ZP	CEC 2014 (10)	CEC 2014 (30)	CEC 2014 (50)	CEC 2014 (100)
HO vs. SSA	1.46E-22	2.38E-14	4.20E-13	4.93E-18	8.86E-20	4.23E-18	3.39E-18	4.12E-18	7.00E-18
HO vs. WOA	1.66E-22	3.19E-09	5.70E-14	1.80E-20	1.35E-17	2.46E-20	4.84E-20	5.80E-21	4.12E-20
HO vs. GWO	1.34E-21	3.09E-06	1.17E-13	2.27E-19	4.89E-18	7.82E-19	1.08E-19	1.17E-19	6.35E-19
HO vs. PSO	1.61E-22	9.78E-10	4.85E-14	6.41E-18	3.61E-18	1.09E-17	3.99E-18	3.24E-17	8.30E-18
HO vs. SCA	1.02E-22	3.99E-15	2.63E-24	2.80E-21	1.42E-19	9.24E-21	1.14E-21	1.08E-21	4.00E-22
HO vs. FA	8.07E-24	2.43E-16	1.27E-18	6.34E-20	2.50E-20	2.04E-19	1.11E-20	4.03E-21	5.14E-21
HO vs. GOA	4.05E-23	3.78E-15	2.41E-26	8.42E-20	1.03E-19	2.22E-18	6.09E-19	4.04E-19	1.19E-18
HO vs. TLBO	3.30E-21	0.17E-5	2.52E-12	5.28E-19	6.88E-19	1.34E-17	3.52E-18	1.00E-17	3.65E-18
HO vs. CMA-ES	5.64E-24	3.74E-17	1.10E-12	5.78E-20	3.84E-21	8.70E-18	3.14E-19	1.06E-20	6.30E-21
HO vs. MFO	5.07E-24	6.95E-19	1.97E-13	4.88E-19	6.07E-19	2.23E-19	7.26E-20	3.36E-20	1.11E-20
HO vs. AOA	6.93E-22	1.60E-08	1.72E-25	9.53E-21	6.91E-18	5.14E-22	1.34E-21	2.94E-21	4.77E-21
HO vs. IWO	2.85E-23	2.57E-15	1.31E-17	2.20E-20	1.08E-20	7.73E-20	1.03E-19	6.96E-20	4.36E-20

The Friedman test is indeed a non-parametric statistical test used to determine if there are statistically significant differences among multiple related groups (Table 8). This research divided the benchmark functions into seven distinct groups to ensure the test's reliability. The initial group consists of functions delineated in Tables 2, 3, 4, encompassing unimodal, multimodal, and composition functions (F1-F23). The second group comprises the category of ZP functions illustrated in Table 5, while the third group is formed by CEC 2019 functions illustrated in Table 6. The fourth, fifth, sixth, and seventh groups included CEC 2014 functions in different dimensions, respectively (Table S1-S3)^141^.

**Table 8:** Friedman mean rank test results.

Algorithms	Functions						
	F1-F23	ZP	CEC 2019	CEC 2014 (10)	CEC 2014 (30)	CEC 2014 (50)	CEC 2014 (100)
HO	2.2468	2.0335	2.1434	2.0226	2.0284	2.0217	2.0256
WOA	4.693	4.966	8.5141	8.8319	7.1239	8.434	6.8774
GWO	4.53	7.1087	7.1867	6.9419	6.5996	7.3247	6.0552
SSA	4.8758	9.5542	6.2015	6.424	5.646	5.9983	6.0104
PSO	6.3291	7.3217	4.6159	5.8865	3.9804	3.7454	3.9899
SCA	9.4641	8.2399	9.4312	8.9309	9.298	9.517	9.2506
FA	10.005	9.824	7.3172	7.3571	7.1632	8.7833	8.1341
GOA	8.9501	8.3386	6.9473	6.7838	5.6549	6.3476	6.0404
TLBO	3.8652	7.3906	6.2127	4.2202	5.5663	5.696	6.0148
CMA-ES	4.4836	10.278	7.3832	6.1895	5.7586	7.7156	7.4959
MFO	6.9976	8.0348	7.055	7.1332	7.0379	7.6824	7.3757
AOA	6.3965	6.5091	8.3732	9.1576	7.9849	9.2846	8.2072
IWO	7.7272	9.9419	8.303	7.7436	6.9385	7.6078	6.2446

A post-hoc Nemenyi test was utilized to delve deeper into the distinctions among the algorithms. If the null hypothesis is rejected, a post-hoc test can be conducted. The Nemenyi test is employed when conducting pairwise comparisons among all algorithms. The performance disparity between two classifiers is deemed significant if their respective average ranks exhibit a difference equal to or exceeding the $CD$ (Eq. 20)^141^.

20 $$CD={q}_{\alpha }\sqrt{\frac{k(k+1)}{6N}}$$

**Figure 11:**
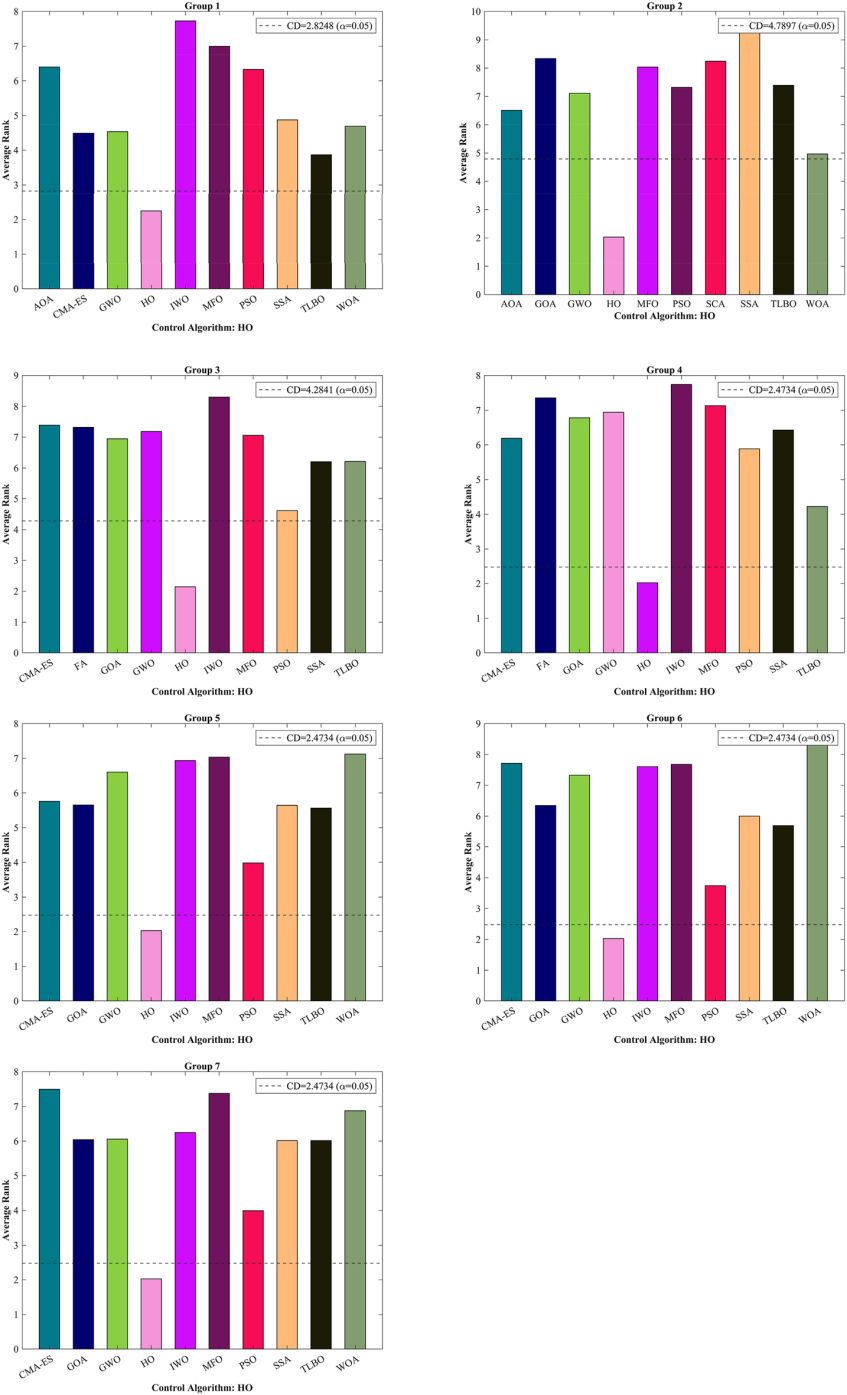
Nemenyi test for top ten algorithms in each group with $\alpha$= 0.05.

$N$ represents the number of BFs in each group, $k$ represents the number of algorithms under comparison and in each group, we selected the top 10 algorithms for comparison. At a significance level of $\alpha = 0.05$, the critical value for 10 algorithms, the associated $CD$ for each group has been specified in Fig. 11. To identify distinctions among the ten algorithms, the $CD$ derived from the Nemenyi test was employed. The $CD$ diagrams depicted in Fig. 11 offer straightforward and intuitive visualizations of the outcomes from a Nemenyi post-hoc test. This test is specifically designed to assess the statistical significance of differences in average ranks among a collection of ten algorithms, each evaluated on a set of seven groups.

Following the revelation of notable variations in performance among various algorithms, it becomes imperative to identify which algorithms exhibit significantly different performances compared to HO. HO is regarded as control algorithm in this context. Figure 11 displays the average ranking of each method across seven groups, with significance levels of 0.05 in 30 distinct runs. HO demonstrates significant superiority over algorithms whose average ranking exceeds the threshold line indicated in the figure. In group 1, HO held the first rank in all groups and exhibited significant superiority over TLBO, CMA-ES, GWO, WOA respectively. Moving to group 2, WOA secured the second position after HO and could significantly outperform AOA, GWO, and PSO while in group 3, PSO attained the second position following HO and TLBO, SSA, and GOA are ranked 3, 4, and 5, respectively. In group 4, TLBO outperforms algorithm PSO, and consequently, we observe the placement of algorithms, HO, TLBO, PSO, CMA-ES, SSA but within group 5, the PSO algorithm performs better than the TLBO algorithm. As a result, the arrangement or ranking of algorithms within this group is as follows: HO, PSO, TLBO, GOA, CMA-ES. Continuing, in group 6, it is observable that HO outperforms the other algorithms, and furthermore, the sequence of algorithms is as follows: PSO, TLBO, SSA, GOA, GWO. Lastly in group, the line-up of algorithms is as follows: HO, TLBO, PSO, CMA-ES, SSA.

A post-hoc analysis determines that if the disparity in mean Friedman values between the two algorithms falls below the $CD$ threshold, there is no notable distinction between them; conversely, if it surpasses the $CD$ value, a significant difference between the algorithms exists. In Table 9, a comparison has been conducted between 12 algorithms and HO across all seven BF groups. Algorithms that are not significantly different from the HO algorithm are highlighted with a red mark. Conversely, algorithms that are deemed significantly different from the HO algorithm are highlighted with a green mark in this table. In accordance with Table 9, none of the examined algorithms in this article can serve as a substitute for algorithm HO. This observation underscores the necessity of the existence of algorithm HO, which can potentially address limitations not covered by other algorithms.

**Table 9:** Nemenyi’s statistical test for seven groups of BFs (Control Algorithm: HO).

Algorithms	Functions						
	**F1-F23**	**ZP**	**CEC 2019**	**CEC 2014 (10)**	**CEC 2014 (30)**	**CEC 2014 (50)**	**CEC 2014 (100)**
HO vs. WOA	❌	❌	✅	✅	✅	✅	✅
HO vs. GWO	❌	✅	✅	✅	✅	✅	✅
HO vs. SSA	❌	✅	❌	✅	✅	✅	✅
HO vs. PSO	✅	✅	❌	✅	❌	❌	❌
HO vs. SCA	✅	✅	✅	✅	✅	✅	✅
HO vs. FA	✅	✅	✅	✅	✅	✅	✅
HO vs. GOA	✅	✅	✅	✅	✅	✅	✅
HO vs. TLBO	❌	✅	❌	❌	✅	✅	✅
HO vs. CMA-ES	❌	✅	✅	✅	✅	✅	✅
HO vs. MFO	✅	✅	✅	✅	✅	✅	✅
HO vs. AOA	✅	❌	✅	✅	✅	✅	✅
HO vs. IWO	✅	✅	✅	✅	✅	✅	✅

#### Sensitivity analysis

HO is a swarm-based optimizer that conducts the optimization procedure through iterative calculations. Hence, it is anticipated that the hyperparameters $\mathcal{N}$ (representing the population size) and $\mathcal{T}$ (indicating the total number of algorithm iterations) will influence the optimization performance of HO. Consequently, the sensitivity analysis of HO to hyperparameters $\mathcal{N}$ and $\mathcal{T}$ is provided in this subsection. To analyze the sensitivity of HO to hyperparameter $\mathcal{N}$, the proposed algorithm is employed for different values of $\mathcal{N}$, specifically 20, 30, 50, and 100. This variation in $\mathcal{N}$ is utilized to optimize functions from F1 to F23 BFs.

**Figure 12:**
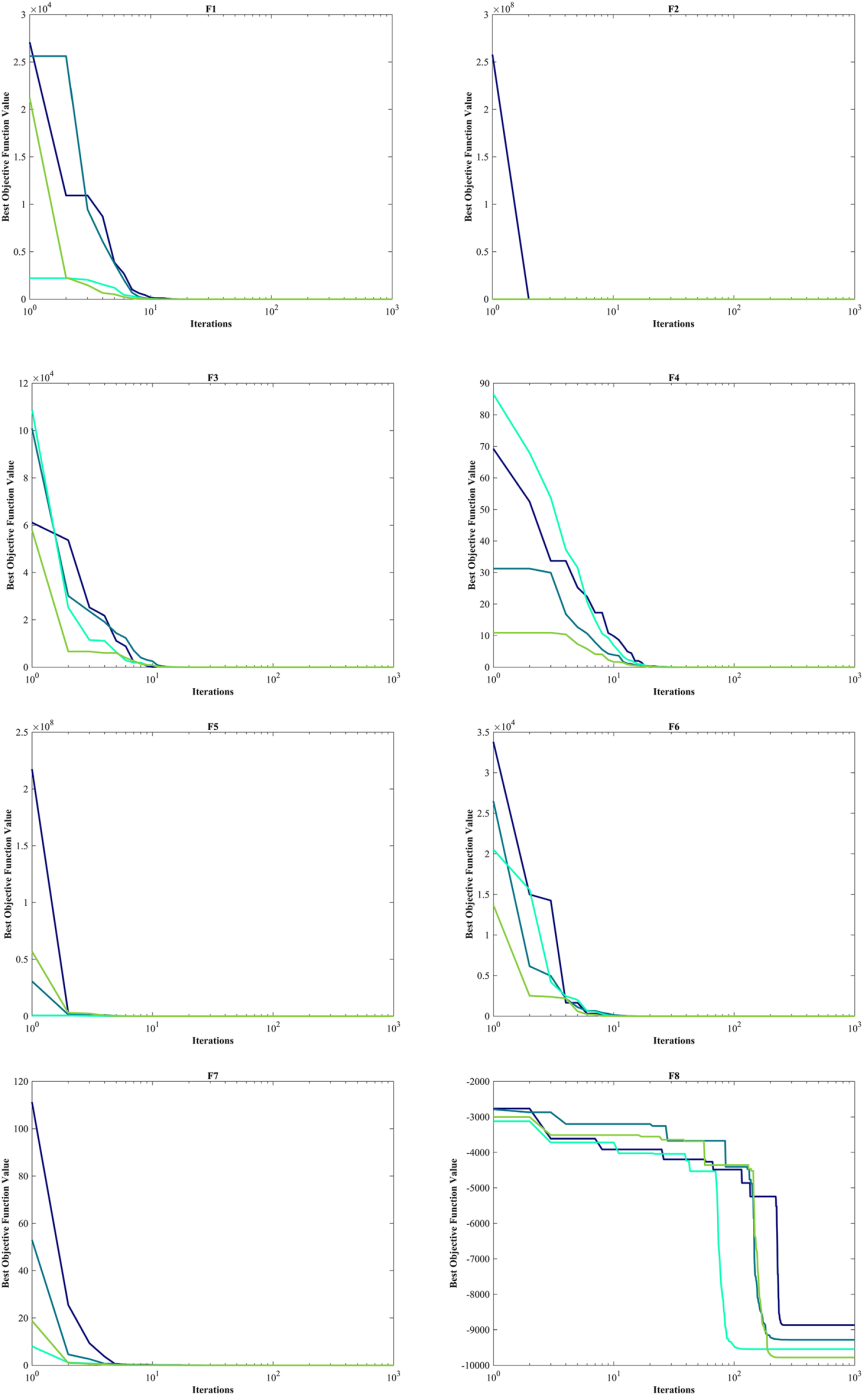

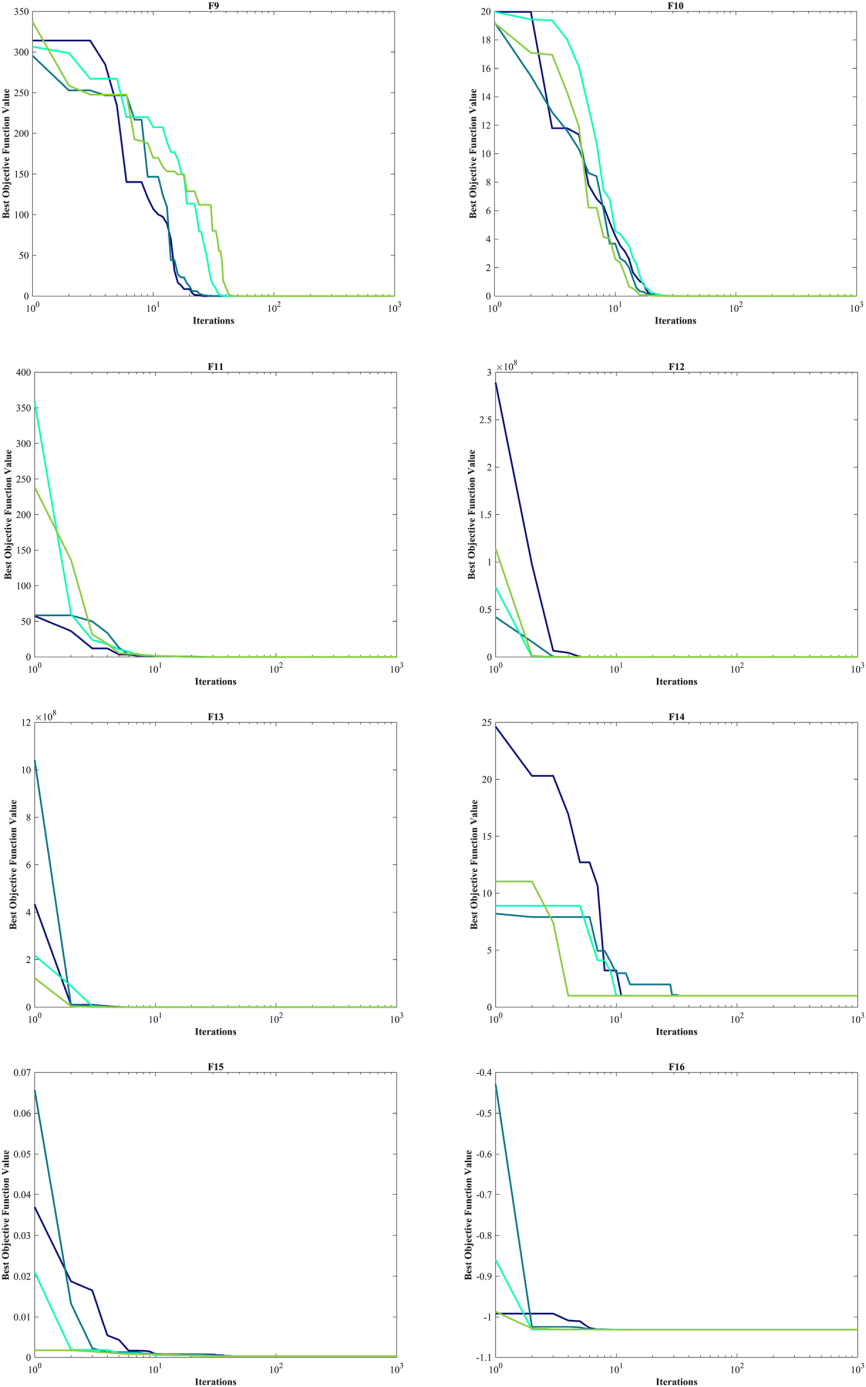

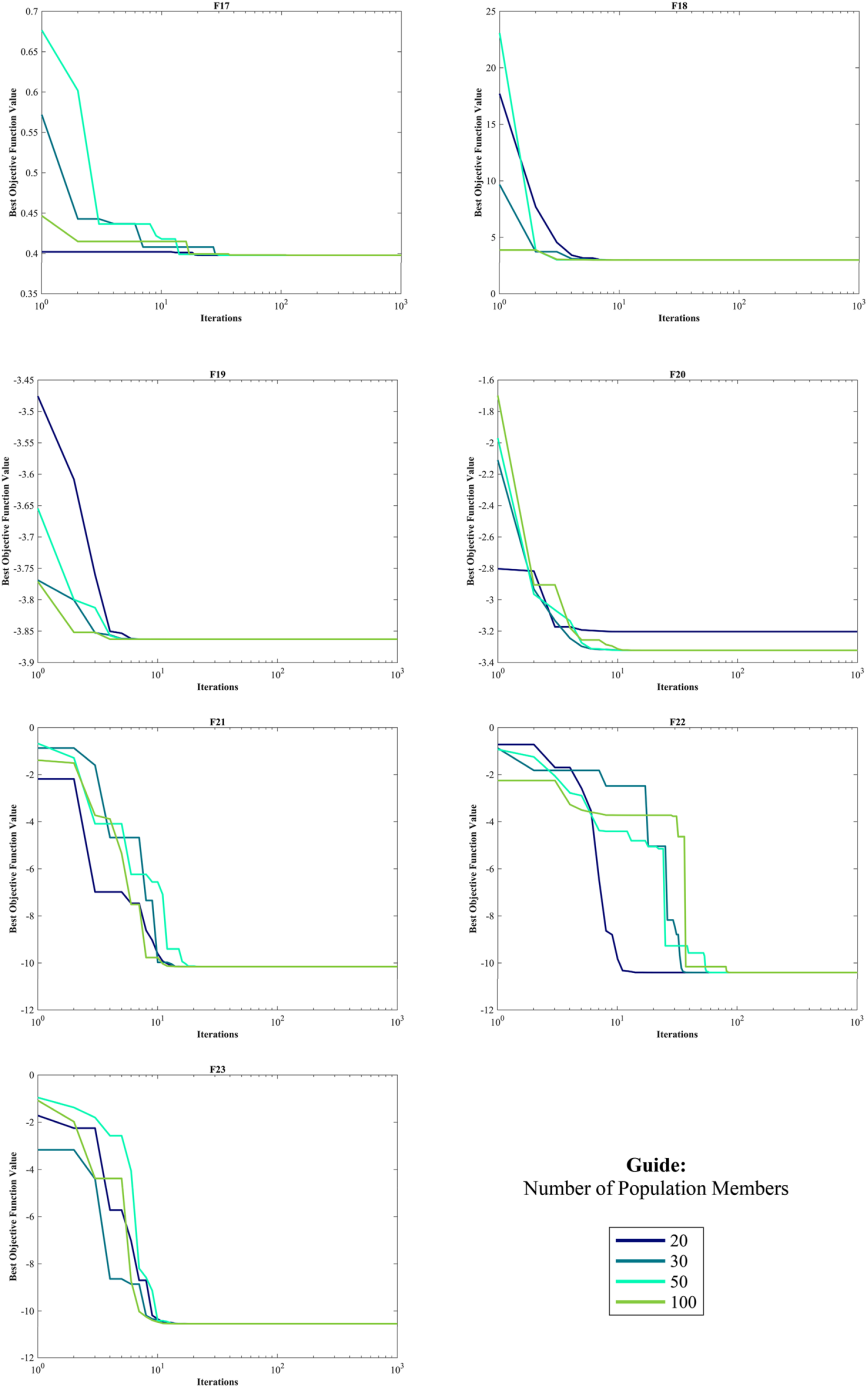
The convergence curves of HO during the investigation of sensitivity analysis regarding parameter $\mathcal{N}$.

The optimization results are provided in Table 10, and the convergence curves of HO under this analysis are depicted in Fig. 12. What is evident from the analysis of HO’s sensitivity to the hyperparameter $\mathcal{N}$ is that increasing the searcher agents improves HO’s search capability in scanning the search space, which enhances the performance of the proposed algorithm and reduces the values of the objective function.

**Table 10:** Findings from the sensitivity analysis of HO concerning parameter $\mathcal{N}$.

Objective Functions	Number of population members			
	20	30	50	100
F1	0	0	0	0
F2	0	0	0	0
F3	0	0	0	0
F4	0	0	0	0
F5	15.4682	2.0125	7.0959E-06	1.4080E-06
F6	0	0	0	0
F7	4.3541E-05	3.8741E-05	2.5178E-05	2.1020E-05
F8	− 9265.3130	− 9282.0349	− 9543.2299	− 9778.5922
F9	0	0	0	0
F10	4.4409E-16	4.4409E-16	4.4409E-16	4.4409E-16
F11	0	0	0	0
F12	3.1556E-28	1.5705E-32	1.5705E-32	1.5705E-32
F13	1.5802E-24	1.3498E-32	1.3498E-32	1.3498E-32
F14	0.9980	0.9980	0.9980	0.9980
F15	3.0749E-04	3.0749E-04	3.0749E-04	3.0749E-04
F16	− 1.0316	− 1.0316	− 1.0316	− 1.0316
F17	0.3979	0.3979	0.3979	0.3979
F18	3	3	3	3
F19	− 3.8628	− 3.8628	− 3.8628	− 3.8628
F20	− 3.3210	− 3.3220	− 3.3220	− 3.3220
F21	− 10.1532	− 10.1532	− 10.1532	− 10.1532
F22	− 10.4029	− 10.4029	− 10.4029	− 10.4029
F23	− 10.5364	− 10.5364	− 10.5364	− 10.5364

**Figure 13:**
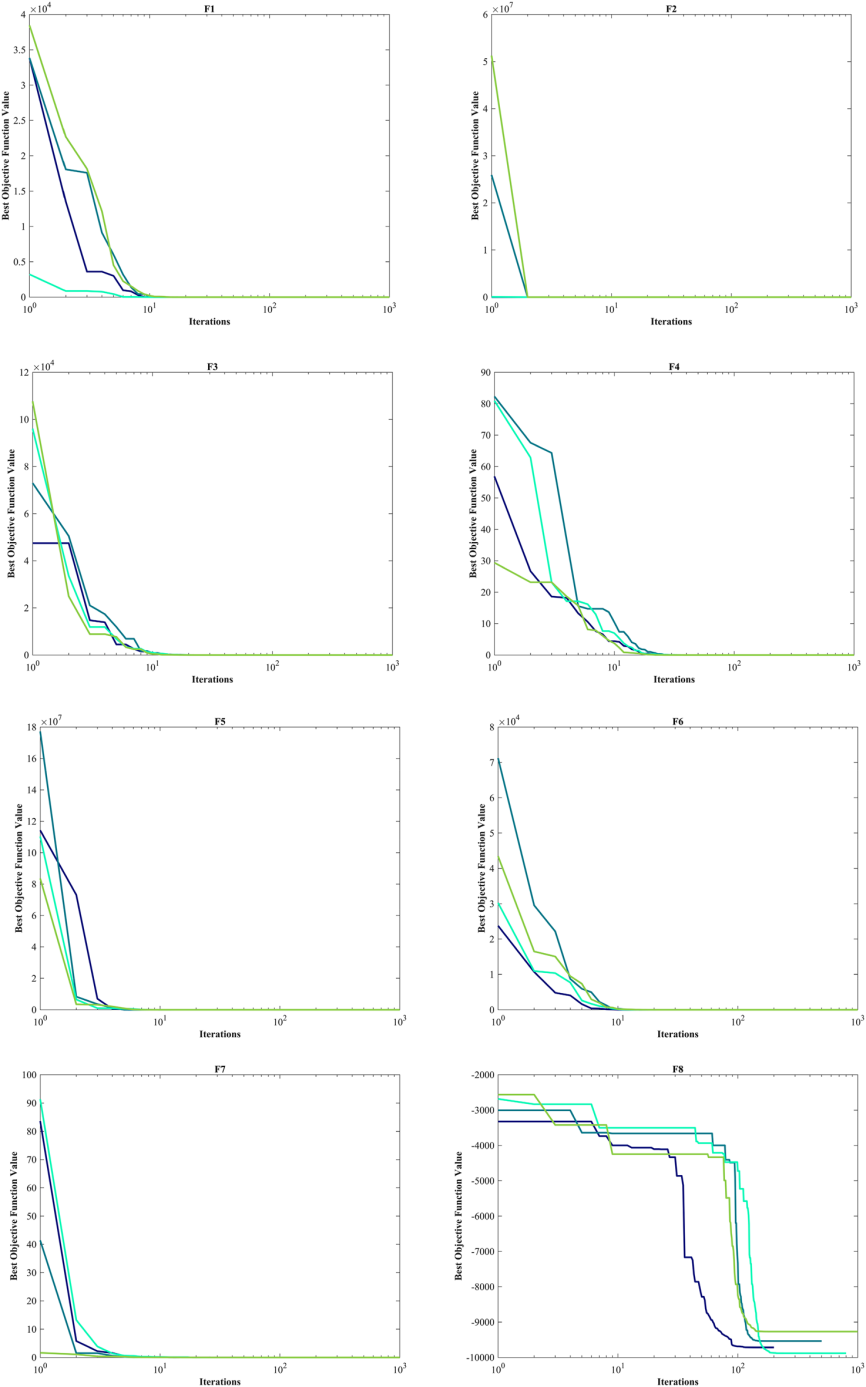

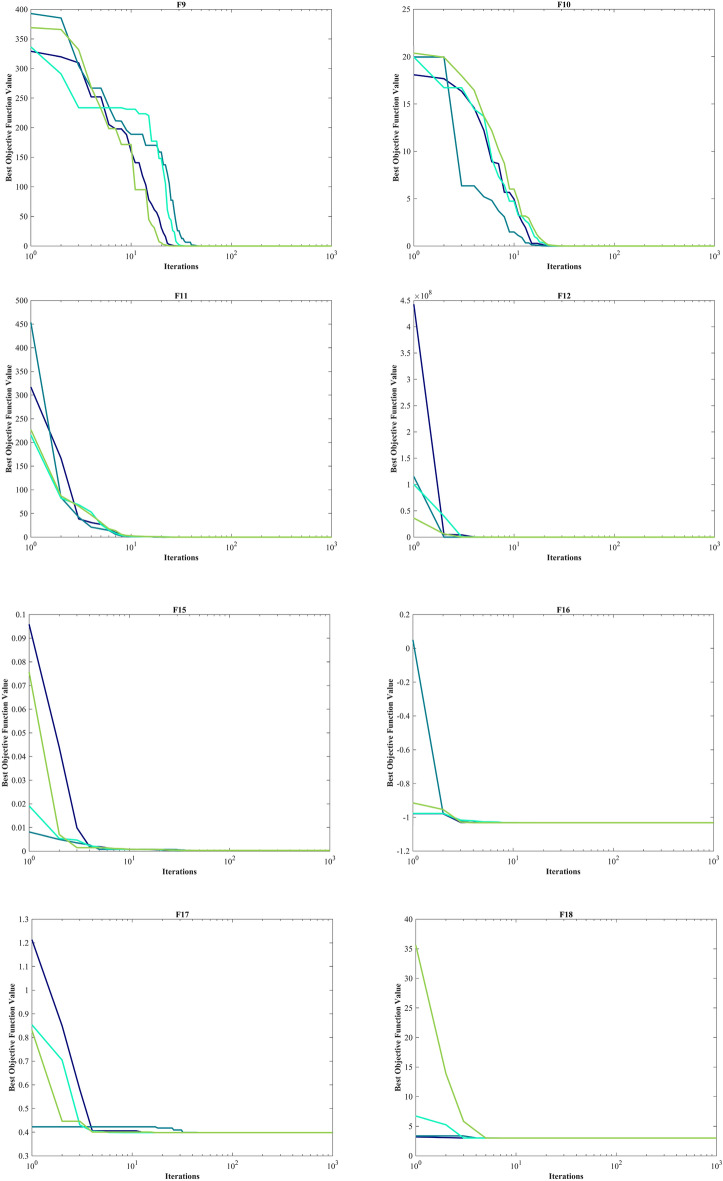

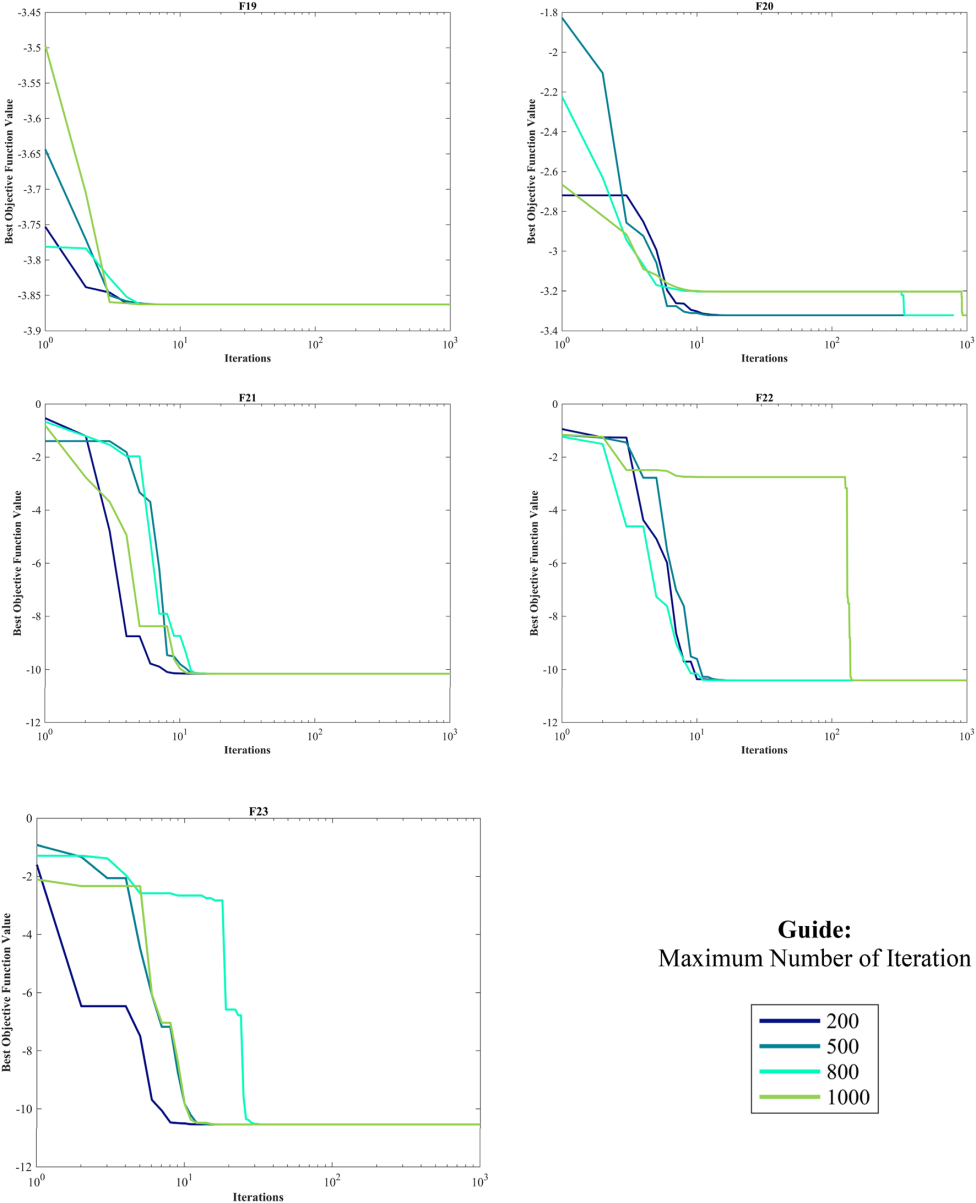
The convergence curves of HO during the investigation of sensitivity analysis regarding parameter $\mathcal{T}$.

To analyze the sensitivity of the proposed algorithm to hyperparameter $\mathcal{T}$, HO is utilized for different values of $\mathcal{T}$, specifically 200, 500, 800, and 1000. These variations in $\mathcal{T}$ are employed to optimize functions from F1 to F23 BFs. The optimization results are provided in Table 11, and the convergence curves of HO under this analysis are depicted in Fig. 13. According results, it is observed that higher values of $\mathcal{T}$ provide the algorithm with increased opportunities to converge to superior solutions, primarily due to enhanced exploitation ability. Hence, it is evident that as the values of $\mathcal{T}$ increase, the optimization process becomes more efficient, leading to decreased values of the objective function.

**Table 11:** Findings from the sensitivity analysis of HO concerning parameter $\mathcal{T}$.

Objective Functions	Maximum number of iteration			
	200	500	800	1000
F1	0	0	0	0
F2	0	0	0	0
F3	1.5456E-41	0	0	0
F4	1.6531E-66	0	0	0
F5	20.9691	13.3132	5.4924	3.3495
F6	0	0	0	0
F7	2.6782E-04	7.1535E-05	7.0243E-05	1.5269E-05
F8	− 9.7163E + 03	− 9.5356E + 03	− 9.8802E + 03	− 9.2684E + 03
F9	0	0	0	0
F10	4.4409E− 16	4.4409E-16	4.4409E-16	4.4409E-16
F11	0	0	0	0
F12	1.2730E-10	3.3597E-24	1.5705E-32	1.5705E-32
F13	3.5137E-08	7.2720E-21	1.3498E-32	1.3498E-32
F14	0.9980	0.9980	0.9980	0.9980
F15	3.0749E-04	3.0749E-04	3.0749E-04	3.0749E-04
F16	− 1.0316	− 1.0316	− 1.0316	− 1.0316
F17	0.3979	0.3979	0.3979	0.3979
F18	3	3	3	3
F19	− 3.8628	− 3.8628	− 3.8628	− 3.8628
F20	− 3.3220	− 3.3220	− 3.3220	− 3.3220
F21	− 10.1532	− 10.1532	− 10.1532	− 10.1532
F22	− 10.4029	− 10.4029	− 10.4029	− 10.4029
F23	− 10.5364	− 10.5364	− 10.5364	− 10.5364

According to Tables 10 which iteration hyperparameter is kept constant and Table 11 which population parameter is held constant, the performance of the HO algorithm improves with an increase in population and iteration, except for F8 as shown in Table 11. Based on the results, it is observed that the algorithm is less sensitive to changes in the iteration hyperparameter (Table 12).

**Table 12:** The attributes of the wind turbine.

Parameter	Value
Thrust coefficient (${C}_{T}$)	0.88
Ground surface roughness (${h}_{0}$)	0.3
Hub height ($h$)	60 m
Rotor diameter (${D}_{0}$)	40 m
Turbine efficiency ( $\eta$)	40%
Air density (${\rho }_{air}$)	1.225 kg/m3
Wind speed (${V}_{0}$)	12 m/s
Wind directions (degree)	{10°, 20°, 30°,…,360°} wind direction angle w.r.t. + x (East)

### Hippopotamus optimization algorithm for engineering problems

In this section, the effectiveness of the HO is evaluated in relation to its ability to address practical optimization problems in four of problem distinct engineering design challenges. The HO is employed to solve these problems, utilizing a total of 30,000 evaluations. The statistical outcomes obtained using various methodologies are showcased in Table 13. Additionally, Fig. 18 illustrates the boxplots of the algorithms.

**Table 13:** Evaluation results of the engineering problems.

F	M	Optimization Algorithms												
		HO	WOA	GWO	SSA	PSO	SCA	FA	GOA	TLBO	CMA-ES	MFO	AOA	IWO
TCS	Mean	0.012665	0.013544	0.012769	0.013057	0.013759	0.013033	0.01277	0.62228	0.012677	0.013488	0.013466	0.017608	0.012829
	Best	0.012665	0.012675	0.012672	0.012669	0.012667	0.012728	0.012666	0.013193	0.012665	0.012665	0.012667	0.013195	0.012713
	Worst	0.012665	0.016002	0.013689	0.015436	0.016642	0.013222	0.01311	2.7935	0.012703	0.017773	0.017773	0.034425	0.014
	Std	2.95E-09	0.0010105	0.00019007	0.00065595	0.0011223	0.00014162	0.0001116	1.0199	9.61E-06	0.0012441	0.001263	0.0080586	0.0002798
	Median	0.012665	0.013124	0.012722	0.012824	0.013348	0.013033	0.012719	0.050421	0.012676	0.012973	0.013193	0.013261	0.012747
	Rank	1	10	3	7	11	6	4	13	2	9	8	12	5
PV	Mean	6102.7	7217.2	6976	6304.6	6327.7	6718.2	6294.4	7817.5	12,292	6534.1	6587.4	6471.7	8758.1
	Best	6059.7	6149.7	6069.6	6059.7	6059.7	6173.7	6059.7	6059.7	6069.6	6059.7	6059.7	6059.7	6540.5
	Worst	7306.6	7857.6	10,069	6820.4	7047.3	7690.7	7306.5	17,459	34,344	7368.1	7682.9	7306.9	11,672
	Std	227.45	592.21	915.11	244.96	262.97	476.06	386.97	2691.5	6840.1	524.75	592.82	436.61	1704.9
	Median	6059.7	7596.2	7307.6	6237.2	6315.8	6503.8	6090.5	6523.2	10,020	6315.9	6317.1	6363	8520.7
	Rank	1	10	9	3	4	8	2	11	13	6	7	5	12
WB	Mean	1.7249	2.7799	1.7273	1.8772	1.7249	1.87	1.7249	3.9995	1.7249	1.7934	1.7768	2.4201	1.9001
	Best	1.7249	1.821	1.7254	1.7296	1.7249	1.7794	1.7249	1.9706	1.7249	1.7249	1.7249	2.0184	1.7268
	Worst	1.7249	6.0369	1.7359	2.1965	1.7249	1.9655	1.7249	5.9584	1.7249	2.3189	2.3788	2.7457	2.2028
	Std	1.16E-15	1.1889	0.0022462	0.14429	4.57E-09	0.036549	8.13E-08	1.0734	9.12E-16	0.13205	0.15727	0.14915	0.2014
	Median	1.7249	2.1722	1.7266	1.8146	1.7249	1.8729	1.7249	3.8978	1.7249	1.732	1.7252	2.4641	1.7552
	Rank	1	9	2	6	1	5	1	10	1	4	3	8	7
WFLO	Mean	0.0014571	0.0014576	0.0014672	0.0014678	0.0014621	0.0014644	0.0014581	0.0014662	0.0014735	0.0014689	0.0014752	0.0014635	0.0014638
	Best	0.0014557	0.0014565	0.0014638	0.0014653	0.0014595	0.0014608	0.0014567	0.0014622	0.0014669	0.0014612	0.0014683	0.0014583	0.0014581
	Worst	0.0014596	0.0014593	0.0014692	0.0014702	0.0014662	0.0014671	0.0014616	0.0014686	0.001478	0.0014793	0.0014807	0.0014697	0.0014705
	Std	6.39E-07	1.44E-06	1.19E-06	1.08E-06	1.80E-06	1.64E-06	1.10E-06	1.94E-06	2.64E-06	4.78E-06	3.82E-06	2.58E-06	3.40E-06
	Median	0.0014563	0.0014575	0.0014673	0.0014683	0.0014621	0.0014647	0.0014579	0.0014671	0.0014738	0.0014689	0.0014758	0.0014633	0.0014638
	Rank	1	2	9	10	4	7	3	8	12	11	13	5	6

#### TCS design

**Figure 14:**
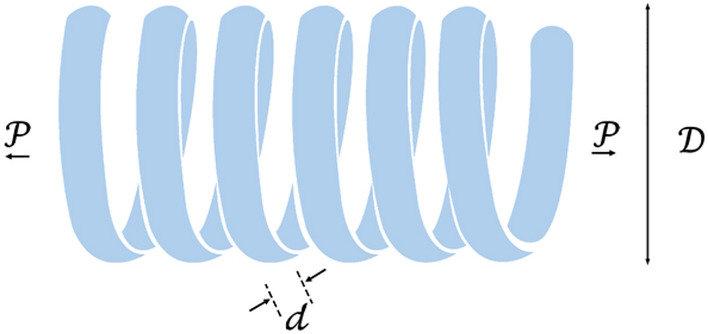
TCS.

This problem's primary aim entails minimizing the mass associated with the spring, as illustrated in Fig. 14, considering whether it is stretched or compressed. In order to achieve optimal design, it is important to ensure wave frequency, deflection limits, and stress are met. The mathematical representation of this engineering design can be described by the equation in Supplementary^142^. Based on the obtained outcomes, the HO has successfully obtained the optimal solution. Simultaneously, it ensures compliance with the specified constraints, as detailed in the references^45,102,142–145^. The optimal solutions achieved through the utilization of HO for this particular problem are {${\mathcalligra{z}}_{1}=0.051689714188651, {\mathcalligra{z}}_{2}= 0.356733450209264, {\mathcalligra{z}}_{3}= 11.288045038991518$}.

#### WB design

**Figure 15:**
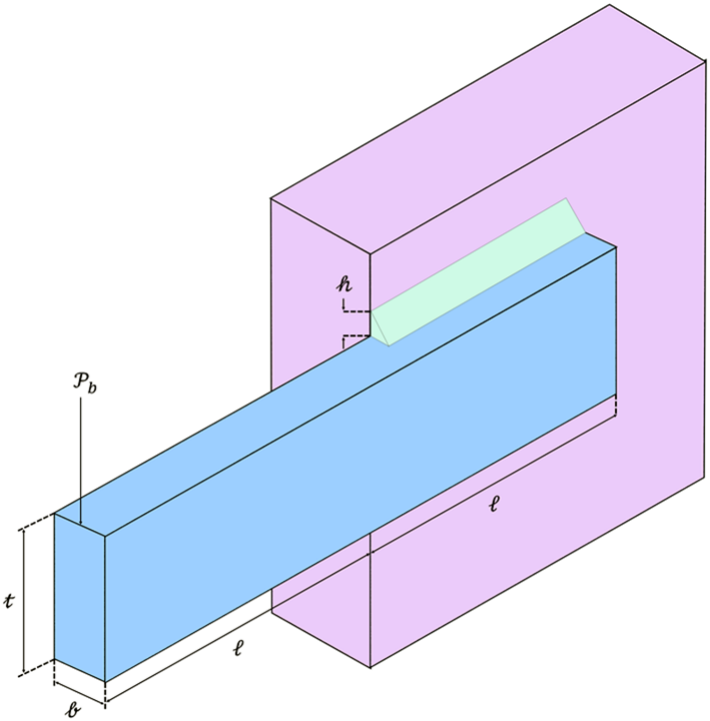
WB.

The objective is to minimize the cost associated with the welding beam. This objective is achieved by simultaneously addressing seven constraints. The problem concerning the design of a welded beam is visually depicted in Fig. 15. The optimal design problem for the welded beam is formulated as described in Supplementary^49^. The HO has the capability to identify the most favourable value for the optimization variables. Statistical analysis determined that that the HO exhibits superior performance. The optimal solutions achieved through the utilization of HO for this particular problem are {${\mathcalligra{z}}_{1}=0.205729639786079, {\mathcalligra{z}}_{2}=3.470488665628001, {\mathcalligra{z}}_{3}=9.036623910357633, {\mathcalligra{z}}_{4}= 0.205729639786079$}.

#### PV design

**Figure 16:**
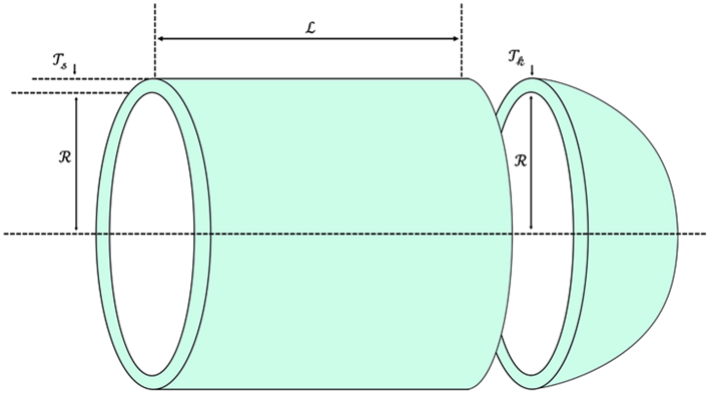
PV.

The primary objective revolves around minimizing the overall cost associated with the tank under pressurized conditions, considering factors such as forming techniques, welding methods, and material costs, as depicted in Fig. 16. The design process involves considering four variables and four constraints. The PV design problem is formulated as described in Supplementary^49^ .According to the reported results, the HO outperformed other methods. The optimal solutions achieved through the utilization of HO for this particular problem are {${\mathcalligra{z}}_{1}=13.4141563816526, {\mathcalligra{z}}_{2}=7.3495109848502, {\mathcalligra{z}}_{3}=42.0984455958549, {\mathcalligra{z}}_{4}=176.6365958424392$}. Further details regarding these constraints can be found in references^69^ and^145^.

#### WFLO

**Figure 17:**
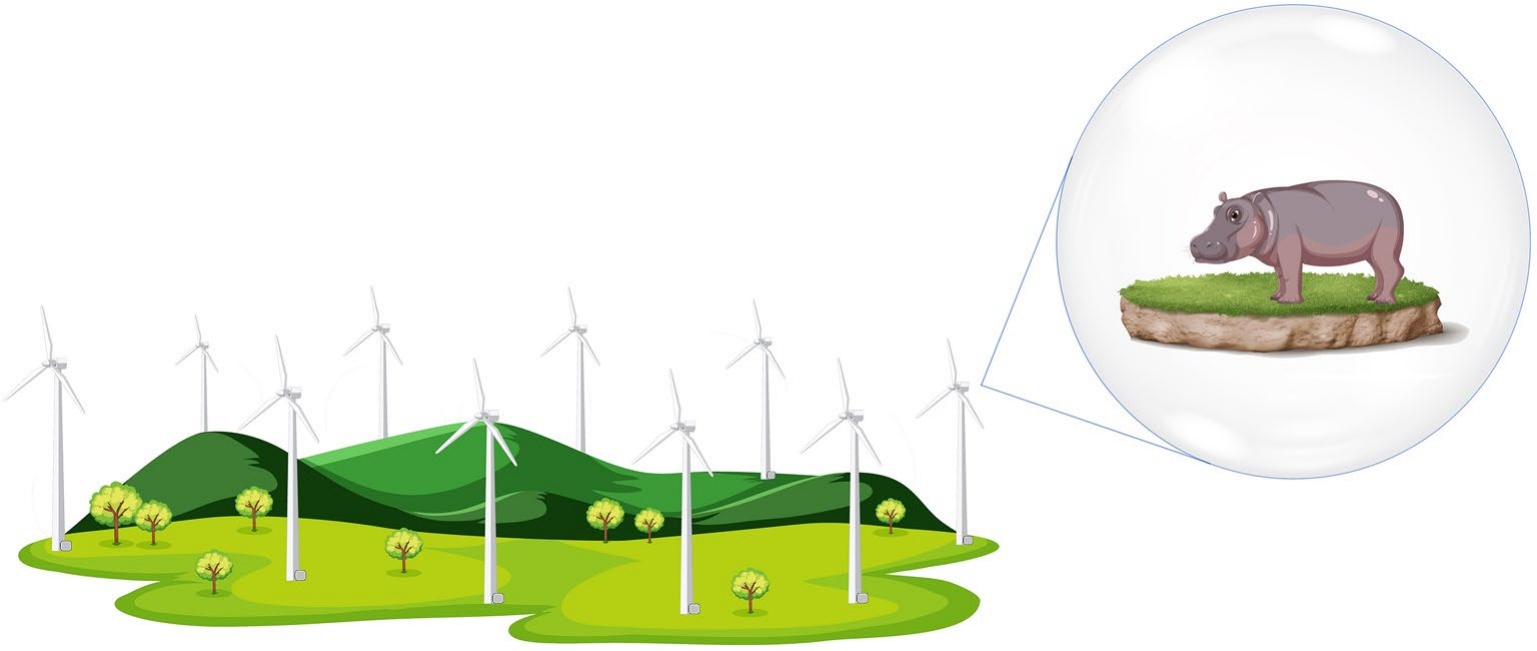
WFLO with HO.

We're figuring out where to place wind turbines on a 10 $\times$ 10 grid. We have 100 different options for where to put the turbines. We can have anywhere from 1 to 39 turbines in the wind farm. We're simulating wind coming from 36 different directions, all at a steady speed of 12 m per second. The objective is to minimize expenditures, maximize the aggregate power output, reduce acoustic emissions, and optimize various performance and cost-related metrics^13^ (Fig. 17). The attributes of the wind turbine are documented in Table 12. The formulation of WFLO problem is articulated as follows:

21 $$Minimizie:\mathcal{F}\left(\mathcalligra{z}\right)=\frac{\mathcal{C}\mathcalligra{o}\mathcalligra{s}\mathcalligra{t}}{{\mathcal{P}}_{\mathcalligra{t}\mathcalligra{o}\mathcalligra{t}\mathcalligra{a}\mathcalligra{l}}}$$

**Figure 18:**
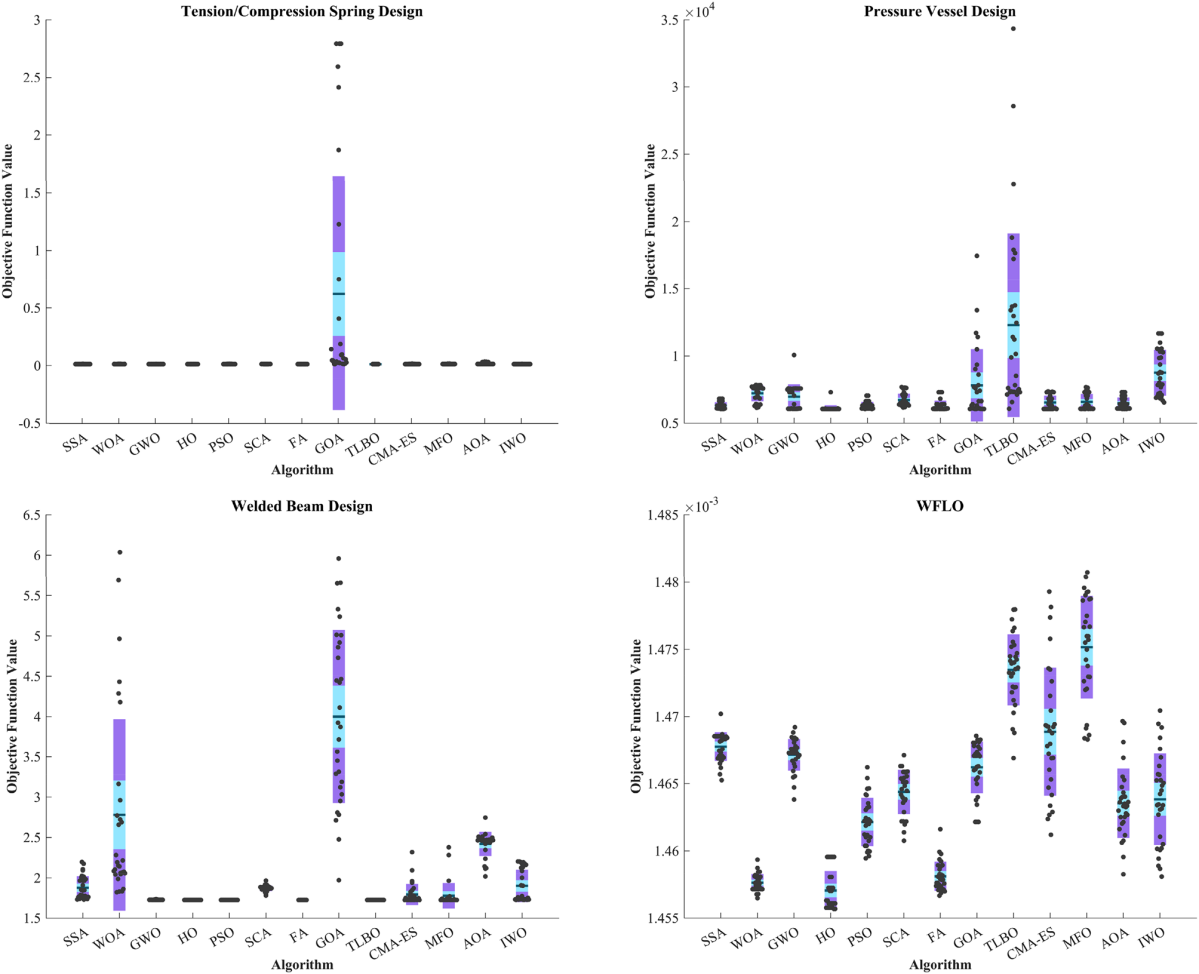
Boxplot illustrating the performance of the HO in comparison to twelve algorithms for optimizing TCS, WB, PV and WFLO.

Herein, $\mathcalligra{z}$ represents a vector comprising design variables, while ${\mathcal{P}}_{\mathcalligra{t}\mathcalligra{o}\mathcalligra{t}\mathcalligra{a}\mathcalligra{l}}$ denotes the aggregate power output generated by a wind farm. The computation of the $\mathcal{C}\mathcalligra{o}\mathcalligra{s}\mathcalligra{t}$ function can be derived according to the method described in^146^ (Fig. 18).

22 $$\mathcal{C}\mathcalligra{o}\mathcalligra{s}\mathcalligra{t}={\mathcal{N}}_{\mathcal{T}}(\frac{2}{3}+\frac{1}{3}{\mathcalligra{e}}^{-0.00174{\mathcal{N}}_{\mathcal{T}}^{2}})$$

The HO demonstrating superior performance compared to alternative approaches.

### Ethical approval

This article does not contain any studies with human participants or animals performed by any of the authors. Informed consent was not required as no human or animals were involved.

## Conclusions and future works

In this paper, we introduced a novel nonparametric optimization algorithm called the Hippopotamus Optimization (HO). The real inspiration behind the HO is to simulate the behaviors of hippopotamuses, incorporating their spatial positioning in the water, defense strategies against threats, and evasion techniques from predators. The algorithm is outlined conceptually through a trinary-phase model of their position update in river and pound, defense, and evading predators, each mathematically defined. In light of the results from addressing four distinct engineering design challenges, the HO has effectively achieved the most efficient resolution while concurrently upholding adherence to the designated constraints. The acquired outcomes from the HO were compared with the performance of 12 established metaheuristic algorithms. The algorithm achieved the highest ranking across 115 out of 161 BFs in finding optimal value. These benchmarks span various function types, including UM and HM functions, FM functions, in addition to the CEC 2019 test suite and CEC 2014 dimensions encompassing 10, 30, 50, and 100, along with the ZP.

The results of CEC 2014 test suite indicate that the HO swiftly identifies optimal solutions, avoiding entrapment in local minima. It consistently pursues highly optimal solutions at an impressive pace by employing efficient local search strategies. Furthermore, upon evaluation using the CEC 2019 test, it can be confidently asserted that the HO effectively finds the global optimal solution. Additionally, in the ZP, the HO demonstrates significantly superior performance compared to its competitors, achieving an optimal solution that remains unattainable for other investigated algorithms. Moreover, the observed lower Std. than that of the other investigated algorithms suggests that the HO displays resilience and efficacy in effectively addressing these functions.

Considering the outcomes derived from tackling four unique engineering design challenges, the HO has effectively demonstrated the most efficient resolution while maintaining strict adherence to the specified constraints. The application of the Wilcoxon signed test, Friedman and Nemenyi post-hoc test confirms that the HO displays a remarkable and statistically significant advantage over the algorithms under investigation in effectively addressing the optimization problems scrutinized in this study. The findings indicate that Ho exhibits lower sensitivity to changes in the iteration hyperparameter than the population hyperparameter.

The suggested methodology, HO, presents numerous avenues for future research exploration. Particularly, an area ripe with potential is the advancement of binary and multi-objective variants based on this proposed methodology. Furthermore, an avenue worth investigating in forthcoming research involves employing HO in optimizing diverse problem sets across multiple domains and real-world contexts.

### Supplementary Information

Supplementary Information.

## Data Availability

All data generated or analyzed during this study are included directly in the text of this submitted manuscript. There are no additional external files with datasets.

## Abbreviations

MaxIter: Max number of iterations
BF: Benchmark Function
UM: Unimodal
MM: Multimodal
FM: Fixed-dimension Multimodal
HM: High-dimensional Multimodal
ZP: Zigzag Pattern benchmark test
TCS: Tension/Compression Spring
WB: Welded Beam
PV: Pressure Vessel
WFLO: Wind Farm Layout Optimization
F: Function
CEC: IEEE Congress on Evolutionary Computation
D: Dimension
C19: CEC2019
C14: CEC2014
CMA-ES: Evolution Strategy with Covariance Matrix Adaptation
MFO: Moth-flame Optimization
AOA: Arithmetic Optimization Algorithm
TLBO: Teaching-Learning-Based Optimization
IWO: Invasive Weed Optimization
GOA: Grasshopper Optimization Algorithm
FA: Firefly Algorithm
PSO: Particle Swarm Optimization
SSA: Salp Swarm Algorithm
CD: Critical Difference
GWO: Gray Wolf Optimization
SCA: Sine Cosine Algorithm
WOA: Whale Optimization Algorithm
Best: The best result
Worst: The worst result
Std.: Standard Deviation
Mean: Average best result
