# Supplementary material for: Hippopotamus optimization algorithm: a novel nature-inspired optimization algorithm
Source: Sci Rep. 2024 Feb 29;14:5032. doi: 10.1038/s41598-024-54910-3 (PMC10904400; doi:10.1038/s41598-024-54910-3)
Supplement: Supplementary file 1 — Supplementary Information. [file 41598_2024_54910_MOESM1_ESM.docx]

In Fig. [S1](#f1) The defensive behavior of the hippo against the predator is show.

| **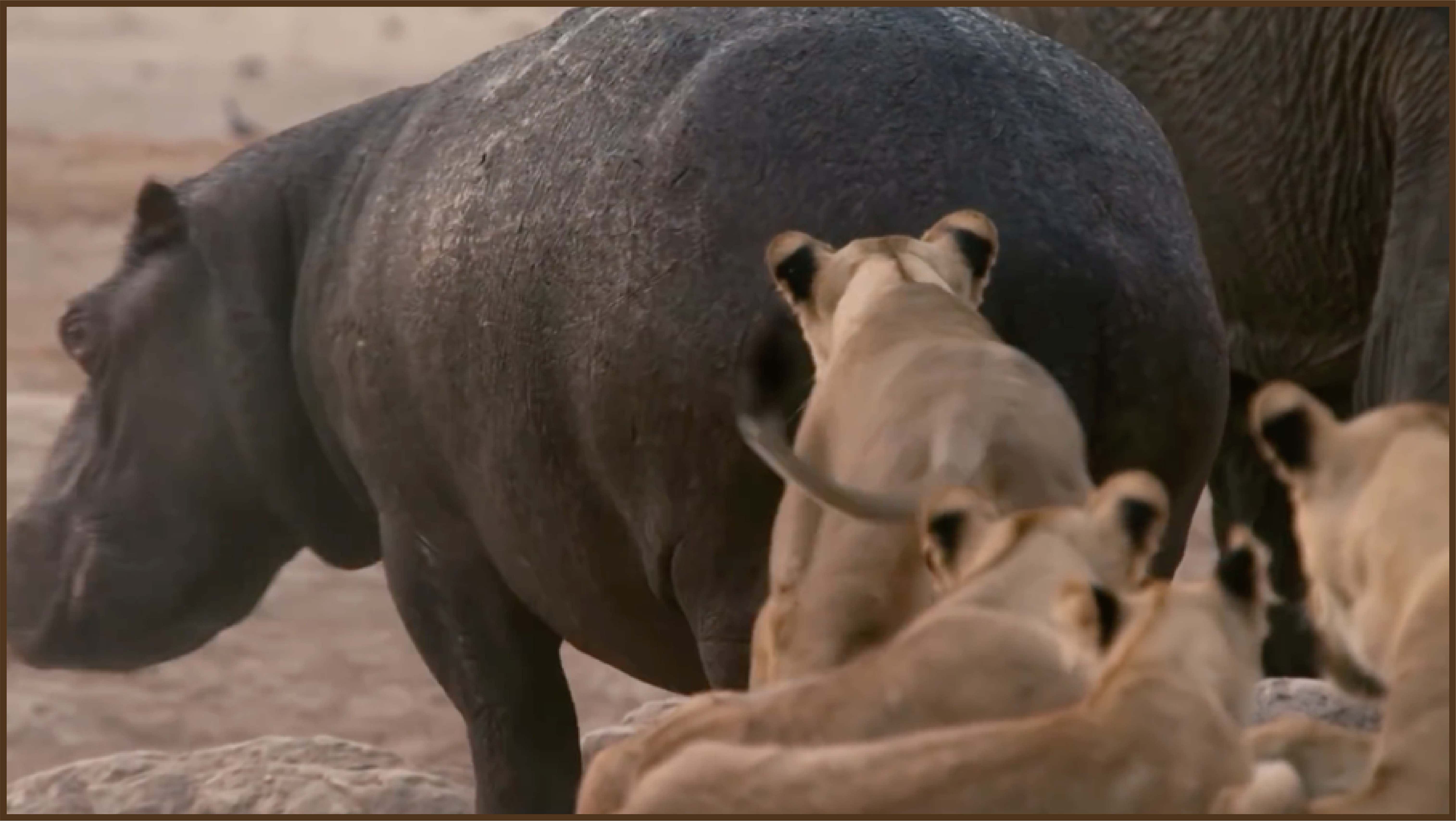**  **(b)** | **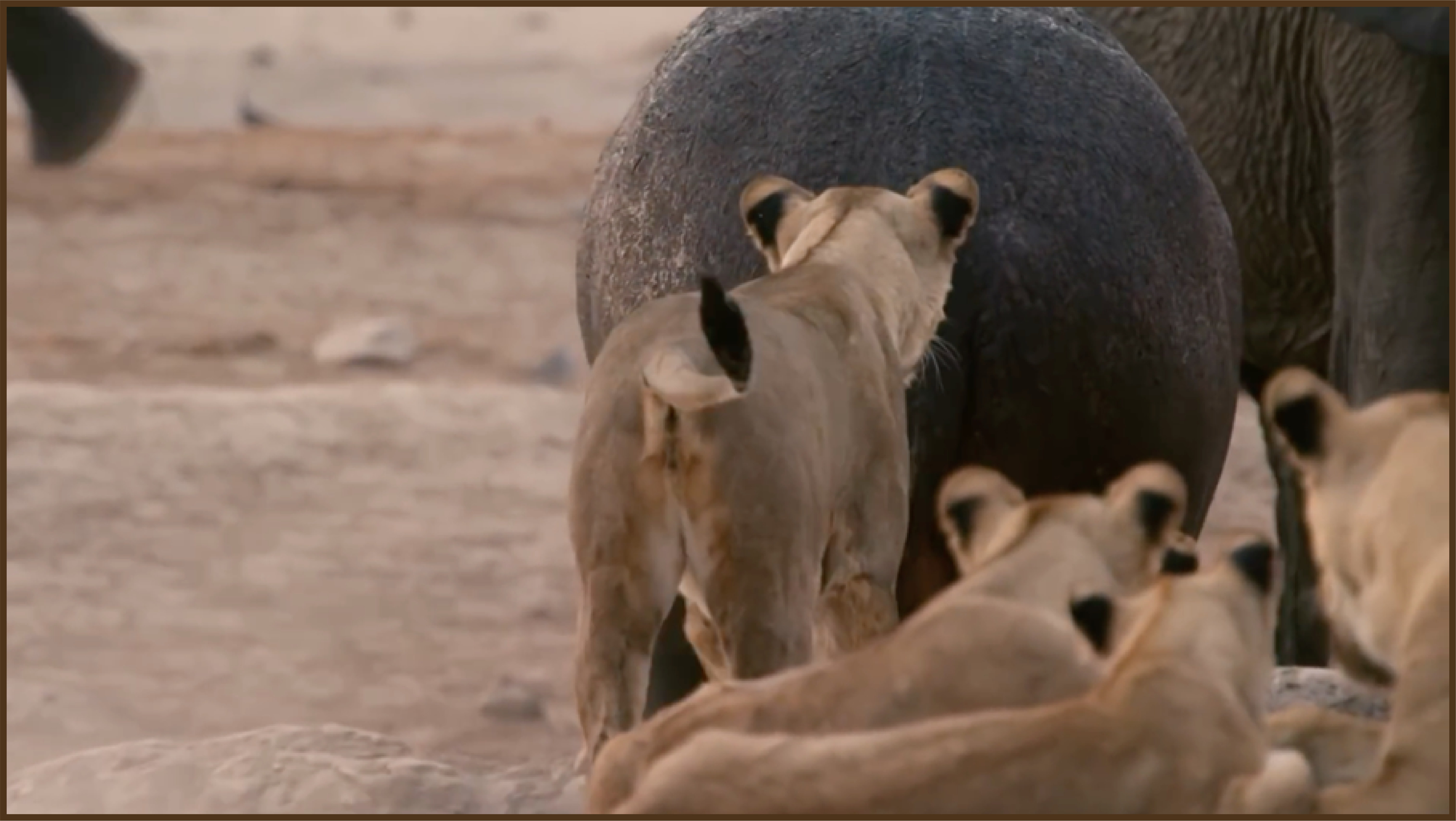**  **(a)** |
| --- | --- |
| **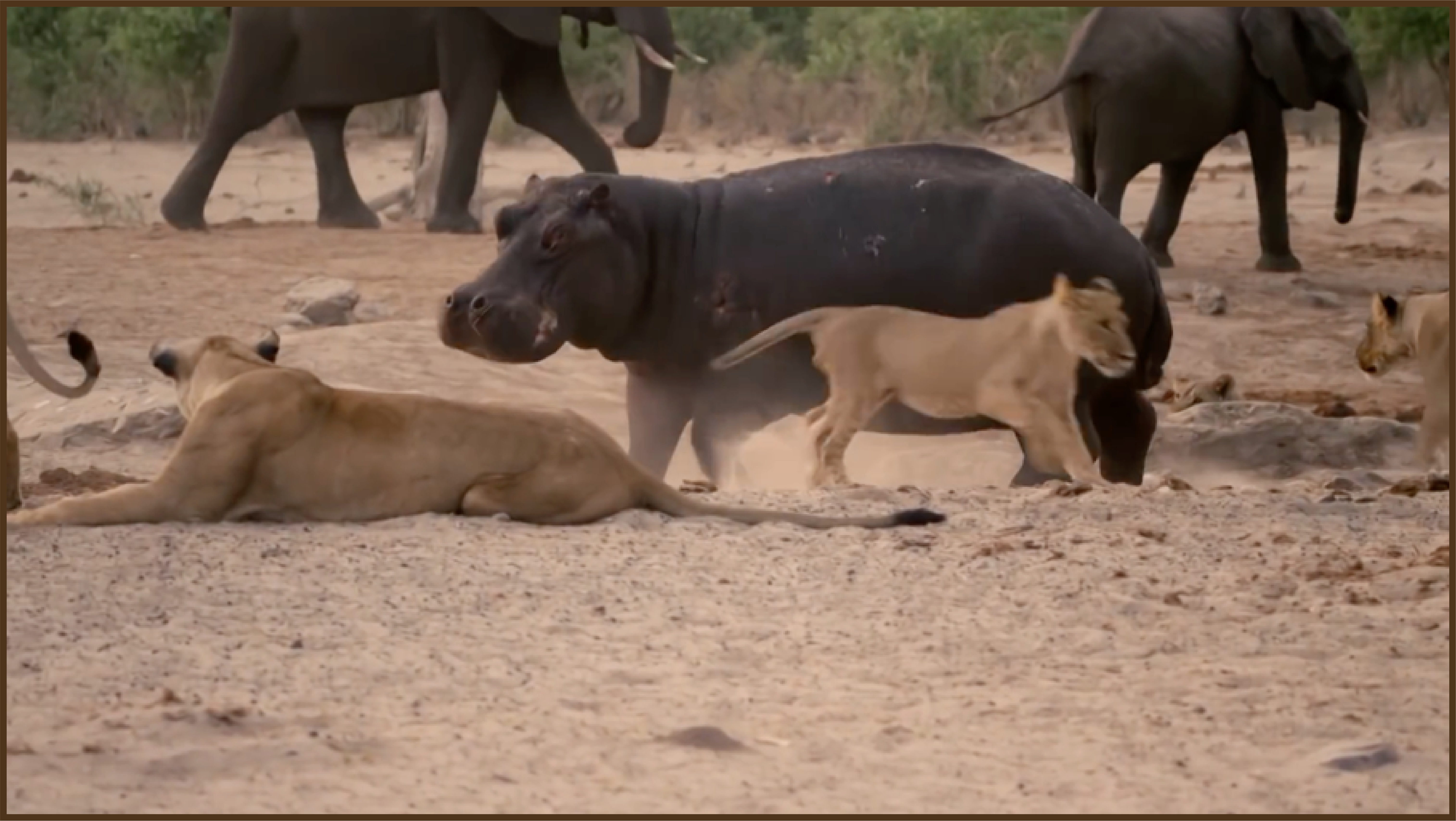**  **(d)** | **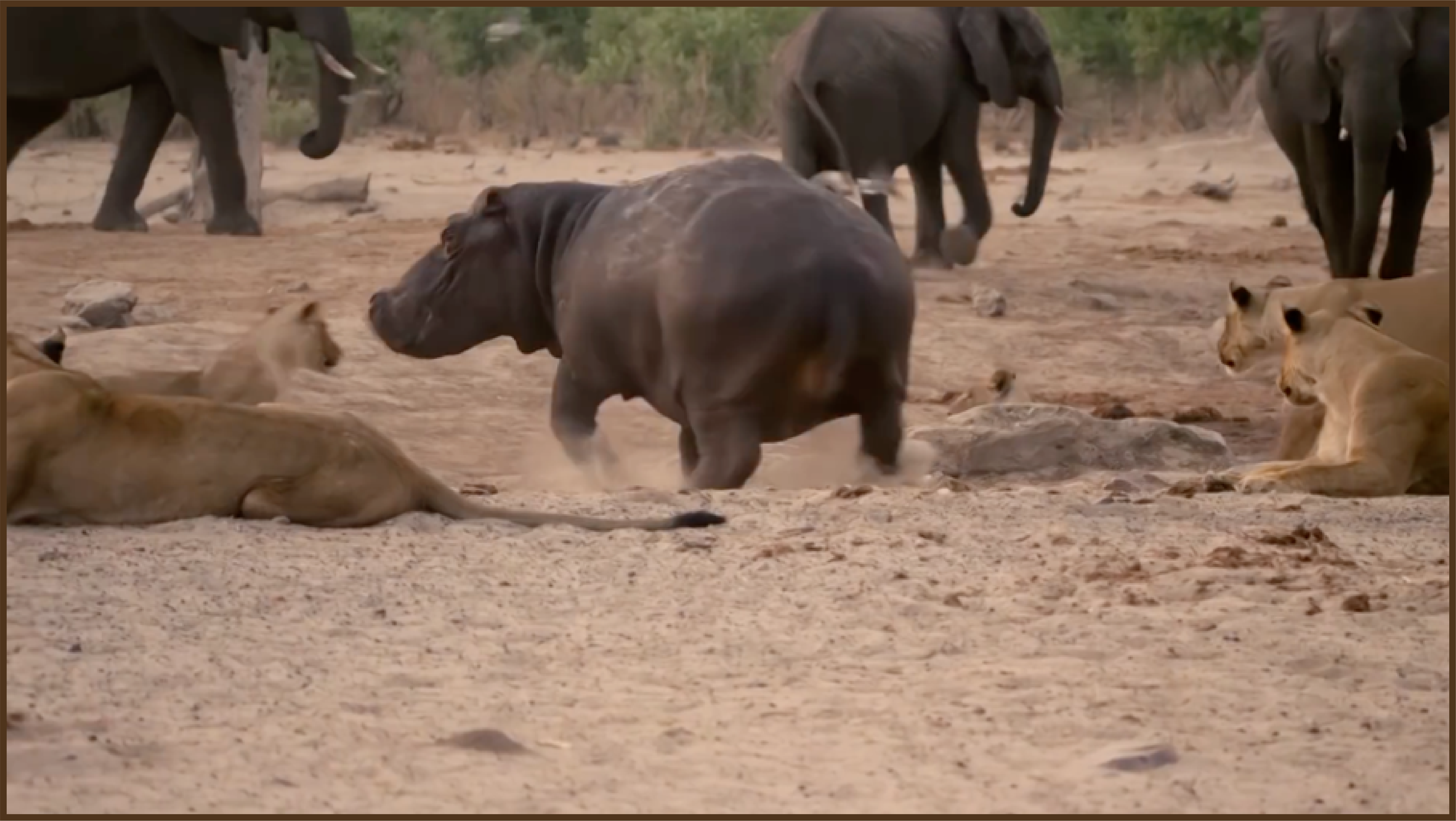**  **(c)** |

**Figure S1.** (a) - (d) shows the defensive behavior of the hippopotamus against the predator. (close-up)

**Evaluation of the CEC 2014 test suite**

This section reports the results of HO and twelve investigated algorithms for optimization problems (CEC 2014) of dimensions 10, 30, 50, and 100.

Evaluation outcomes for the objectives specified in the CEC 2014 (D = 10).

| **F** | **M** | **Optimization Algorithms** | | | | | | | | | | | | |
| --- | --- | --- | --- | --- | --- | --- | --- | --- | --- | --- | --- | --- | --- | --- |
|  |  | **HO** | **WOA** | **GWO** | **SSA** | **PSO** | **SCA** | **FA** | **GOA** | **TLBO** | **CMA-ES** | **MFO** | **AOA** | **IWO** |
| C14-F1 | Mean | 100 | 1.08E+07 | 1.26E+07 | 9.42E+05 | 30150 | 9.41E+06 | 9.54E+05 | 3.39E+05 | 26094 | 1.14E+05 | 1.75E+06 | 2.19E+07 | 2.22E+05 |
|  | Best | 100 | 9.28E+05 | 3.18E+06 | 52562 | 190.43 | 2.22E+06 | 25530 | 1809.7 | 1532.4 | 4277.7 | 1799.6 | 6.75E+06 | 6501.2 |
|  | Worst | 100 | 2.64E+07 | 4.10E+07 | 2.66E+06 | 1.08E+05 | 1.60E+07 | 8.82E+06 | 2.70E+06 | 89975 | 2.51E+05 | 7.31E+06 | 6.68E+07 | 1.08E+06 |
|  | Std. | 4.23E-07 | 7.65E+06 | 1.01E+07 | 7.24E+05 | 29545 | 3.55E+06 | 1.69E+06 | 7.48E+05 | 22756 | 75790 | 2.03E+06 | 1.40E+07 | 2.70E+05 |
|  | Median | 100 | 7.79E+06 | 9.01E+06 | 6.84E+05 | 22183 | 9.28E+06 | 3.67E+05 | 77750 | 20284 | 92467 | 1.09E+06 | 1.75E+07 | 1.08E+05 |
|  | Rank | 1 | 11 | 12 | 7 | 3 | 10 | 8 | 6 | 2 | 4 | 9 | 13 | 5 |
| C14-F2 | Mean | 200 | 4.59E+06 | 1.73E+08 | 3590.5 | 1748.6 | 8.46E+08 | 2499.4 | 4322.9 | 679.6 | 5819.7 | 7788.7 | 5.26E+09 | 3885.8 |
|  | Best | 200 | 7.35E+05 | 1416.3 | 208.22 | 201.56 | 2.52E+08 | 200.22 | 214.84 | 201.16 | 290.12 | 285.31 | 2.30E+09 | 209.85 |
|  | Worst | 200 | 4.53E+07 | 9.25E+08 | 10893 | 8624.9 | 1.76E+09 | 7798.2 | 12034 | 2755.1 | 11524 | 12034 | 7.59E+09 | 9699.6 |
|  | Std. | 1.38E-05 | 8.10E+06 | 2.17E+08 | 3075.7 | 1987.1 | 4.00E+08 | 2143.6 | 3990.7 | 612.71 | 3538.4 | 4267 | 1.30E+09 | 2997.4 |
|  | Median | 200 | 2.52E+06 | 6.60E+07 | 3018.2 | 910.15 | 7.22E+08 | 1896.5 | 2781.5 | 396.81 | 6103.4 | 9955.2 | 5.42E+09 | 3451.8 |
|  | Rank | 1 | 10 | 11 | 5 | 3 | 12 | 4 | 7 | 2 | 8 | 9 | 13 | 6 |

**Table S1.** Evaluation outcomes for the objectives specified in the CEC 2014 (D=10).

| C14-F3 | Mean | 300 | 28703 | 8160.4 | 8419.5 | 2220.8 | 6091.3 | 6968 | 9787.5 | 738.8 | 8145 | 21907 | 19714 | 28151 |
| --- | --- | --- | --- | --- | --- | --- | --- | --- | --- | --- | --- | --- | --- | --- |
|  | Best | 300 | 5218.3 | 1494 | 3630.4 | 304.03 | 1979.7 | 578.8 | 302.32 | 341.57 | 813.42 | 663.68 | 13347 | 11462 |
|  | Worst | 300 | 65692 | 17777 | 18109 | 13397 | 13046 | 15227 | 95286 | 2188 | 23449 | 83169 | 36193 | 57900 |
|  | Std. | 3.55E-10 | 16973 | 4221.4 | 3657.4 | 3121.6 | 3677 | 3543.6 | 17726 | 410.14 | 6569.9 | 19622 | 6034 | 11897 |
|  | Median | 300 | 23474 | 6755.2 | 7610.6 | 521.41 | 4748.3 | 7078.6 | 3951.3 | 612.53 | 7020.8 | 16258 | 17133 | 26171 |
|  | Rank | 1 | 13 | 7 | 8 | 3 | 4 | 5 | 9 | 2 | 6 | 11 | 10 | 12 |
| C14-F4 | Mean | 407.1 | 440.74 | 437.09 | 426.36 | 426.85 | 459.35 | 430.62 | 420.64 | 417.57 | 434.78 | 435.12 | 1734.5 | 425.82 |
|  | Best | 400 | 401.39 | 405.94 | 400.1 | 400.15 | 430.02 | 400 | 400 | 400 | 434.78 | 400.27 | 469.71 | 400.04 |
|  | Worst | 434.78 | 533.28 | 514.96 | 469.06 | 434.78 | 495.73 | 456.54 | 450.32 | 434.78 | 434.78 | 547.95 | 4914.6 | 434.78 |
|  | Std. | 12.739 | 27.785 | 24.487 | 16.996 | 14.644 | 12.312 | 14.463 | 16.958 | 16.451 | 1.73E-13 | 27.011 | 1014.4 | 15.137 |
|  | Median | 400 | 435.51 | 435.28 | 434.78 | 434.78 | 458.78 | 434.93 | 434.78 | 404.34 | 434.78 | 434.78 | 1556.6 | 434.78 |
|  | Rank | 1 | 11 | 10 | 5 | 6 | 12 | 7 | 3 | 2 | 8 | 9 | 13 | 4 |
| C14-F5 | Mean | 519.33 | 520.19 | 519.63 | 520.02 | 520.09 | 520.43 | 520 | 520.01 | 518 | 520.4 | 520.15 | 520.11 | 520 |
|  | Best | 500 | 520.05 | 505.55 | 520 | 520 | 520.24 | 520 | 520 | 500 | 520.27 | 520 | 520.06 | 520 |
|  | Worst | 520.01 | 520.52 | 520.27 | 520.2 | 520.39 | 520.62 | 520 | 520.11 | 520.52 | 520.55 | 520.47 | 520.2 | 520 |
|  | Std. | 3.6516 | 0.12842 | 2.6599 | 0.050517 | 0.13113 | 0.0996 | 0.0002836 | 0.032114 | 6.1606 | 0.078245 | 0.12862 | 0.037359 | 4.20E-05 |
|  | Median | 520 | 520.17 | 520.09 | 520 | 520.01 | 520.43 | 520 | 520 | 520.36 | 520.4 | 520.14 | 520.1 | 520 |
|  | Rank | 2 | 11 | 3 | 7 | 8 | 13 | 4 | 6 | 1 | 12 | 10 | 9 | 5 |
| C14-F6 | Mean | 600.17 | 608.42 | 604.19 | 604.2 | 602.95 | 606.9 | 608.15 | 606.07 | 601.69 | 601.75 | 604.47 | 609.46 | 609.63 |
|  | Best | 600 | 605.65 | 601.71 | 601.3 | 600.15 | 605.09 | 604.15 | 602.59 | 600.03 | 600 | 600.02 | 606.41 | 606.31 |
|  | Worst | 602.03 | 610.86 | 607.47 | 607.71 | 606.27 | 610.1 | 611.23 | 609.7 | 604.12 | 605.26 | 607.4 | 611.17 | 613.51 |
|  | Std. | 0.38402 | 1.3157 | 1.5737 | 1.6428 | 1.4521 | 1.3025 | 1.7676 | 2.0346 | 1.1008 | 1.5902 | 1.9522 | 1.1757 | 1.9612 |
|  | Median | 600.05 | 608.39 | 604.07 | 604.2 | 603.26 | 606.62 | 608.39 | 606.16 | 601.4 | 601.62 | 604.98 | 609.6 | 609.47 |
|  | Rank | 1 | 11 | 5 | 6 | 4 | 9 | 10 | 8 | 2 | 3 | 7 | 12 | 13 |
| C14-F7 | Mean | 700.05 | 701.44 | 703.14 | 700.23 | 700.12 | 710.67 | 710.47 | 700.38 | 700.11 | 700.5 | 701.24 | 816.1 | 700.08 |
|  | Best | 700 | 700.68 | 700.4 | 700.06 | 700.04 | 704.6 | 700.05 | 700.1 | 700.01 | 700.2 | 700.04 | 722.89 | 700 |
|  | Worst | 700.1 | 703.16 | 729.06 | 700.54 | 700.39 | 717.1 | 742.99 | 704.29 | 700.2 | 700.68 | 724.34 | 912.31 | 700.19 |
|  | Std. | 0.028074 | 0.70009 | 5.438 | 0.1212 | 0.074817 | 2.7603 | 11.119 | 0.74319 | 0.052126 | 0.10991 | 4.4845 | 49.342 | 0.0448 |
|  | Median | 700.05 | 701.11 | 701.41 | 700.19 | 700.09 | 710.25 | 707.58 | 700.25 | 700.1 | 700.5 | 700.14 | 817.1 | 700.07 |
|  | Rank | 1 | 9 | 10 | 5 | 4 | 12 | 11 | 6 | 3 | 7 | 8 | 13 | 2 |
| C14-F8 | Mean | 803.15 | 852.2 | 827.65 | 823.71 | 819.7 | 840.72 | 861.83 | 816.93 | 810.28 | 827.05 | 828.63 | 839.22 | 870.11 |
|  | Best | 800 | 828.05 | 806.43 | 805.97 | 803.98 | 828.62 | 822.88 | 805.97 | 802.98 | 817.38 | 807.96 | 814.18 | 825.87 |
|  | Worst | 806.96 | 895.92 | 853.15 | 856.71 | 832.83 | 855.05 | 896.51 | 833.83 | 820.89 | 837.1 | 864.25 | 854.61 | 914.42 |
|  | Std. | 1.5911 | 15.369 | 10.894 | 12.667 | 7.0422 | 6.5846 | 20.509 | 7.0187 | 4.262 | 5.0181 | 13.297 | 9.905 | 21.836 |
|  | Median | 802.98 | 849.45 | 828.9 | 819.9 | 819.4 | 841.3 | 864.7 | 816.42 | 809.95 | 826.95 | 827.4 | 839.32 | 871.14 |
|  | Rank | 1 | 11 | 7 | 5 | 4 | 10 | 12 | 3 | 2 | 6 | 8 | 9 | 13 |
| C14-F9 | Mean | 909.55 | 949.62 | 925.34 | 925.87 | 924.67 | 943.23 | 961.62 | 957.06 | 910.72 | 930.82 | 928.24 | 938.42 | 979.41 |
|  | Best | 903.98 | 928.72 | 911.02 | 908.95 | 904.97 | 922.36 | 926.86 | 917.91 | 902.81 | 918.4 | 909.95 | 921.9 | 930.84 |
|  | Worst | 914.92 | 977.36 | 951.34 | 960.69 | 943.78 | 954.93 | 1000.5 | 993.01 | 923.78 | 940.44 | 967.05 | 953.74 | 1054.2 |
|  | Std. | 2.986 | 12.595 | 9.9631 | 13.737 | 8.9045 | 7.4052 | 17.583 | 20.675 | 4.7466 | 5.7623 | 14.473 | 8.3199 | 26.997 |
|  | Median | 908.95 | 948.24 | 925.55 | 921.89 | 924.87 | 943.14 | 965.24 | 957.71 | 910.41 | 932.01 | 923.44 | 939.32 | 981.59 |
|  | Rank | 1 | 10 | 4 | 5 | 3 | 9 | 12 | 11 | 2 | 7 | 6 | 8 | 13 |

**Table S1.** (continued)

| C14-F10 | Mean | 1085.6 | 1683.8 | 1607.1 | 1613.7 | 1450.1 | 2003.1 | 2096.1 | 1430.9 | 1168.9 | 1086.1 | 1475.3 | 1731 | 2222.6 |
| --- | --- | --- | --- | --- | --- | --- | --- | --- | --- | --- | --- | --- | --- | --- |
|  | Best | 1000.2 | 1320.1 | 1141.1 | 1185.9 | 1003.5 | 1379.4 | 1353.6 | 1129.2 | 1003.7 | 1060.7 | 1073.8 | 1131.3 | 1482.3 |
|  | Worst | 1243.8 | 2171.7 | 2077.2 | 2207.4 | 2034.1 | 2544.3 | 2763.9 | 1841.1 | 1560.4 | 1435.9 | 2116 | 2131.8 | 2947.4 |
|  | Std. | 62.326 | 186.87 | 249.63 | 227.83 | 239.59 | 237.28 | 382.61 | 198.75 | 120.25 | 69.878 | 251.48 | 252.75 | 350.26 |
|  | Median | 1120.7 | 1683.5 | 1633.6 | 1598.8 | 1449.4 | 1981.2 | 2063 | 1375.2 | 1135.8 | 1067.4 | 1440.5 | 1716.2 | 2274.5 |
|  | Rank | 1 | 9 | 7 | 8 | 5 | 11 | 12 | 4 | 3 | 2 | 6 | 10 | 13 |
| C14-F11 | Mean | 1370.3 | 2228.7 | 1847.3 | 1976.8 | 1694.5 | 2526.5 | 2275.8 | 2033.6 | 1702.4 | 2305.9 | 2077.6 | 2014.2 | 2409.9 |
|  | Best | 1131.9 | 1427.4 | 1157 | 1515.6 | 1342.4 | 2127.4 | 1983 | 1552.9 | 1111.9 | 1466.4 | 1376.9 | 1346.4 | 1807.4 |
|  | Worst | 1740.7 | 2845.3 | 2508.6 | 2455.1 | 2169.6 | 2783 | 2725.1 | 2575 | 2312.3 | 2609.2 | 2604.8 | 2551.3 | 3067.7 |
|  | Std. | 160.25 | 346.93 | 285.47 | 241.94 | 229.86 | 179.21 | 210.02 | 246.76 | 368.93 | 262.74 | 281.88 | 275.58 | 326.39 |
|  | Median | 1361.3 | 2236.4 | 1875.2 | 1957.9 | 1679.7 | 2579.8 | 2228.8 | 2017.7 | 1725.8 | 2395.4 | 2034 | 1996.5 | 2365.2 |
|  | Rank | 1 | 9 | 4 | 5 | 2 | 13 | 10 | 7 | 3 | 11 | 8 | 6 | 12 |
| C14-F12 | Mean | 1200 | 1200.9 | 1200.3 | 1200.3 | 1200.1 | 1201.3 | 1200 | 1200.1 | 1201.1 | 1201.2 | 1200.4 | 1200.5 | 1200 |
|  | Best | 1200 | 1200.3 | 1200.1 | 1200.1 | 1200 | 1200.7 | 1200 | 1200 | 1200.6 | 1200.7 | 1200 | 1200.1 | 1200 |
|  | Worst | 1200.1 | 1202.4 | 1200.8 | 1200.8 | 1200.3 | 1201.8 | 1200.1 | 1200.2 | 1201.6 | 1201.5 | 1201.2 | 1201.2 | 1200.1 |
|  | Std. | 0.012152 | 0.42995 | 0.16324 | 0.20813 | 0.085687 | 0.26692 | 0.028172 | 0.049673 | 0.29216 | 0.18234 | 0.24777 | 0.26445 | 0.02455 |
|  | Median | 1200 | 1200.9 | 1200.3 | 1200.2 | 1200.1 | 1201.4 | 1200 | 1200 | 1201.2 | 1201.2 | 1200.3 | 1200.5 | 1200 |
|  | Rank | 1 | 10 | 6 | 7 | 5 | 13 | 2 | 4 | 11 | 12 | 8 | 9 | 3 |
| C14-F13 | Mean | 1300.1 | 1300.5 | 1300.3 | 1300.3 | 1300.2 | 1300.6 | 1300.5 | 1300.3 | 1300.2 | 1300.2 | 1300.4 | 1302.3 | 1300.4 |
|  | Best | 1300 | 1300.2 | 1300.1 | 1300.1 | 1300.1 | 1300.3 | 1300.1 | 1300.1 | 1300.1 | 1300.1 | 1300.1 | 1300.3 | 1300.1 |
|  | Worst | 1300.2 | 1300.9 | 1300.8 | 1300.6 | 1300.6 | 1300.8 | 1301.1 | 1300.8 | 1300.4 | 1300.3 | 1300.7 | 1304.6 | 1301 |
|  | Std. | 0.040254 | 0.17606 | 0.12167 | 0.11471 | 0.1216 | 0.12039 | 0.25166 | 0.17428 | 0.072915 | 0.032415 | 0.15281 | 1.1127 | 0.23002 |
|  | Median | 1300.1 | 1300.4 | 1300.3 | 1300.3 | 1300.2 | 1300.6 | 1300.4 | 1300.3 | 1300.2 | 1300.2 | 1300.4 | 1302.4 | 1300.4 |
|  | Rank | 1 | 10 | 5 | 6 | 2 | 12 | 11 | 7 | 4 | 3 | 8 | 13 | 9 |
| C14-F14 | Mean | 1400.1 | 1400.3 | 1400.4 | 1400.3 | 1400.3 | 1401.1 | 1400.5 | 1400.4 | 1400.3 | 1400.2 | 1400.5 | 1422.6 | 1400.4 |
|  | Best | 1400.1 | 1400.1 | 1400.1 | 1400.1 | 1400.1 | 1400.5 | 1400.2 | 1400.1 | 1400.1 | 1400.1 | 1400.1 | 1403.7 | 1400.1 |
|  | Worst | 1400.2 | 1401 | 1400.7 | 1400.8 | 1400.6 | 1402 | 1401 | 1401.2 | 1400.5 | 1400.3 | 1401.3 | 1444.6 | 1400.8 |
|  | Std. | 0.036225 | 0.25439 | 0.20871 | 0.16356 | 0.11885 | 0.4474 | 0.20709 | 0.26409 | 0.079829 | 0.0526 | 0.31808 | 8.5898 | 0.17844 |
| C14-F15 | Mean | 1500.8 | 1508.8 | 1504.7 | 1501.7 | 1501.1 | 1508.3 | 1533.7 | 1501.1 | 1501.3 | 1502.5 | 1518.5 | 2138.4 | 1502.2 |
|  | Best | 1500.5 | 1501.7 | 1500.8 | 1500.5 | 1500.4 | 1505 | 1501.8 | 1500.4 | 1500.5 | 1501.6 | 1500.7 | 1542.5 | 1500.8 |
|  | Worst | 1501.4 | 1519.1 | 1515.3 | 1504.3 | 1502.5 | 1515.9 | 1598.5 | 1503 | 1502.6 | 1503.6 | 1954.9 | 5590.9 | 1503.6 |
|  | Std. | 0.2259 | 4.6231 | 3.8921 | 0.86942 | 0.48676 | 2.5034 | 25.482 | 0.56504 | 0.5418 | 0.48099 | 82.827 | 889.48 | 0.73914 |
|  | Median | 1500.7 | 1508.7 | 1503.2 | 1501.5 | 1501 | 1507.9 | 1528.2 | 1501 | 1501.2 | 1502.5 | 1501.9 | 1755.9 | 1502.1 |
|  | Rank | 1 | 10 | 8 | 5 | 2 | 9 | 12 | 3 | 4 | 7 | 11 | 13 | 6 |
| C14-F16 | Mean | 1602.2 | 1603.5 | 1603.1 | 1603.1 | 1602.7 | 1603.4 | 1603.9 | 1603.5 | 1602.3 | 1602.9 | 1603.5 | 1603.5 | 1604.1 |
|  | Best | 1601.3 | 1603 | 1602.2 | 1602.5 | 1601.6 | 1602.7 | 1603.3 | 1602.3 | 1601.6 | 1602.3 | 1602.2 | 1602.6 | 1603.4 |
|  | Worst | 1602.8 | 1604.1 | 1604 | 1604 | 1603.5 | 1603.8 | 1604.2 | 1604 | 1603.2 | 1603.7 | 1604.1 | 1604 | 1604.7 |
|  | Std. | 0.39092 | 0.30591 | 0.40693 | 0.40322 | 0.49531 | 0.25013 | 0.21347 | 0.46189 | 0.39581 | 0.38899 | 0.46953 | 0.30539 | 0.30897 |
|  | Median | 1602.3 | 1603.5 | 1603.1 | 1603.1 | 1602.9 | 1603.5 | 1603.9 | 1603.5 | 1602.3 | 1602.9 | 1603.5 | 1603.5 | 1604.2 |
|  | Rank | 1 | 10 | 5 | 6 | 3 | 7 | 12 | 8 | 2 | 4 | 9 | 11 | 13 |
| C14-F17 | Mean | 1717.7 | 3.58E+05 | 40390 | 7644.3 | 4381 | 43215 | 3162.6 | 5051.5 | 2927.3 | 25286 | 1.71E+05 | 2.66E+05 | 5164.6 |
|  | Best | 1701.2 | 7559.4 | 2851.1 | 3097.7 | 2202.4 | 10543 | 2081.9 | 2179.3 | 1860.3 | 2168.1 | 2094.1 | 20843 | 2082.6 |
|  | Worst | 1758.7 | 2.55E+06 | 3.85E+05 | 20377 | 11357 | 3.58E+05 | 9332.1 | 11880 | 6326.3 | 3.68E+05 | 2.59E+06 | 5.65E+05 | 15397 |
|  | Std. | 13.995 | 6.47E+05 | 1.04E+05 | 5276.4 | 2781.6 | 63005 | 1605.2 | 2533.7 | 926.95 | 66594 | 4.84E+05 | 1.55E+05 | 3107.7 |
|  | Median | 1716.7 | 96340 | 4267 | 5493.5 | 3424.1 | 23646 | 2538 | 4204.8 | 2721.6 | 7858.8 | 29972 | 2.42E+05 | 4415.5 |
|  | Rank | 1 | 13 | 9 | 7 | 4 | 10 | 3 | 5 | 2 | 8 | 11 | 12 | 6 |

**Table S1.** (continued)

| C14-F18 | Mean | 1801.1 | 10400 | 16722 | 12425 | 8940.2 | 19072 | 2341.5 | 8779.8 | 3037.2 | 11113 | 13442 | 9095.4 | 11679 |
| --- | --- | --- | --- | --- | --- | --- | --- | --- | --- | --- | --- | --- | --- | --- |
|  | Best | 1800.2 | 2002.4 | 2424.6 | 1966.2 | 1901.3 | 5860.8 | 1852.5 | 1837.6 | 1906.3 | 1807.9 | 1926.7 | 1935.2 | 1861.6 |
|  | Worst | 1803.1 | 32227 | 37710 | 38521 | 24555 | 58361 | 6222.5 | 35380 | 8019.1 | 38811 | 38991 | 29246 | 33726 |
|  | Std. | 0.70879 | 8674.9 | 12749 | 10348 | 6820.7 | 12272 | 970.3 | 8317.9 | 1724.1 | 9909.4 | 13255 | 7189.2 | 9710.1 |
|  | Median | 1801.3 | 8076.8 | 14922 | 9229.7 | 7315.8 | 14726 | 1940.9 | 4798 | 2075.7 | 7372.8 | 6644.7 | 8538.1 | 11360 |
|  | Rank | 1 | 7 | 12 | 10 | 5 | 13 | 2 | 4 | 3 | 8 | 11 | 6 | 9 |
| C14-F19 | Mean | 1900.9 | 1905.8 | 1903.3 | 1903.5 | 1902.4 | 1905.8 | 1913.5 | 1903.9 | 1901.9 | 1901.8 | 1903 | 1925.9 | 1904.5 |
|  | Best | 1900.2 | 1902.8 | 1901.8 | 1901.8 | 1900.1 | 1904 | 1902.7 | 1901.6 | 1900.2 | 1901 | 1901.1 | 1906.6 | 1901.6 |
|  | Worst | 1901.5 | 1909.9 | 1906.7 | 1905.9 | 1906.7 | 1907.2 | 1966.7 | 1908.2 | 1904.2 | 1904.1 | 1905.4 | 1961.9 | 1907.9 |
|  | Std. | 0.33956 | 1.6068 | 1.1948 | 1.0864 | 1.2133 | 0.73971 | 13.156 | 1.6565 | 0.97364 | 0.6353 | 0.9929 | 18.275 | 1.3838 |
|  | Median | 1901 | 1905.5 | 1903.3 | 1903.5 | 1902.6 | 1905.9 | 1909.1 | 1903.3 | 1901.7 | 1901.8 | 1902.8 | 1917.6 | 1904.5 |
|  | Rank | 1 | 11 | 6 | 7 | 4 | 10 | 12 | 8 | 3 | 2 | 5 | 13 | 9 |
| C14-F20 | Mean | 2000.8 | 9339.6 | 7385.6 | 5278.8 | 4714.7 | 5192.7 | 2656.5 | 8474.9 | 2163.6 | 7396.9 | 15970 | 9279.1 | 11332 |
|  | Best | 2000.1 | 3198.9 | 2094.5 | 2090.4 | 2019.9 | 2063.8 | 2020.9 | 2064.3 | 2036.2 | 2168.9 | 2203.3 | 3085 | 2567.7 |
|  | Worst | 2001.9 | 26691 | 14139 | 21662 | 21114 | 11519 | 9512.5 | 22012 | 2696.9 | 30879 | 34687 | 17553 | 31091 |
|  | Std. | 0.48807 | 5559.2 | 4366.5 | 4272.7 | 4342.4 | 2680.7 | 1463.7 | 6478 | 131.84 | 7396 | 13897 | 4001 | 9852.6 |
|  | Median | 2000.6 | 8215 | 7825.7 | 3717.9 | 3194.6 | 4538.6 | 2237.4 | 5403.1 | 2130.8 | 5039 | 8594.6 | 9194.1 | 5076.3 |
|  | Rank | 1 | 11 | 7 | 6 | 4 | 5 | 3 | 9 | 2 | 8 | 13 | 10 | 12 |
| C14-F21 | Mean | 2101.5 | 1.57E+05 | 10975 | 7393.6 | 2681.6 | 12194 | 2881 | 10371 | 2259.3 | 5938.4 | 20378 | 1.48E+05 | 8347.6 |
|  | Best | 2100.1 | 4018.6 | 2988.5 | 2413.3 | 2135.6 | 4416.6 | 2258.2 | 2372.1 | 2106.1 | 2355.1 | 2246.1 | 3175.8 | 2265.7 |
|  | Worst | 2116.8 | 9.05E+05 | 25996 | 27769 | 3644.8 | 42396 | 4444.7 | 32326 | 2579.7 | 24267 | 1.69E+05 | 2.75E+06 | 22705 |
|  | Std. | 2.9296 | 2.09E+05 | 5369.3 | 6629.6 | 452.56 | 8088.5 | 476.77 | 9041.1 | 96.456 | 6022.9 | 33715 | 5.14E+05 | 6702 |
|  | Median | 2100.9 | 49479 | 11052 | 4506.9 | 2574.8 | 10453 | 2748.5 | 7373.5 | 2226.8 | 3119.6 | 10045 | 7673.3 | 4597 |
|  | Rank | 1 | 13 | 9 | 6 | 3 | 10 | 4 | 8 | 2 | 5 | 11 | 12 | 7 |
| C14-F22 | Mean | 2212.5 | 2305.2 | 2322.9 | 2274.3 | 2324.3 | 2276.6 | 2543.1 | 2378.7 | 2226.6 | 2220.5 | 2260.8 | 2391.3 | 2543.6 |
|  | Best | 2200.2 | 2226.4 | 2229.6 | 2225.1 | 2217 | 2236.5 | 2280.8 | 2217 | 2201 | 2200 | 2220.2 | 2247 | 2227.3 |
|  | Worst | 2220.7 | 2493.1 | 2477.4 | 2376.8 | 2390.4 | 2388.4 | 2897.4 | 2781.7 | 2341.2 | 2225.2 | 2464.9 | 2591.2 | 2922.5 |
|  | Std. | 9.7368 | 83.768 | 64.882 | 50.053 | 52.235 | 35.602 | 140.91 | 151.43 | 24.51 | 8.0882 | 62.748 | 83.986 | 204.74 |
|  | Median | 2220.1 | 2256.5 | 2349.5 | 2252.3 | 2340.7 | 2267.6 | 2535.8 | 2360 | 2222.2 | 2223.4 | 2243 | 2354.1 | 2494.4 |
|  | Rank | 1 | 7 | 8 | 5 | 9 | 6 | 12 | 10 | 3 | 2 | 4 | 11 | 13 |
| C14-F23 | Mean | 2500 | 2611 | 2637.3 | 2629.9 | 2629.5 | 2642.9 | 2631 | 2630.2 | 2629.5 | 2629.5 | 2635.8 | 2500 | 2628.8 |
|  | Best | 2500 | 2500 | 2630.2 | 2629.5 | 2629.5 | 2633.4 | 2629.5 | 2629.5 | 2629.5 | 2629.5 | 2629.5 | 2500 | 2600 |
|  | Worst | 2500 | 2639.9 | 2654.2 | 2643.6 | 2629.5 | 2653.4 | 2644.3 | 2642.4 | 2629.5 | 2629.5 | 2666.3 | 2500 | 2636.6 |
|  | Std. | 0 | 50.571 | 5.5458 | 2.5739 | 1.15E-12 | 4.7028 | 3.9232 | 2.9926 | 1.03E-12 | 9.25E-13 | 9.9437 | 0 | 5.6201 |
|  | Median | 2500 | 2631.9 | 2636.5 | 2629.5 | 2629.5 | 2642.7 | 2629.5 | 2629.5 | 2629.5 | 2629.5 | 2629.5 | 2500 | 2629.5 |
|  | Rank | 1 | 3 | 12 | 8 | 5 | 13 | 10 | 9 | 7 | 6 | 11 | 2 | 4 |
| C14-F24 | Mean | 2513.5 | 2584.3 | 2546.2 | 2532.4 | 2554.9 | 2556 | 2594.8 | 2584.2 | 2525.7 | 2536.6 | 2543.4 | 2585.4 | 2618 |
|  | Best | 2508.9 | 2539.5 | 2518.1 | 2510.2 | 2512.4 | 2543.2 | 2561 | 2541 | 2507.4 | 2529.8 | 2517.9 | 2527.7 | 2549.6 |
|  | Worst | 2522.1 | 2617.6 | 2608.3 | 2591.7 | 2608 | 2568.6 | 2638.5 | 2612.6 | 2603.2 | 2545.8 | 2608 | 2600 | 2702.1 |
|  | Std. | 3.0481 | 25.099 | 26.738 | 16.617 | 33.281 | 7.5681 | 22.146 | 22.144 | 26.929 | 4.2021 | 20.006 | 20.997 | 36.157 |
|  | Median | 2513.3 | 2597.8 | 2536.5 | 2528.8 | 2547.6 | 2555.9 | 2600.6 | 2584 | 2516.5 | 2536.3 | 2536.9 | 2600 | 2618.1 |
|  | Rank | 1 | 10 | 6 | 3 | 7 | 8 | 12 | 9 | 2 | 4 | 5 | 11 | 13 |

**Table S1.** (continued)

| C14-F25 | Mean | 2625 | 2697.3 | 2695.5 | 2687.6 | 2693.2 | 2699.6 | 2703.7 | 2699.5 | 2643.2 | 2701.6 | 2696.3 | 2698 | 2706.5 |
| --- | --- | --- | --- | --- | --- | --- | --- | --- | --- | --- | --- | --- | --- | --- |
|  | Best | 2613.5 | 2676.3 | 2644.3 | 2640.3 | 2640.5 | 2660.3 | 2681.8 | 2641.4 | 2614.2 | 2699.6 | 2624.8 | 2676.6 | 2683.5 |
|  | Worst | 2639.2 | 2702.2 | 2704.2 | 2702.9 | 2703.9 | 2704.2 | 2722.7 | 2705.8 | 2701.6 | 2703.2 | 2704.4 | 2700 | 2733.6 |
|  | Std. | 7.0756 | 5.8435 | 14.71 | 20.361 | 16.122 | 9.2848 | 7.0624 | 12.708 | 26.05 | 0.56031 | 18.254 | 5.9074 | 8.0159 |
|  | Median | 2623.4 | 2700 | 2700.2 | 2699.7 | 2700.1 | 2702.3 | 2703.8 | 2702.9 | 2633.1 | 2701.6 | 2702.5 | 2700 | 2704.8 |
|  | Rank | 1 | 7 | 5 | 3 | 4 | 10 | 12 | 9 | 2 | 11 | 6 | 8 | 13 |
| C14-F26 | Mean | 2700.1 | 2700.4 | 2703.6 | 2700.3 | 2713.5 | 2700.7 | 2703.7 | 2700.4 | 2700.2 | 2700.2 | 2700.3 | 2706 | 2727.9 |
|  | Best | 2700 | 2700.1 | 2700.2 | 2700.1 | 2700 | 2700.4 | 2700.1 | 2700.1 | 2700.1 | 2700.1 | 2700.1 | 2701.9 | 2700.1 |
|  | Worst | 2700.2 | 2700.7 | 2800 | 2700.8 | 2800 | 2701.2 | 2800 | 2701 | 2700.3 | 2700.3 | 2700.7 | 2800 | 2927.8 |
|  | Std. | 0.032726 | 0.14794 | 18.208 | 0.13478 | 34.518 | 0.15201 | 18.2 | 0.22282 | 0.049122 | 0.03811 | 0.16239 | 17.757 | 55.268 |
|  | Median | 2700.1 | 2700.3 | 2700.2 | 2700.3 | 2700.2 | 2700.7 | 2700.2 | 2700.3 | 2700.2 | 2700.2 | 2700.2 | 2702.7 | 2700.4 |
|  | Rank | 1 | 6 | 9 | 4 | 12 | 8 | 10 | 7 | 2 | 3 | 5 | 11 | 13 |
| C14-F27 | Mean | 2841.4 | 3111.5 | 3072.4 | 2944.5 | 3045.7 | 3055 | 3233.8 | 3141.9 | 2945.5 | 3005.4 | 3049 | 2891.8 | 3220.4 |
|  | Best | 2701.8 | 2708.2 | 2704.5 | 2702.1 | 2701.7 | 2718.1 | 2704.3 | 2703.3 | 2702.2 | 3000 | 2706.5 | 2728.5 | 2702.9 |
|  | Worst | 3100.1 | 3276.4 | 3146.8 | 3186 | 3210.5 | 3111.8 | 3439.2 | 3298.8 | 3102.8 | 3017.4 | 3213.8 | 2900 | 3409.9 |
|  | Std. | 186.03 | 119.98 | 103.44 | 200.57 | 145.02 | 131.89 | 144.06 | 104.97 | 175.19 | 5.9636 | 158.52 | 33.608 | 175.53 |
|  | Median | 2703.2 | 3108.4 | 3101.2 | 3100.6 | 3092.9 | 3104.7 | 3265.7 | 3142.8 | 3033.6 | 3002.8 | 3102.3 | 2900 | 3280.8 |
|  | Rank | 1 | 10 | 9 | 3 | 6 | 8 | 13 | 11 | 4 | 5 | 7 | 2 | 12 |
| C14-F28 | Mean | 3000 | 3441.8 | 3337.5 | 3219.3 | 3421.8 | 3279.7 | 3888.8 | 3657.4 | 3230.7 | 3186.6 | 3210.3 | 3000 | 4181.4 |
|  | Best | 3000 | 3000 | 3178.6 | 3168.9 | 2900.5 | 3204.1 | 3474.4 | 3292 | 3178.3 | 3169.4 | 3178.4 | 3000 | 3531.1 |
|  | Worst | 3000 | 3799.5 | 3816.6 | 3337 | 3921.2 | 3384.9 | 4221.5 | 4119.4 | 3340.7 | 3280.3 | 3336.2 | 3000 | 5170.8 |
|  | Std. | 0 | 154.77 | 143.46 | 61.618 | 221.93 | 58.403 | 218.72 | 213.39 | 52.913 | 25.469 | 41.514 | 0 | 422.18 |
|  | Median | 3000 | 3485.5 | 3304.5 | 3179.1 | 3338.3 | 3255.1 | 3890.4 | 3628 | 3203.3 | 3180.6 | 3193.7 | 3000 | 4168.7 |
|  | Rank | 1 | 10 | 8 | 5 | 9 | 7 | 12 | 11 | 6 | 3 | 4 | 2 | 13 |
| C14-F29 | Mean | 3108.8 | 7.22E+05 | 8.06E+05 | 1.41E+05 | 5.12E+05 | 13599 | 1.09E+06 | 1.44E+06 | 1.18E+05 | 3717.3 | 4203.9 | 1.12E+06 | 1.98E+06 |
|  | Best | 3032.4 | 3361.9 | 3156.6 | 3429.9 | 3148.3 | 3491 | 3155.8 | 3260 | 3342.5 | 3453.6 | 3259 | 3100 | 3254.6 |
|  | Worst | 3123.6 | 3.67E+06 | 4.65E+06 | 2.05E+06 | 3.88E+06 | 46504 | 5.86E+06 | 8.92E+06 | 1.73E+06 | 4069.8 | 9628.5 | 1.51E+07 | 9.02E+06 |
|  | Std. | 28.596 | 1.12E+06 | 1.36E+06 | 5.18E+05 | 1.12E+06 | 11736 | 1.53E+06 | 2.25E+06 | 4.37E+05 | 140.47 | 1216.8 | 3.10E+06 | 2.58E+06 |
|  | Median | 3122 | 4733.9 | 4616.5 | 4797.7 | 3300.5 | 7865.2 | 3354.6 | 3859.4 | 3486.5 | 3709.3 | 3886.9 | 3333.8 | 9266.9 |
|  | Rank | 1 | 8 | 9 | 6 | 7 | 4 | 10 | 12 | 5 | 2 | 3 | 11 | 13 |
| C14-F30 | Mean | 3478.7 | 5933.2 | 4900.4 | 4540.1 | 4656.2 | 4799.5 | 4668.9 | 4618.4 | 3680.3 | 3522 | 3849.8 | 26382 | 5175.8 |
|  | Best | 3374.5 | 4311.2 | 3575.7 | 3548 | 3999.3 | 4040.6 | 3857.9 | 3762.9 | 3468.7 | 3459.7 | 3486.3 | 3200 | 3774.4 |
|  | Worst | 3536.5 | 9783.7 | 6795.9 | 6115.3 | 5526.7 | 6475.2 | 5714.3 | 5827.8 | 4329.1 | 3616.2 | 4538.6 | 1.75E+05 | 6798.2 |
|  | Std. | 29.027 | 1204 | 815.46 | 750.27 | 373.41 | 642.03 | 426.61 | 493.79 | 244.2 | 50.486 | 338.02 | 39664 | 855.09 |
|  | Median | 3473.7 | 5745.2 | 4782.7 | 4368.7 | 4695.3 | 4600.7 | 4620.4 | 4546.5 | 3601.2 | 3497.3 | 3760 | 9110.3 | 5151.4 |
|  | Rank | 1 | 12 | 10 | 5 | 7 | 9 | 8 | 6 | 3 | 2 | 4 | 13 | 11 |
| Sum rank | | 31 | 288 | 231 | 174 | 147 | 289 | 266 | 219 | 94 | 171 | 237 | 299 | 284 |
| Mean rank | | 1.0333 | 9.6000 | 7.7000 | 5.8000 | 4.9000 | 9.6333 | 8.8666 | 7.3000 | 3.1333 | 5.7000 | 7.9000 | 9.9666 | 9.4666 |
| Total rank | | 1 | 11 | 7 | 5 | 3 | 12 | 9 | 6 | 2 | 4 | 8 | 13 | 10 |

**Table S1.** (continued)

| 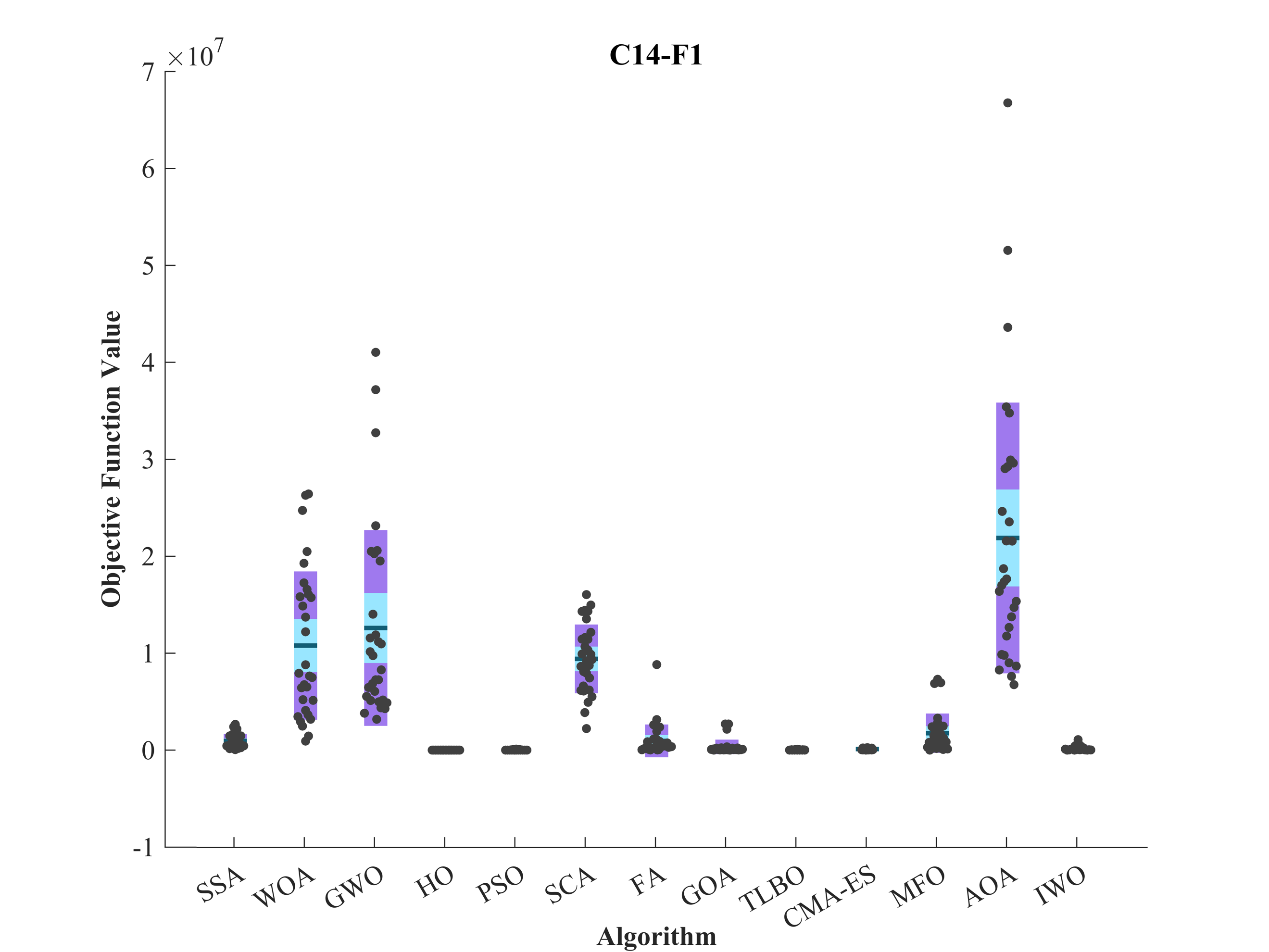 | 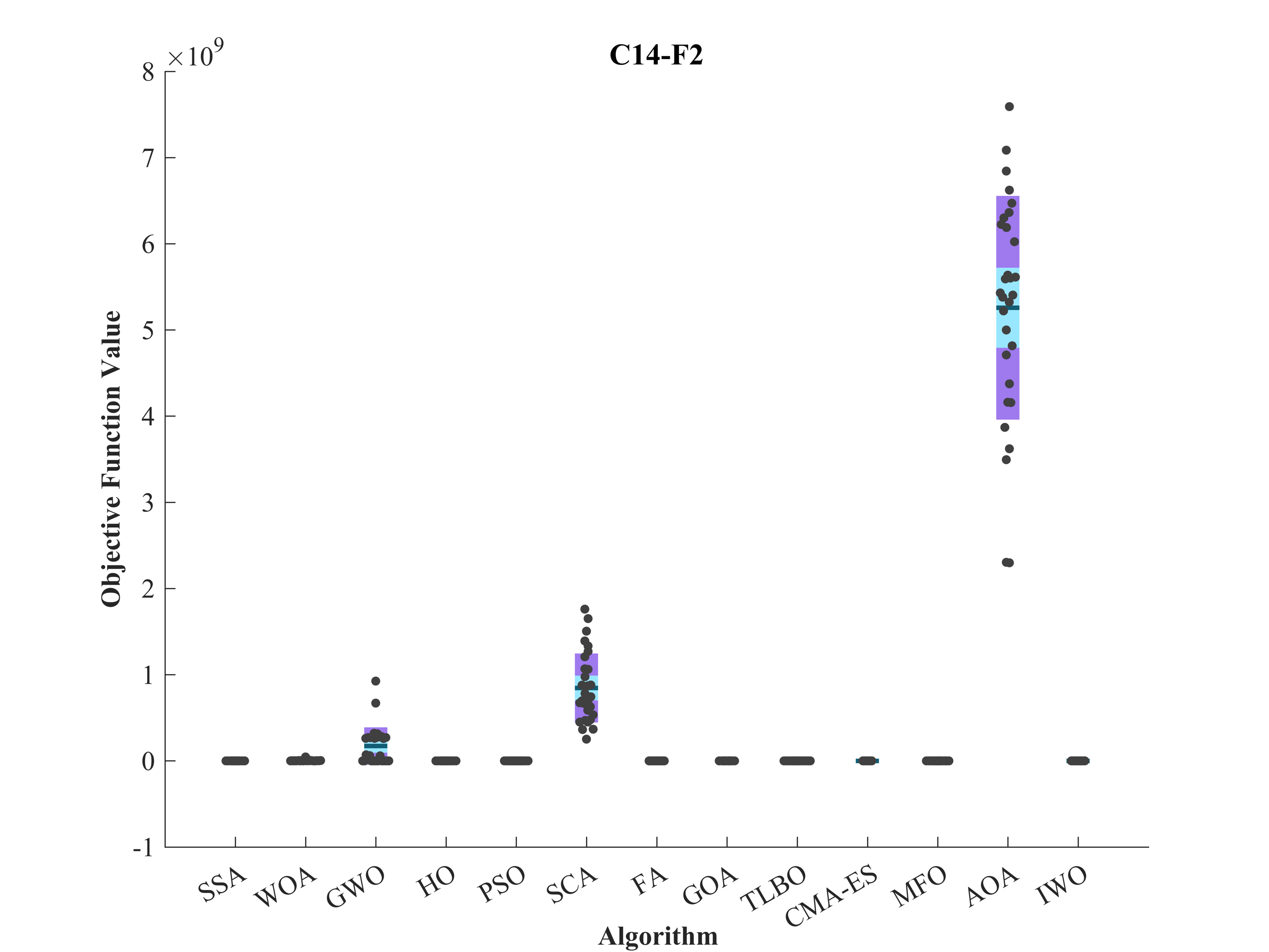 |
| --- | --- |
| 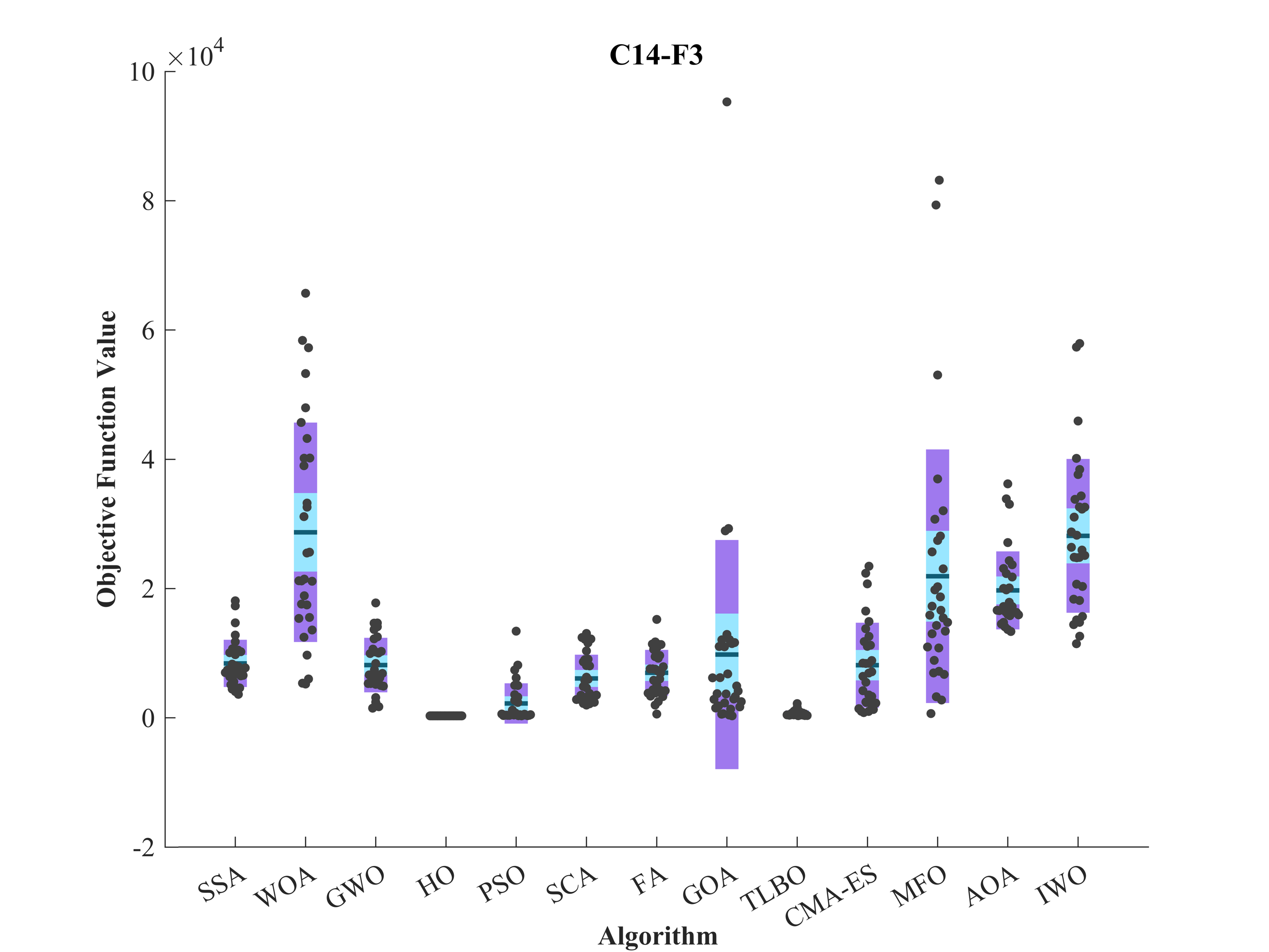 | 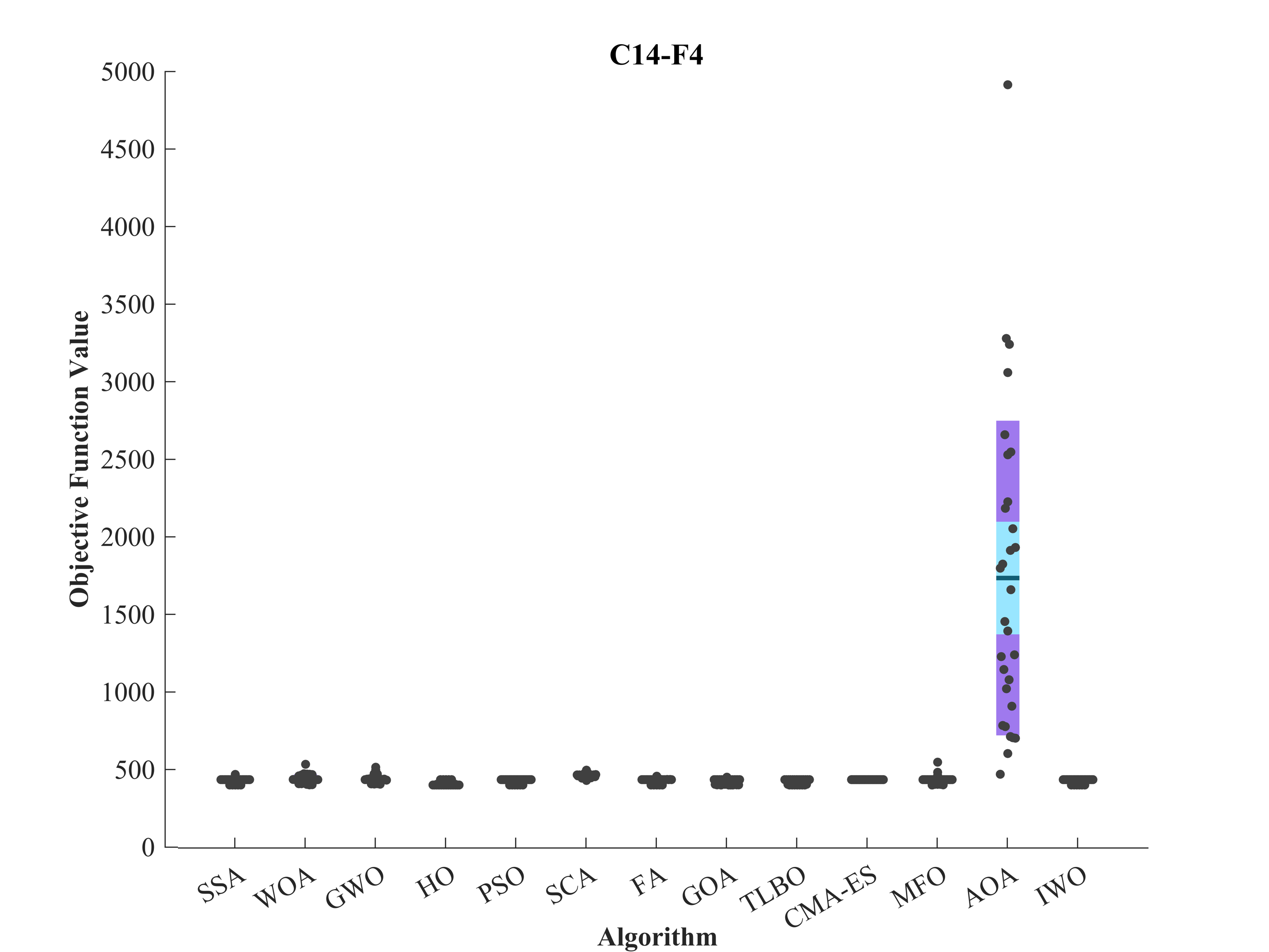 |
| 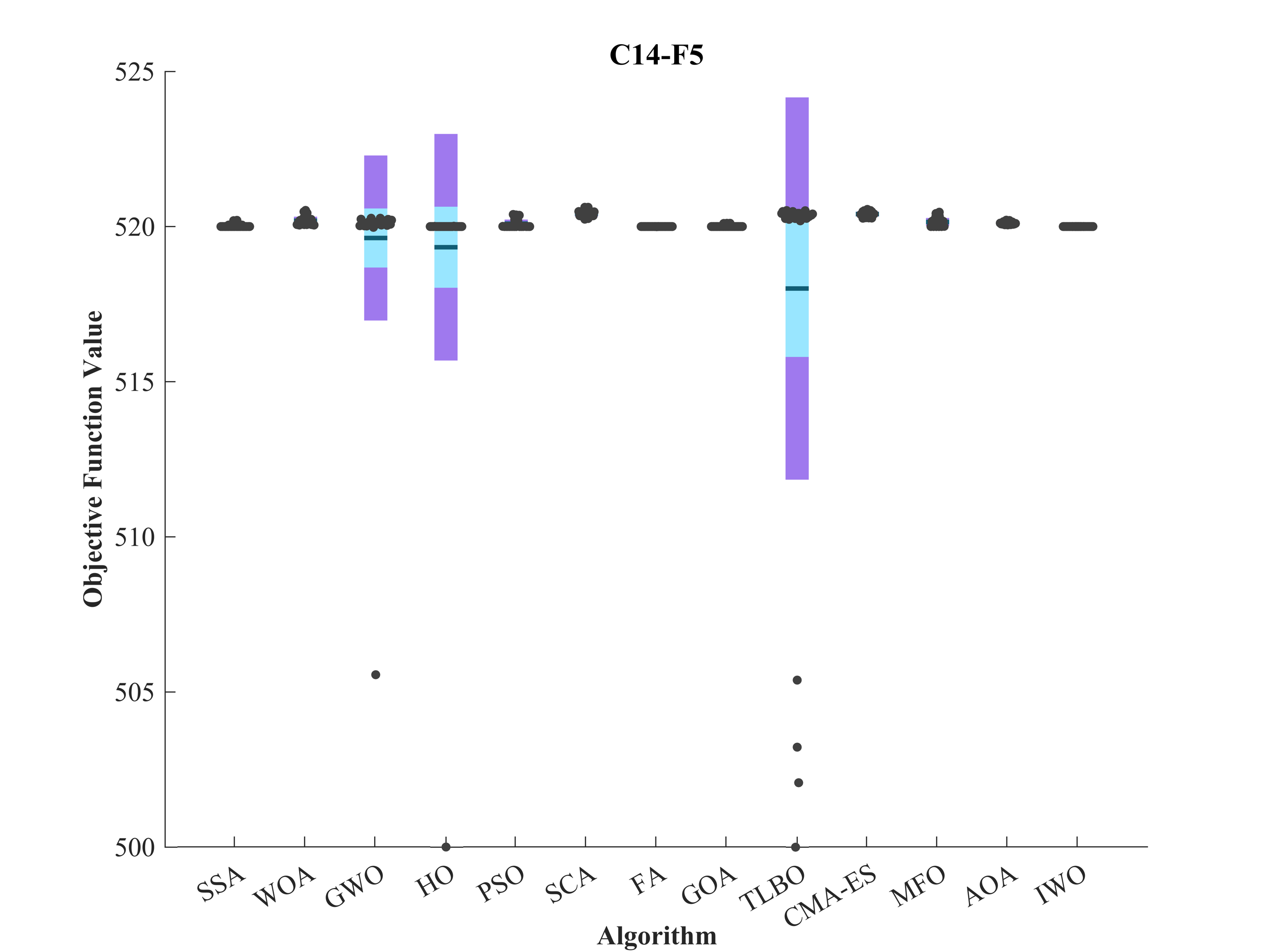 | 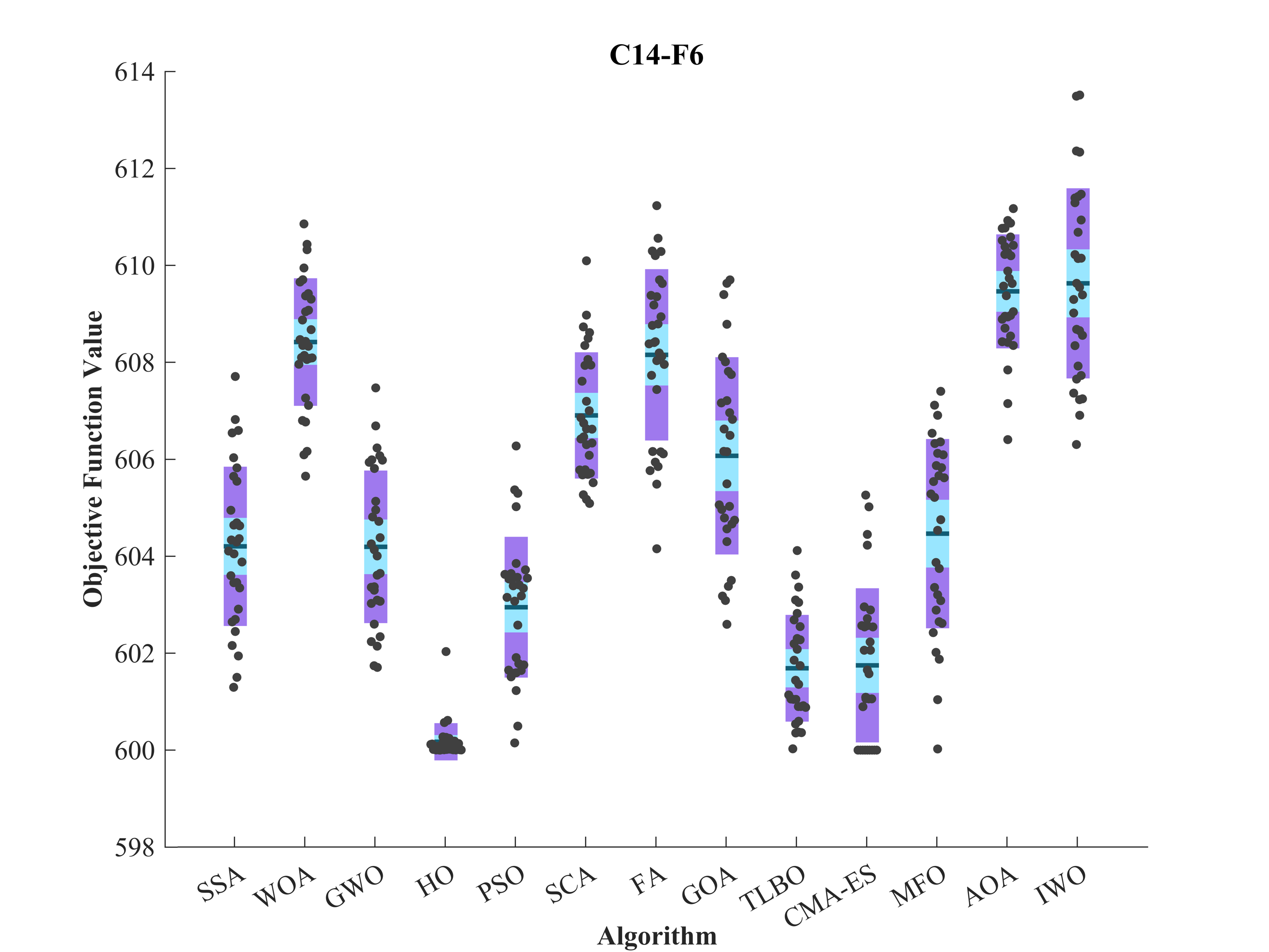 |
| 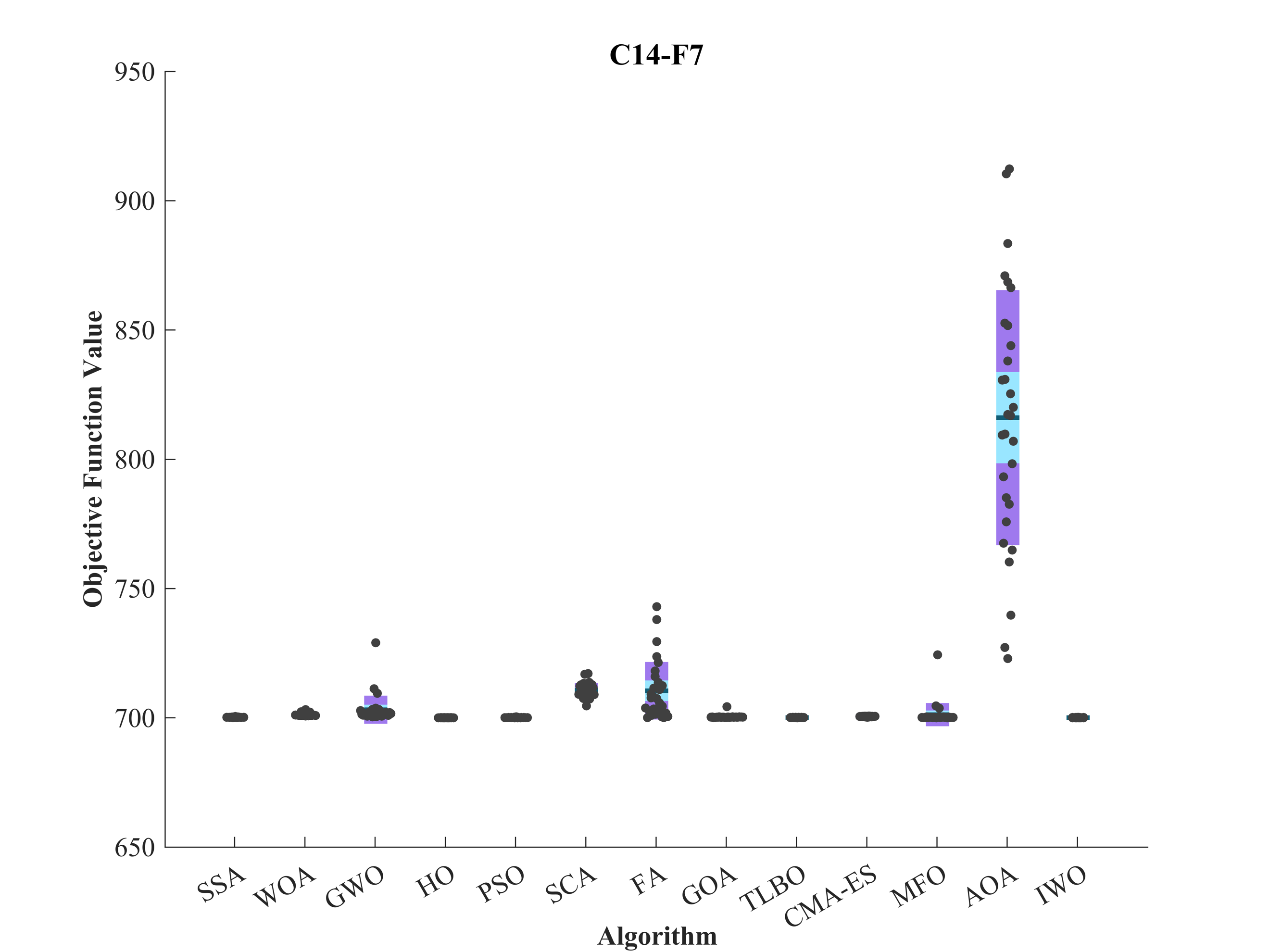 | 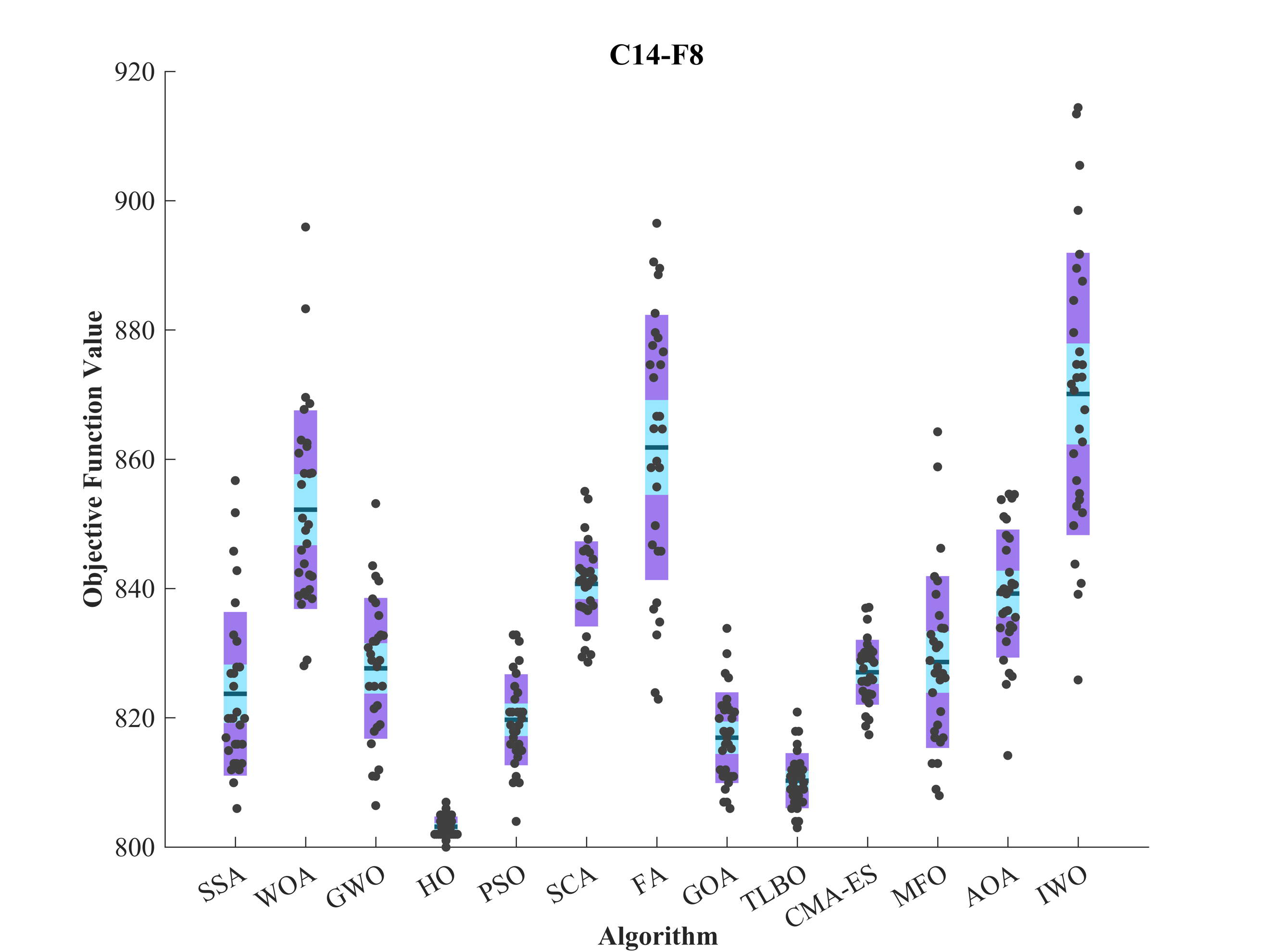 |

**Figure S2.** Boxplot illustrating the performance of the HO in comparison to competing algorithms for optimizing CEC 2014 (D = 10).

| 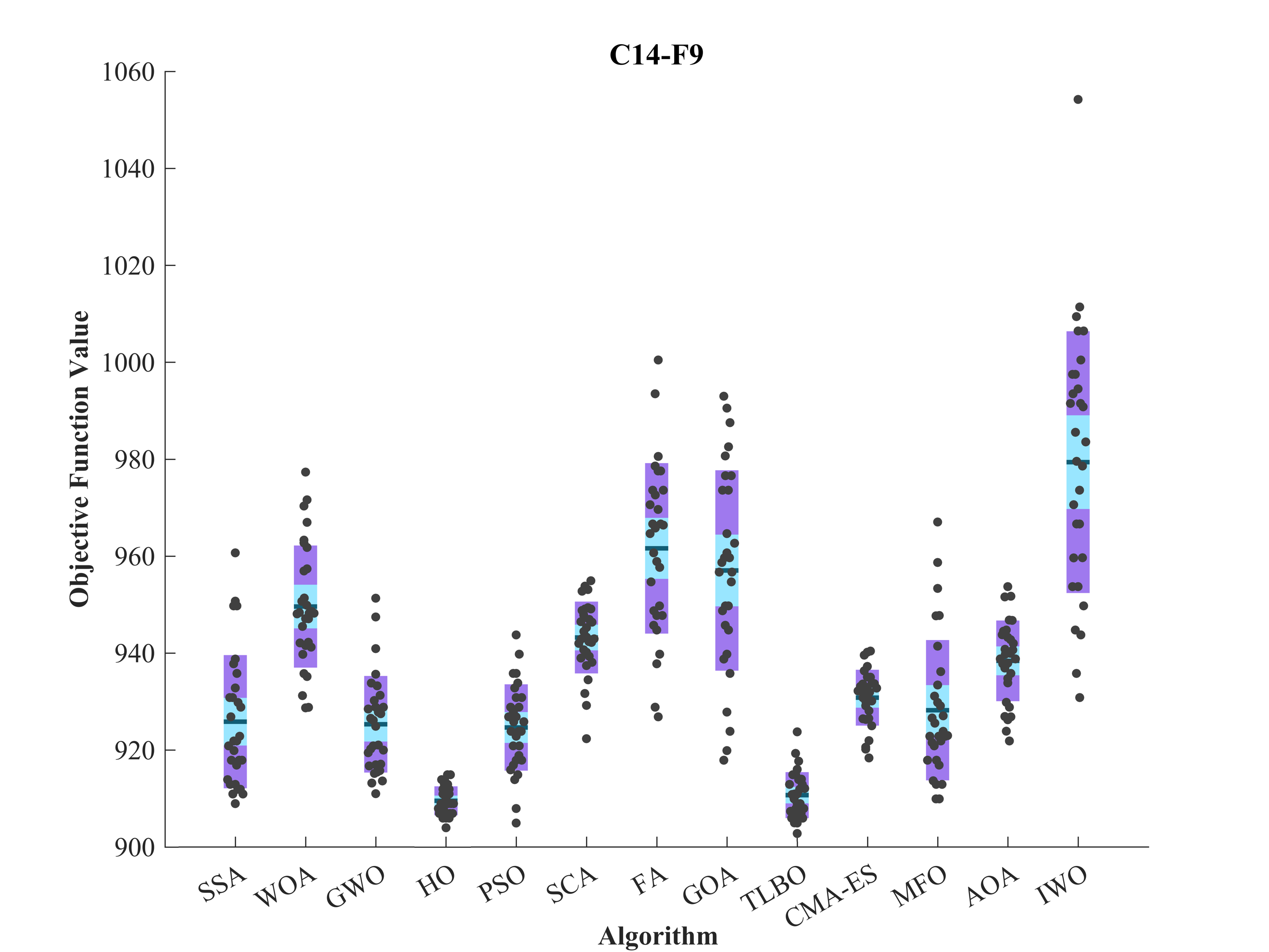 | 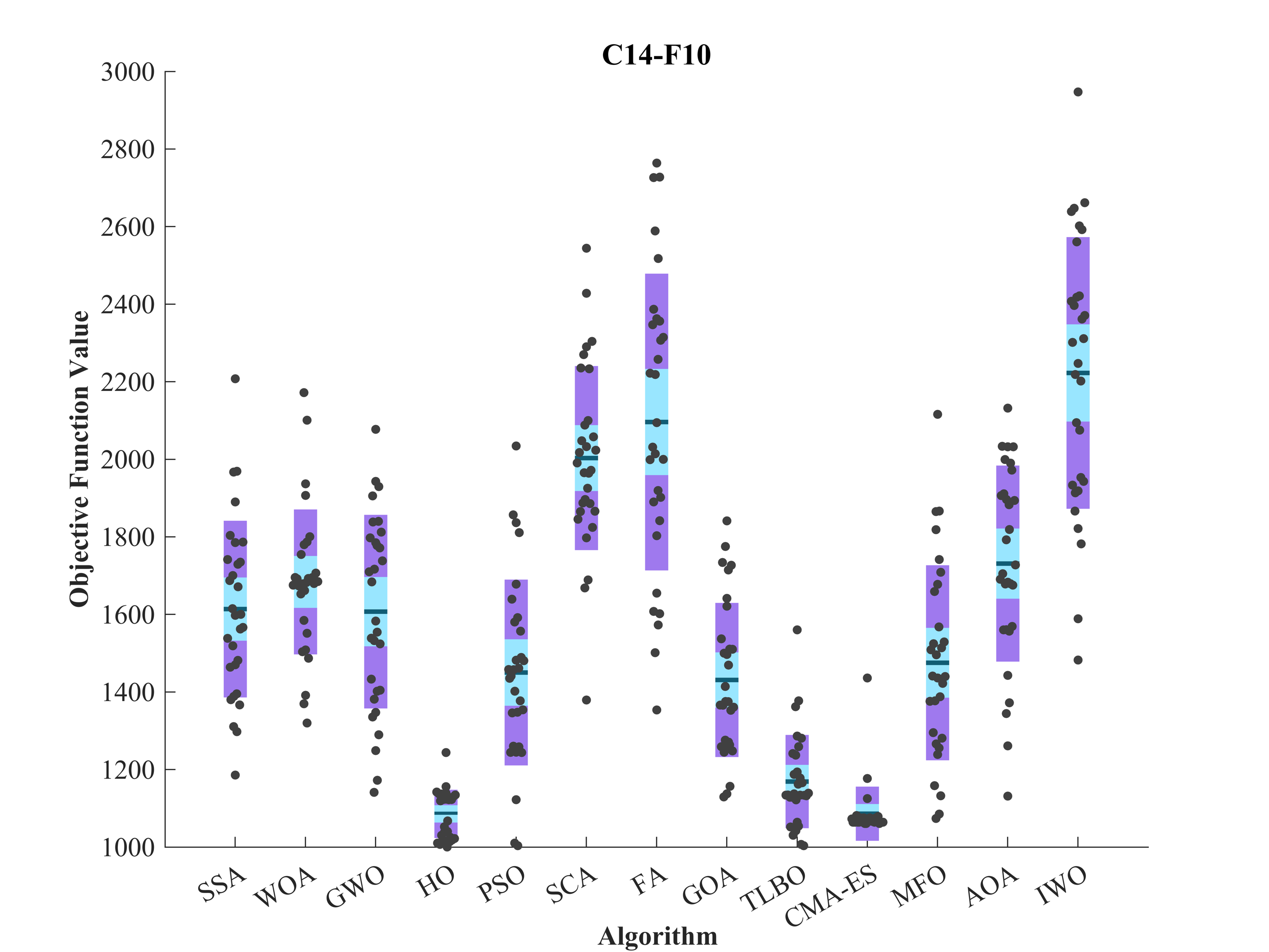 |
| --- | --- |
| 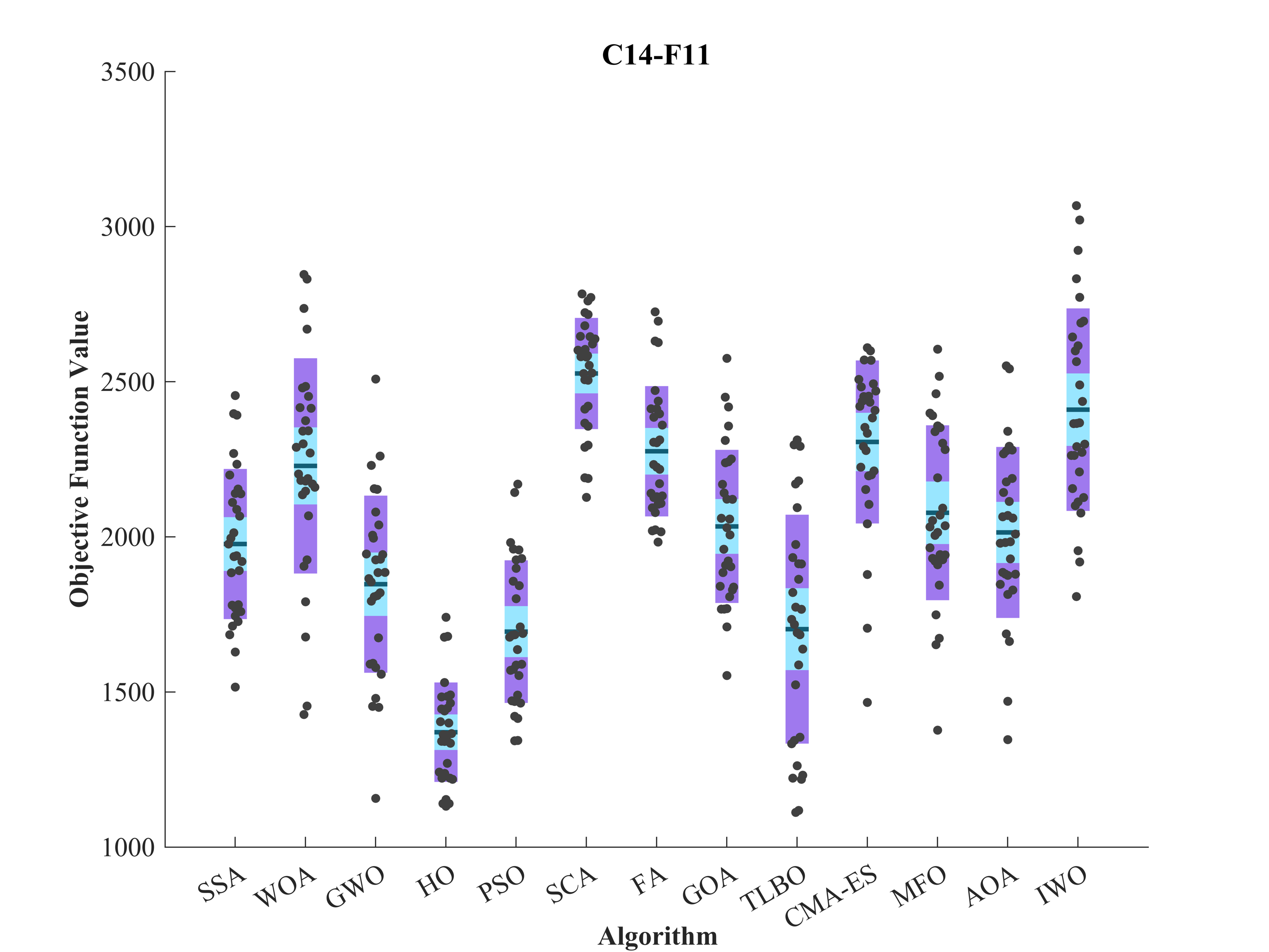 | 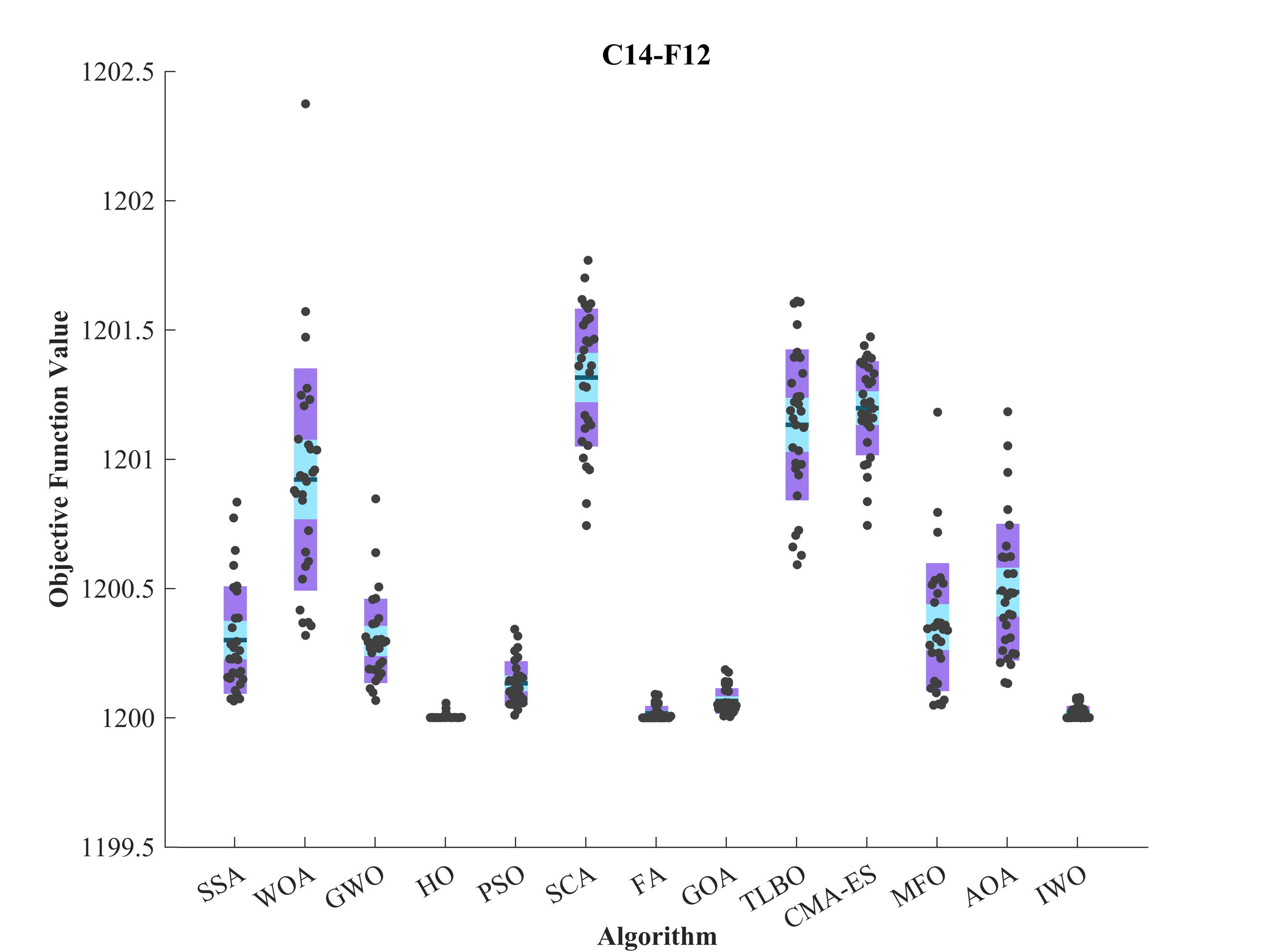 |
| 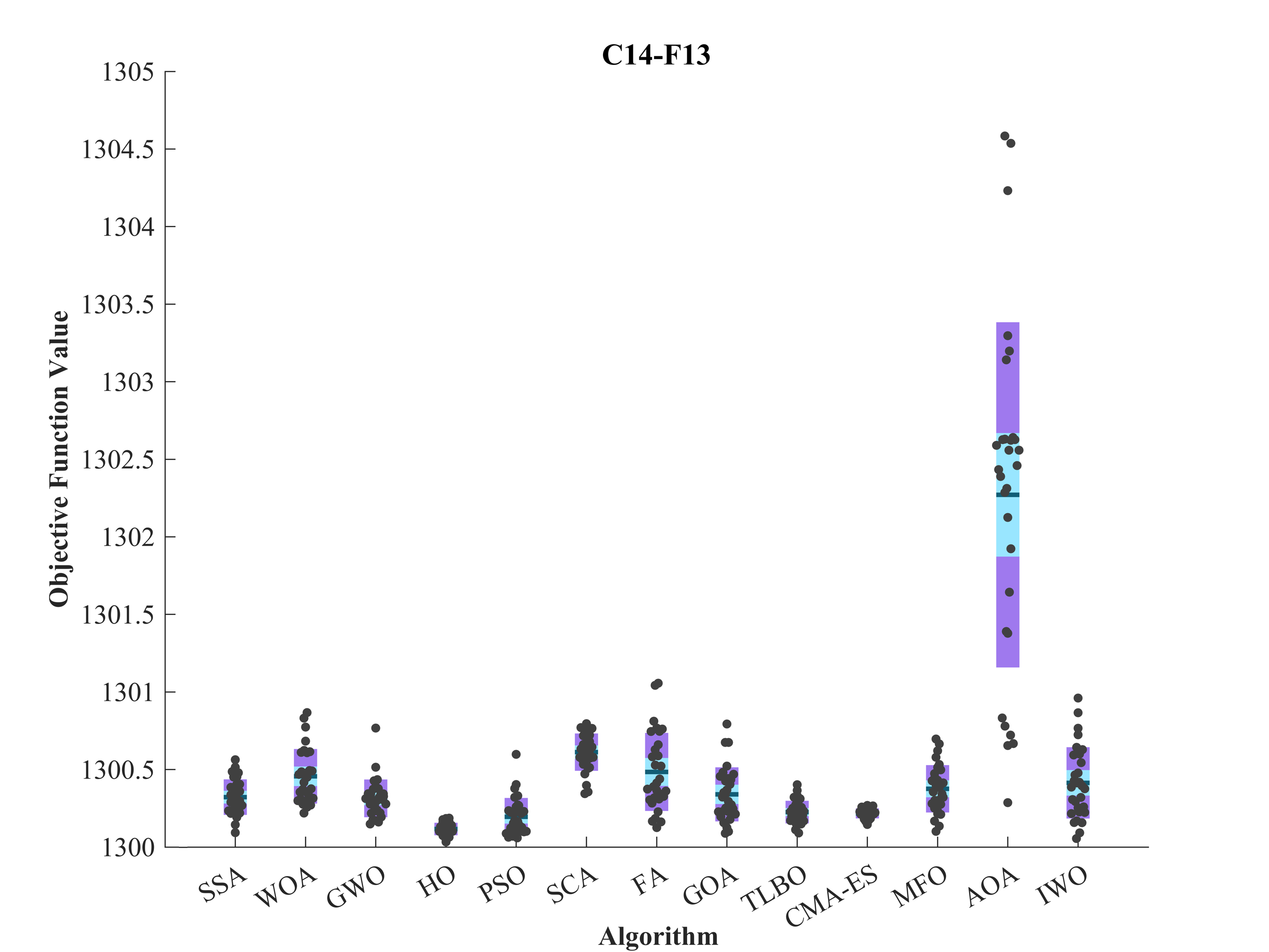 | 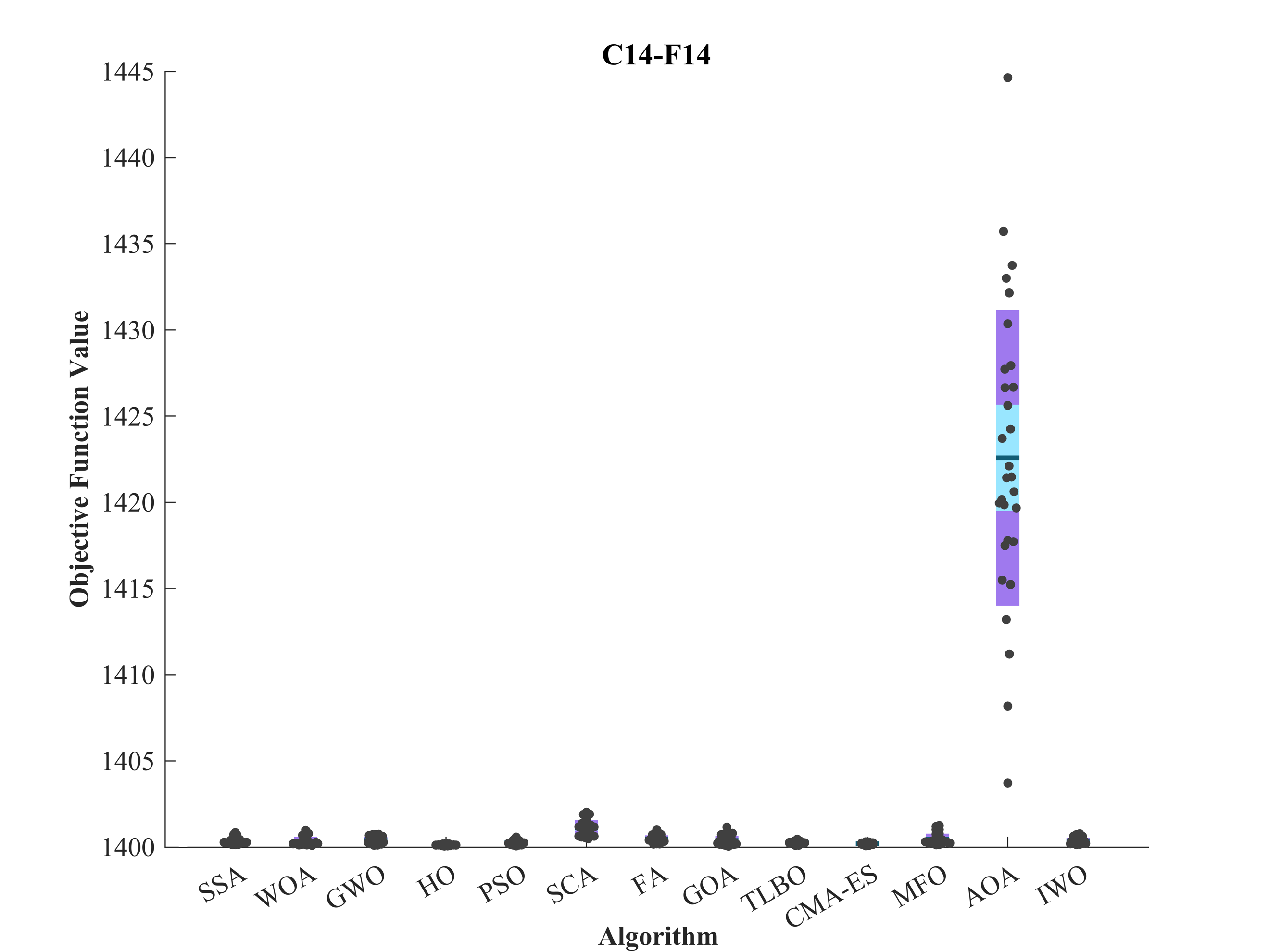 |
| 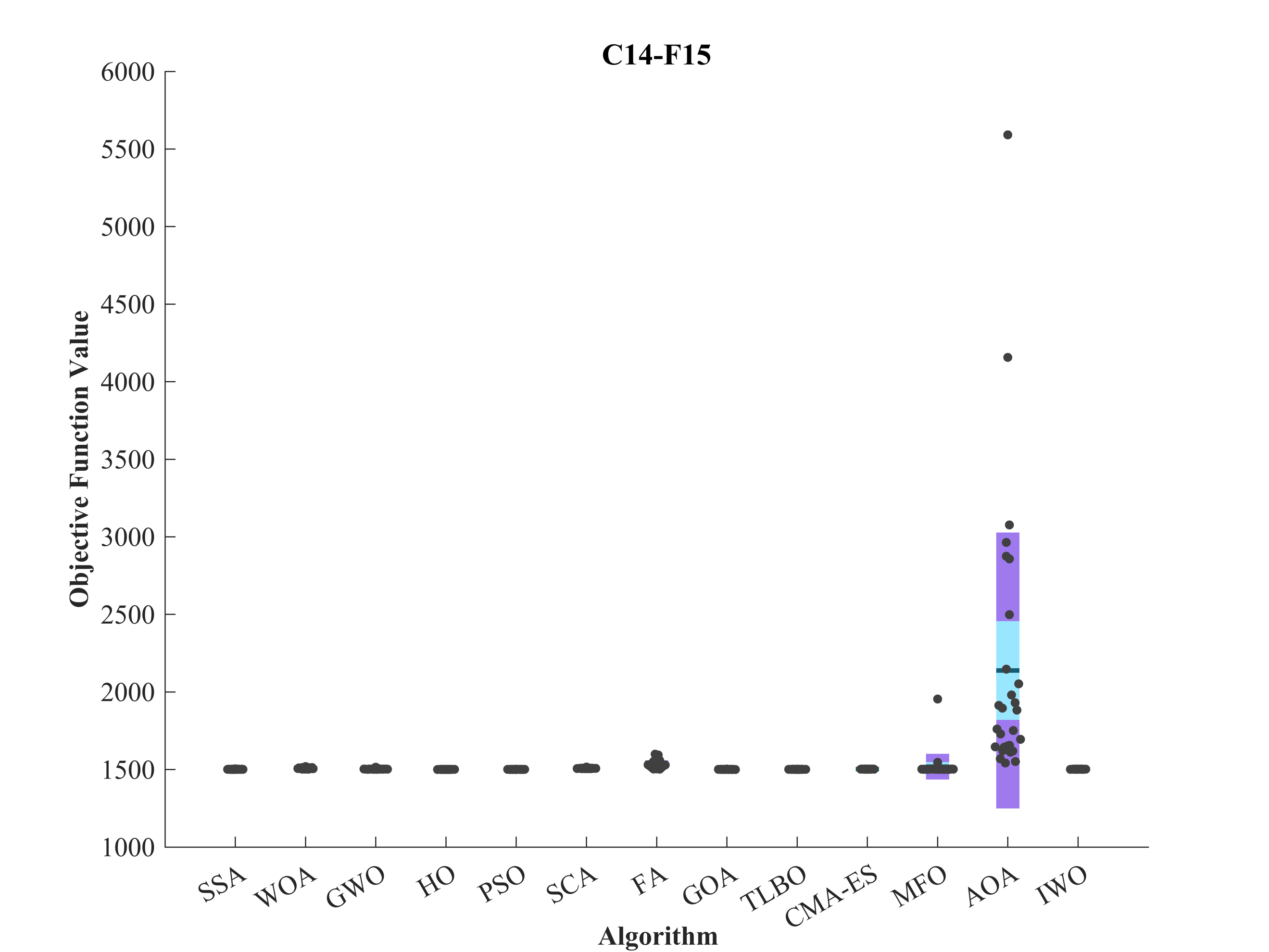 | 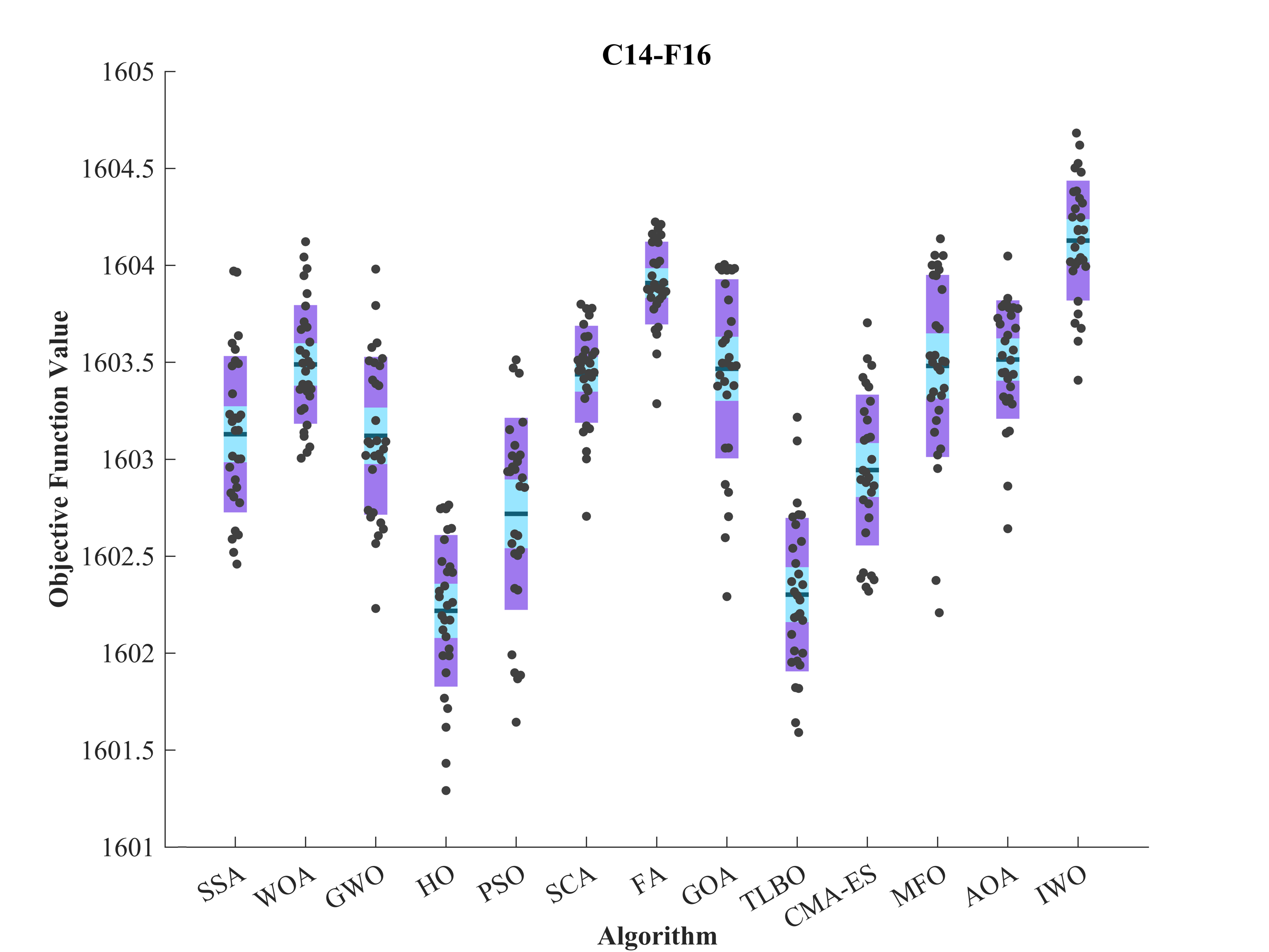 |

**Figure S2.** (continued)

| 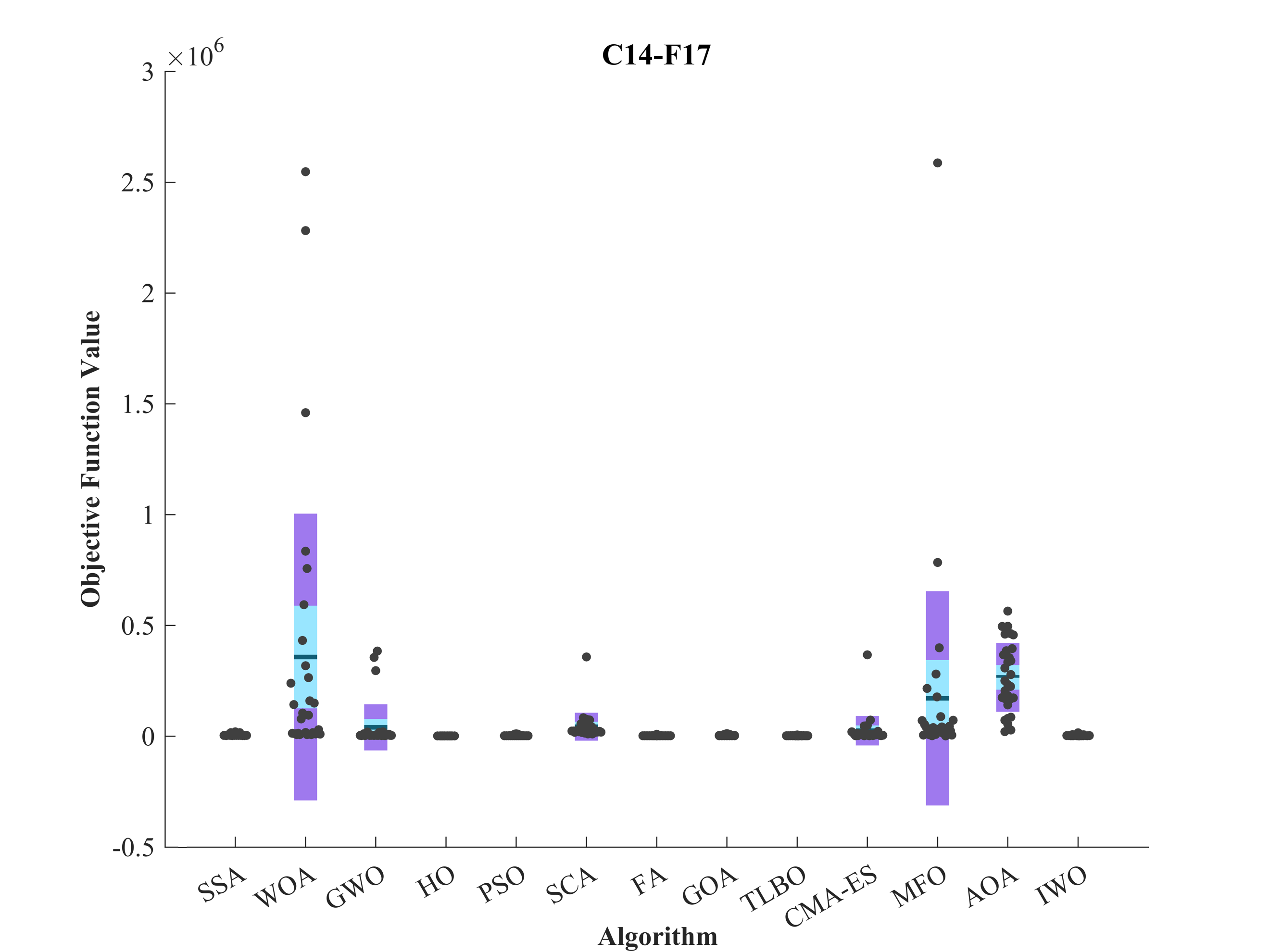 | 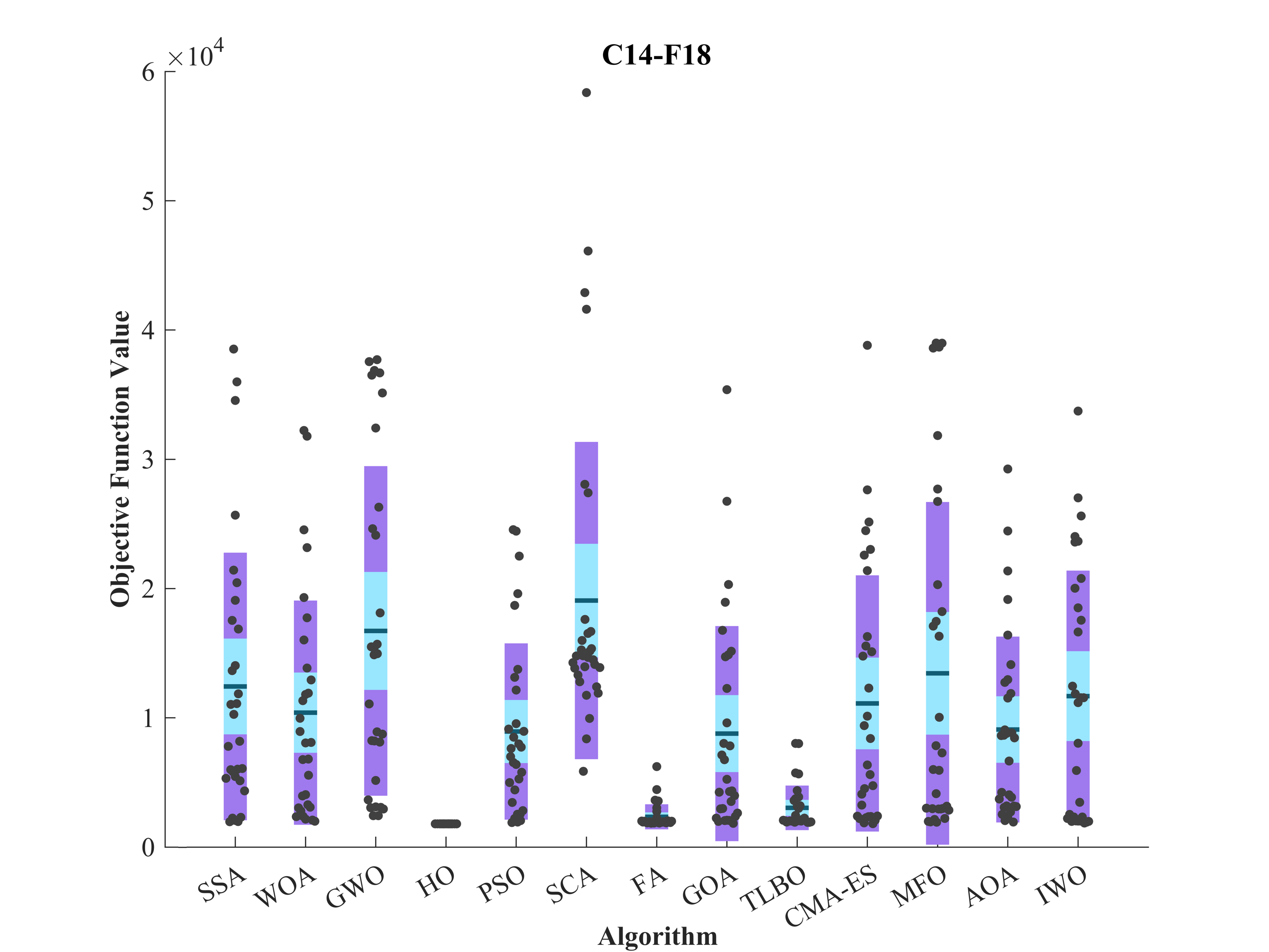 |
| --- | --- |
| 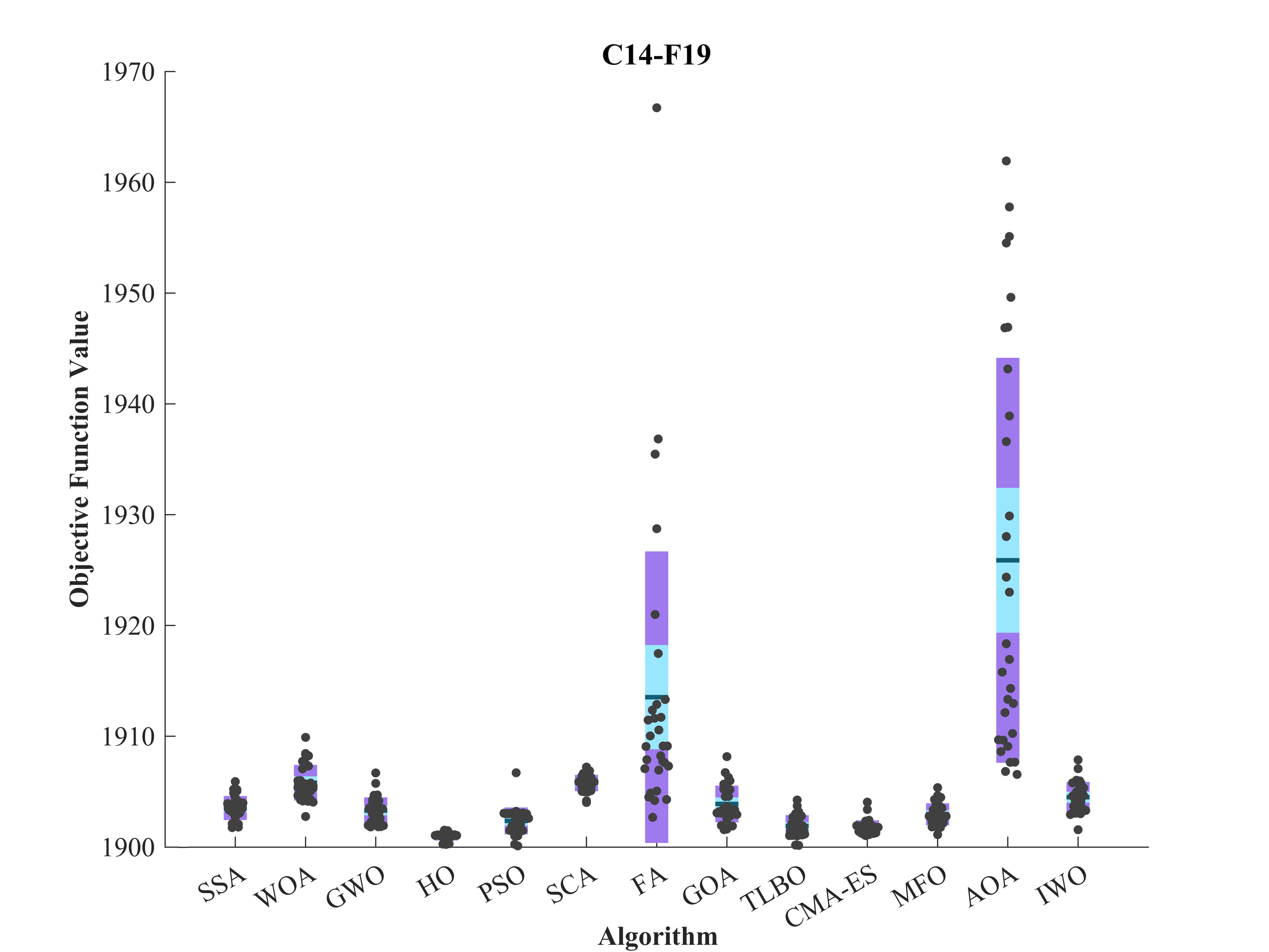 | 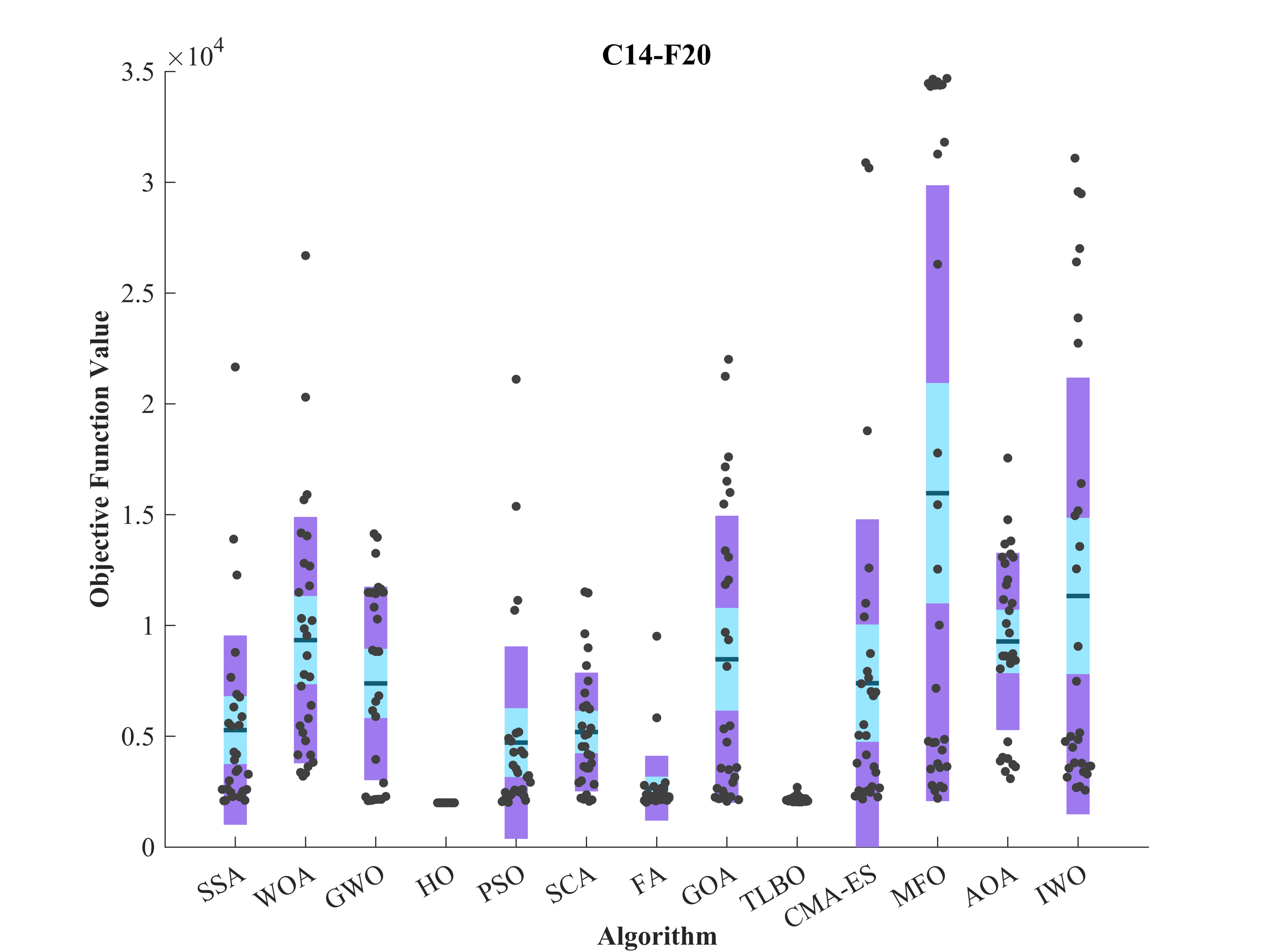 |
| 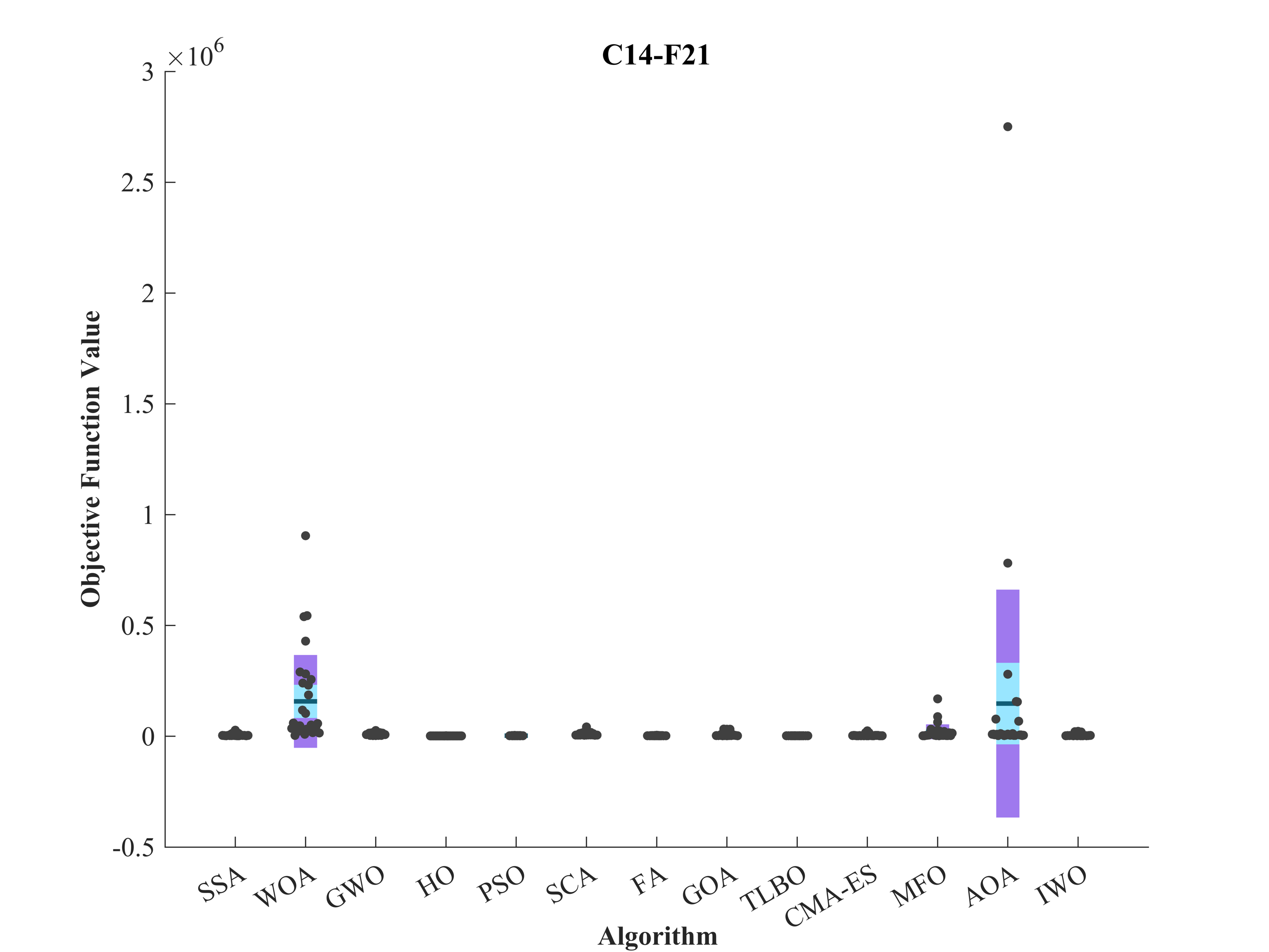 | 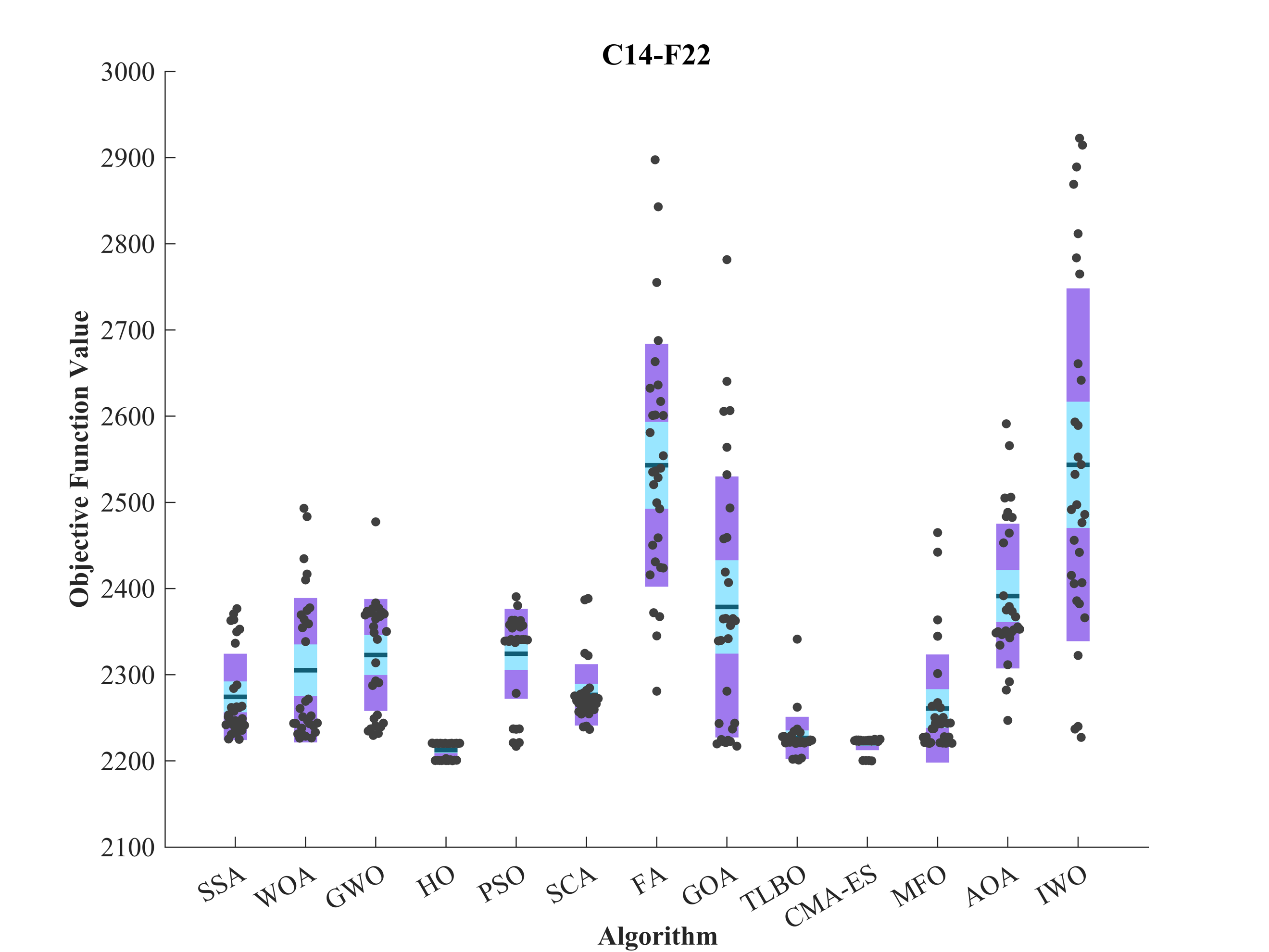 |
| 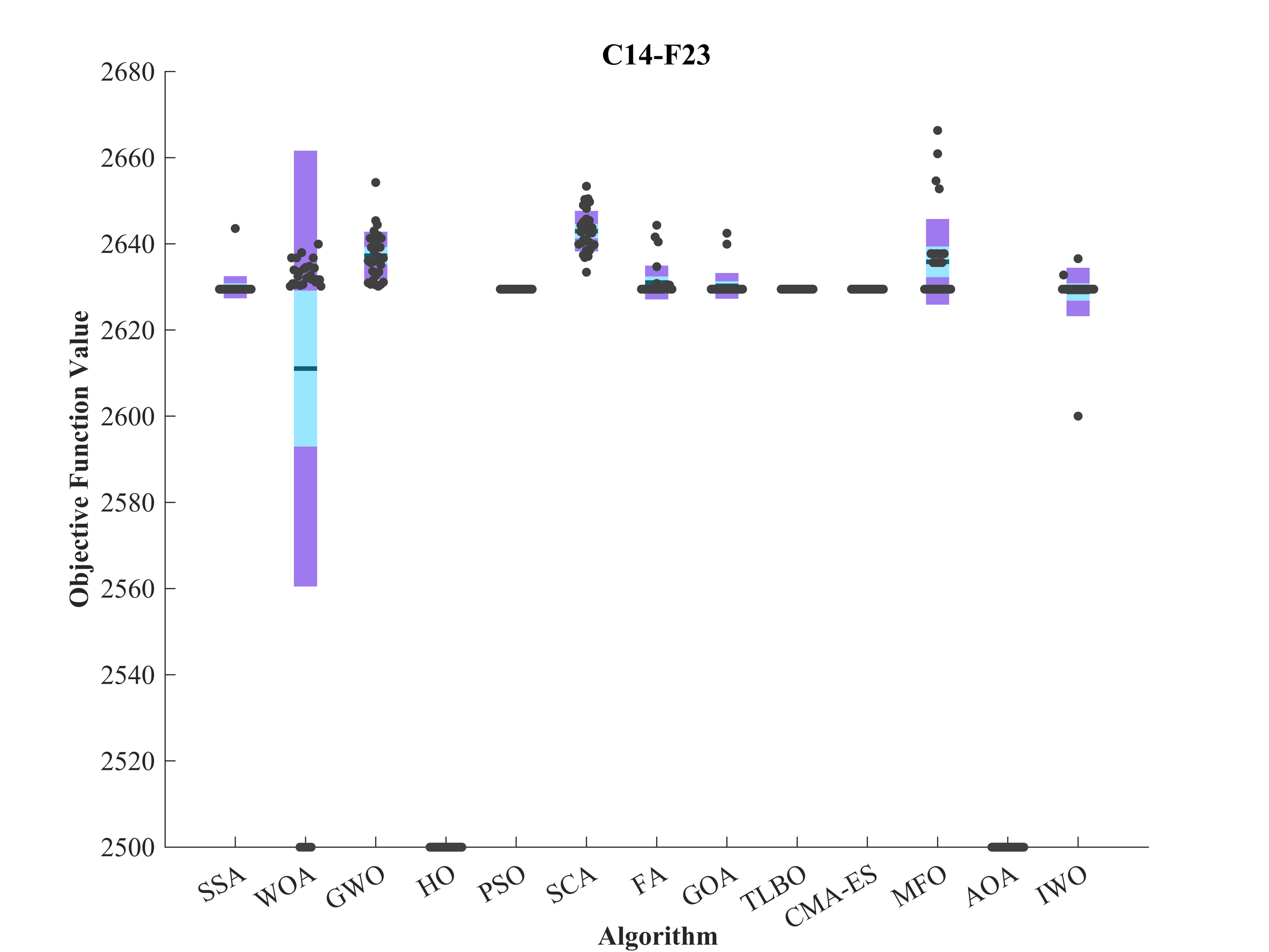 | 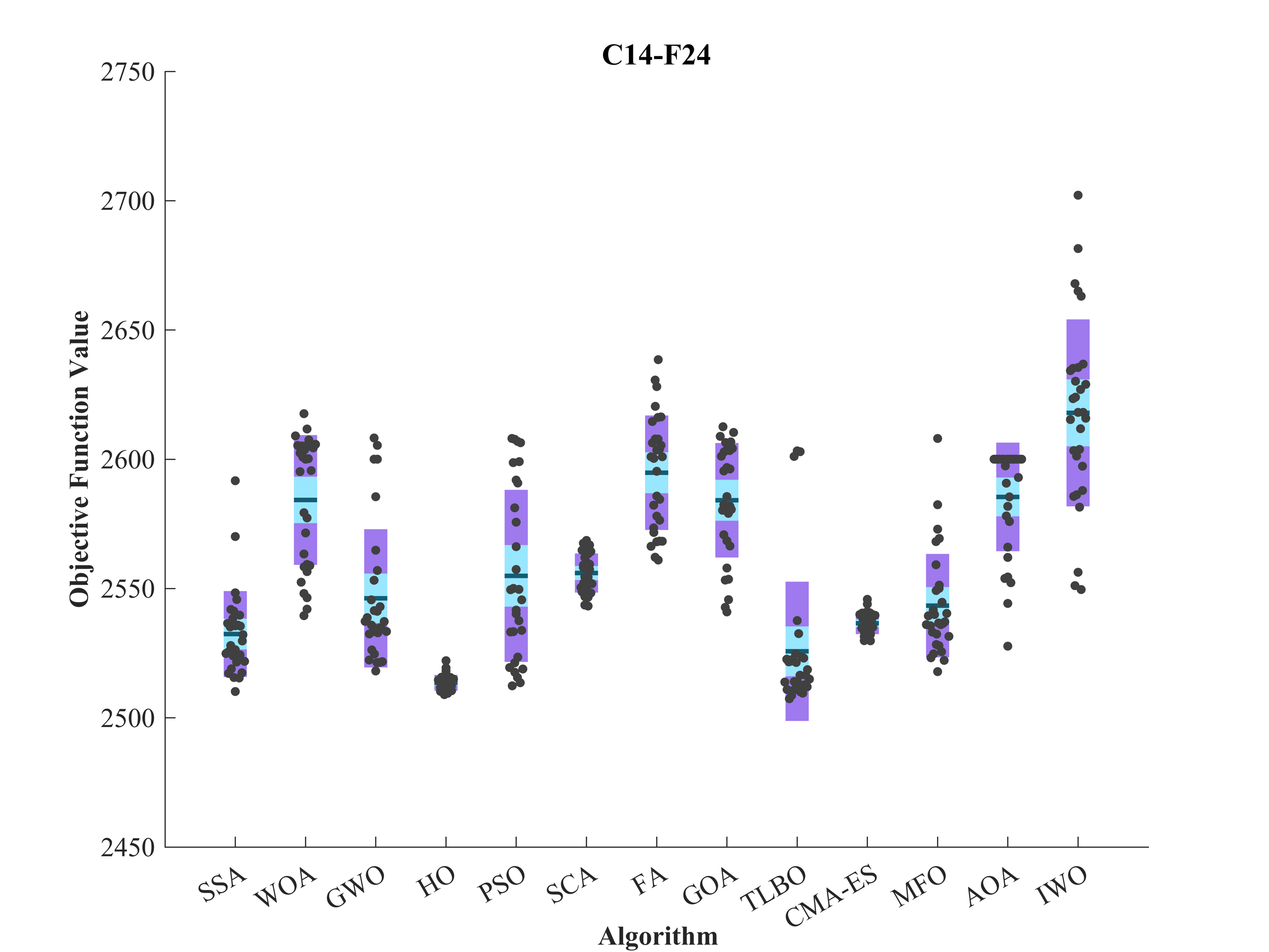 |

**Figure S2.** (continued)

| 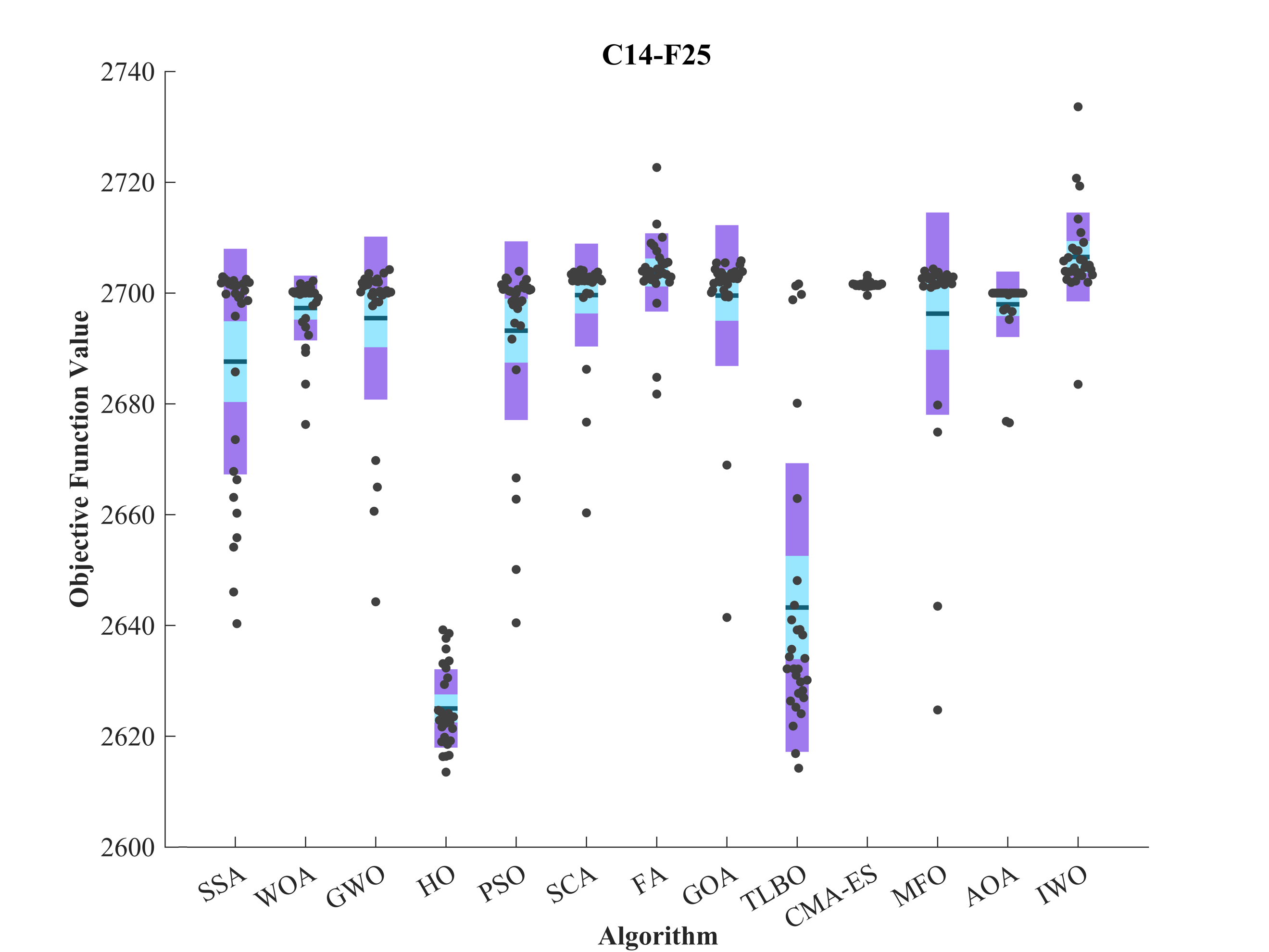 | 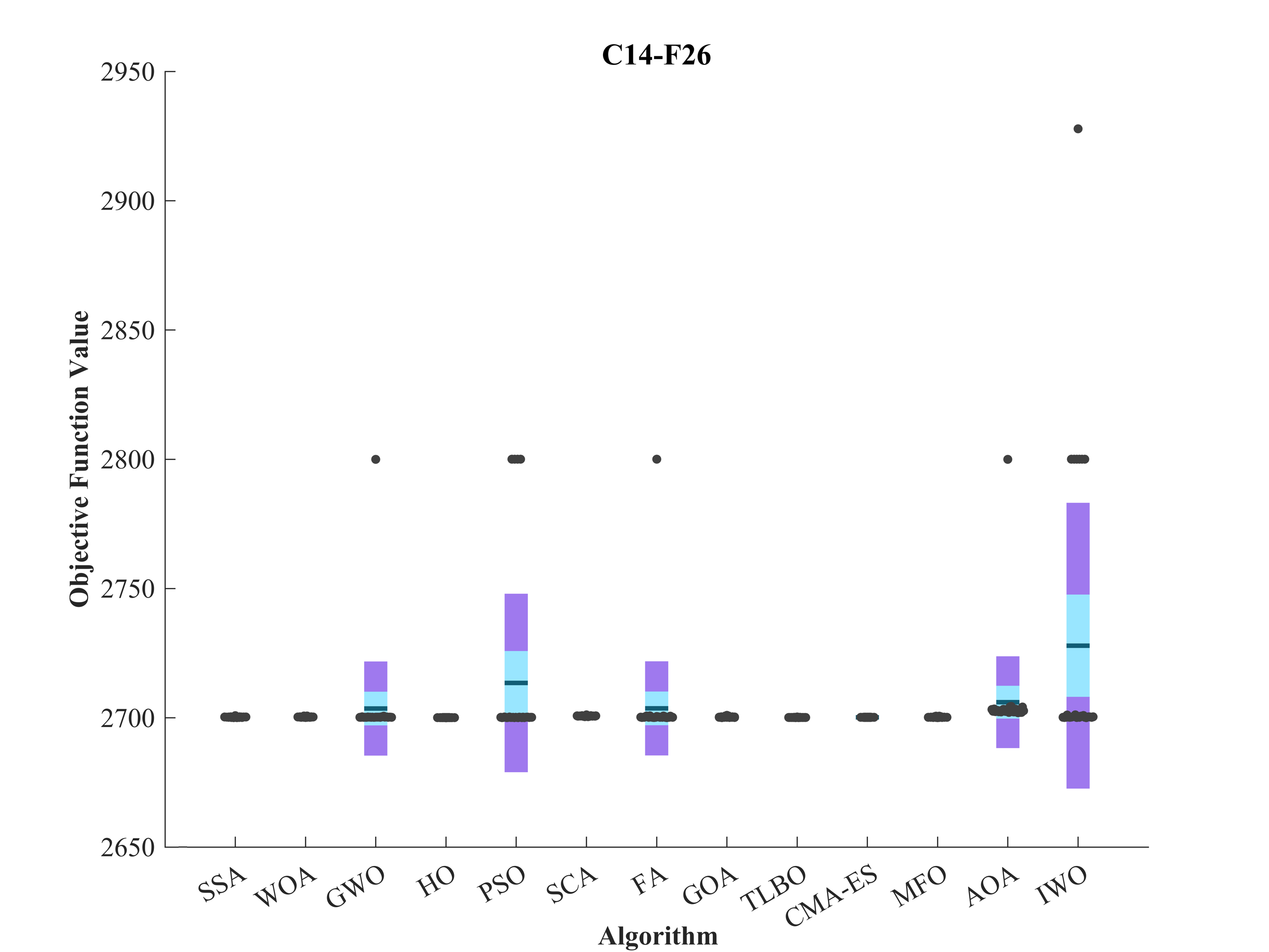 |
| --- | --- |
| 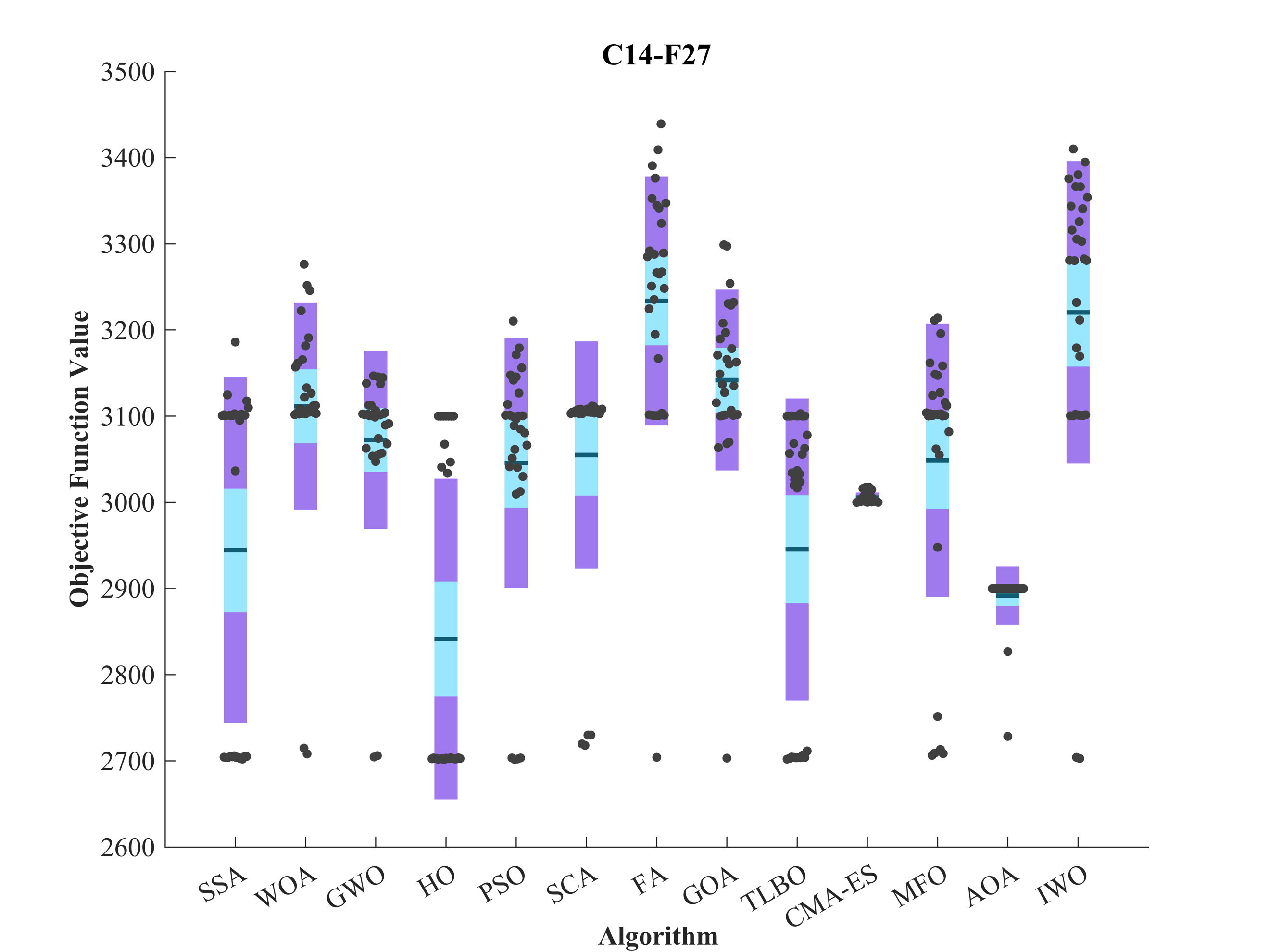 | 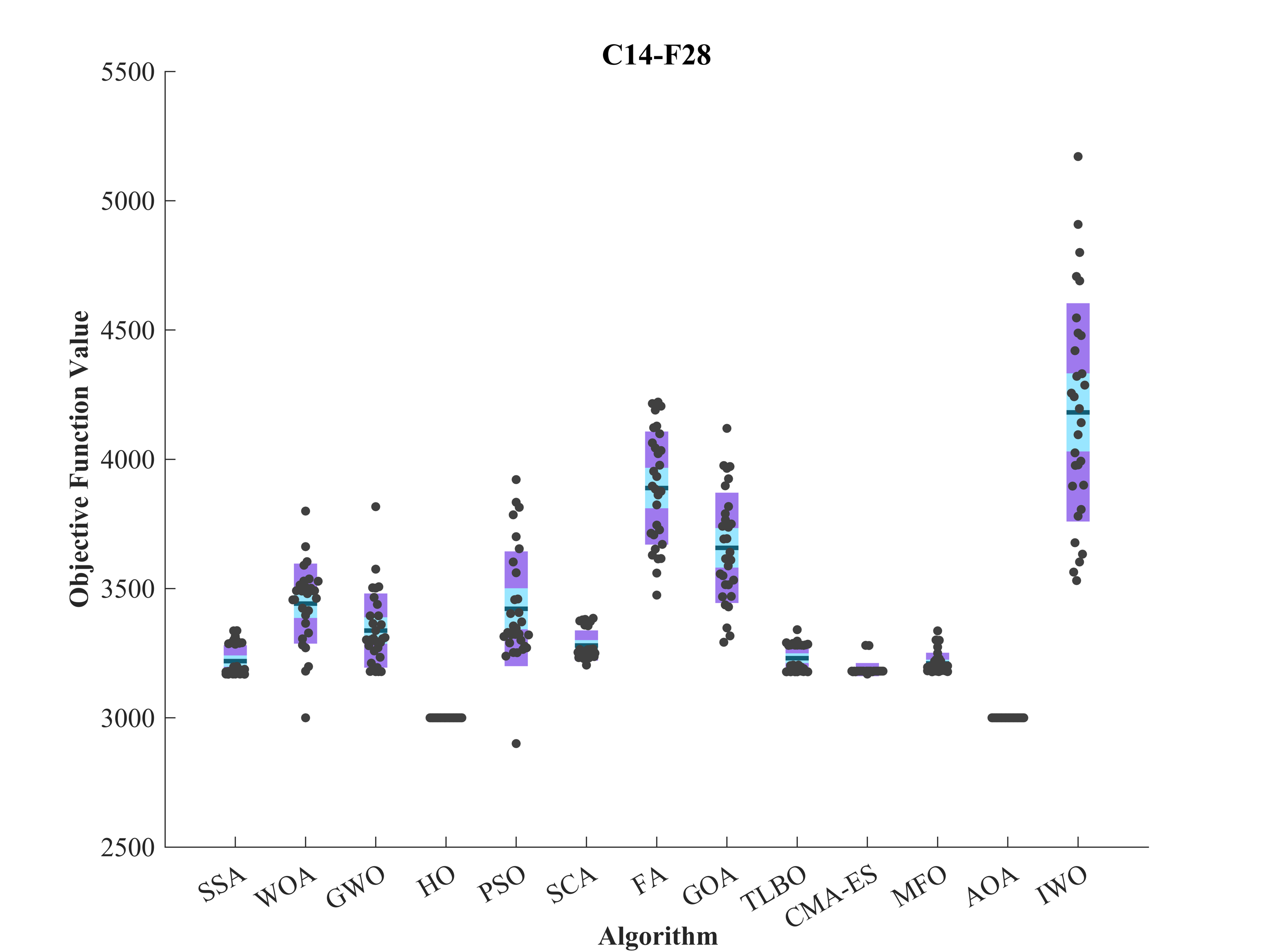 |
| 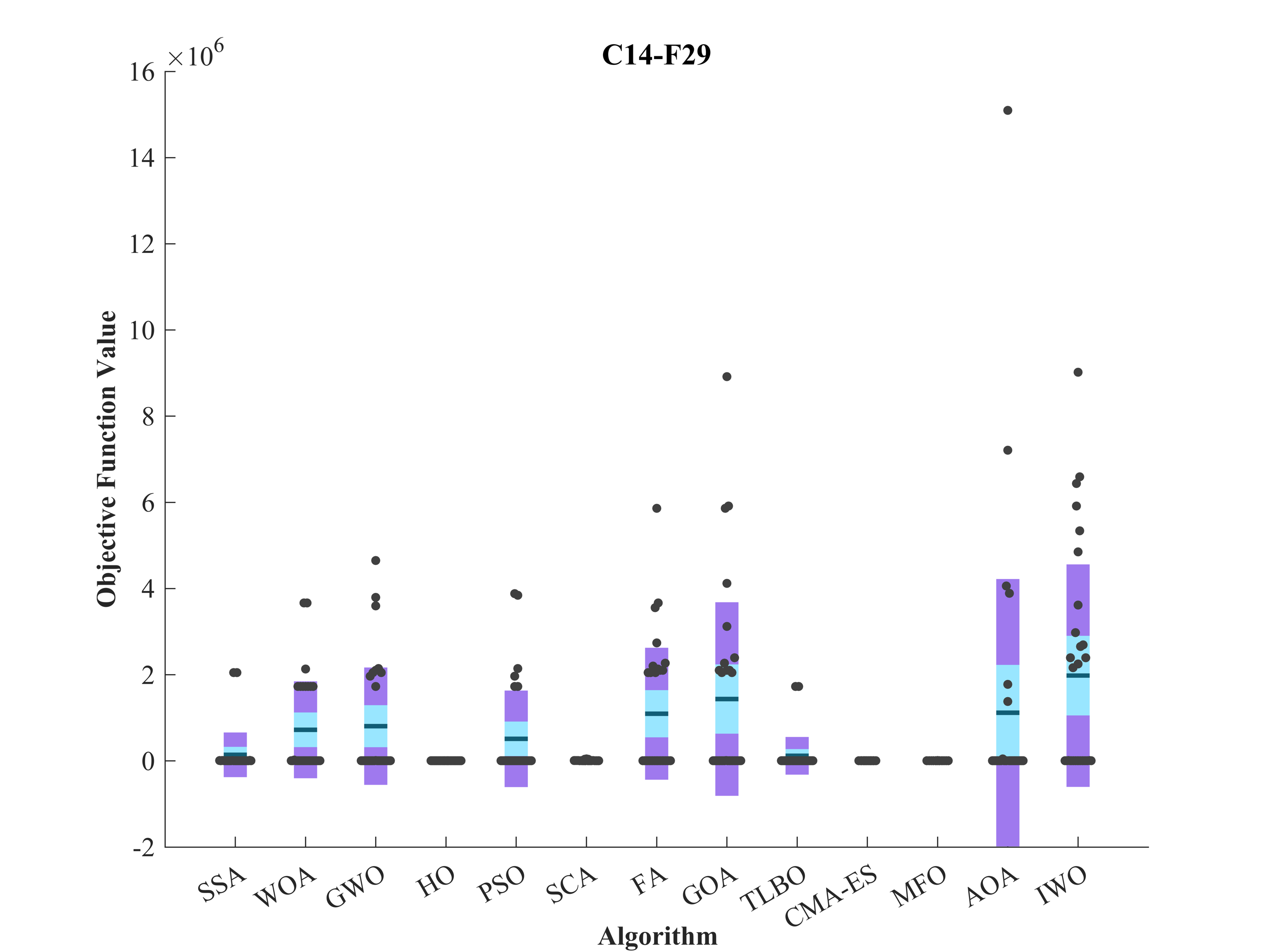 | 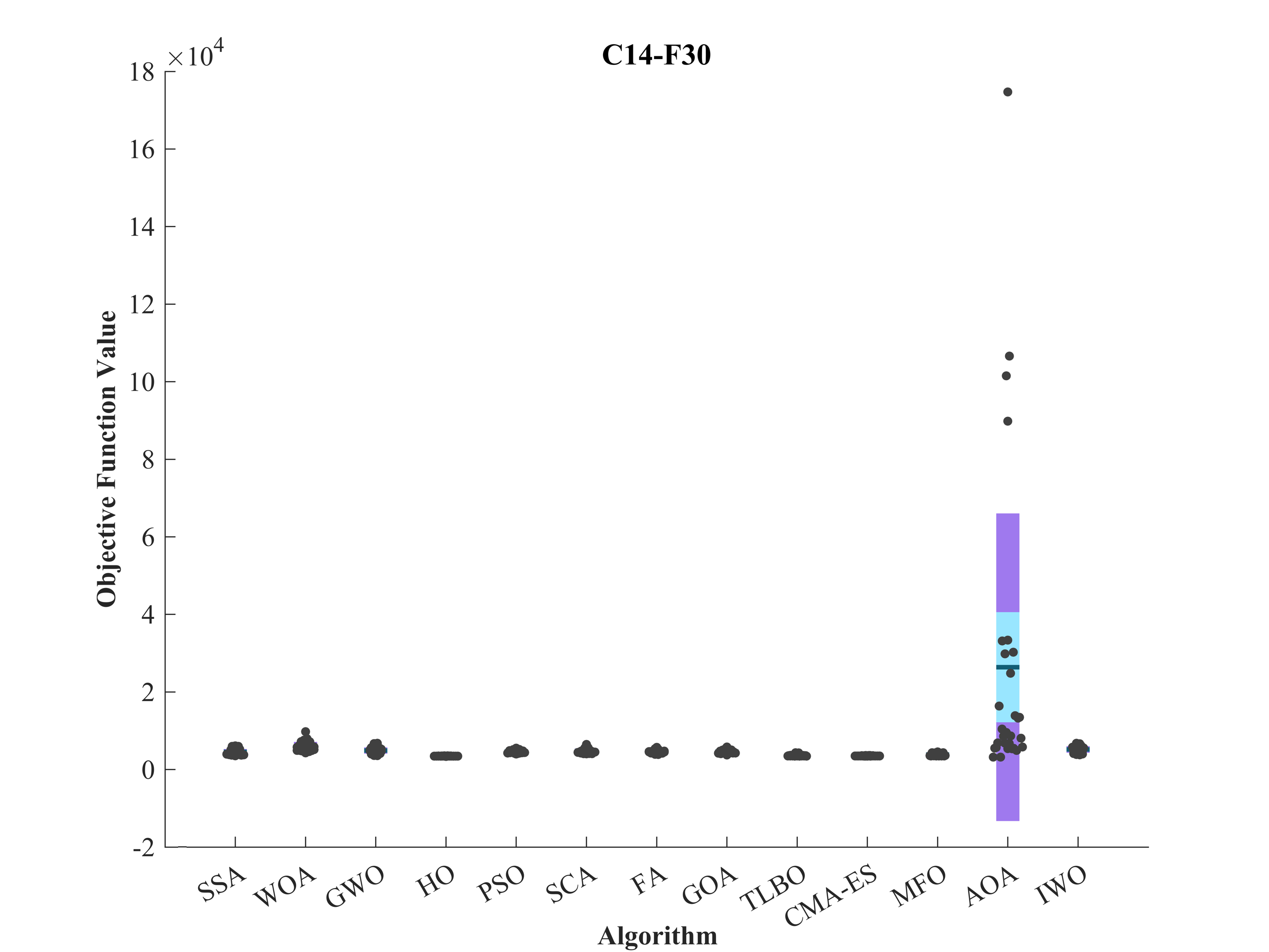 |

**Figure S2.** (continued)

| 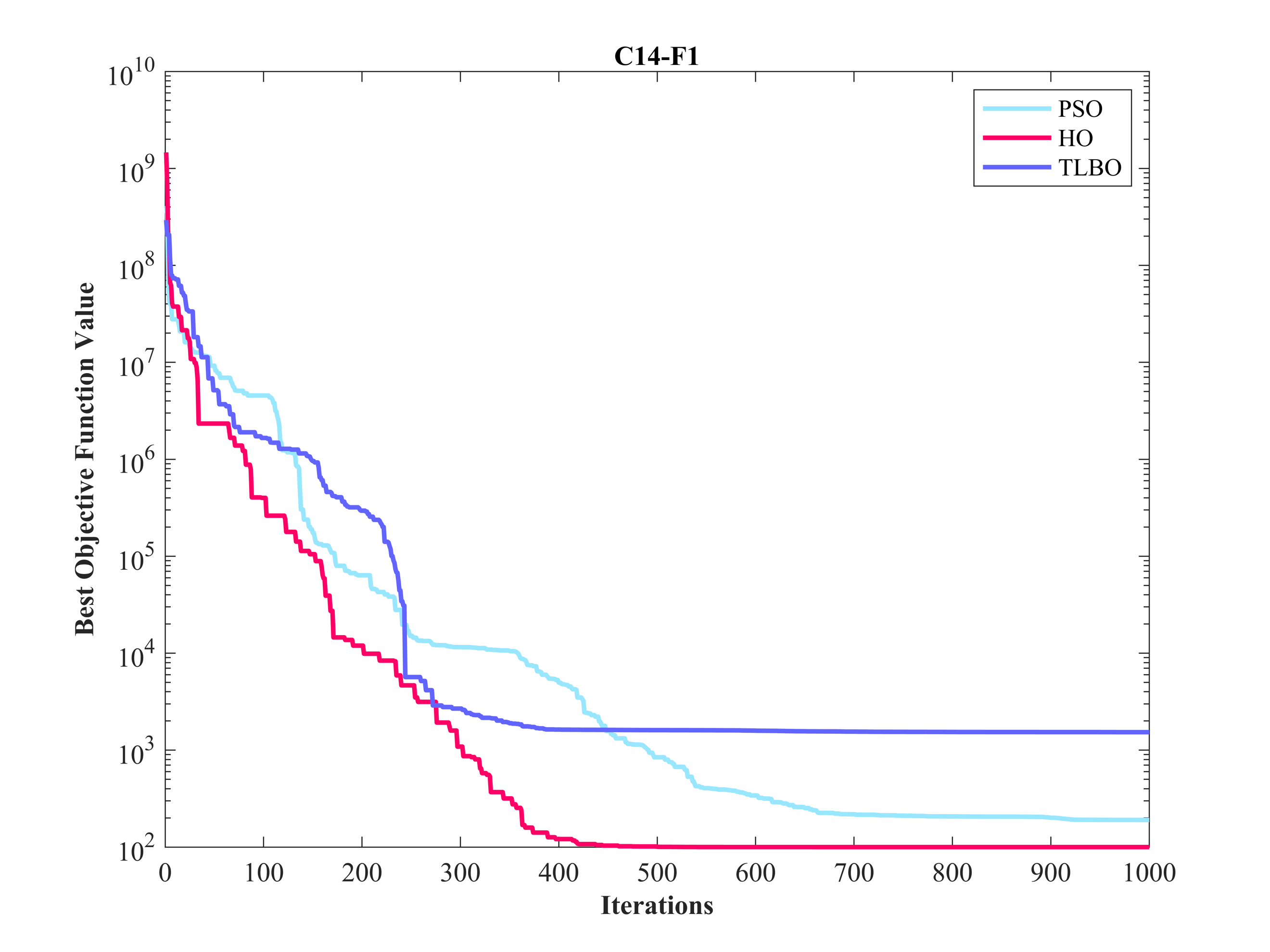 | 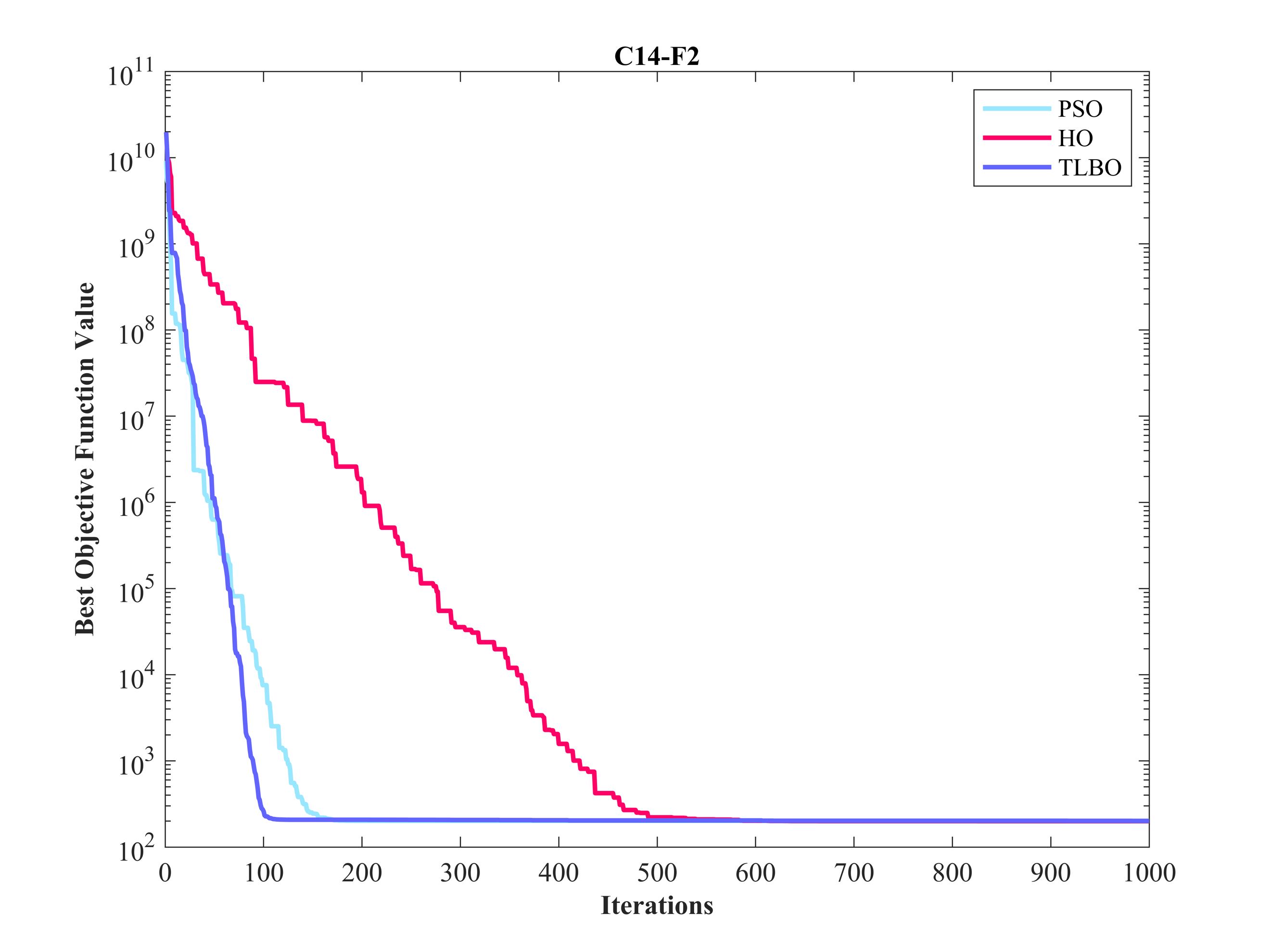 |
| --- | --- |
| 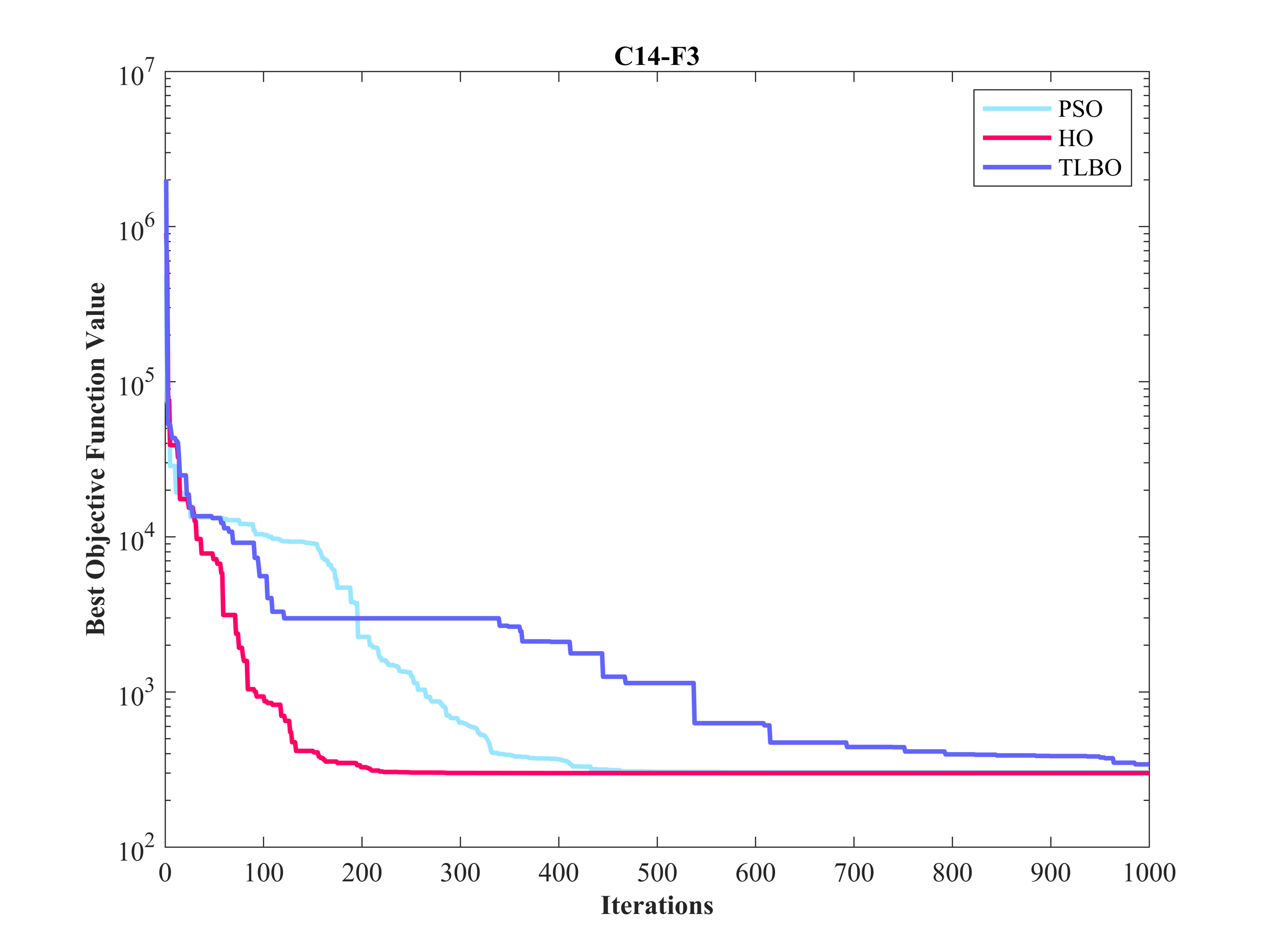 | 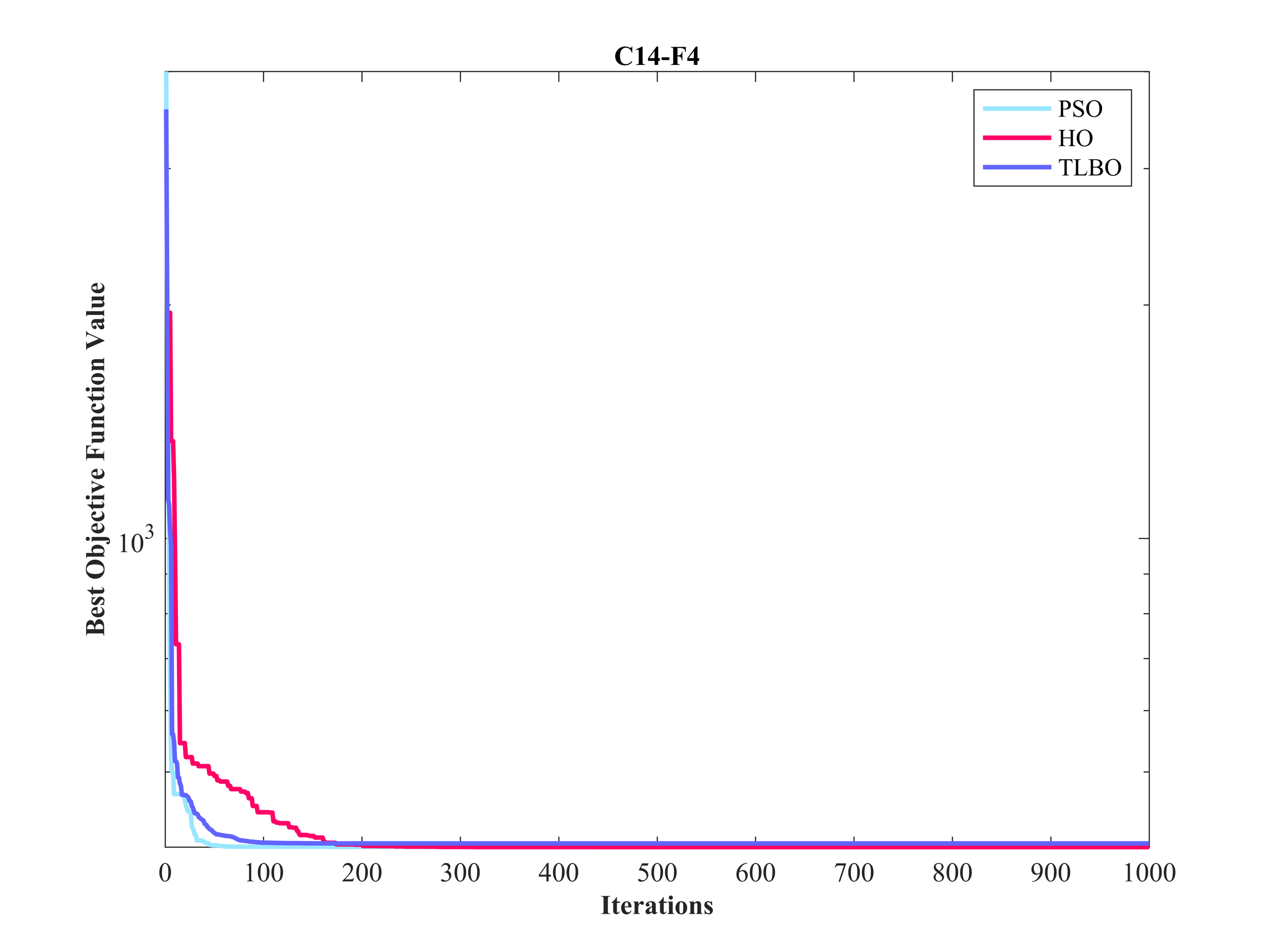 |
| 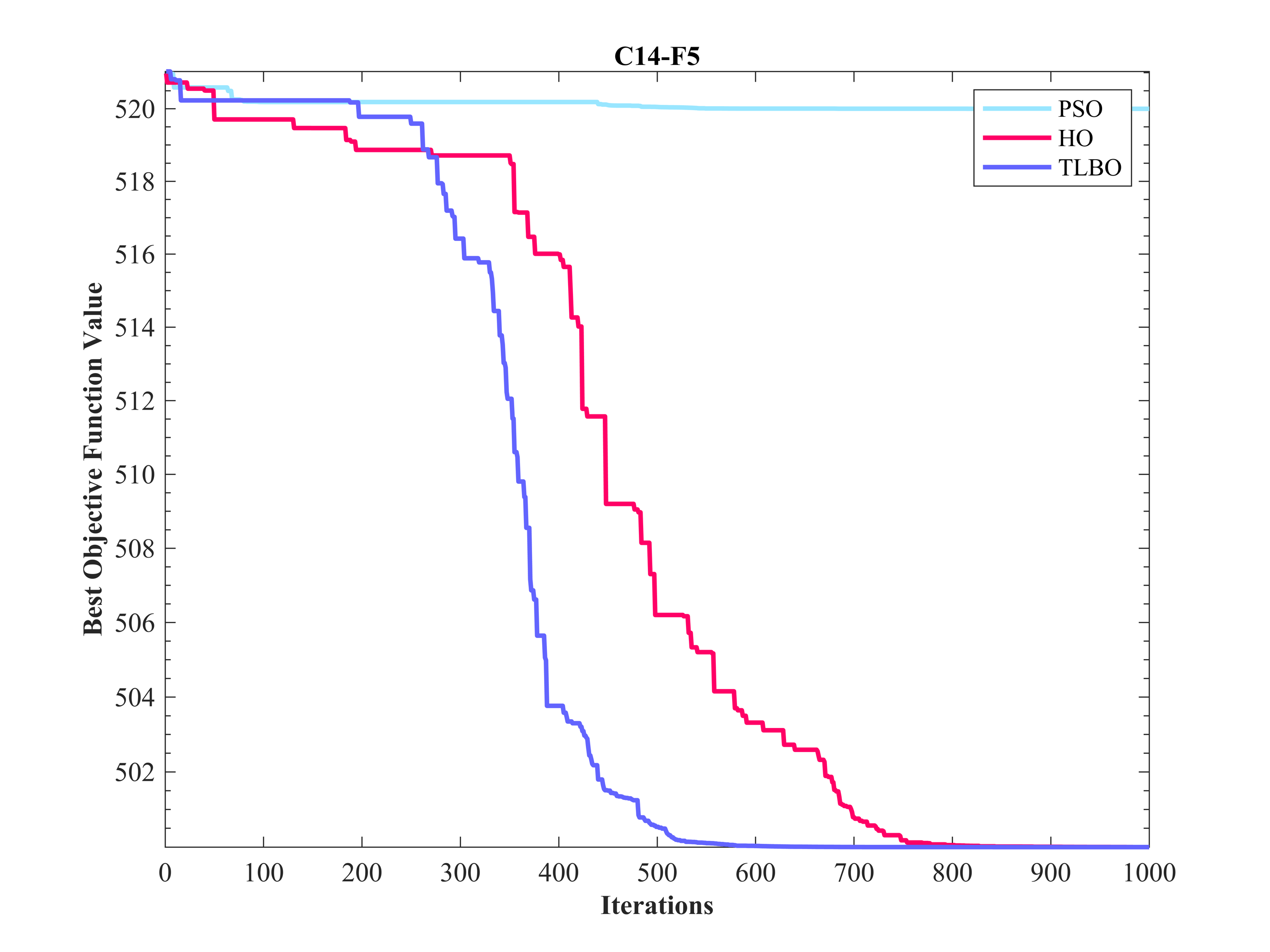 | 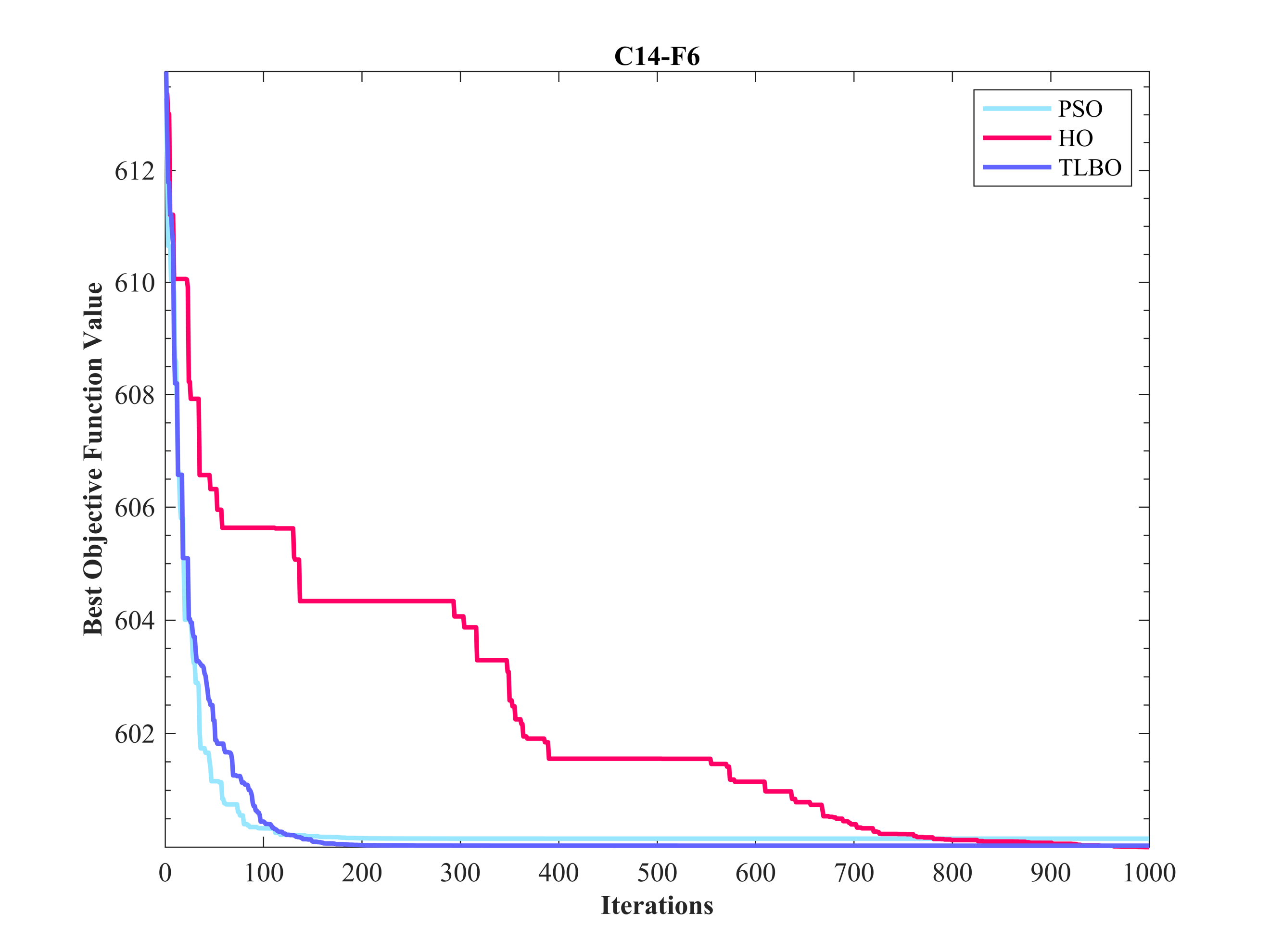 |
| 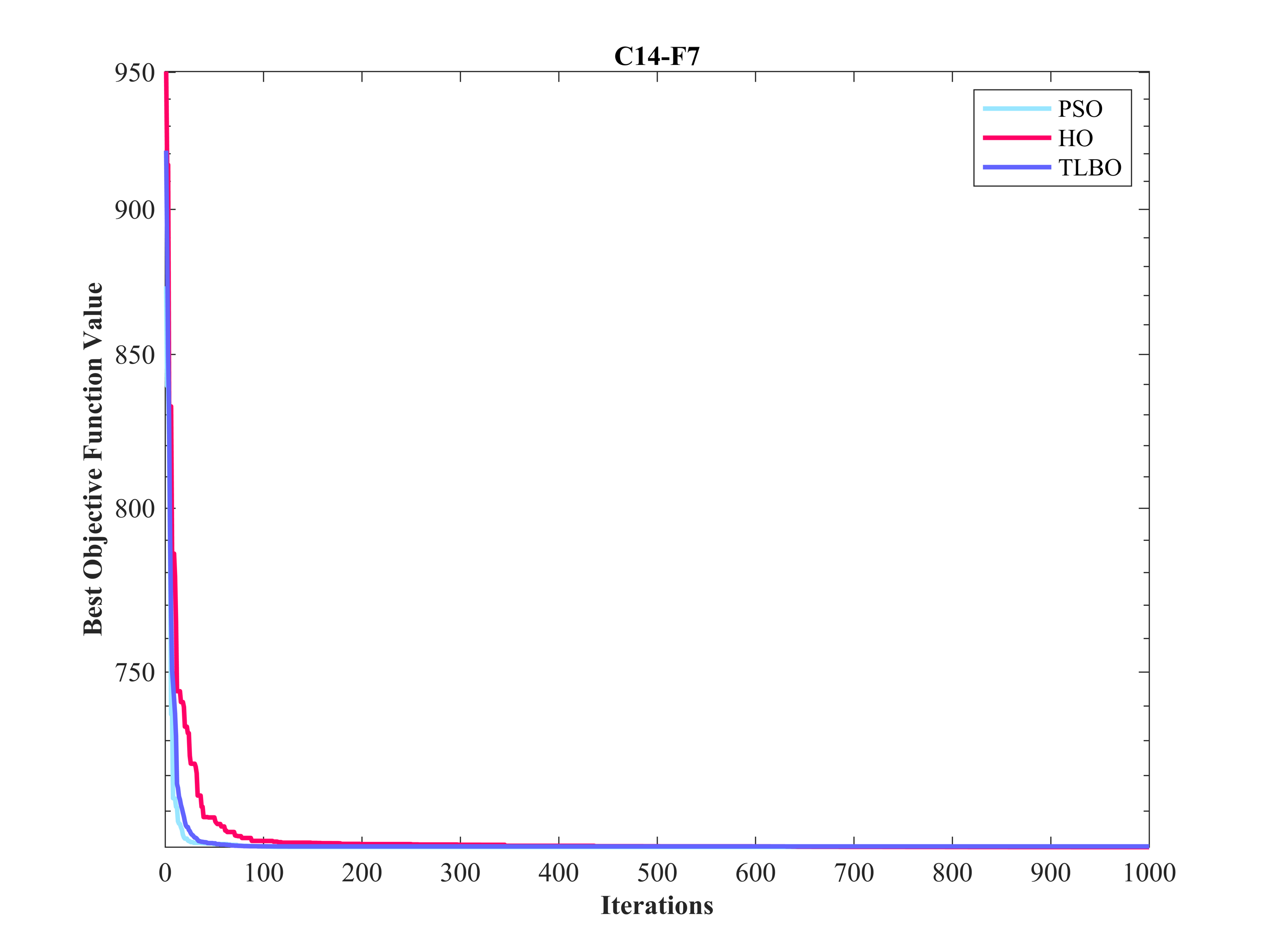 | 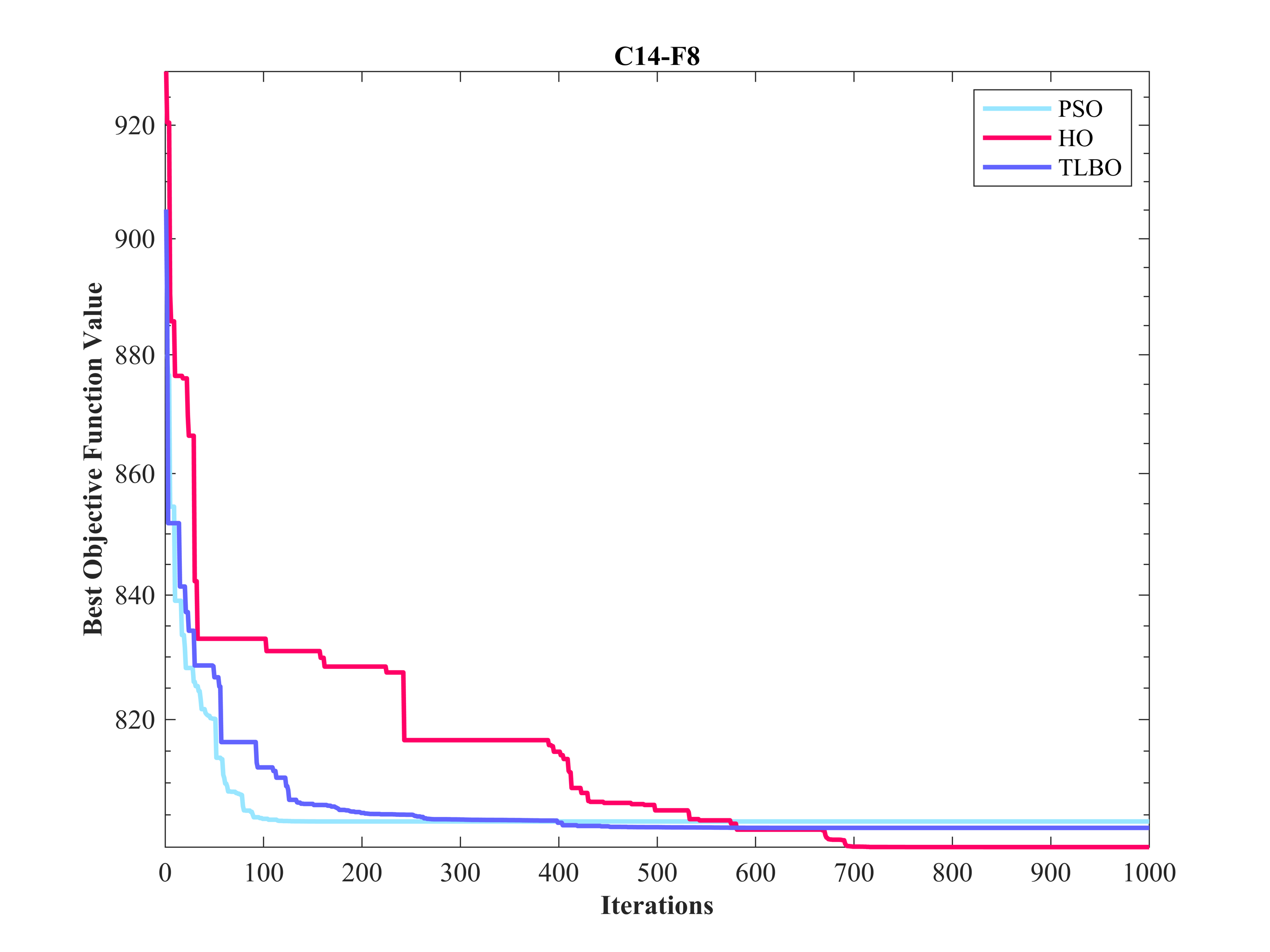 |

**Figure S3.** Convergence curves of the top three algorithms in each function in CEC 2014 (D = 10).

| 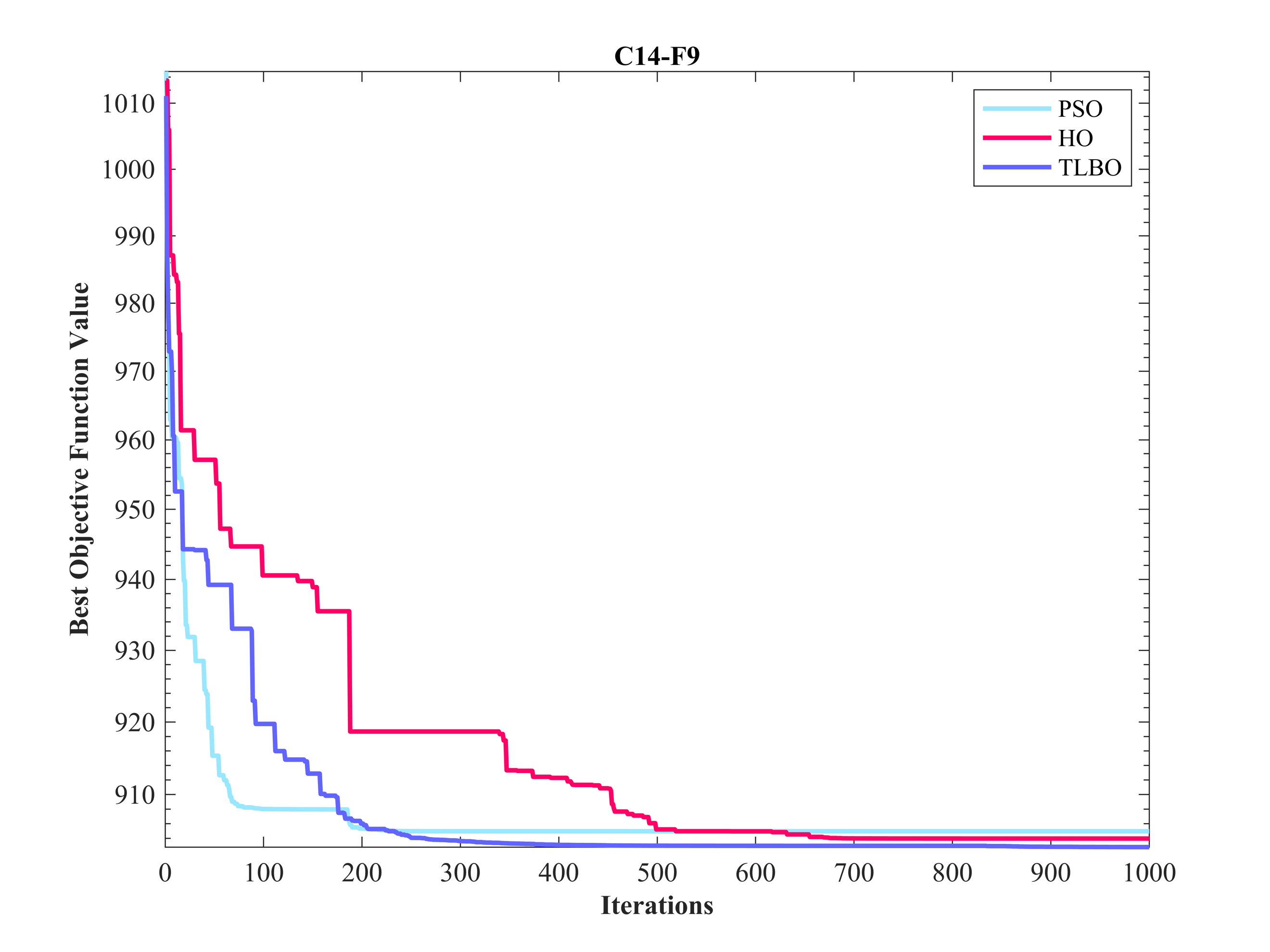 | 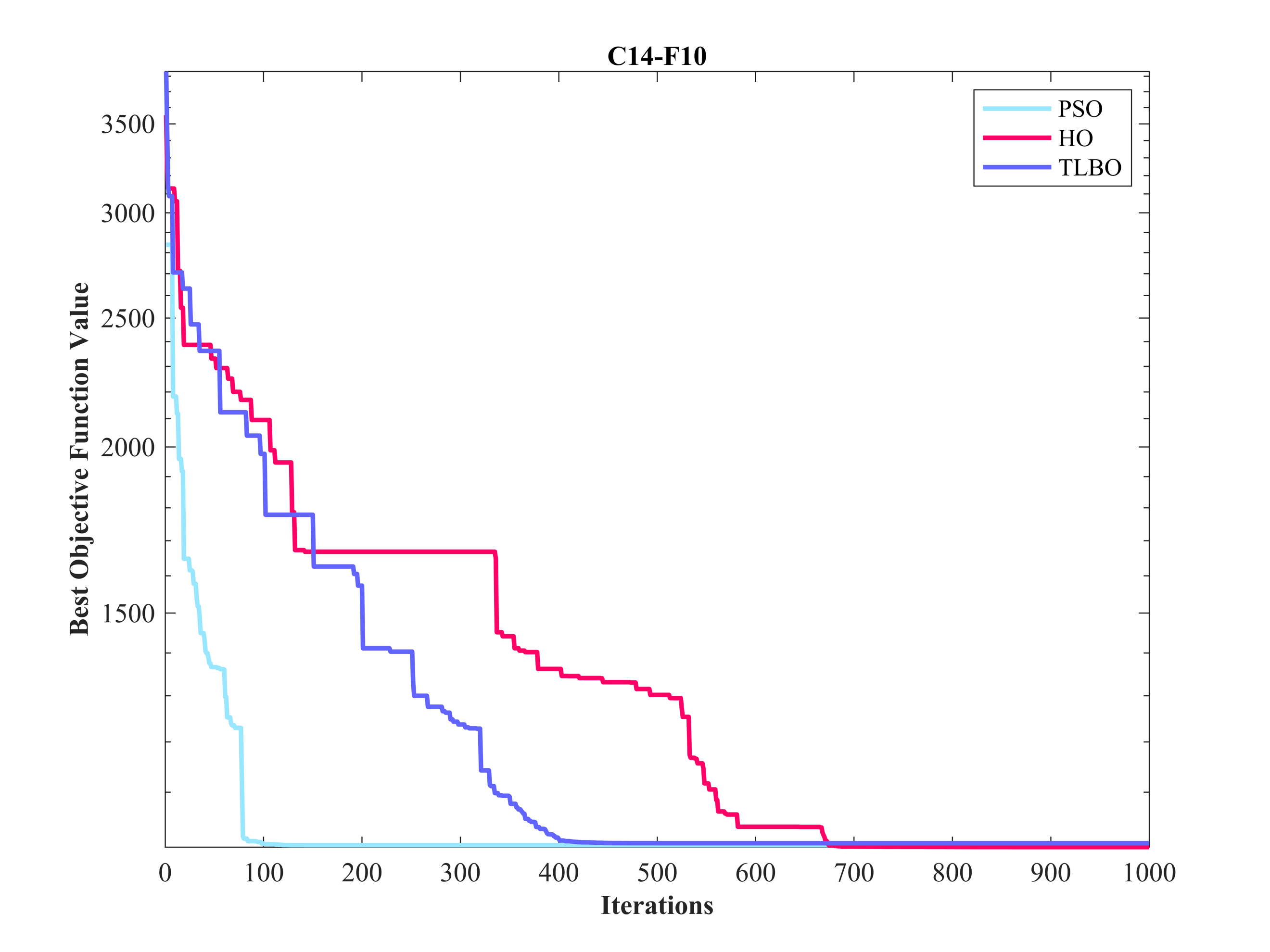 |
| --- | --- |
| 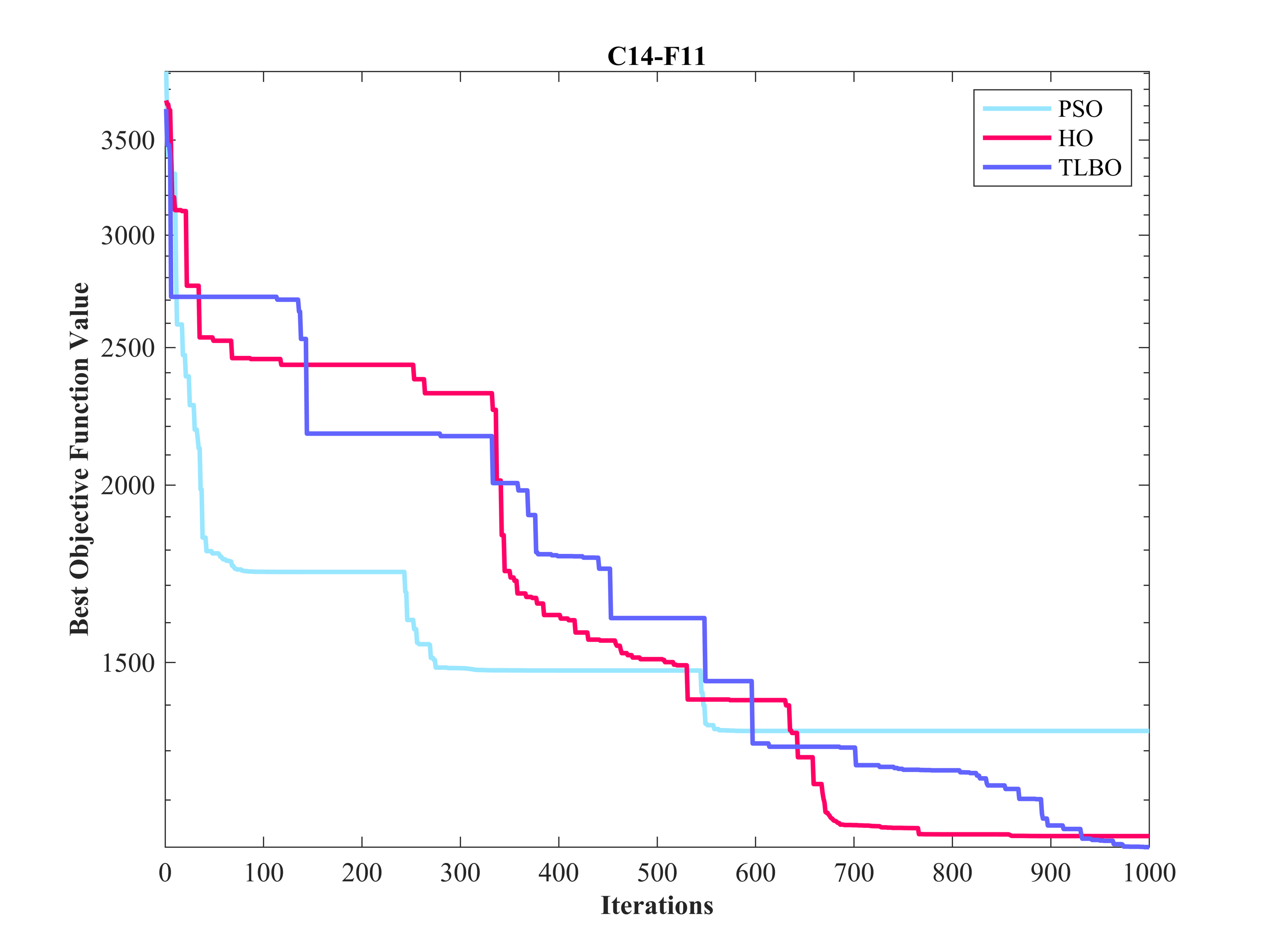 | 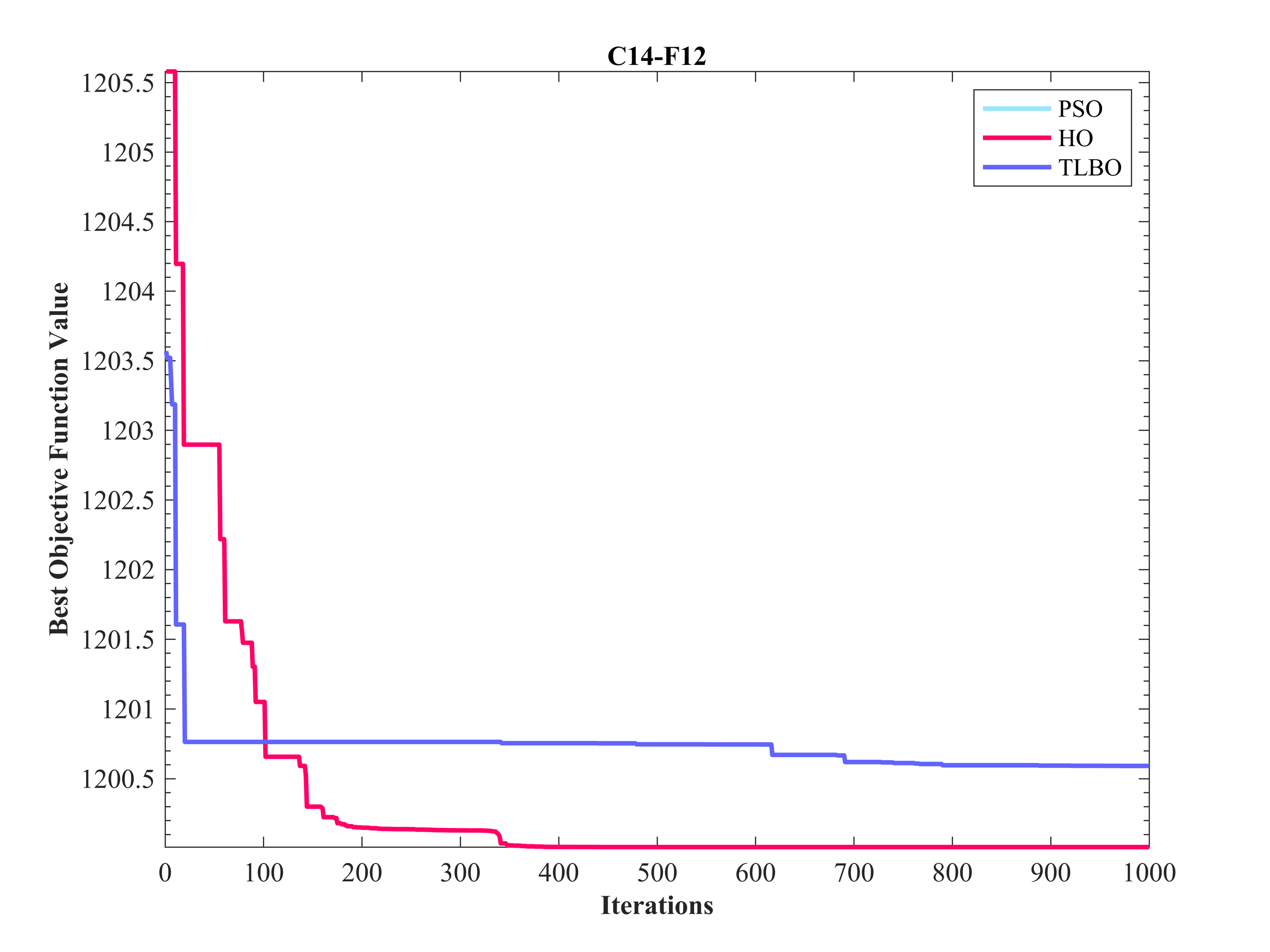 |
| 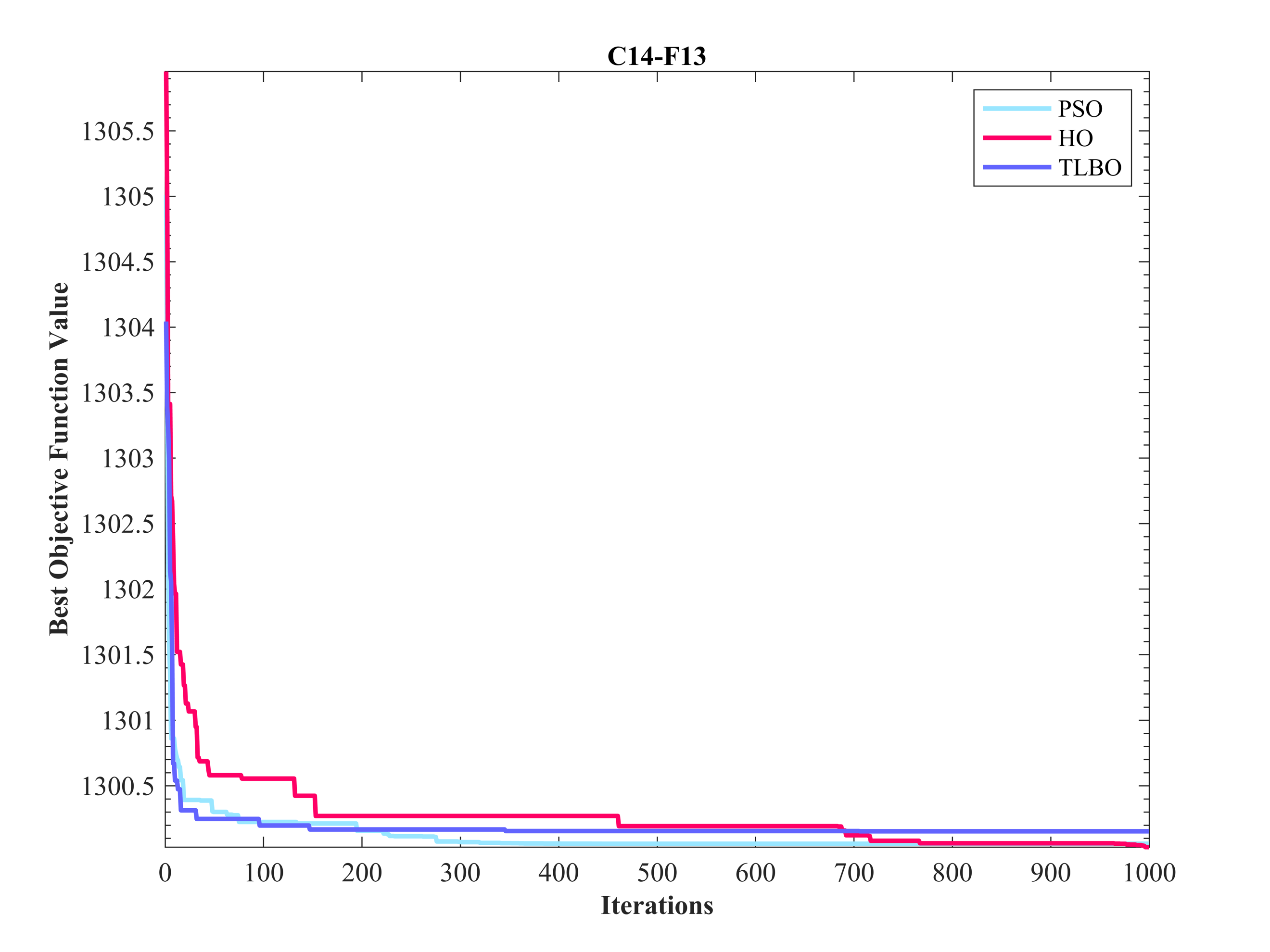 | 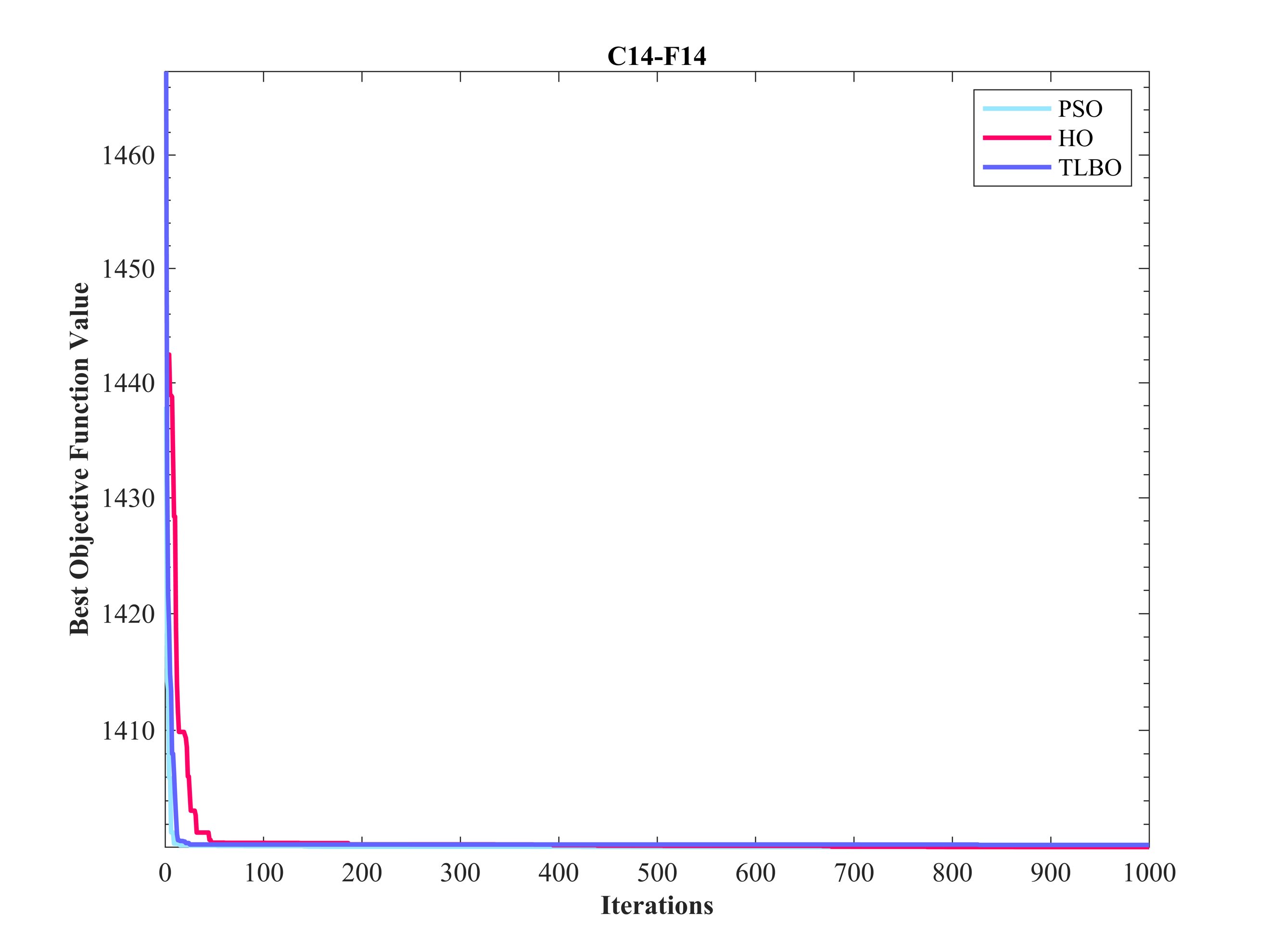 |
| 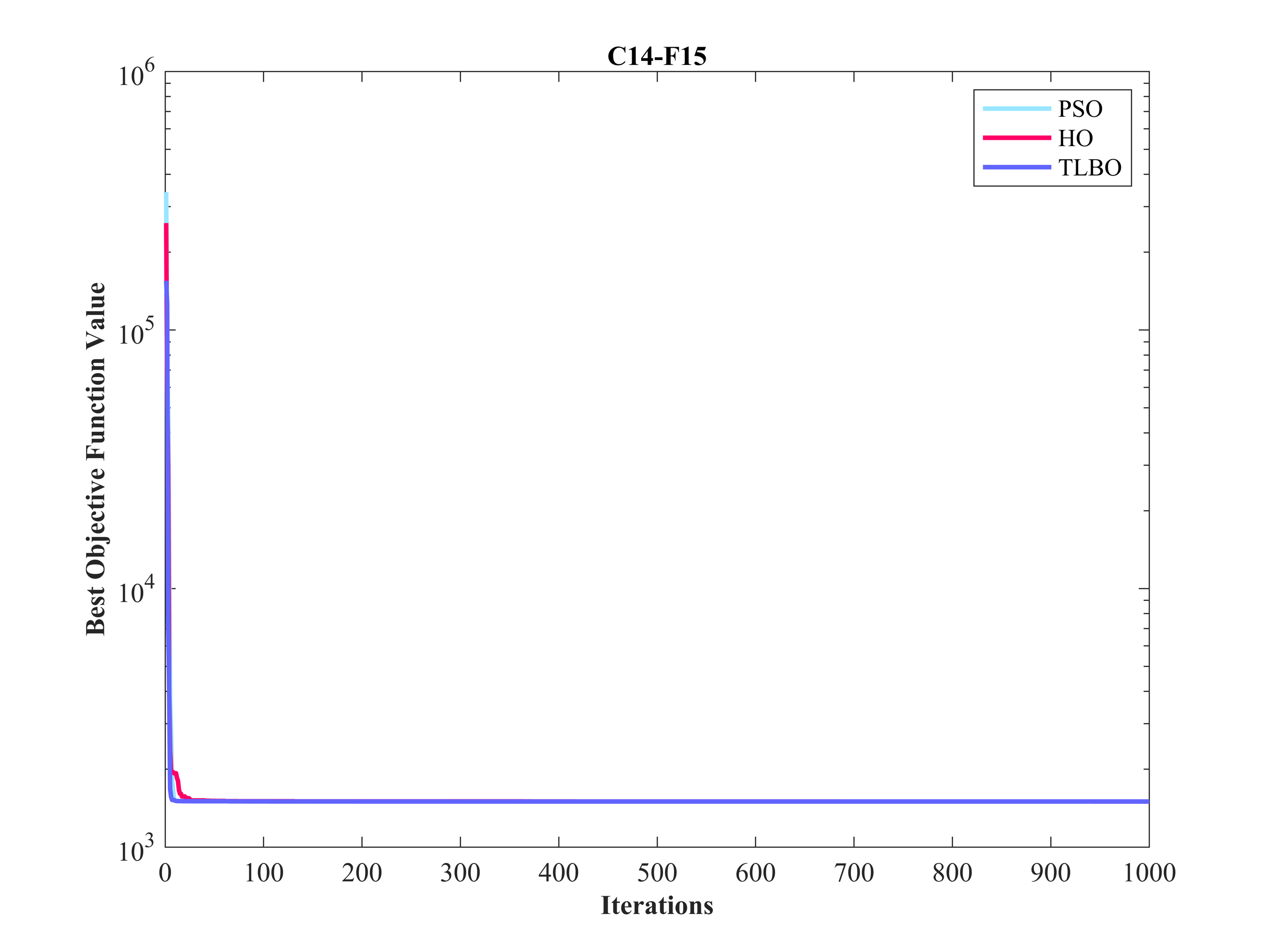 | 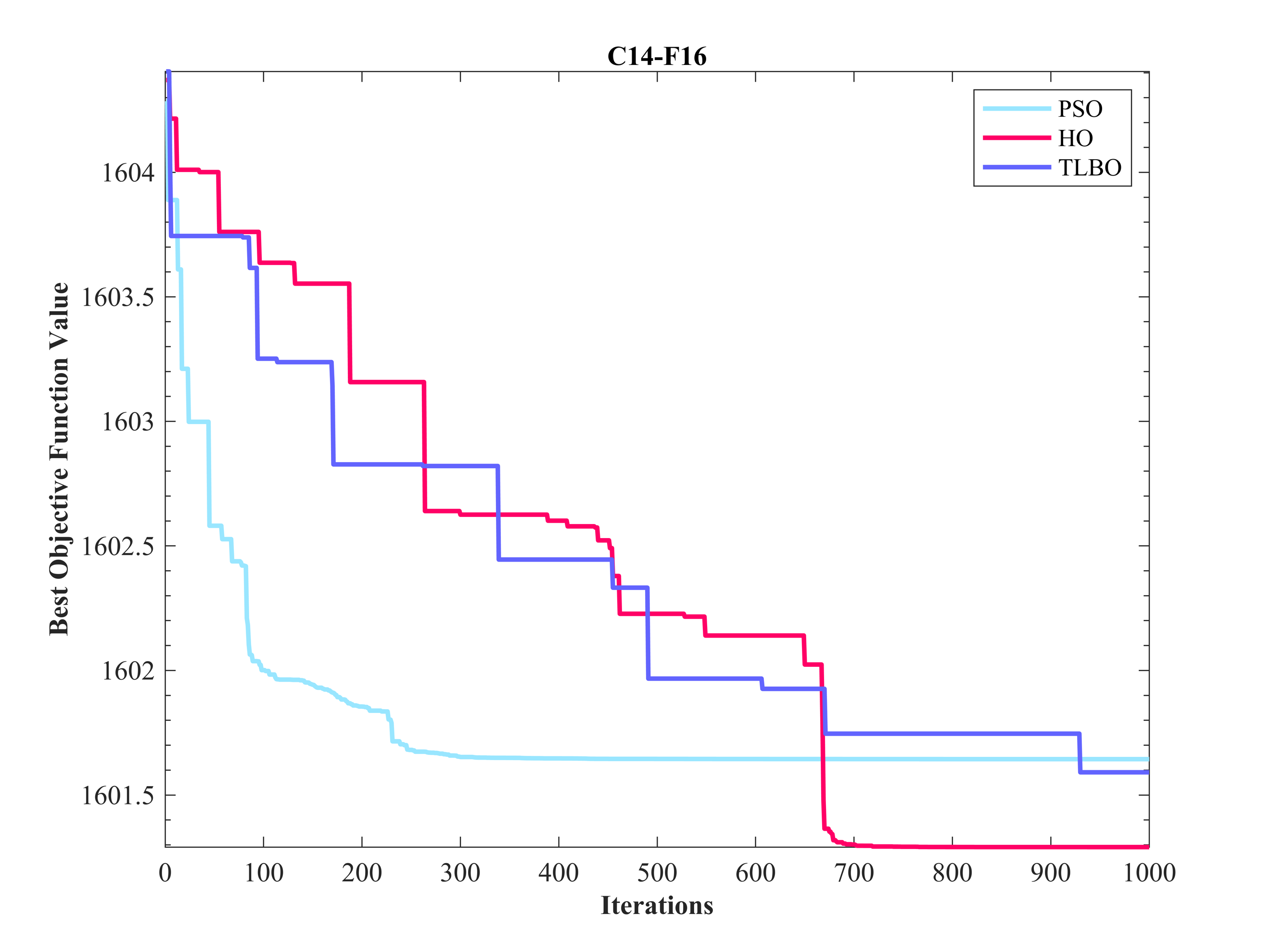 |

**Figure S3.** (continued)

| 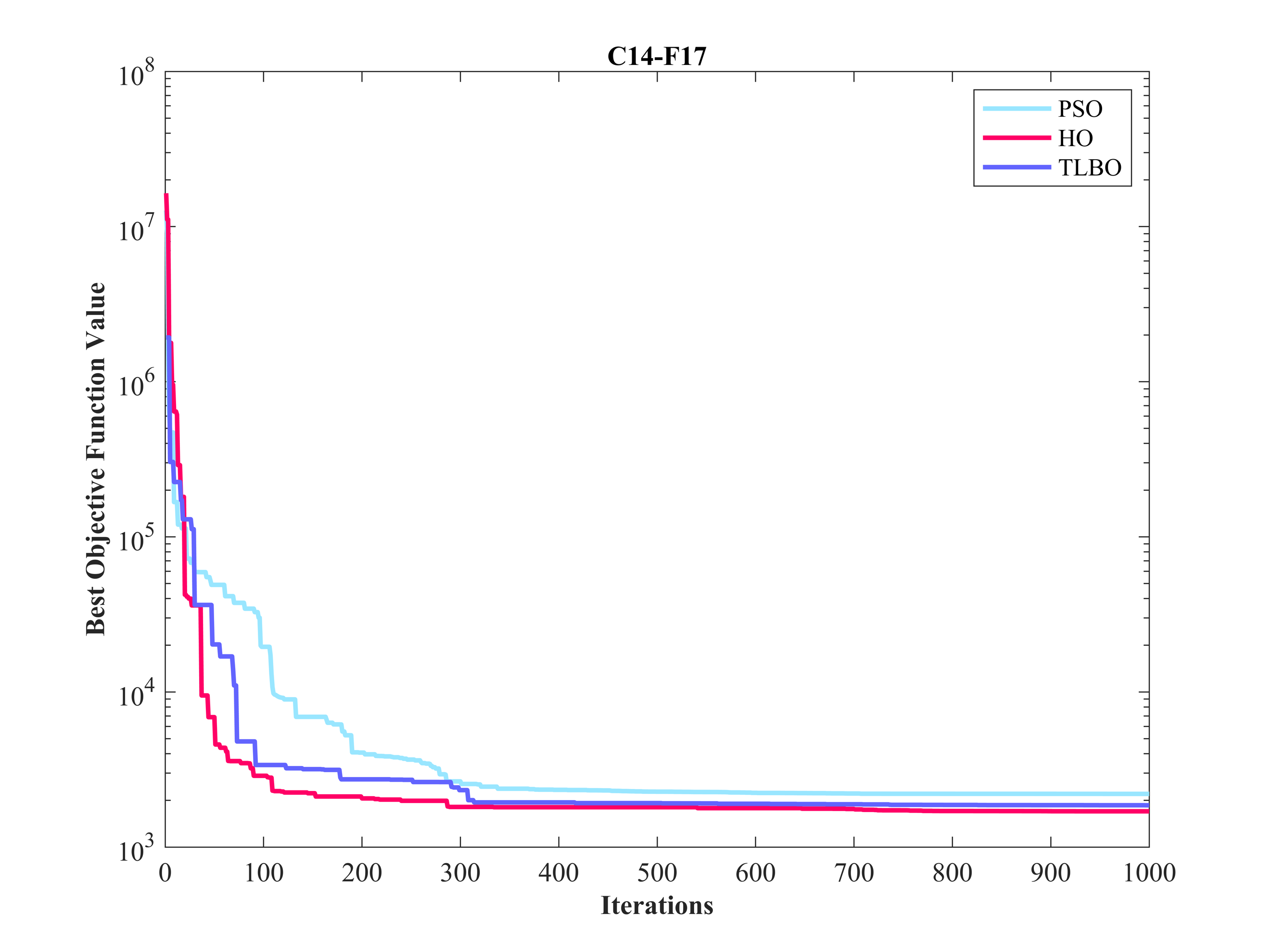 | 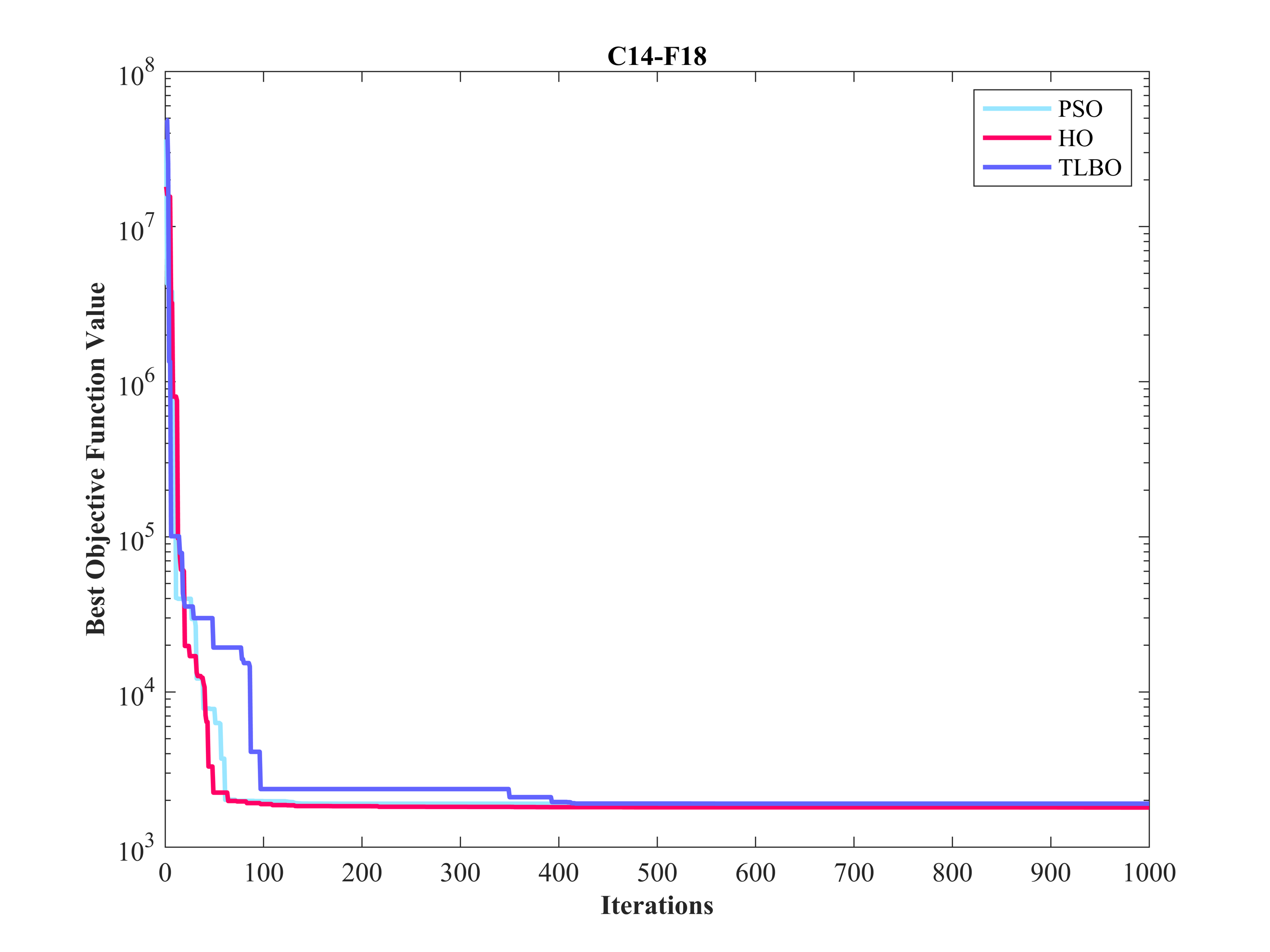 |
| --- | --- |
| 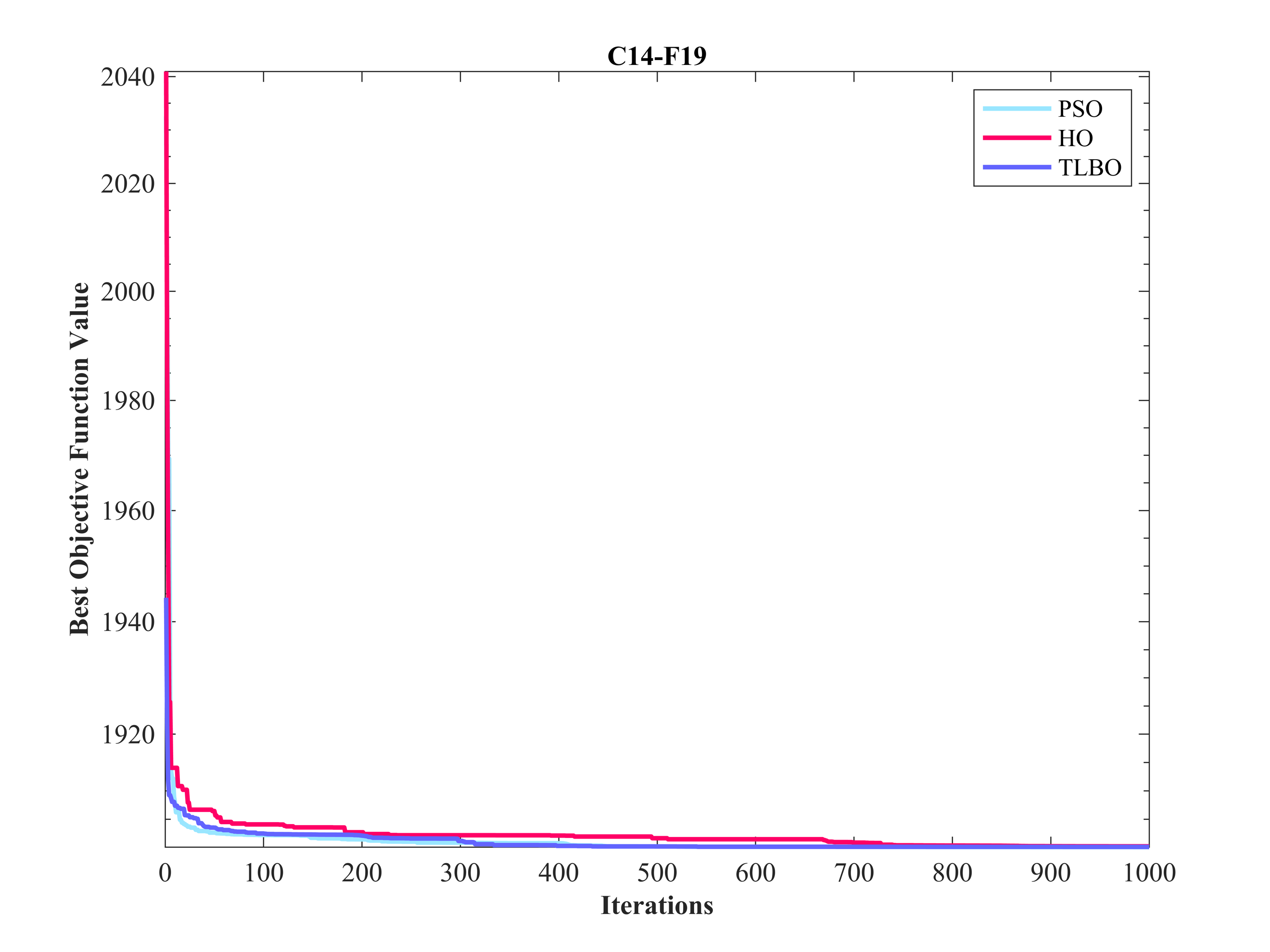 | 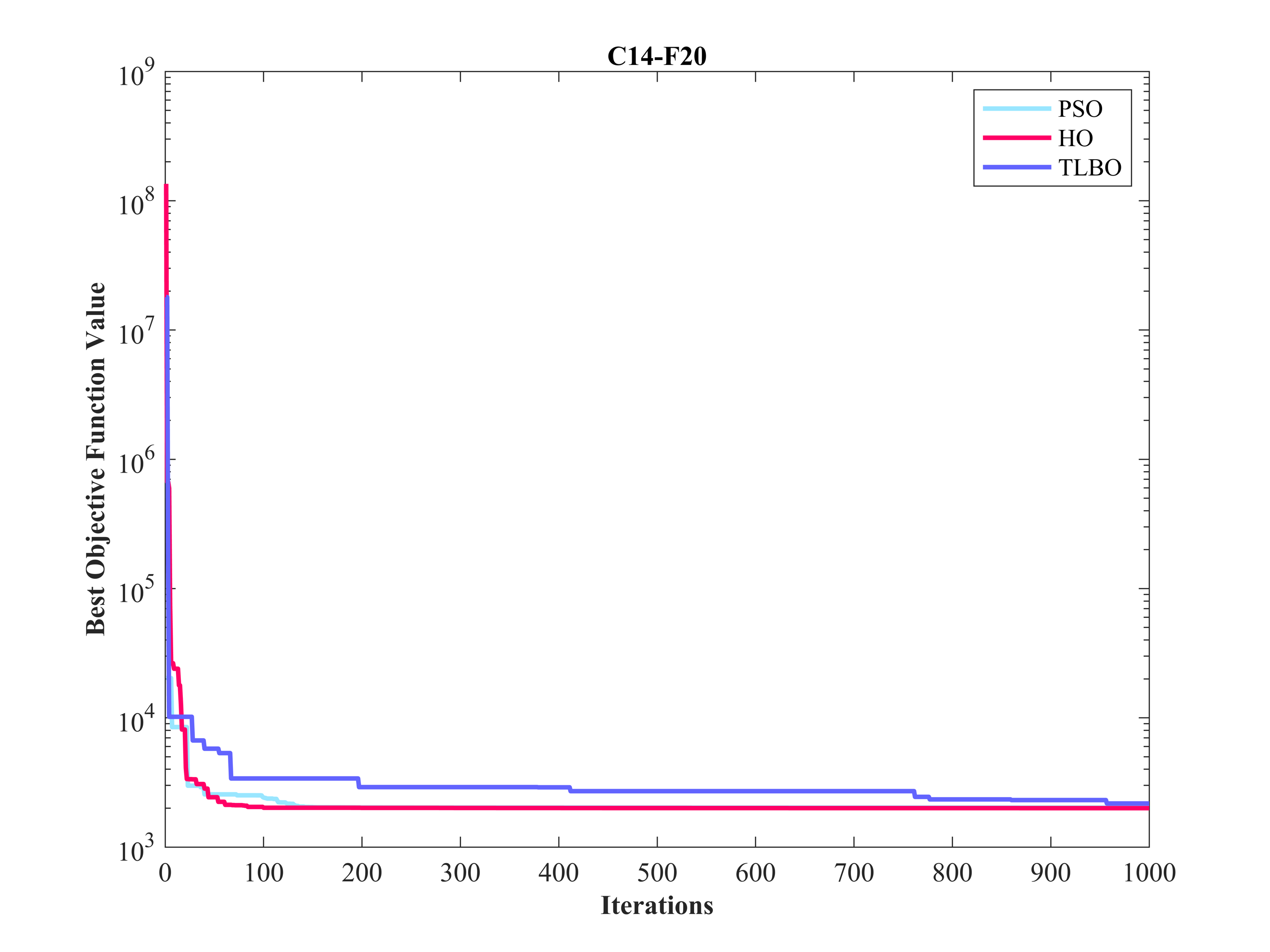 |
| 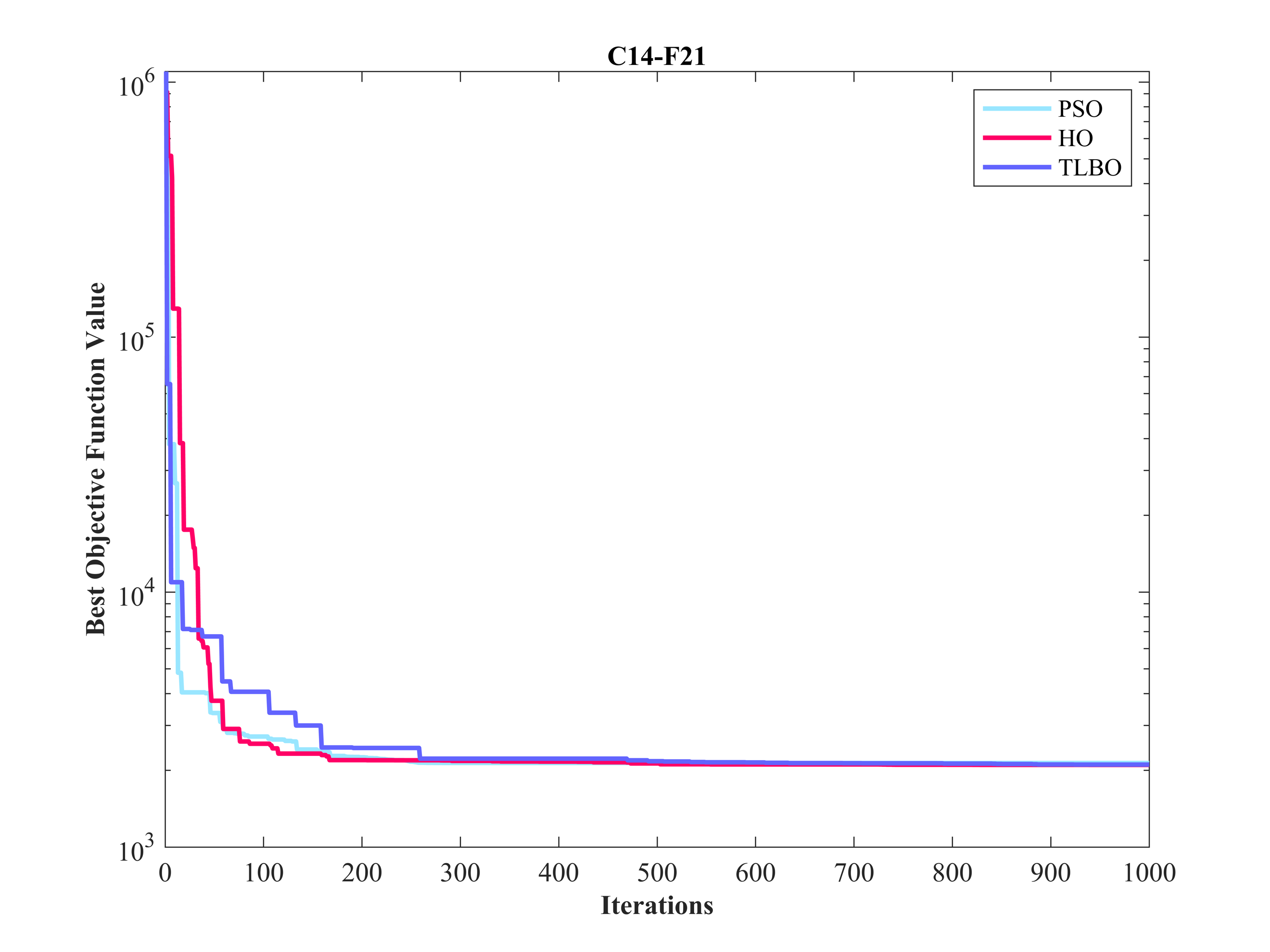 | 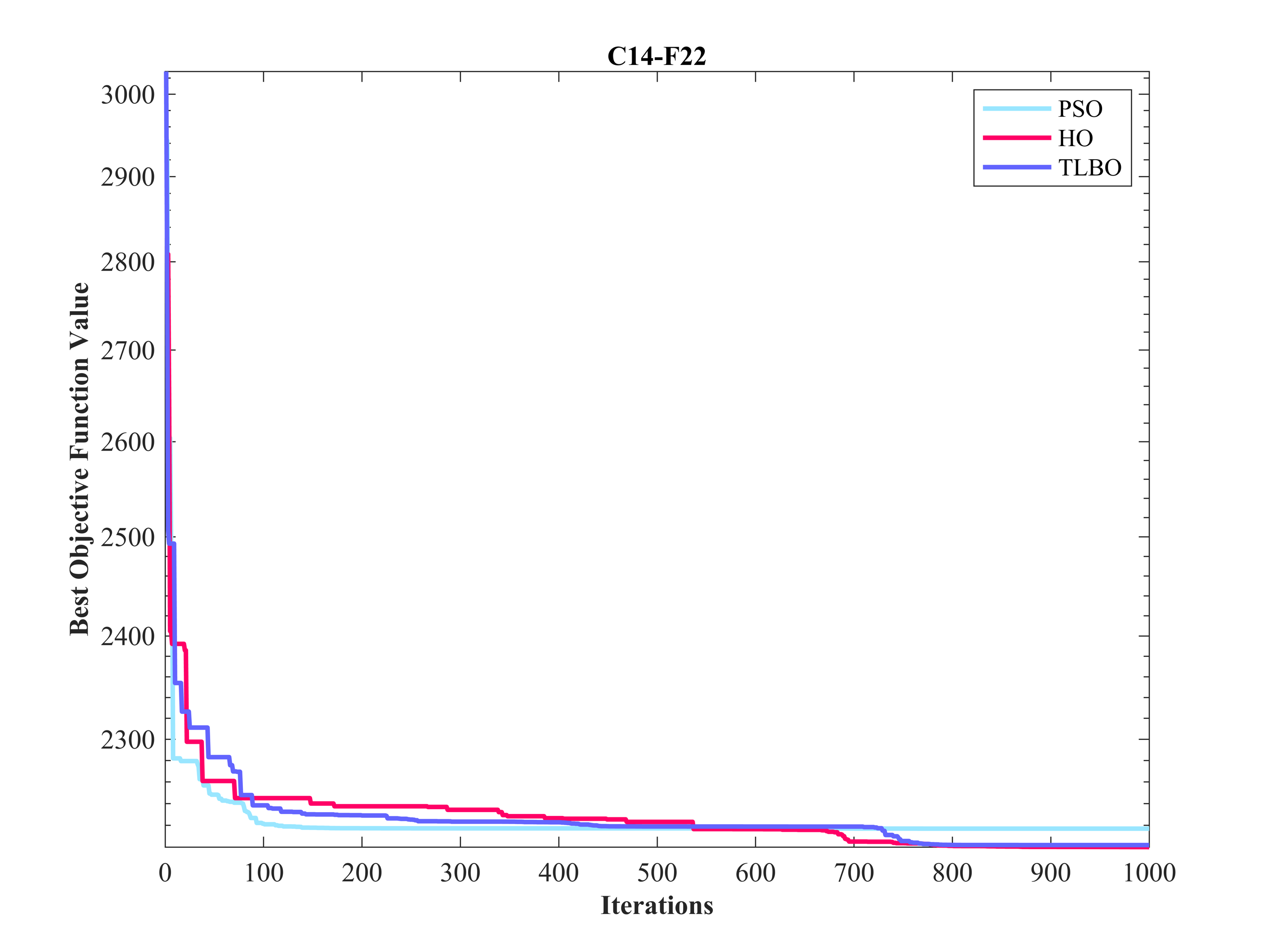 |
| 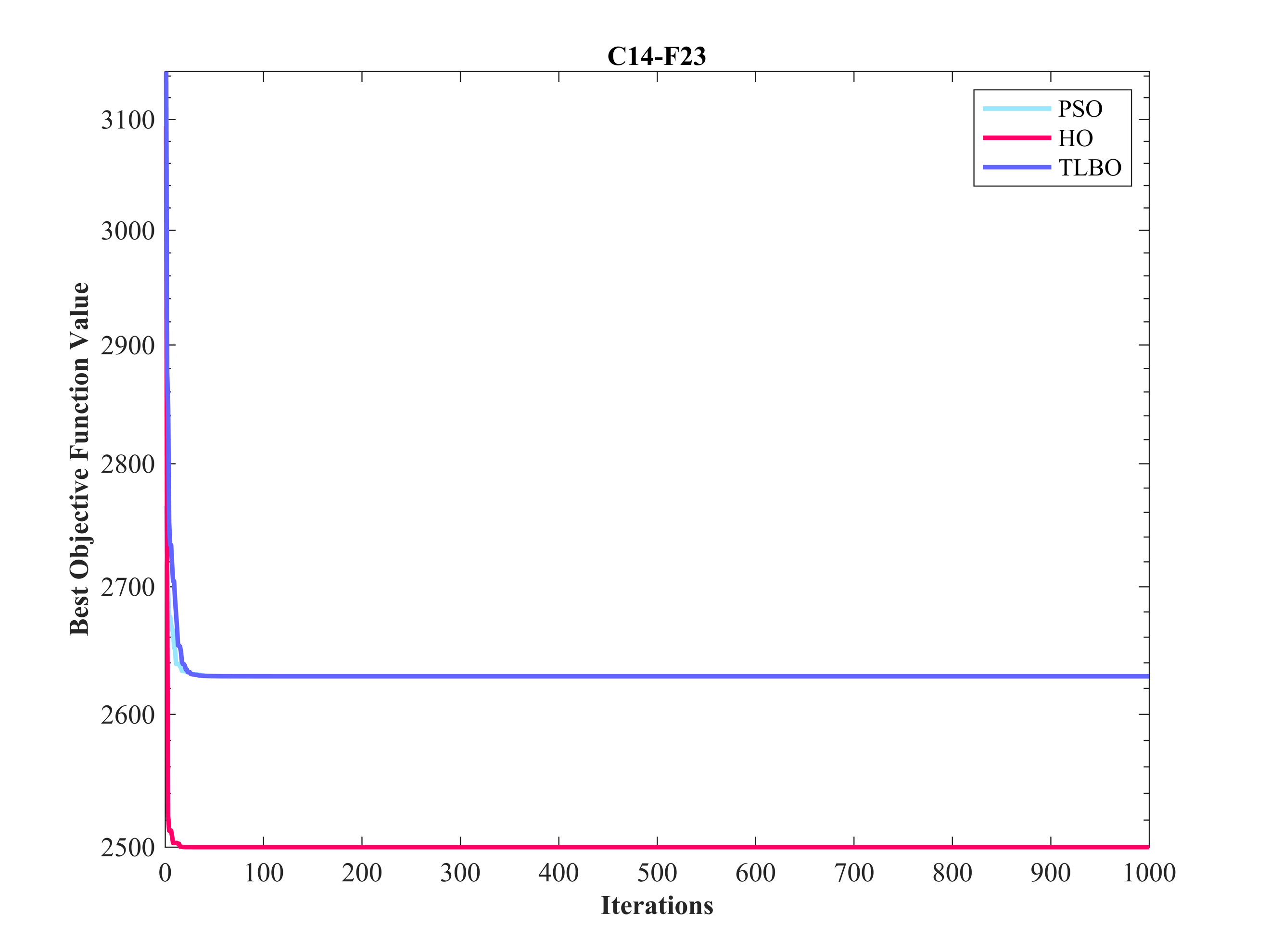 | 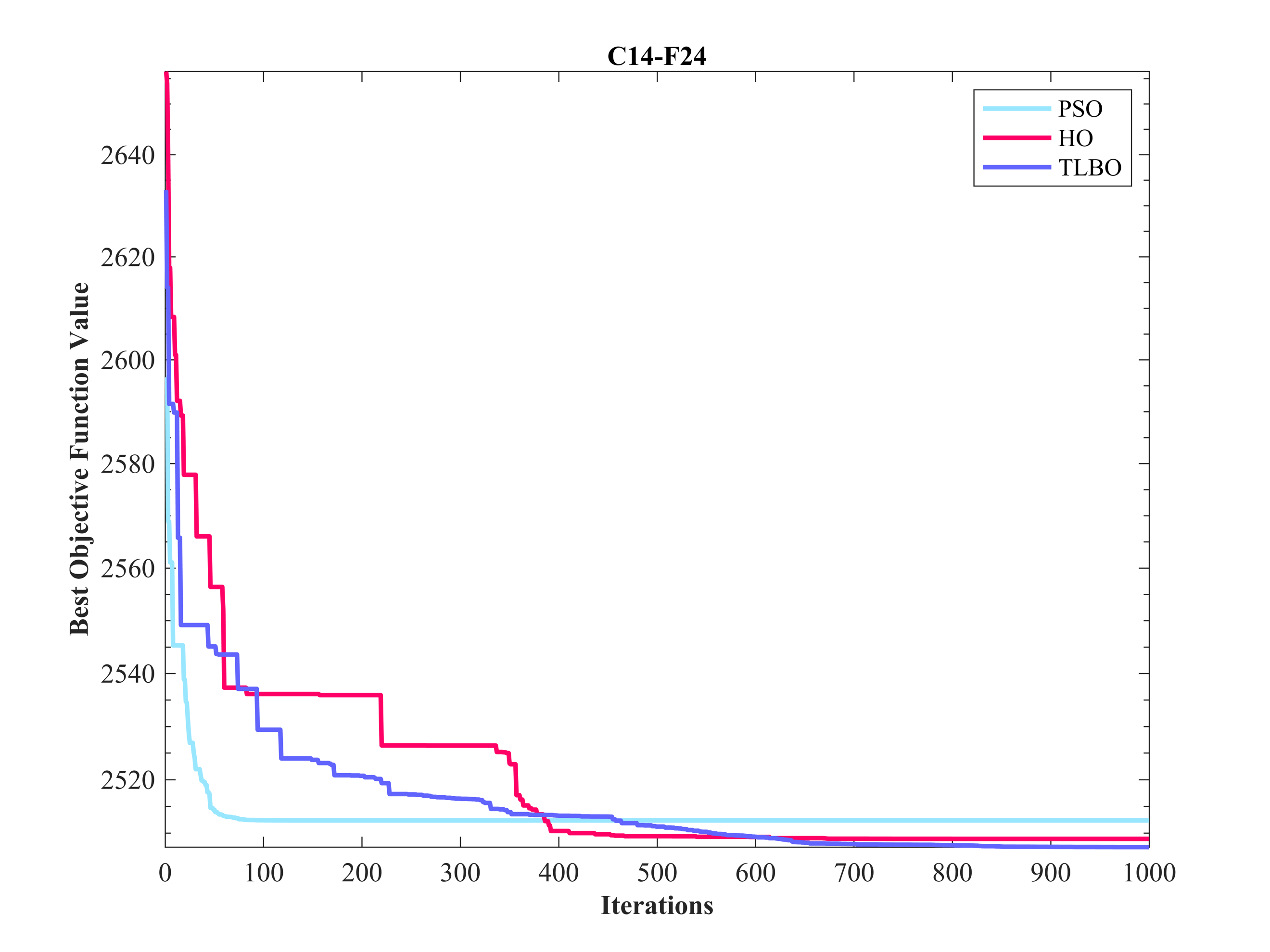 |

**Figure S3.** (continued)

| 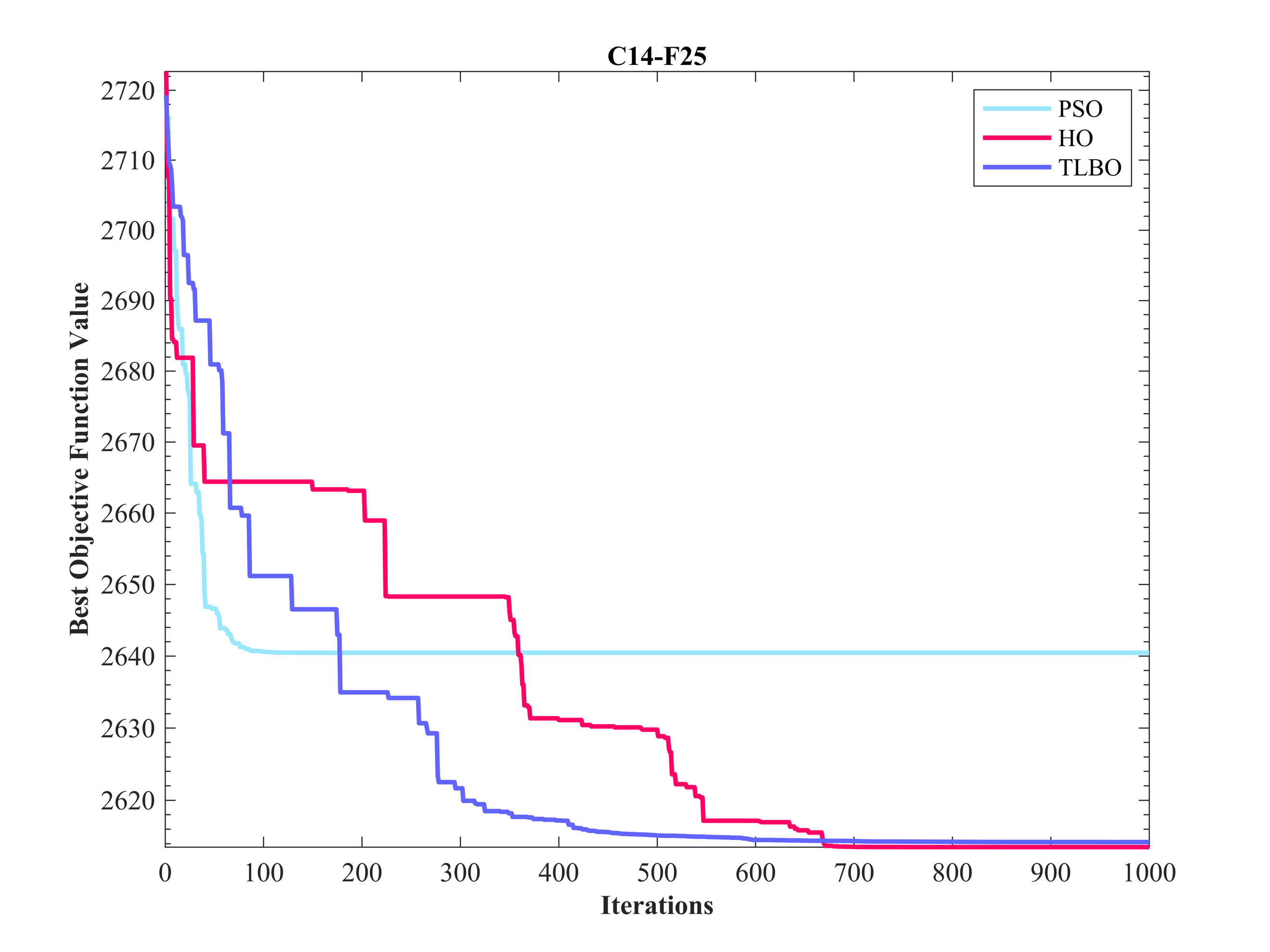 | 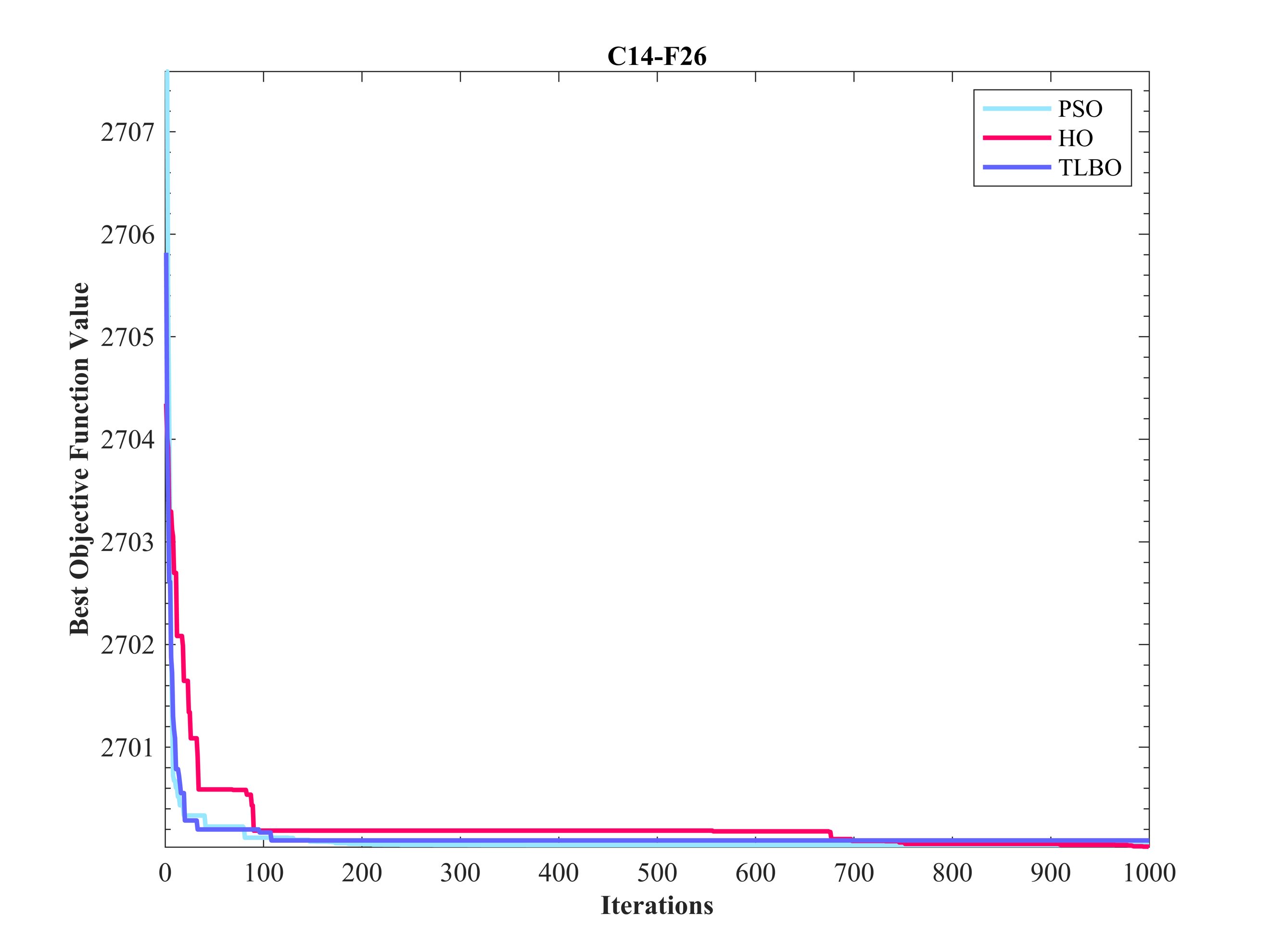 |
| --- | --- |
| 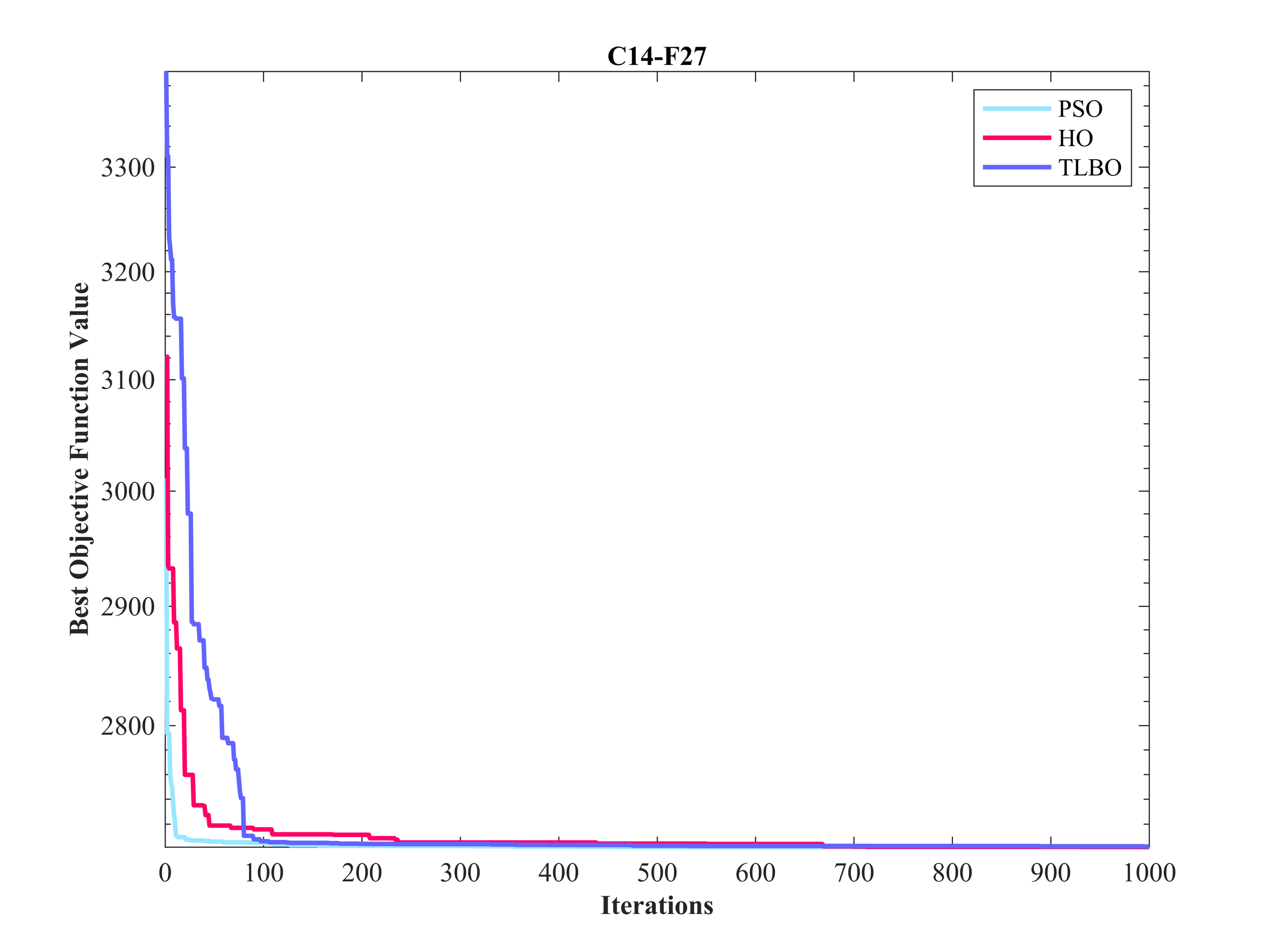 | 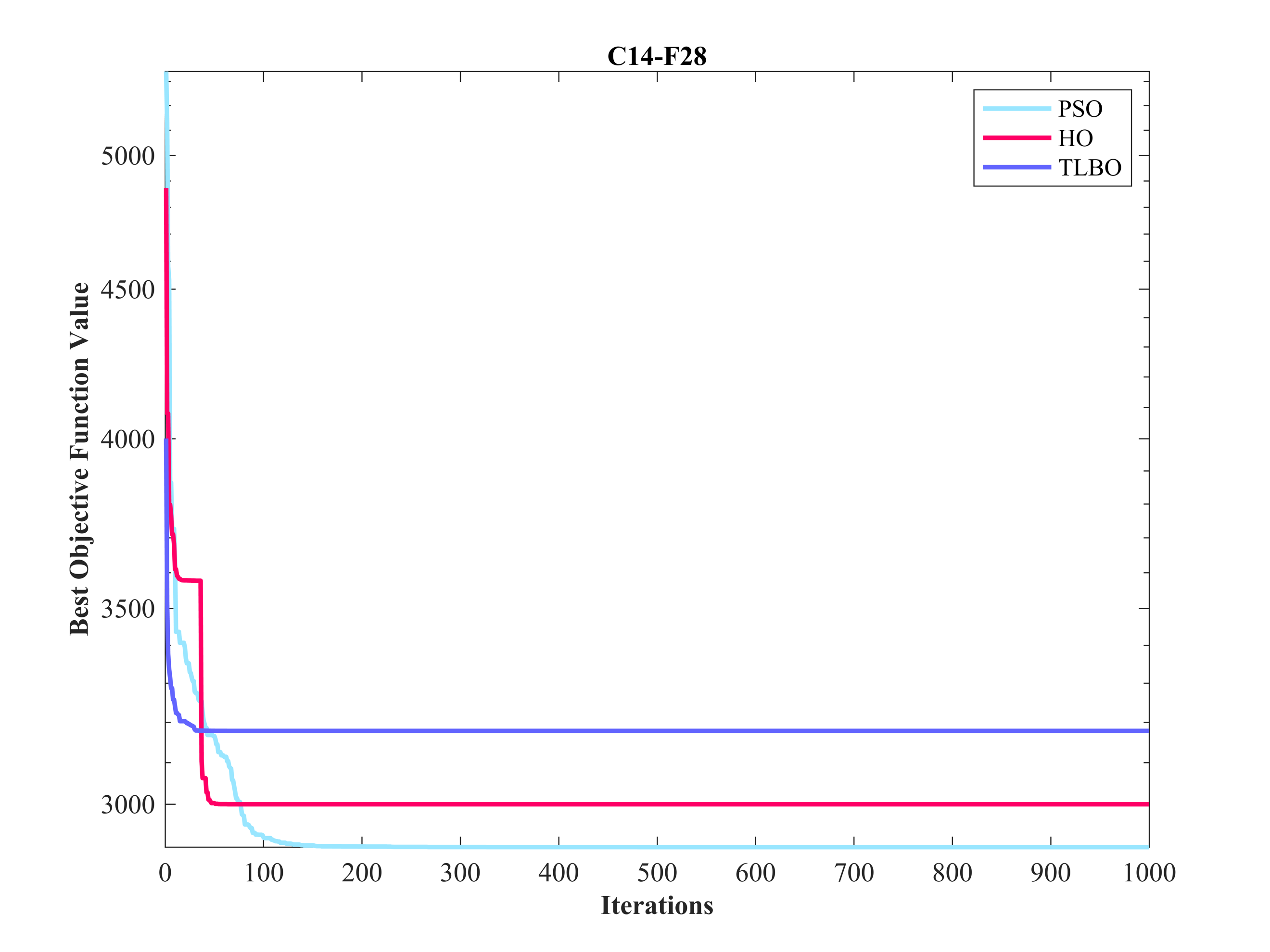 |
| 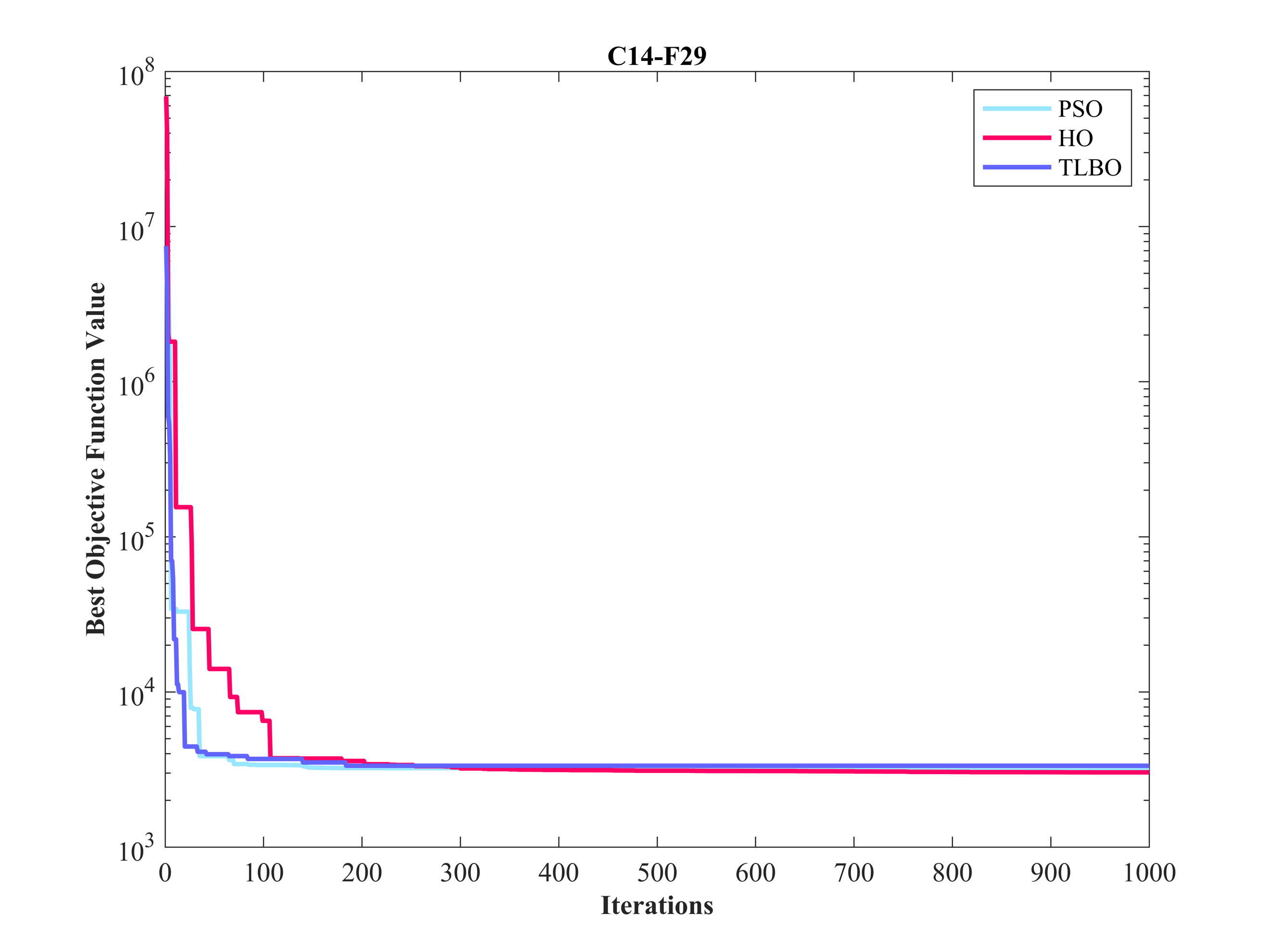 | 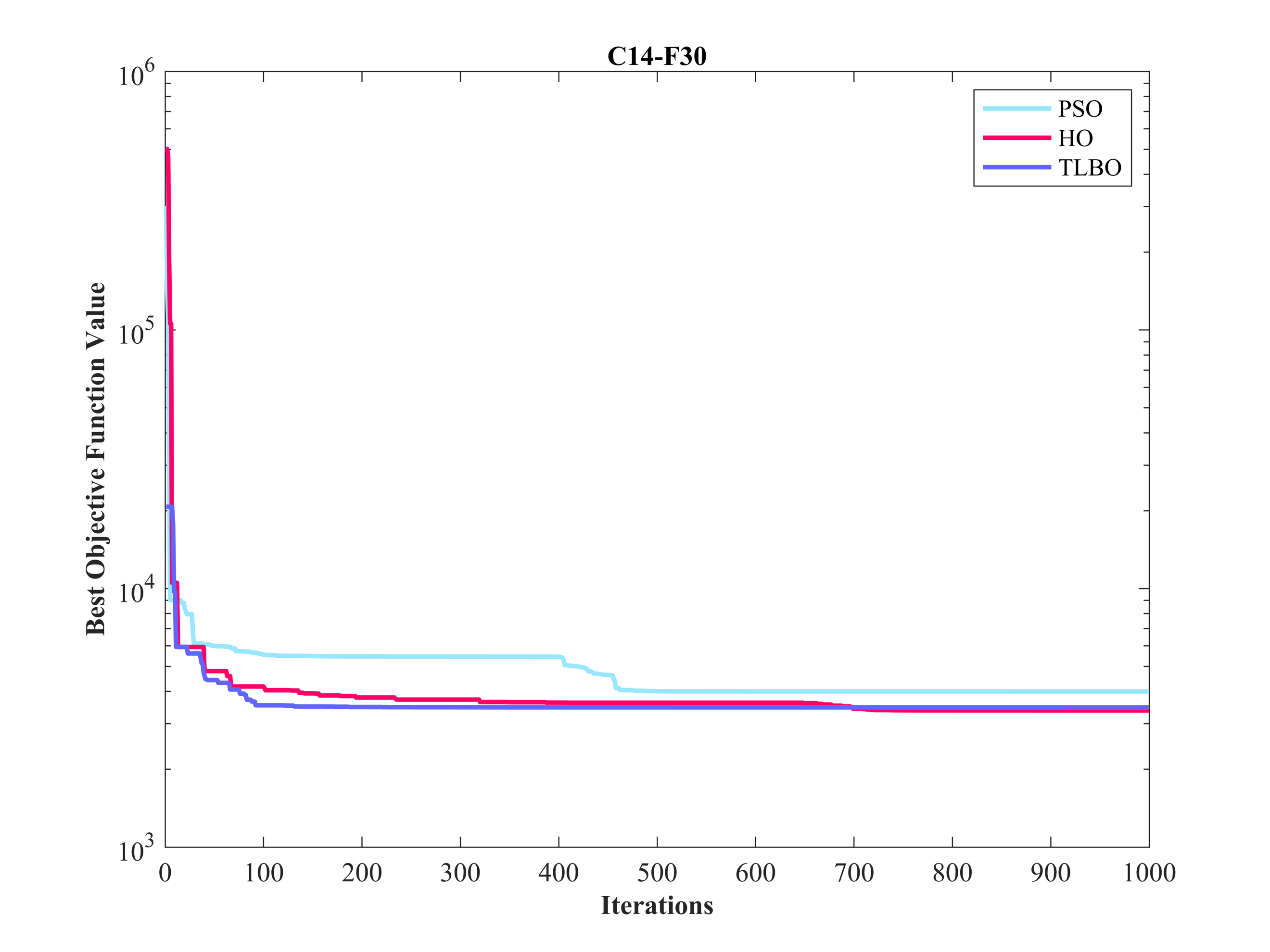 |

**Figure S3.** (continued)

Evaluation outcomes for the objectives specified in the CEC 2014. (D = 30).

| **F** | **M** | **Optimization Algorithms** | | | | | | | | | | | | |
| --- | --- | --- | --- | --- | --- | --- | --- | --- | --- | --- | --- | --- | --- | --- |
|  |  | **HO** | **WOA** | **GWO** | **SSA** | **PSO** | **SCA** | **FA** | **GOA** | **TLBO** | **CMA-ES** | **MFO** | **AOA** | **IWO** |
| C14-F1 | Mean | 1.37E+06 | 1.25E+08 | 1.69E+08 | 2.30E+07 | 1.87E+06 | 4.42E+08 | 1.51E+08 | 5.72E+06 | 2.26E+06 | 1.25E+08 | 1.02E+08 | 1.11E+09 | 1.54E+07 |
|  | Best | 3.99E+05 | 4.82E+07 | 3.84E+07 | 5.70E+06 | 4.99E+05 | 2.09E+08 | 3.10E+07 | 4.02E+05 | 3.88E+05 | 3.98E+07 | 2.12E+07 | 2.83E+08 | 1.97E+06 |
|  | Worst | 3.36E+06 | 2.48E+08 | 3.88E+08 | 5.23E+07 | 4.15E+06 | 7.41E+08 | 3.64E+08 | 3.97E+07 | 1.02E+07 | 2.62E+08 | 5.27E+08 | 2.04E+09 | 7.03E+07 |
|  | Std. | 7.87E+05 | 4.99E+07 | 1.08E+08 | 1.16E+07 | 9.02E+05 | 1.34E+08 | 9.40E+07 | 7.56E+06 | 2.21E+06 | 5.07E+07 | 1.25E+08 | 4.27E+08 | 1.37E+07 |
|  | Median | 1.28E+06 | 1.26E+08 | 1.27E+08 | 1.97E+07 | 1.72E+06 | 4.18E+08 | 1.26E+08 | 2.87E+06 | 1.61E+06 | 1.21E+08 | 5.91E+07 | 1.03E+09 | 1.15E+07 |
|  | Rank | 1 | 9 | 11 | 6 | 2 | 12 | 10 | 4 | 3 | 8 | 7 | 13 | 5 |
| C14-F2 | Mean | 30516 | 1.26E+09 | 8.85E+09 | 15671 | 10213 | 2.53E+10 | 5.10E+09 | 4.82E+07 | 13391 | 3.98E+05 | 1.23E+10 | 7.14E+10 | 8705.5 |
|  | Best | 1721.9 | 3.84E+08 | 2.77E+09 | 217.36 | 515.94 | 1.83E+10 | 8.65E+08 | 395.82 | 547.06 | 35413 | 1.80E+09 | 4.96E+10 | 316.26 |
|  | Worst | 1.43E+05 | 5.11E+09 | 1.88E+10 | 32315 | 21259 | 3.09E+10 | 1.28E+10 | 1.45E+09 | 34668 | 1.63E+06 | 3.25E+10 | 8.83E+10 | 34304 |
|  | Std. | 28989 | 9.46E+08 | 3.83E+09 | 11407 | 5640.8 | 3.41E+09 | 2.95E+09 | 2.64E+08 | 11457 | 3.49E+05 | 7.15E+09 | 1.06E+10 | 8432.2 |
|  | Median | 24408 | 1.08E+09 | 8.65E+09 | 16806 | 10868 | 2.60E+10 | 4.76E+09 | 14452 | 9254.9 | 3.68E+05 | 1.14E+10 | 7.13E+10 | 6460.5 |
|  | Rank | 5 | 8 | 10 | 4 | 2 | 12 | 9 | 7 | 3 | 6 | 11 | 13 | 1 |
| C14-F3 | Mean | 393.22 | 89733 | 50682 | 71608 | 5476.1 | 62678 | 88426 | 16322 | 12493 | 71049 | 1.13E+05 | 81426 | 1.72E+05 |
|  | Best | 301.15 | 40832 | 31503 | 42641 | 314.17 | 40434 | 47061 | 585.22 | 3141.6 | 15663 | 6960 | 59947 | 94517 |
|  | Worst | 1249.6 | 3.07E+05 | 65594 | 1.21E+05 | 20037 | 98791 | 1.75E+05 | 47098 | 25671 | 1.29E+05 | 2.32E+05 | 1.18E+05 | 2.88E+05 |
|  | Std. | 192.95 | 55615 | 7758.5 | 25179 | 4935.1 | 13431 | 29779 | 14111 | 5255 | 26331 | 57463 | 10239 | 45014 |
|  | Median | 342.4 | 72675 | 49417 | 61709 | 3640.3 | 64531 | 89088 | 10770 | 11893 | 78337 | 1.20E+05 | 81170 | 1.65E+05 |
|  | Rank | 1 | 11 | 5 | 8 | 2 | 6 | 10 | 4 | 3 | 7 | 12 | 9 | 13 |
| C14-F4 | Mean | 488.6 | 828.93 | 938.46 | 534.85 | 489.62 | 2424.3 | 1061.6 | 524.66 | 529.16 | 522.74 | 1291.7 | 12097 | 539.9 |
|  | Best | 400.66 | 659.01 | 663.99 | 476.63 | 400.53 | 1175.9 | 737.59 | 404.42 | 426.73 | 516.16 | 529.67 | 4303 | 472.79 |
|  | Worst | 543.18 | 1058 | 1672.6 | 635.08 | 554.23 | 4649.2 | 1491.4 | 984.36 | 613.39 | 532.43 | 4739.4 | 19289 | 637.58 |
|  | Std. | 28.425 | 94.448 | 251.43 | 33.112 | 40.288 | 817.69 | 204.47 | 102.37 | 39.752 | 3.9382 | 892.83 | 4144.7 | 36.955 |
|  | Median | 480.68 | 814.3 | 854.38 | 535.78 | 477.87 | 2203.9 | 1071.3 | 501.61 | 532.33 | 521.63 | 1053.8 | 12223 | 540.23 |
|  | Rank | 1 | 8 | 9 | 6 | 2 | 12 | 10 | 4 | 5 | 3 | 11 | 13 | 7 |
| C14-F5 | Mean | 520 | 520.8 | 520.64 | 520.09 | 520.74 | 521.05 | 520.07 | 520.02 | 520.99 | 520.99 | 520.27 | 520.91 | 520 |
|  | Best | 520 | 520.48 | 520.38 | 520 | 520.17 | 520.93 | 520 | 520 | 520.8 | 520.79 | 520 | 520.78 | 520 |
|  | Worst | 520 | 521 | 520.95 | 520.47 | 521.06 | 521.13 | 520.23 | 520.1 | 521.07 | 521.08 | 520.64 | 521.04 | 520 |
|  | Std. | 7.53E-06 | 0.13284 | 0.137 | 0.11797 | 0.25878 | 0.048846 | 0.050923 | 0.027256 | 0.064693 | 0.05028 | 0.14802 | 0.080663 | 0.00096255 |
|  | Median | 520 | 520.79 | 520.63 | 520.04 | 520.81 | 521.05 | 520.06 | 520 | 521.01 | 521 | 520.27 | 520.9 | 520 |
|  | Rank | 1 | 9 | 7 | 5 | 8 | 13 | 4 | 3 | 12 | 11 | 6 | 10 | 2 |
| C14-F6 | Mean | 613.18 | 638.45 | 624.19 | 624.5 | 618.15 | 637.73 | 633.42 | 626.25 | 618.75 | 628.49 | 623.07 | 638.66 | 634.45 |
|  | Best | 605.66 | 633.01 | 618.09 | 615.99 | 609.83 | 633.72 | 625.5 | 620.69 | 613.7 | 610.74 | 615.22 | 633.81 | 627 |
|  | Worst | 620.24 | 644.42 | 632.75 | 635.55 | 625.28 | 642.28 | 641.15 | 632.61 | 625.07 | 637.31 | 629.75 | 642.97 | 639.81 |
|  | Std. | 3.1868 | 3.0866 | 3.6512 | 4.7831 | 3.4807 | 1.9942 | 3.1898 | 3.2351 | 2.8018 | 8.2019 | 3.5927 | 2.5776 | 3.0816 |
|  | Median | 613.4 | 637.97 | 623.89 | 623.55 | 617.6 | 637.36 | 633.58 | 626.21 | 619.38 | 631.86 | 622.69 | 639.32 | 635.45 |
|  | Rank | 1 | 12 | 5 | 6 | 2 | 11 | 9 | 7 | 3 | 8 | 4 | 13 | 10 |
| C14-F7 | Mean | 700.01 | 708.57 | 779.7 | 700.18 | 700.02 | 914.06 | 760.99 | 700.53 | 700.15 | 700.62 | 808.6 | 1386.4 | 700.01 |
|  | Best | 700 | 704 | 719.14 | 700.04 | 700 | 850.86 | 705.58 | 700 | 700 | 700.12 | 722.47 | 1209.9 | 700 |
|  | Worst | 700.03 | 715.78 | 851.01 | 700.67 | 700.07 | 974.99 | 841.68 | 708.05 | 702.06 | 700.88 | 943.76 | 1626.3 | 700.04 |
|  | Std. | 0.0081264 | 2.808 | 35.529 | 0.13614 | 0.021583 | 37.052 | 38.42 | 1.9326 | 0.37788 | 0.21522 | 63.351 | 98.142 | 0.011793 |
|  | Median | 700.01 | 707.95 | 780.03 | 700.14 | 700.01 | 915.99 | 757.91 | 700.01 | 700.04 | 700.7 | 788.99 | 1388 | 700.01 |
|  | Rank | 1 | 8 | 10 | 5 | 3 | 12 | 9 | 6 | 4 | 7 | 11 | 13 | 2 |

**Table S2.** Evaluation outcomes for the objectives specified in the CEC 2014 (D =30).

| C14-F8 | Mean | 849.7 | 1036.6 | 972.95 | 950.14 | 917.04 | 1066.8 | 1076.8 | 997.76 | 885.42 | 1012.4 | 938.82 | 1135 | 1083.2 |
| --- | --- | --- | --- | --- | --- | --- | --- | --- | --- | --- | --- | --- | --- | --- |
|  | Best | 830.85 | 985.03 | 891.92 | 886.64 | 881.59 | 1033.5 | 1010.9 | 931.09 | 851.05 | 967 | 879.75 | 1083.8 | 1004 |
|  | Worst | 879.61 | 1158.7 | 1032.9 | 1045.8 | 958.2 | 1109.4 | 1168.1 | 1133.7 | 924.37 | 1032.2 | 1051.6 | 1191.3 | 1164.2 |
|  | Std. | 12.07 | 48.978 | 34.562 | 37.147 | 19.119 | 18.13 | 38.96 | 44.439 | 16.048 | 15.299 | 34.929 | 25.952 | 48.579 |
|  | Median | 848.27 | 1026.4 | 971.86 | 951.23 | 916.91 | 1064.7 | 1075.6 | 989.78 | 886.56 | 1014.8 | 941.43 | 1134.2 | 1080 |
|  | Rank | 1 | 9 | 6 | 5 | 3 | 10 | 11 | 7 | 2 | 8 | 4 | 13 | 12 |
| C14-F9 | Mean | 997.21 | 1190.1 | 1078.6 | 1065.9 | 1023.4 | 1214.7 | 1273.1 | 1255 | 1004.8 | 1136.5 | 1110.8 | 1218.8 | 1332.5 |
|  | Best | 944.36 | 1076.4 | 996.07 | 978.6 | 970.64 | 1180.5 | 1149.7 | 1154.7 | 977.43 | 1099.1 | 1015.4 | 1162.7 | 1159.7 |
|  | Worst | 1039.4 | 1363.2 | 1163 | 1128.8 | 1102 | 1249.2 | 1398.5 | 1379.6 | 1042.8 | 1158 | 1283.8 | 1268.9 | 1476.1 |
|  | Std. | 23.774 | 59.004 | 34.352 | 36.058 | 32.25 | 20.091 | 62.297 | 61.51 | 15.597 | 12.309 | 57.218 | 23.703 | 82.346 |
|  | Median | 998.24 | 1184.8 | 1071.3 | 1066.7 | 1014.4 | 1213.1 | 1266.1 | 1246.8 | 1008.7 | 1137.2 | 1105 | 1219.1 | 1341.9 |
|  | Rank | 1 | 8 | 5 | 4 | 3 | 9 | 12 | 11 | 2 | 7 | 6 | 10 | 13 |
| C14-F10 | Mean | 2191.5 | 5900.4 | 4679.4 | 5005.7 | 3578.3 | 7744.3 | 5319.8 | 3733.7 | 3253.5 | 6641 | 4480.2 | 7365.5 | 5436 |
|  | Best | 1681.4 | 4190 | 3586.5 | 3281.9 | 2329.3 | 6755.8 | 3810.8 | 2743.4 | 1915.5 | 5912.3 | 2370.9 | 6347.6 | 3660.4 |
|  | Worst | 2748.1 | 7405.9 | 5882.3 | 6467 | 5058.3 | 8711.2 | 6189.7 | 4536.4 | 4537.5 | 7471.8 | 5619.1 | 8198.3 | 6662.2 |
|  | Std. | 302.18 | 857.57 | 606.83 | 816.89 | 702.88 | 531.38 | 612.06 | 449.53 | 556.84 | 400.14 | 811.47 | 490.26 | 783 |
|  | Median | 2136.6 | 5872.3 | 4786.7 | 5076.8 | 3568.1 | 7808.8 | 5356.3 | 3747.3 | 3207.4 | 6698.4 | 4600.6 | 7338.1 | 5729.3 |
|  | Rank | 1 | 10 | 6 | 7 | 3 | 13 | 8 | 4 | 2 | 11 | 5 | 12 | 9 |
| C14-F11 | Mean | 3825.2 | 7234 | 5278 | 4860.4 | 4844.3 | 8584.8 | 5164.5 | 4846.7 | 7771.5 | 8517.4 | 5252.8 | 7687.3 | 5533.2 |
|  | Best | 2546.2 | 5915.8 | 3949.6 | 3211.8 | 3248.3 | 7788.1 | 4331.9 | 3486.3 | 6625 | 8025 | 4088.3 | 6568.4 | 4385.2 |
|  | Worst | 4602.5 | 8629.9 | 6376.2 | 6009.4 | 6201.6 | 9286.1 | 5875.4 | 6093.5 | 8464.3 | 8947.6 | 7451.7 | 8678.7 | 6550.8 |
|  | Std. | 506.44 | 833.12 | 633.79 | 737.6 | 739.57 | 362.02 | 429.84 | 647.24 | 607.54 | 249.84 | 810.34 | 538.63 | 611.06 |
|  | Median | 3836.7 | 7302.1 | 5215.9 | 4901.8 | 4847.6 | 8702 | 5192.9 | 4874.5 | 7989.4 | 8510.4 | 5229.2 | 7874.3 | 5505.4 |
|  | Rank | 1 | 9 | 7 | 4 | 2 | 13 | 5 | 3 | 11 | 12 | 6 | 10 | 8 |
| C14-F12 | Mean | 1200 | 1202.1 | 1200.8 | 1200.7 | 1200.4 | 1203.1 | 1200.1 | 1200.2 | 1202.9 | 1202.9 | 1200.5 | 1202 | 1200.1 |
|  | Best | 1200 | 1201.2 | 1200.3 | 1200.2 | 1200.1 | 1202.4 | 1200 | 1200.1 | 1201.8 | 1202.1 | 1200.1 | 1200.7 | 1200.1 |
|  | Worst | 1200 | 1203.1 | 1201.7 | 1201.8 | 1200.9 | 1203.7 | 1200.1 | 1200.4 | 1203.6 | 1203.7 | 1201.7 | 1202.6 | 1200.3 |
|  | Std. | 0.0096435 | 0.62065 | 0.35359 | 0.34554 | 0.19763 | 0.31092 | 0.031548 | 0.087011 | 0.41566 | 0.37858 | 0.31636 | 0.39974 | 0.053524 |
|  | Median | 1200 | 1202.1 | 1200.7 | 1200.7 | 1200.4 | 1203.2 | 1200.1 | 1200.2 | 1203 | 1203 | 1200.4 | 1202 | 1200.1 |
|  | Rank | 1 | 10 | 8 | 7 | 5 | 13 | 2 | 4 | 11 | 12 | 6 | 9 | 3 |
| C14-F13 | Mean | 1300.6 | 1300.5 | 1301.7 | 1300.3 | 1300.4 | 1303.9 | 1301.4 | 1300.6 | 1300.5 | 1300.7 | 1302 | 1307.3 | 1300.4 |
|  | Best | 1300.3 | 1300.3 | 1300.5 | 1300.2 | 1300.3 | 1303.2 | 1300.3 | 1300.4 | 1300.3 | 1300.4 | 1300.6 | 1305.6 | 1300.3 |
|  | Worst | 1300.8 | 1300.8 | 1303.2 | 1300.5 | 1300.7 | 1304.9 | 1302.8 | 1300.9 | 1300.7 | 1300.9 | 1303.7 | 1309.5 | 1300.6 |
|  | Std. | 0.1208 | 0.11144 | 0.95456 | 0.076651 | 0.093736 | 0.4701 | 0.9236 | 0.132 | 0.095834 | 0.098663 | 1.2056 | 0.88688 | 0.077431 |
|  | Median | 1300.6 | 1300.5 | 1301.6 | 1300.3 | 1300.4 | 1303.9 | 1301.4 | 1300.6 | 1300.5 | 1300.7 | 1302.2 | 1307.4 | 1300.4 |
|  | Rank | 6 | 4 | 10 | 1 | 2 | 12 | 9 | 7 | 5 | 8 | 11 | 13 | 3 |
| C14-F14 | Mean | 1400.3 | 1400.9 | 1419.7 | 1400.4 | 1400.3 | 1472.1 | 1422.4 | 1400.5 | 1400.3 | 1400.9 | 1430.3 | 1649.7 | 1400.3 |
|  | Best | 1400.2 | 1400.2 | 1400.2 | 1400.2 | 1400.2 | 1446.1 | 1400.4 | 1400.2 | 1400.2 | 1400.4 | 1400.4 | 1580.5 | 1400.1 |
|  | Worst | 1400.9 | 1414.9 | 1440.5 | 1401.2 | 1400.8 | 1509.6 | 1444 | 1401 | 1400.9 | 1401.2 | 1526.1 | 1726.7 | 1400.4 |
|  | Std. | 0.1204 | 2.8275 | 11.103 | 0.23532 | 0.14778 | 12.488 | 11.981 | 0.26794 | 0.1474 | 0.259 | 28.431 | 39.696 | 0.059527 |
|  | Median | 1400.2 | 1400.3 | 1419.2 | 1400.3 | 1400.3 | 1471 | 1420.5 | 1400.4 | 1400.3 | 1401 | 1422.9 | 1641.8 | 1400.3 |
|  | Rank | 1 | 8 | 9 | 5 | 4 | 12 | 10 | 6 | 3 | 7 | 11 | 13 | 2 |

**Table S2.** (continued)

| C14-F15 | Mean | 1507.9 | 1671.8 | 3361.4 | 1513.4 | 1509.7 | 18485 | 3219.1 | 1526 | 1535.5 | 1522.5 | 3.47E+05 | 2.52E+05 | 1520.5 |
| --- | --- | --- | --- | --- | --- | --- | --- | --- | --- | --- | --- | --- | --- | --- |
|  | Best | 1503.3 | 1558 | 1579.8 | 1506.5 | 1504.8 | 3950.2 | 2076.1 | 1504.9 | 1515.6 | 1519.7 | 1537.3 | 41918 | 1512.2 |
|  | Worst | 1515.3 | 1944.3 | 8282.3 | 1524.1 | 1515.1 | 1.07E+05 | 6979.4 | 1957 | 1599 | 1524.6 | 1.81E+06 | 5.94E+05 | 1532.1 |
|  | Std. | 3.3678 | 87.407 | 1747.3 | 4.4518 | 2.5794 | 19065 | 1319.3 | 81.605 | 17.713 | 1.2967 | 4.68E+05 | 1.50E+05 | 5.0549 |
|  | Median | 1507.3 | 1661.4 | 2805.1 | 1511.9 | 1509.3 | 13293 | 2599.8 | 1510 | 1533.3 | 1522.5 | 59053 | 2.27E+05 | 1520 |
|  | Rank | 1 | 8 | 10 | 3 | 2 | 11 | 9 | 6 | 7 | 5 | 13 | 12 | 4 |
| C14-F16 | Mean | 1611.4 | 1612.9 | 1612.3 | 1612.3 | 1612 | 1613.2 | 1613.3 | 1613.4 | 1612.3 | 1613.1 | 1612.6 | 1612.9 | 1613.7 |
|  | Best | 1610.3 | 1612.1 | 1610.6 | 1610.8 | 1610.7 | 1612.9 | 1612.7 | 1612.4 | 1610.9 | 1612.2 | 1611.5 | 1612.1 | 1612.7 |
|  | Worst | 1612.4 | 1613.8 | 1613.4 | 1613.5 | 1612.7 | 1613.6 | 1613.7 | 1614.3 | 1613.1 | 1613.6 | 1613.7 | 1613.6 | 1614.4 |
|  | Std. | 0.60221 | 0.40008 | 0.59927 | 0.59998 | 0.45025 | 0.19033 | 0.31419 | 0.4112 | 0.43388 | 0.2696 | 0.54524 | 0.36699 | 0.42611 |
|  | Median | 1611.5 | 1612.9 | 1612.3 | 1612.2 | 1612.1 | 1613.2 | 1613.4 | 1613.4 | 1612.3 | 1613.2 | 1612.7 | 1612.9 | 1613.6 |
|  | Rank | 1 | 8 | 3 | 4 | 2 | 10 | 11 | 12 | 5 | 9 | 6 | 7 | 13 |
| C14-F17 | Mean | 2763.9 | 1.53E+07 | 4.54E+06 | 1.41E+06 | 4.27E+05 | 1.51E+07 | 2.41E+06 | 5.01E+05 | 4.32E+05 | 3.82E+06 | 3.74E+06 | 8.91E+07 | 4.35E+05 |
|  | Best | 2139.1 | 9.91E+05 | 3.97E+05 | 3.13E+05 | 33183 | 5.41E+06 | 65907 | 20009 | 62153 | 7.32E+05 | 2.77E+05 | 9.10E+06 | 32211 |
|  | Worst | 3491.5 | 4.91E+07 | 1.49E+07 | 3.53E+06 | 1.15E+06 | 3.21E+07 | 7.44E+06 | 2.87E+06 | 1.98E+06 | 8.77E+06 | 2.89E+07 | 2.01E+08 | 1.03E+06 |
|  | Std. | 350.3 | 1.33E+07 | 3.79E+06 | 8.88E+05 | 2.73E+05 | 7.09E+06 | 2.38E+06 | 6.71E+05 | 4.11E+05 | 2.03E+06 | 5.25E+06 | 4.13E+07 | 2.91E+05 |
|  | Median | 2674.1 | 1.13E+07 | 3.55E+06 | 1.20E+06 | 3.52E+05 | 1.33E+07 | 1.21E+06 | 2.77E+05 | 3.17E+05 | 3.14E+06 | 2.16E+06 | 8.58E+07 | 3.67E+05 |
|  | Rank | 1 | 12 | 10 | 6 | 2 | 11 | 7 | 5 | 3 | 9 | 8 | 13 | 4 |
| C14-F18 | Mean | 1889.3 | 5.36E+05 | 5.15E+07 | 9658.7 | 4501.8 | 3.36E+08 | 3272.1 | 8189.2 | 4124.2 | 4763.5 | 4.24E+07 | 2.97E+09 | 6087.4 |
|  | Best | 1853.4 | 12117 | 8213.1 | 2227.6 | 1873.1 | 1.05E+08 | 2080.4 | 2231.2 | 1888.3 | 1935 | 2851.3 | 2.77E+08 | 2289.6 |
|  | Worst | 1925.7 | 3.39E+06 | 1.62E+08 | 29499 | 19302 | 6.19E+08 | 8933.4 | 27724 | 10781 | 14048 | 7.51E+08 | 8.24E+09 | 15108 |
|  | Std. | 18.205 | 7.90E+05 | 5.41E+07 | 8880.2 | 4246.1 | 1.42E+08 | 1857.3 | 5983.3 | 2601 | 3479.5 | 1.49E+08 | 2.08E+09 | 4158.1 |
|  | Median | 1894.8 | 2.35E+05 | 3.13E+07 | 6061.9 | 2372.3 | 3.44E+08 | 2311.6 | 6127.8 | 3044.2 | 3764.7 | 13651 | 2.65E+09 | 4154.1 |
|  | Rank | 1 | 9 | 11 | 8 | 4 | 12 | 2 | 7 | 3 | 5 | 10 | 13 | 6 |
| C14-F19 | Mean | 1907.3 | 1971.9 | 1970.6 | 1920.2 | 1924.5 | 2028.2 | 2009.5 | 1933.2 | 1923.1 | 1909.2 | 1979 | 2250.6 | 1924.8 |
|  | Best | 1905.2 | 1927.7 | 1914.6 | 1907.3 | 1907.7 | 1982.5 | 1937.5 | 1910.3 | 1907.4 | 1906.2 | 1909.9 | 2074.8 | 1909.8 |
|  | Worst | 1909.2 | 2097.3 | 2103.4 | 1987 | 1971.5 | 2073.5 | 2093.7 | 2031 | 1985.6 | 1914.1 | 2161.3 | 2435.1 | 1983.7 |
|  | Std. | 0.94157 | 36.058 | 35.49 | 16.789 | 24.771 | 23.681 | 42.762 | 33.074 | 23.197 | 2.0817 | 71.05 | 97.409 | 23.859 |
|  | Median | 1907 | 1967.7 | 1970.8 | 1916.2 | 1912.4 | 2022.4 | 2009.1 | 1914 | 1912.7 | 1908.5 | 1967.2 | 2213.5 | 1915.5 |
|  | Rank | 1 | 9 | 8 | 3 | 5 | 12 | 11 | 7 | 4 | 2 | 10 | 13 | 6 |
| C14-F20 | Mean | 2053.9 | 1.14E+05 | 36413 | 34780 | 15176 | 44898 | 31108 | 27526 | 8068.5 | 31548 | 92553 | 1.81E+05 | 51120 |
|  | Best | 2022.7 | 16064 | 8465.6 | 6731.1 | 5575.2 | 12674 | 7547.7 | 4447.2 | 3183.8 | 8120.1 | 10781 | 51734 | 20041 |
|  | Worst | 2094 | 3.78E+05 | 1.09E+05 | 98468 | 34175 | 1.68E+05 | 85071 | 58450 | 22306 | 69338 | 6.58E+05 | 3.28E+05 | 97180 |
|  | Std. | 19.831 | 93460 | 23532 | 22117 | 7204.3 | 29911 | 17200 | 14546 | 4718.4 | 13858 | 1.19E+05 | 93156 | 21801 |
|  | Median | 2055.3 | 87319 | 31241 | 28291 | 12633 | 40851 | 29728 | 27052 | 7107.9 | 30357 | 60038 | 1.71E+05 | 50773 |
|  | Rank | 1 | 12 | 8 | 7 | 3 | 9 | 5 | 4 | 2 | 6 | 11 | 13 | 10 |

**Table S2.** (continued)

| C14-F21 | Mean | 2769.8 | 6.27E+06 | 1.95E+06 | 4.25E+05 | 1.78E+05 | 3.81E+06 | 4.20E+05 | 1.45E+05 | 1.66E+05 | 8.49E+05 | 2.05E+06 | 2.87E+07 | 2.16E+05 |
| --- | --- | --- | --- | --- | --- | --- | --- | --- | --- | --- | --- | --- | --- | --- |
|  | Best | 2505.1 | 3.39E+05 | 69121 | 36754 | 10762 | 5.21E+05 | 26691 | 16660 | 16368 | 78488 | 1.30E+05 | 2.48E+06 | 21519 |
|  | Worst | 3055.7 | 2.26E+07 | 8.73E+06 | 1.23E+06 | 5.58E+05 | 7.65E+06 | 3.87E+06 | 6.95E+05 | 5.36E+05 | 3.15E+06 | 3.07E+07 | 7.26E+07 | 5.71E+05 |
|  | Std. | 158.46 | 5.19E+06 | 2.65E+06 | 3.65E+05 | 1.48E+05 | 1.83E+06 | 7.05E+05 | 1.68E+05 | 1.25E+05 | 7.26E+05 | 5.52E+06 | 1.95E+07 | 1.70E+05 |
|  | Median | 2799.5 | 5.30E+06 | 8.31E+05 | 2.73E+05 | 1.16E+05 | 3.35E+06 | 1.57E+05 | 75933 | 1.27E+05 | 6.94E+05 | 6.48E+05 | 2.41E+07 | 1.52E+05 |
|  | Rank | 1 | 12 | 9 | 7 | 4 | 11 | 6 | 2 | 3 | 8 | 10 | 13 | 5 |
| C14-F22 | Mean | 2461.1 | 3161.2 | 2763.9 | 2794.3 | 2771.1 | 3248.9 | 3181.6 | 3219.9 | 2527.8 | 2725.8 | 3019.4 | 5989 | 3213.8 |
|  | Best | 2238.7 | 2656.3 | 2361.3 | 2495 | 2466.8 | 2696.3 | 2267.7 | 2713.8 | 2273.9 | 2327.6 | 2456 | 2873.1 | 2485 |
|  | Worst | 2627 | 3649.7 | 3240.9 | 3176.9 | 3158.8 | 3545 | 3953.3 | 3783.4 | 2834.2 | 2943.3 | 3399.9 | 56066 | 3810 |
|  | Std. | 98.962 | 247.61 | 246.77 | 177.38 | 226.41 | 201.23 | 356.67 | 297.76 | 144.57 | 132.17 | 213.19 | 9490.8 | 299.38 |
|  | Median | 2479.5 | 3146.9 | 2745.4 | 2782.9 | 2787.9 | 3295.7 | 3170.1 | 3236.2 | 2497.1 | 2735.4 | 3029.9 | 4369.3 | 3233.7 |
|  | Rank | 1 | 8 | 4 | 6 | 5 | 12 | 9 | 11 | 2 | 3 | 7 | 13 | 10 |
| C14-F23 | Mean | 2500 | 2663 | 2657.2 | 2632.7 | 2615.2 | 2706.8 | 2679.3 | 2617.4 | 2615.2 | 2615.3 | 2659 | 2500 | 2627.5 |
|  | Best | 2500 | 2500 | 2627.4 | 2622.5 | 2615.2 | 2655 | 2630.1 | 2615.2 | 2615.2 | 2615.3 | 2618.1 | 2500 | 2616.1 |
|  | Worst | 2500 | 2705.9 | 2723.6 | 2664.2 | 2615.2 | 2813.1 | 2783.6 | 2625.1 | 2615.3 | 2615.4 | 2774 | 2500 | 2648.2 |
|  | Std. | 0 | 46.58 | 23.591 | 9.3661 | 2.80E-06 | 33.998 | 35.381 | 2.6411 | 0.015676 | 0.026891 | 33.01 | 0 | 7.6786 |
|  | Median | 2500 | 2675.3 | 2650.1 | 2630.6 | 2615.2 | 2699.2 | 2666.9 | 2616 | 2615.2 | 2615.3 | 2649.3 | 2500 | 2626.7 |
|  | Rank | 1 | 11 | 9 | 8 | 3 | 13 | 12 | 6 | 4 | 5 | 10 | 2 | 7 |
| C14-F24 | Mean | 2600 | 2608.2 | 2600 | 2643.5 | 2630.9 | 2610.8 | 2774.7 | 2651.2 | 2600 | 2630.5 | 2669 | 2600.1 | 2727.9 |
|  | Best | 2600 | 2600 | 2600 | 2627.4 | 2623.6 | 2600.8 | 2677 | 2624.9 | 2600 | 2625.6 | 2637 | 2600 | 2655 |
|  | Worst | 2600 | 2618.9 | 2600.1 | 2654.7 | 2646.4 | 2665.5 | 2913.7 | 2803.1 | 2600 | 2633.9 | 2760 | 2600.2 | 2956.3 |
|  | Std. | 0.0021742 | 4.9851 | 0.011507 | 7.164 | 6.7341 | 14.783 | 75.514 | 31.173 | 0.0033973 | 1.9843 | 32.899 | 0.08832 | 96.848 |
|  | Median | 2600 | 2607.5 | 2600 | 2645 | 2628.3 | 2604 | 2751.6 | 2645 | 2600 | 2630.7 | 2658.5 | 2600.1 | 2675.5 |
|  | Rank | 1 | 5 | 3 | 9 | 8 | 6 | 13 | 10 | 2 | 7 | 11 | 4 | 12 |
| C14-F25 | Mean | 2700 | 2709.1 | 2711.4 | 2717.7 | 2715.7 | 2737.3 | 2744.7 | 2725.6 | 2700.5 | 2723.8 | 2715 | 2700.3 | 2730.1 |
|  | Best | 2700 | 2700 | 2700 | 2712 | 2708.6 | 2712.5 | 2720.1 | 2704.9 | 2700 | 2713.7 | 2704.9 | 2700 | 2708.3 |
|  | Worst | 2700 | 2751.6 | 2722.7 | 2728.2 | 2723 | 2760.6 | 2780.9 | 2773.1 | 2709.2 | 2740.1 | 2741.5 | 2705.6 | 2764.8 |
|  | Std. | 0 | 15.199 | 8.5505 | 4.1419 | 3.89 | 10.07 | 17.458 | 17.941 | 1.8333 | 7.1064 | 7.1575 | 1.2664 | 15.204 |
|  | Median | 2700 | 2700 | 2715.2 | 2716.3 | 2715.3 | 2735.3 | 2741.2 | 2721.8 | 2700 | 2723.3 | 2714 | 2700 | 2730.1 |
|  | Rank | 1 | 4 | 5 | 8 | 7 | 12 | 13 | 10 | 3 | 9 | 6 | 2 | 11 |
| C14-F26 | Mean | 2700.6 | 2713.8 | 2759.4 | 2726.9 | 2776.9 | 2703.6 | 2765.6 | 2774.2 | 2730.4 | 2700.7 | 2702.4 | 2773 | 2796.2 |
|  | Best | 2700.4 | 2700.3 | 2700.6 | 2700.2 | 2700.4 | 2703 | 2700.3 | 2700.5 | 2700.3 | 2700.4 | 2700.6 | 2705.2 | 2700.4 |
|  | Worst | 2700.9 | 2800 | 2967.2 | 2800 | 2800.2 | 2704.6 | 3001.8 | 2971.7 | 2800.1 | 2700.8 | 2704.4 | 2800 | 2987.9 |
|  | Std. | 0.099645 | 34.385 | 63.233 | 44.839 | 42.835 | 0.44513 | 80.089 | 70.638 | 46.394 | 0.10928 | 1.1954 | 42.003 | 63.977 |
|  | Median | 2700.6 | 2700.5 | 2800 | 2700.3 | 2800.1 | 2703.5 | 2752.9 | 2800.1 | 2700.5 | 2700.7 | 2702.7 | 2800 | 2800.3 |
|  | Rank | 1 | 5 | 8 | 6 | 12 | 4 | 9 | 11 | 7 | 2 | 3 | 10 | 13 |
| C14-F27 | Mean | 3103 | 3883.6 | 3566.2 | 3540.6 | 3396.7 | 3759.1 | 3988.6 | 3581 | 3398.5 | 3198.7 | 3627.3 | 3937.8 | 3898.1 |
|  | Best | 3101.6 | 3174.4 | 3122.1 | 3107.1 | 3101.4 | 3244.3 | 3124.3 | 3102.1 | 3103.5 | 3135 | 3148.8 | 2900 | 3103.5 |
|  | Worst | 3106.4 | 4262.8 | 3791.9 | 3784.4 | 3753.8 | 4103.9 | 4394.7 | 4143 | 3775.4 | 3282 | 3811.4 | 4383 | 4323.4 |
|  | Std. | 1.1678 | 345.8 | 231.73 | 169.22 | 230.13 | 314.96 | 397.49 | 407.54 | 221.35 | 40.056 | 198.16 | 426.96 | 414.83 |
|  | Median | 3102.8 | 4018.9 | 3658.4 | 3578.7 | 3474.8 | 3919.4 | 4116.5 | 3774.8 | 3498 | 3191.2 | 3707.3 | 4113.5 | 4092.6 |
|  | Rank | 1 | 10 | 6 | 5 | 3 | 9 | 13 | 7 | 4 | 2 | 8 | 12 | 11 |

**Table S2.** (continued)

| C14-F28 | Mean | 3000 | 5560 | 4953.9 | 4082.8 | 5960.9 | 5535.3 | 7581.6 | 6694.6 | 4202.9 | 3652.4 | 3890.6 | 4272.7 | 8020.1 |
| --- | --- | --- | --- | --- | --- | --- | --- | --- | --- | --- | --- | --- | --- | --- |
|  | Best | 3000 | 3000 | 4085.7 | 3770.3 | 3652 | 4739.6 | 6200.7 | 5267.5 | 3724.8 | 3597.5 | 3748.7 | 3000 | 6440.9 |
|  | Worst | 3000 | 7678.2 | 6371.4 | 4957.5 | 8405.5 | 6891.1 | 8820.4 | 8604.4 | 5132.1 | 3694.1 | 4430.5 | 9288.6 | 9674 |
|  | Std. | 0 | 780.17 | 672.68 | 320.12 | 1113 | 531.33 | 710.06 | 748.23 | 419.02 | 20.69 | 138.77 | 2360.7 | 907.1 |
|  | Median | 3000 | 5587.3 | 4936.5 | 3928.2 | 5803.9 | 5396.5 | 7579 | 6664.8 | 4039.8 | 3654.1 | 3844.9 | 3000 | 8003.2 |
|  | Rank | 1 | 9 | 7 | 4 | 10 | 8 | 12 | 11 | 5 | 2 | 3 | 6 | 13 |
| C14-F29 | Mean | 4112.2 | 1.20E+07 | 9.66E+06 | 6.20E+06 | 6.32E+05 | 3.24E+07 | 2.47E+07 | 1.68E+06 | 4.70E+06 | 1.69E+06 | 2.38E+06 | 3.89E+08 | 1.19E+07 |
|  | Best | 3713.5 | 4.05E+05 | 10528 | 8328.4 | 3584.9 | 1.03E+07 | 4203.7 | 4423.4 | 4220.3 | 4300.2 | 7835.8 | 3100 | 5481.2 |
|  | Worst | 4840.6 | 2.41E+07 | 4.94E+07 | 4.64E+07 | 9.83E+06 | 5.59E+07 | 1.33E+08 | 1.60E+07 | 1.40E+07 | 8.65E+06 | 9.61E+06 | 9.40E+08 | 5.92E+07 |
|  | Std. | 236.52 | 5.56E+06 | 1.21E+07 | 1.18E+07 | 2.39E+06 | 1.34E+07 | 3.50E+07 | 4.41E+06 | 5.37E+06 | 3.43E+06 | 3.73E+06 | 2.59E+08 | 2.00E+07 |
|  | Median | 4074.1 | 1.15E+07 | 5.22E+06 | 29953 | 4076.8 | 3.15E+07 | 8.00E+06 | 12055 | 31019 | 5769.9 | 54831 | 4.23E+08 | 27472 |
|  | Rank | 1 | 10 | 8 | 7 | 2 | 12 | 11 | 3 | 6 | 4 | 5 | 13 | 9 |
| C14-F30 | Mean | 5575 | 3.19E+05 | 1.65E+05 | 38434 | 5629.2 | 5.51E+05 | 1.76E+05 | 11838 | 8106.4 | 6645.7 | 45719 | 5.49E+06 | 29534 |
|  | Best | 4151.4 | 1.22E+05 | 19476 | 16463 | 4517.4 | 2.29E+05 | 13778 | 5356 | 4872.4 | 4320.2 | 7350.7 | 3200 | 7437.9 |
|  | Worst | 11146 | 9.21E+05 | 5.10E+05 | 1.08E+05 | 7894.1 | 9.71E+05 | 4.94E+05 | 1.21E+05 | 26466 | 7855.8 | 1.44E+05 | 1.76E+07 | 1.32E+05 |
|  | Std. | 1493.4 | 2.02E+05 | 1.32E+05 | 18185 | 924.12 | 1.52E+05 | 1.36E+05 | 20799 | 4715.5 | 832.19 | 41165 | 4.30E+06 | 25265 |
|  | Median | 4986 | 2.62E+05 | 1.07E+05 | 33066 | 5430.5 | 5.38E+05 | 1.35E+05 | 7274.4 | 6128.7 | 6687.2 | 26578 | 4.53E+06 | 20526 |
|  | Rank | 1 | 11 | 9 | 7 | 2 | 12 | 10 | 5 | 4 | 3 | 8 | 13 | 6 |
| Sum rank | | 39 | 266 | 226 | 171 | 117 | 324 | 271 | 194 | 133 | 196 | 240 | 323 | 230 |
| Mean rank | | 1.3000 | 8.8666 | 7.5333 | 5.7000 | 3.9000 | 10.8000 | 9.0333 | 6.4666 | 4.4333 | 6.5333 | 8 | 10.7666 | 7.6666 |
| Total rank | | 1 | 10 | 7 | 4 | 2 | 13 | 11 | 5 | 3 | 6 | 9 | 12 | 8 |

**Table S2.** (continued)

| 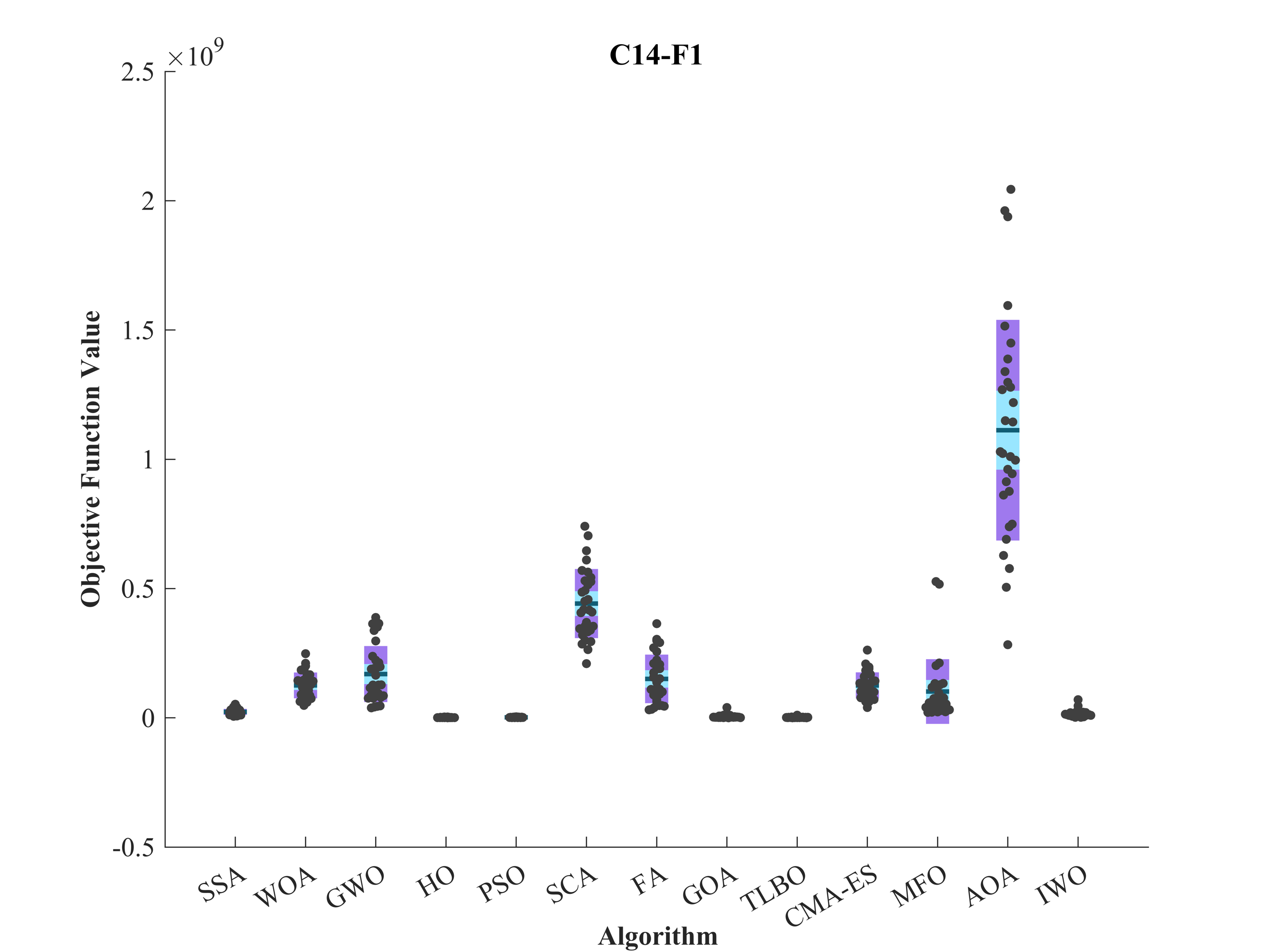 | 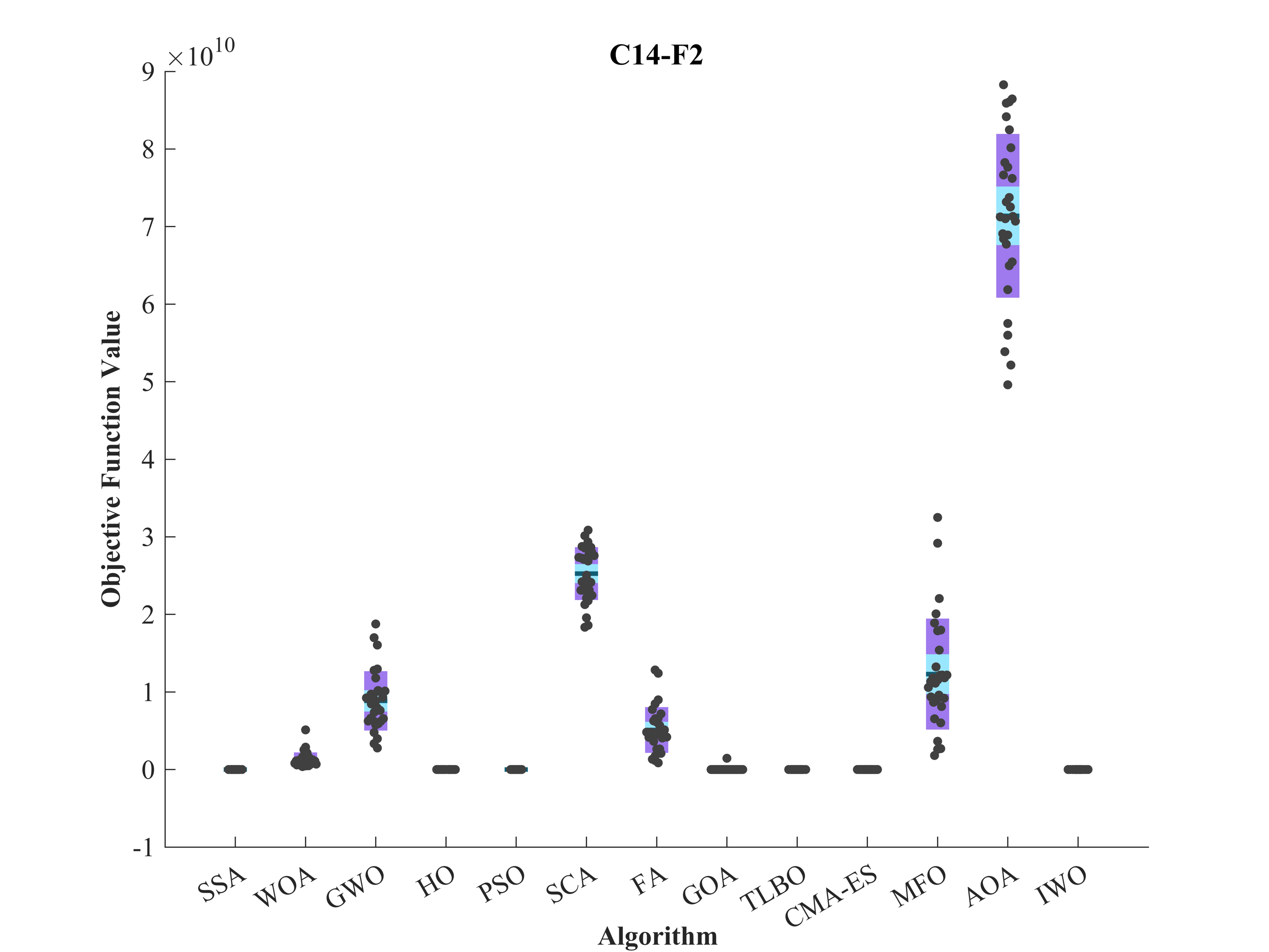 |
| --- | --- |
| 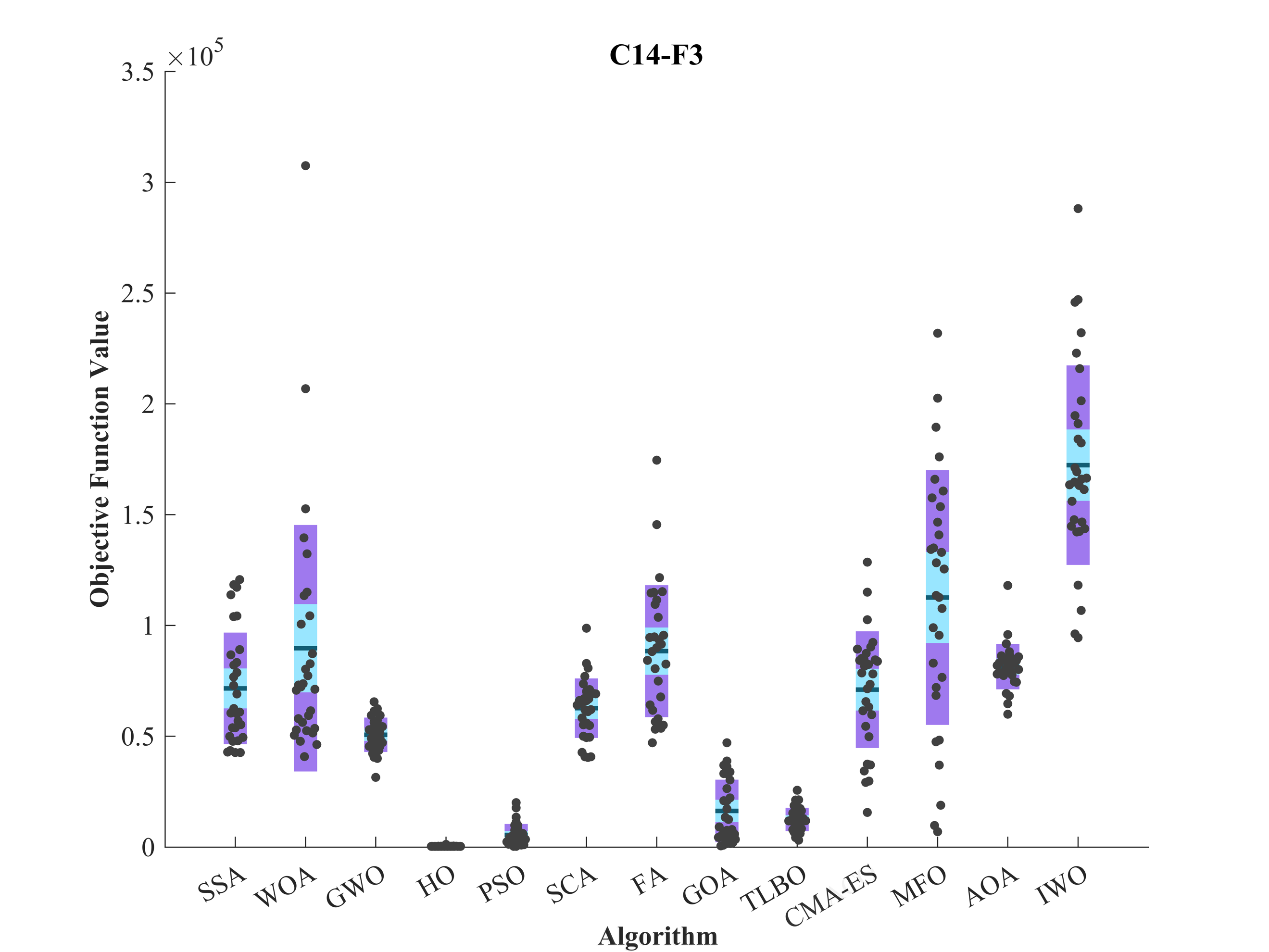 | 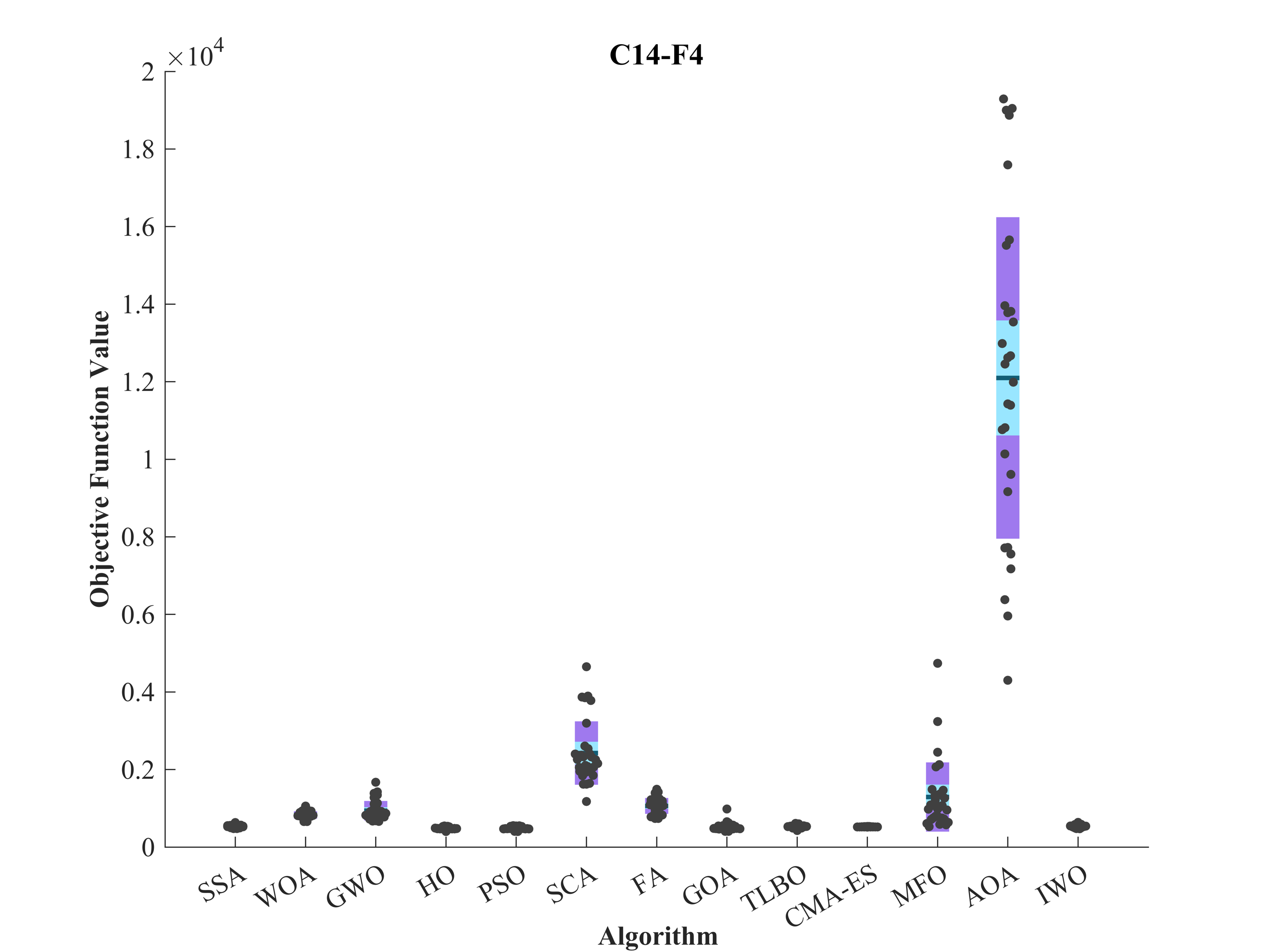 |
| 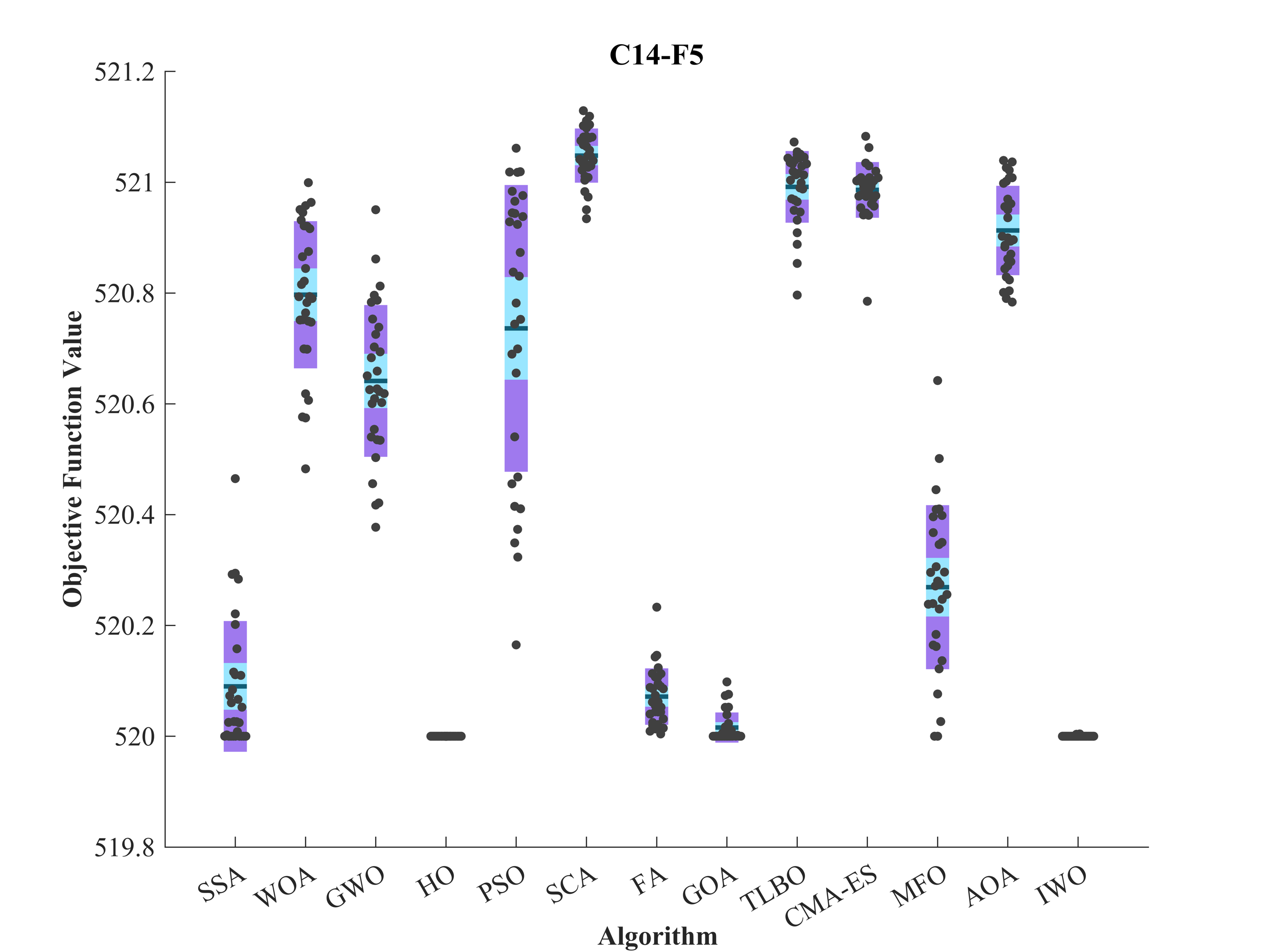 | 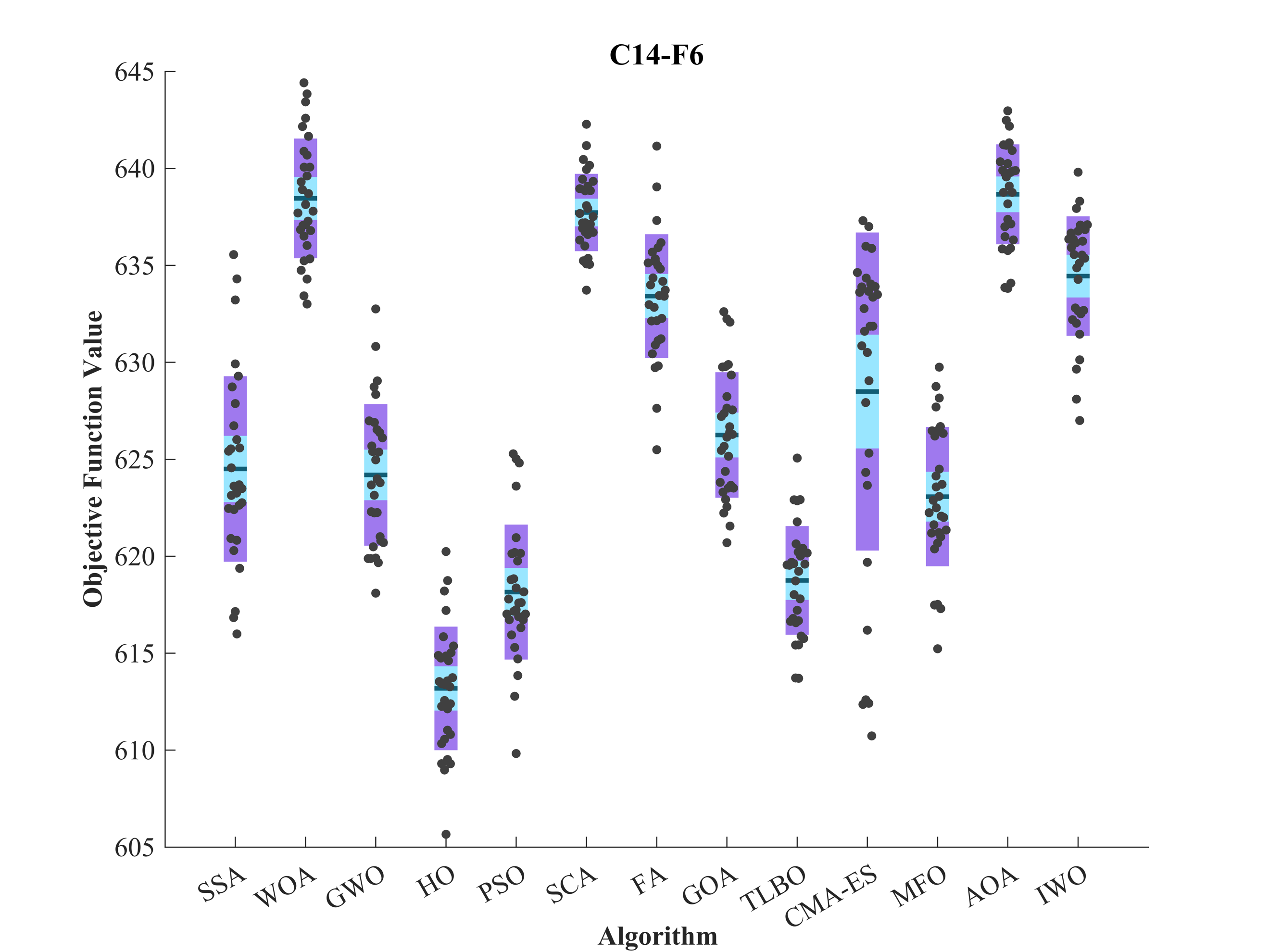 |
| 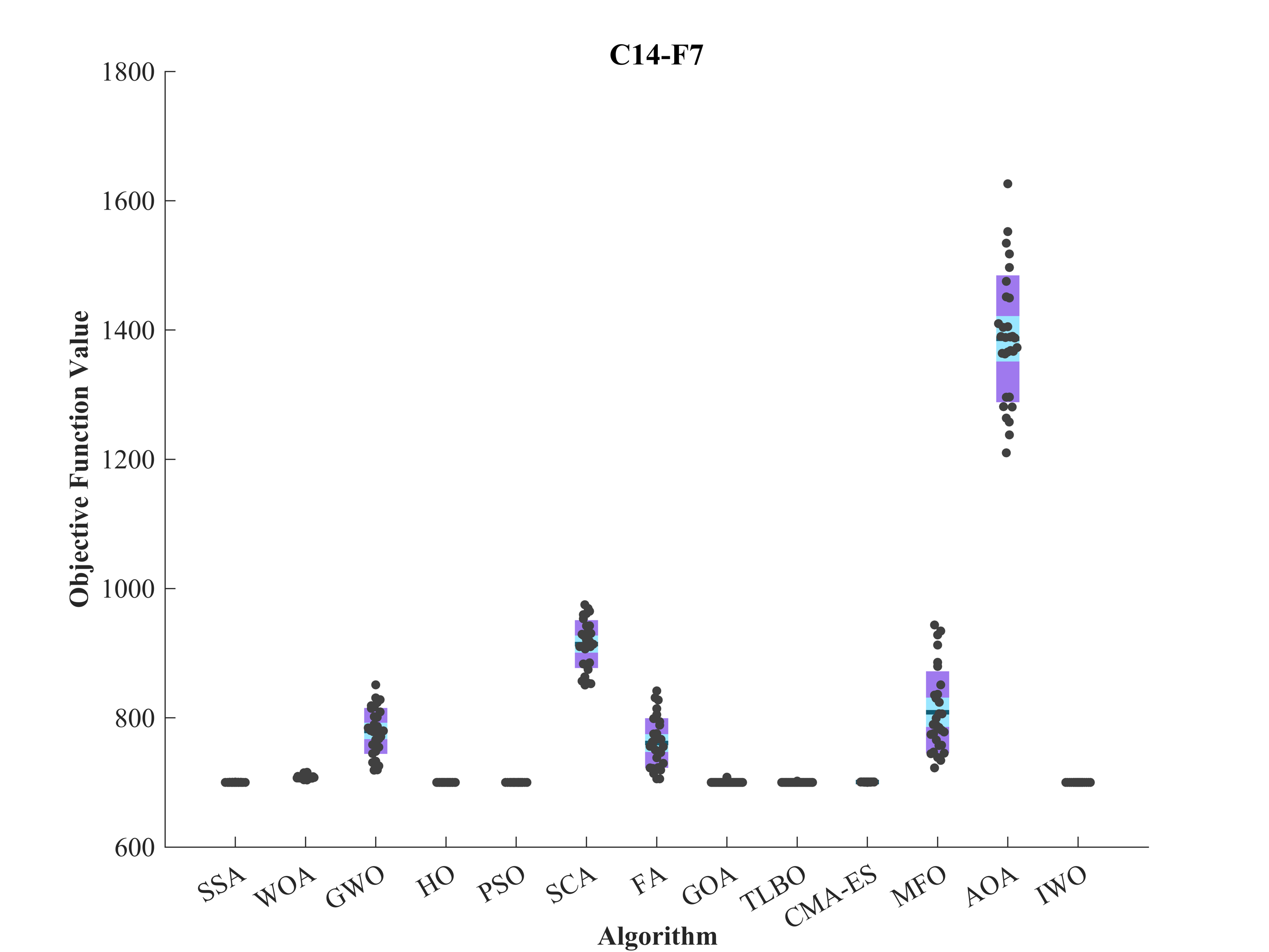 | 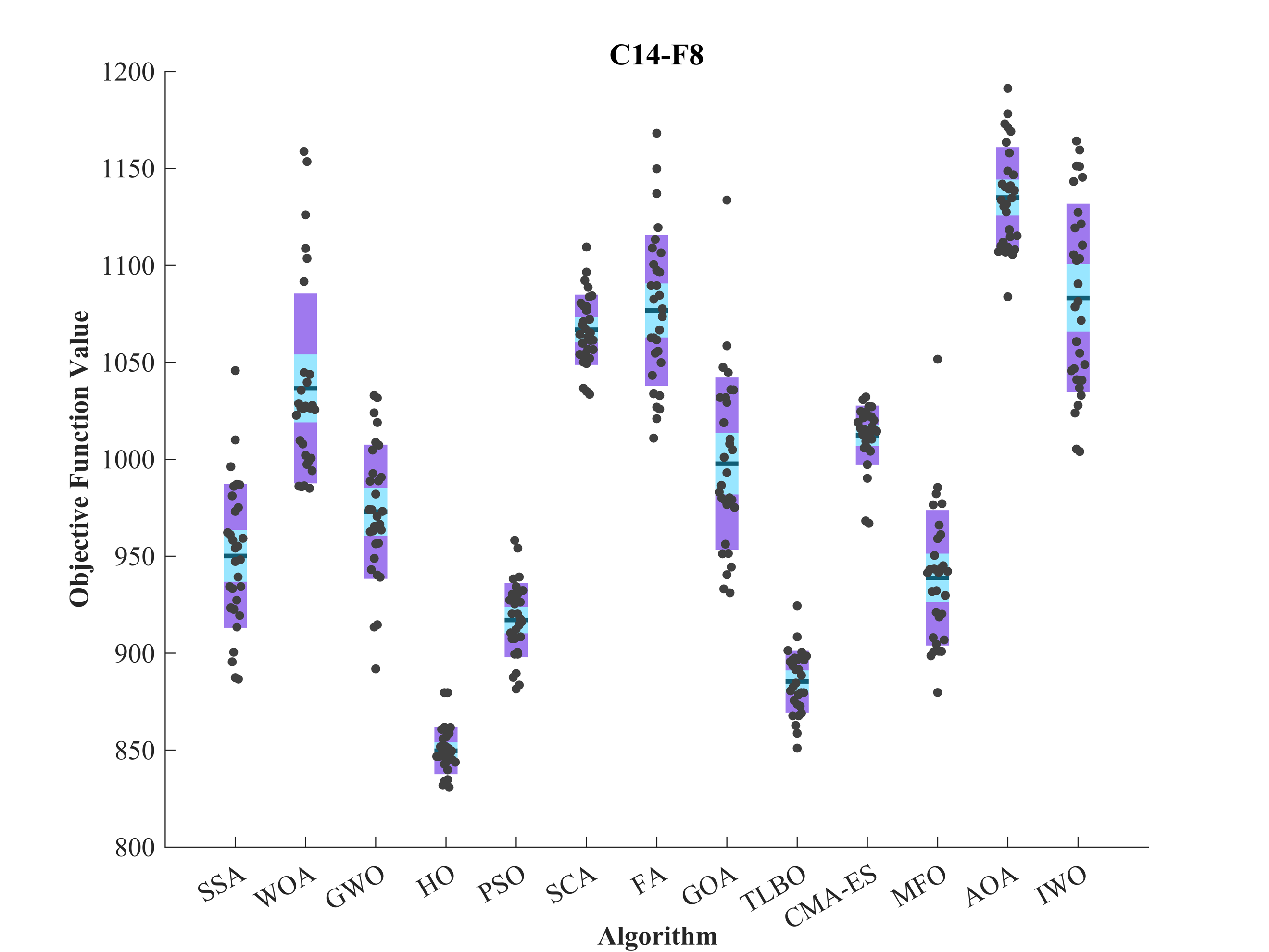 |

**Figure S4.** Boxplot illustrating the performance of the HO in comparison to competing algorithms for optimizing CEC 2014 (D = 30).

| 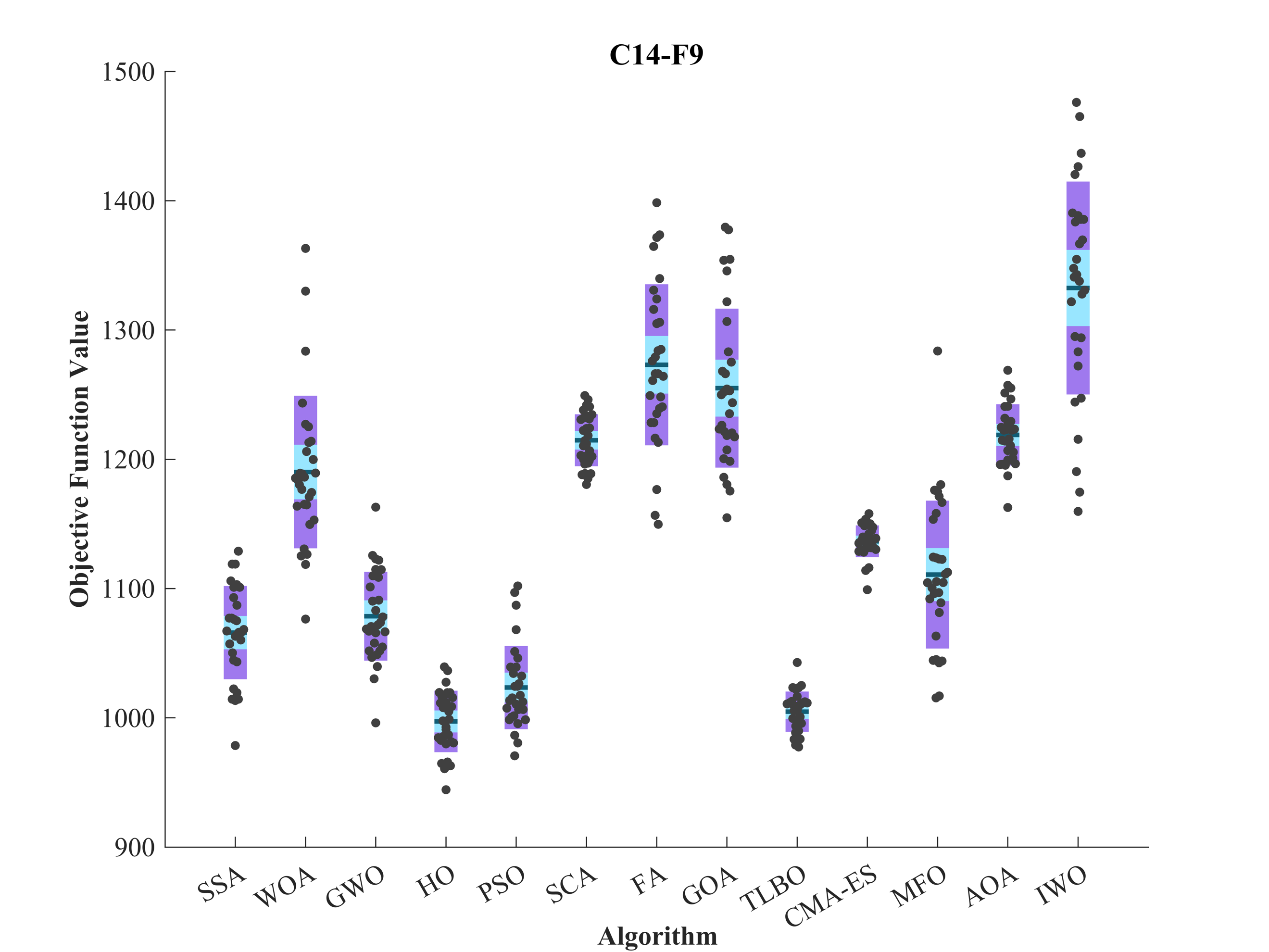 | 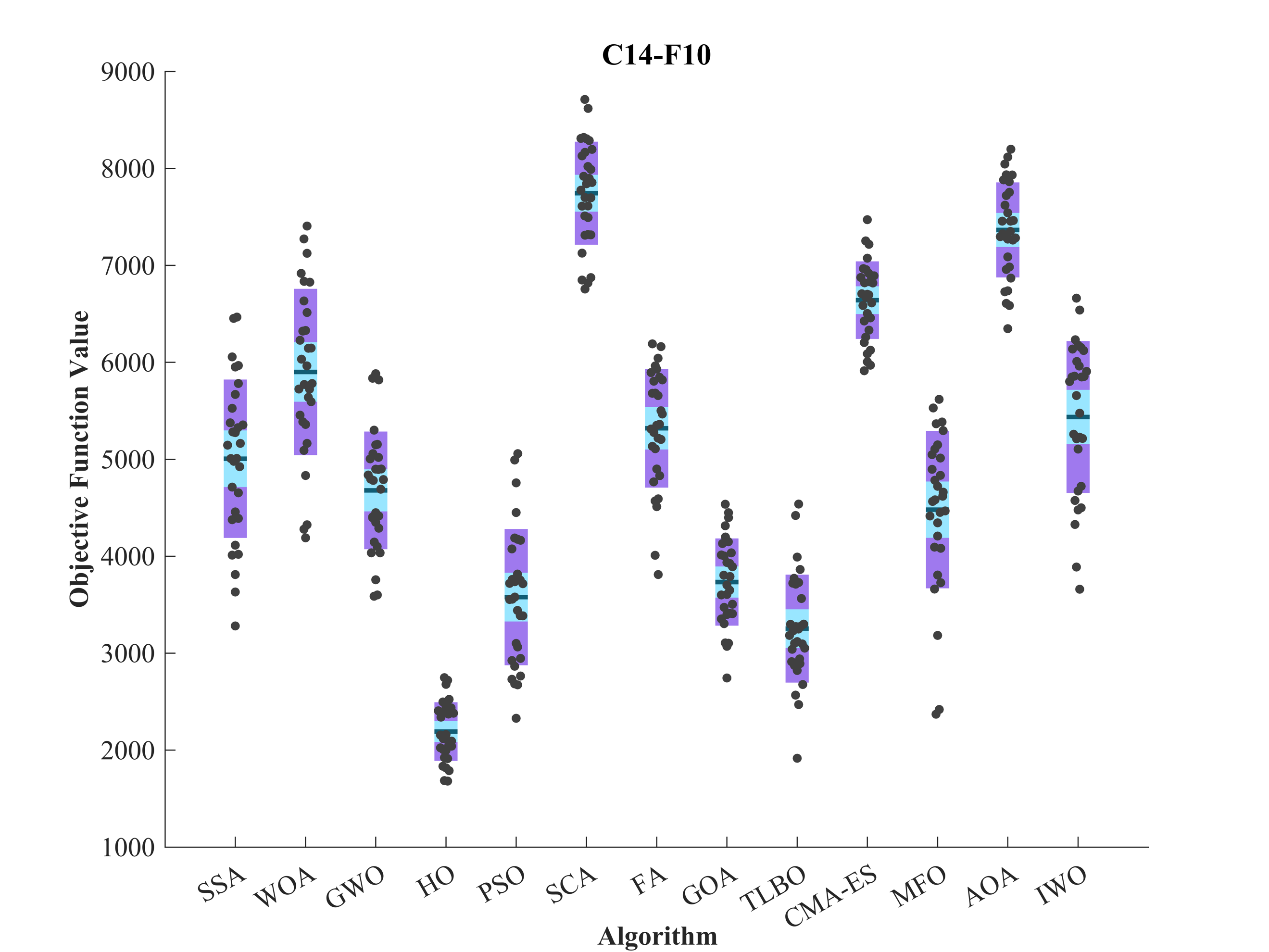 |
| --- | --- |
| 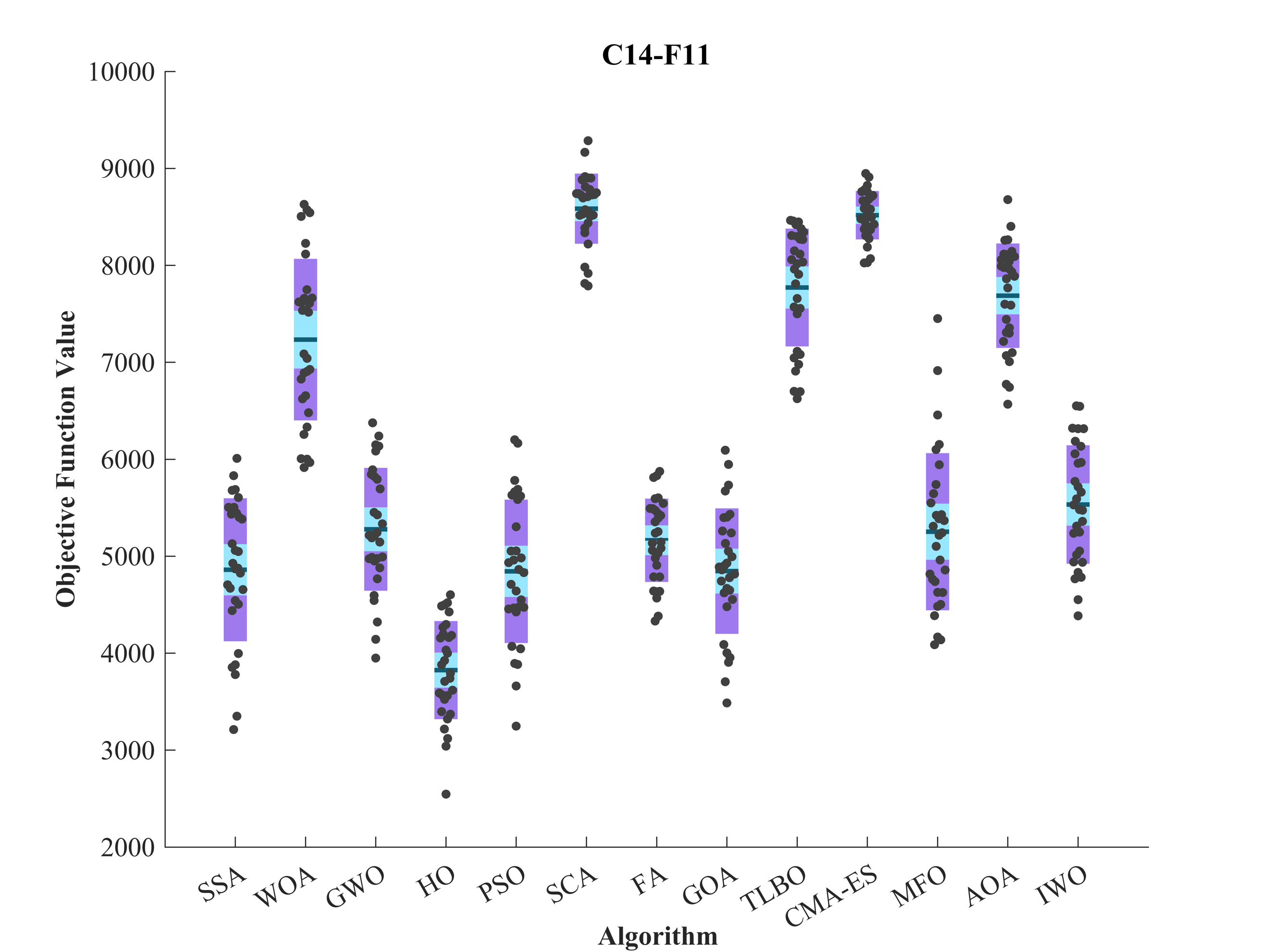 | 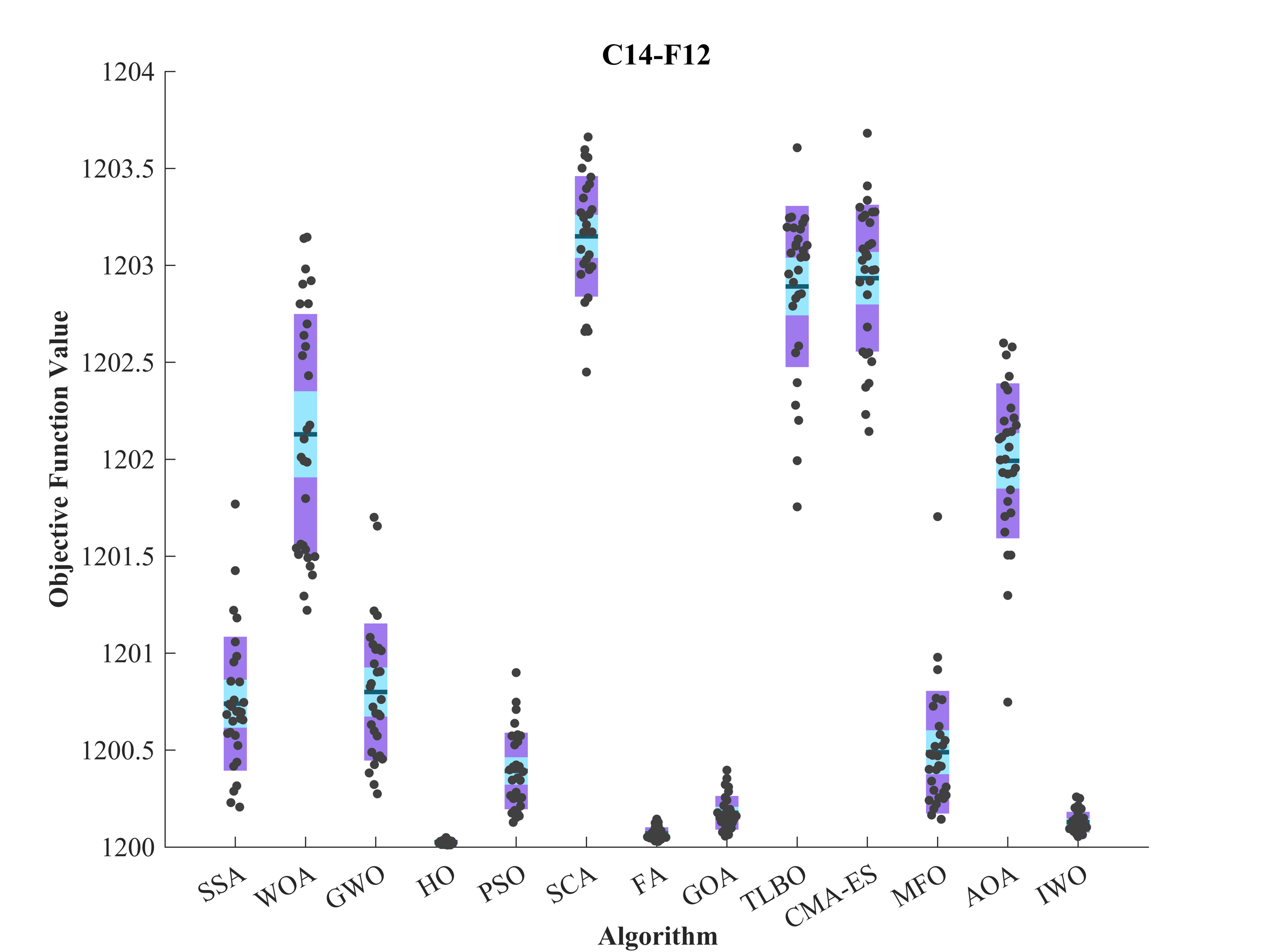 |
| 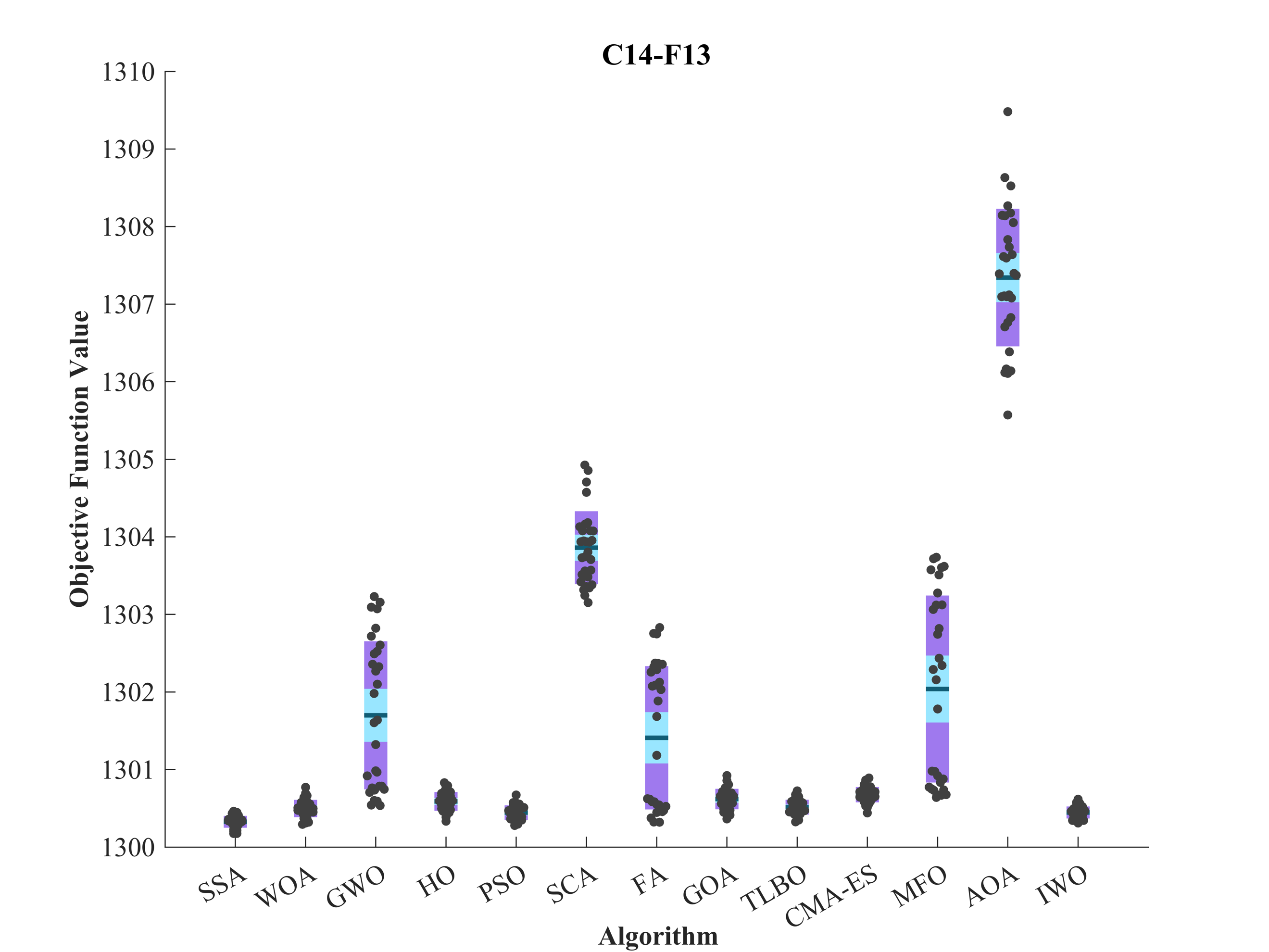 | 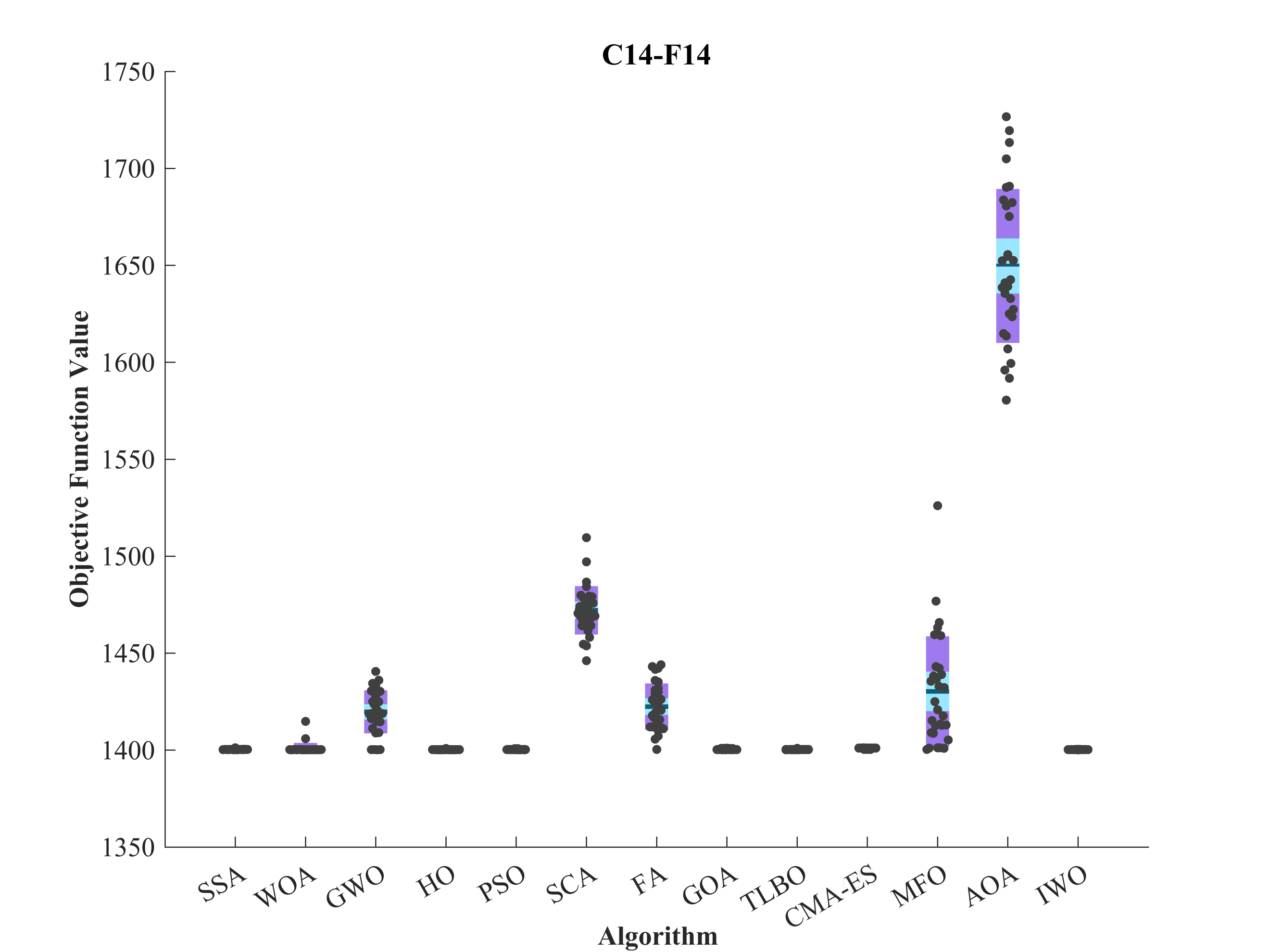 |
| 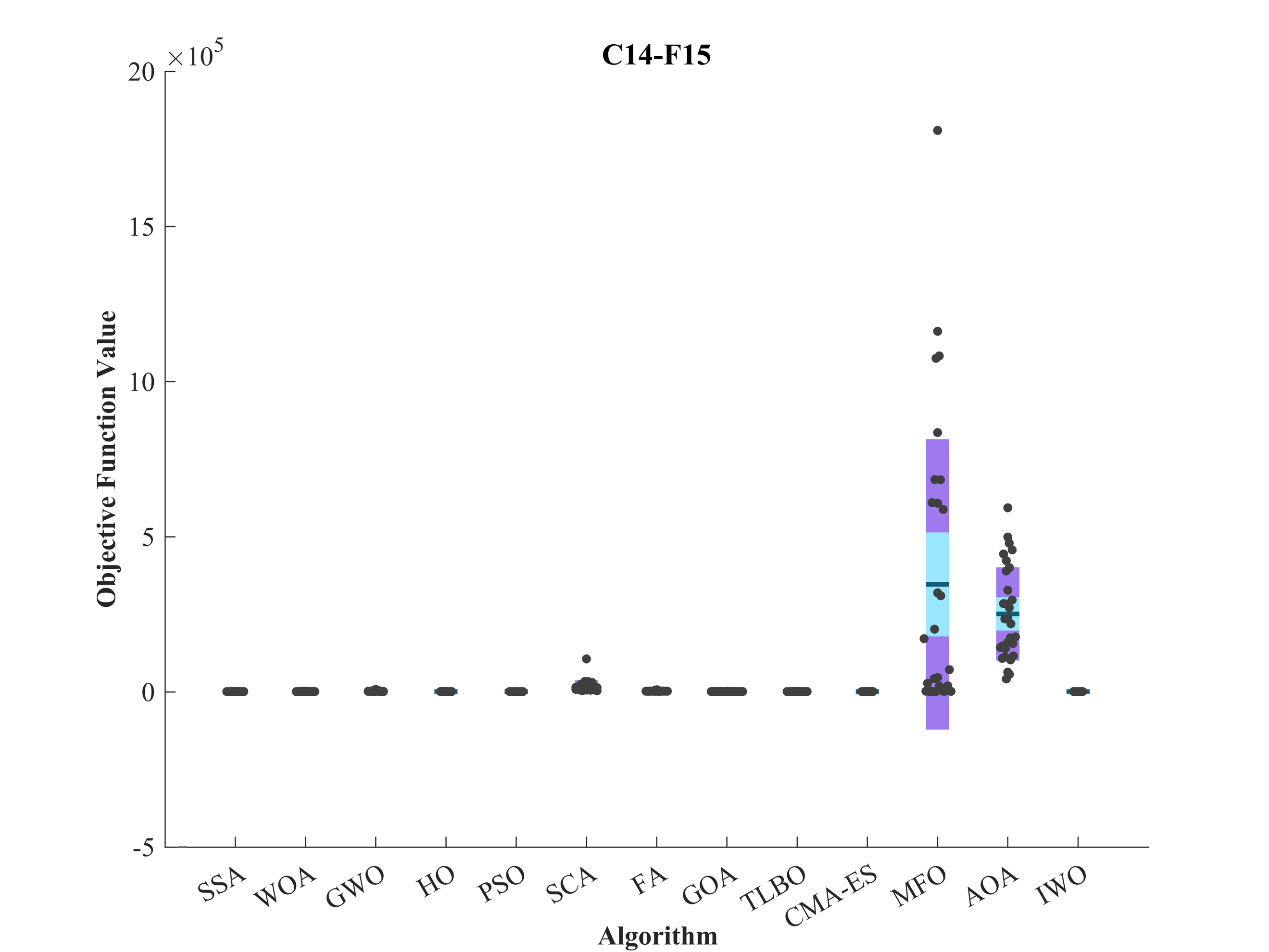 | 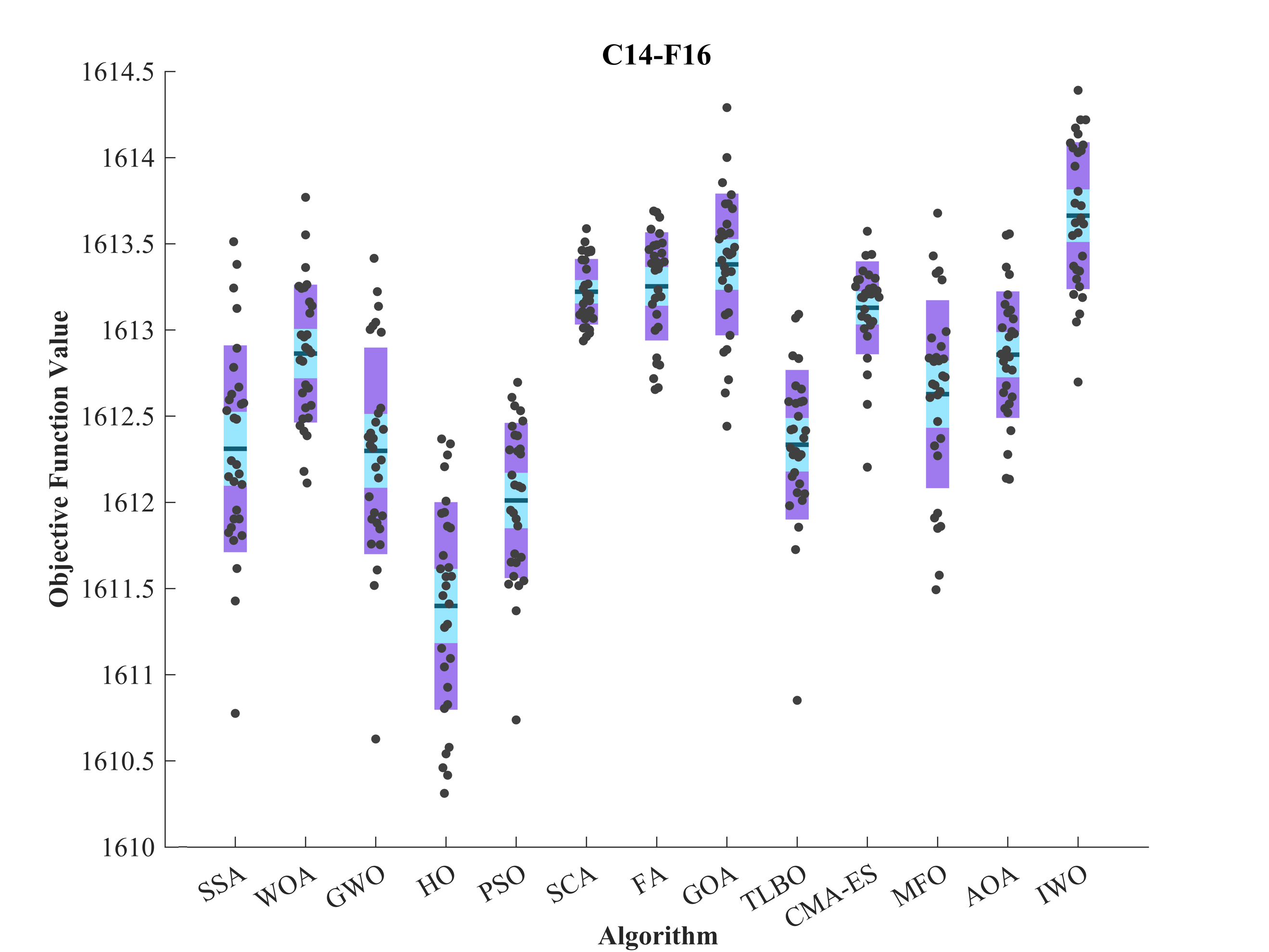 |

**Figure S4.** (continued)

| 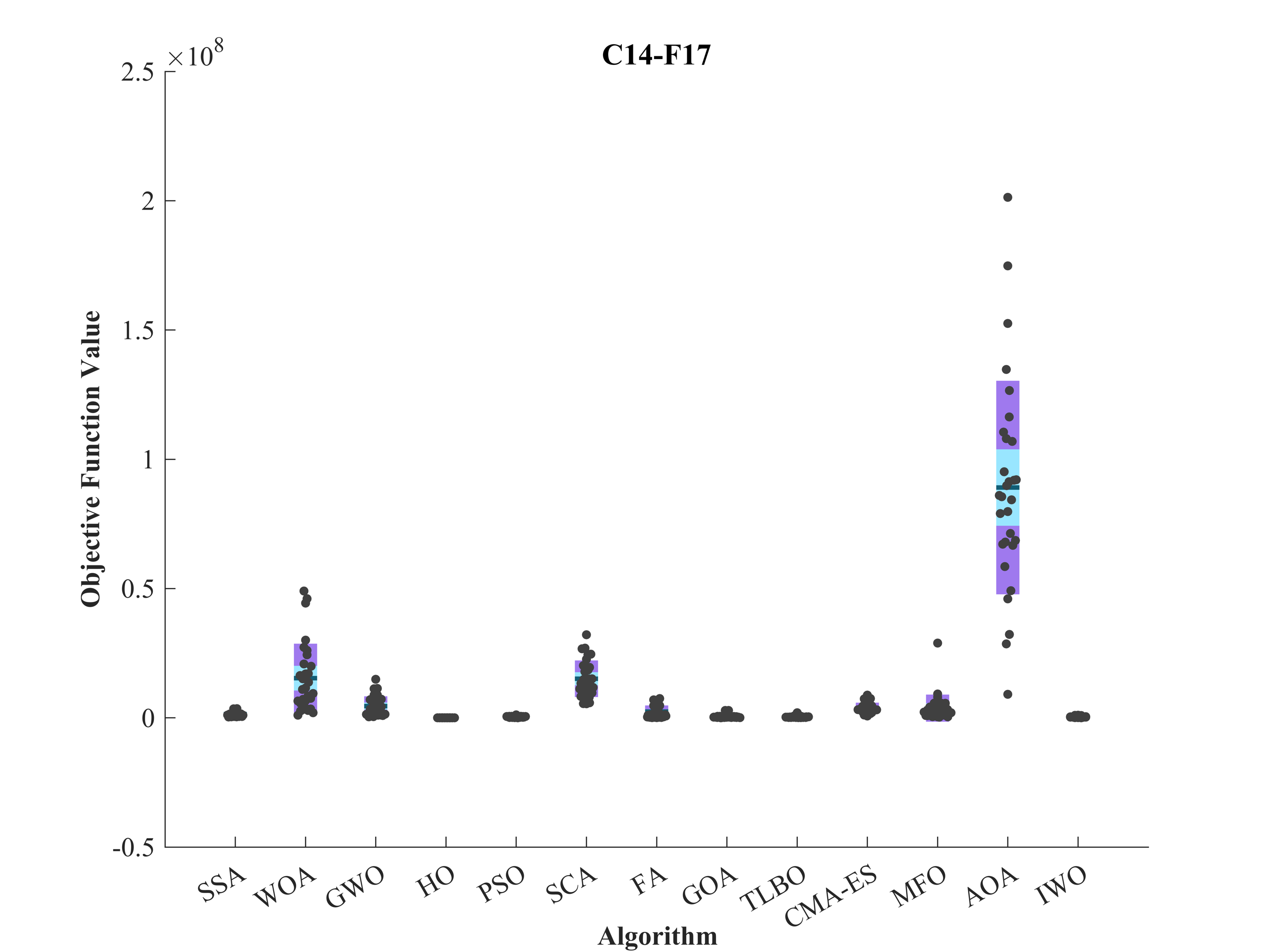 | 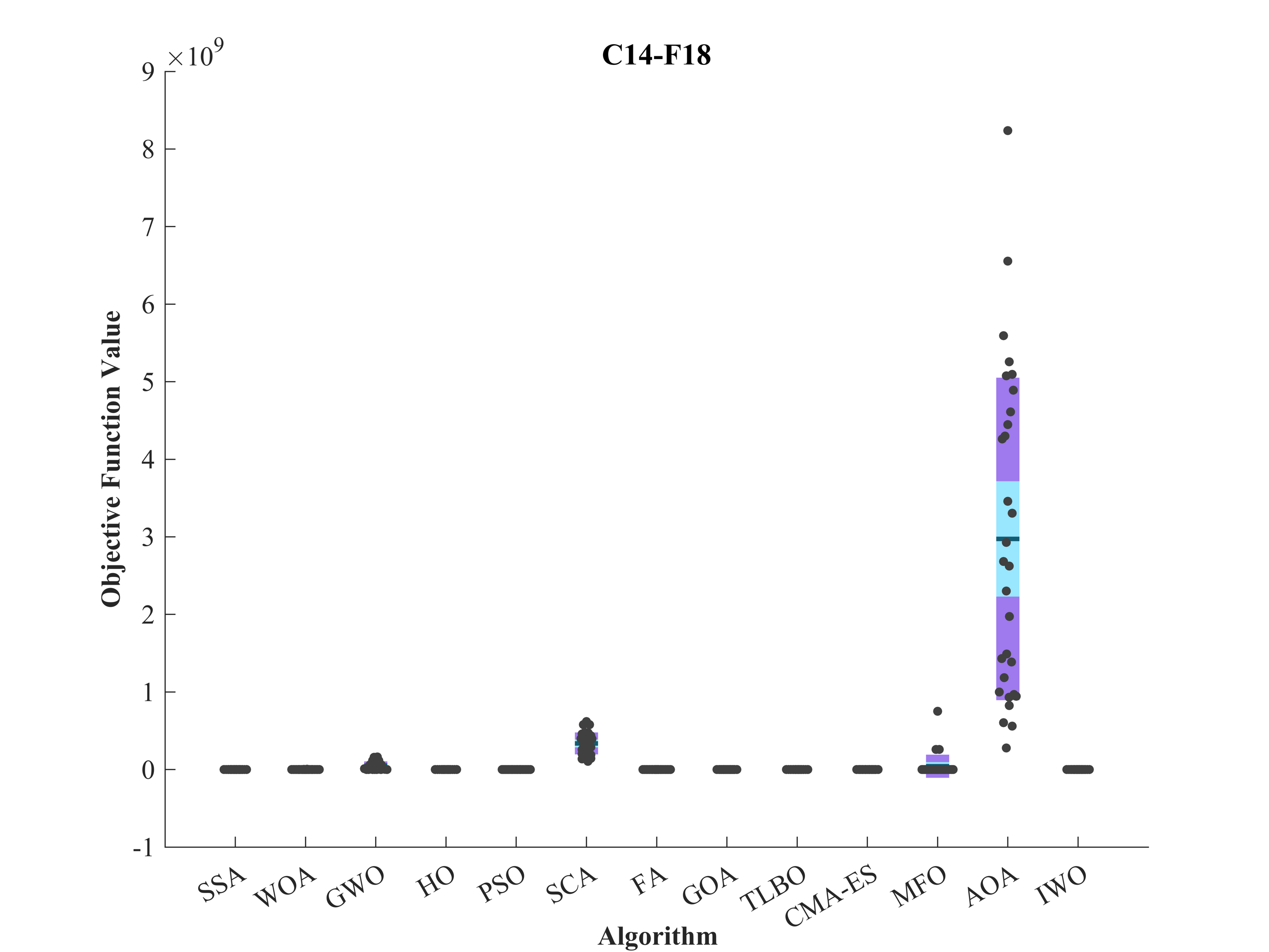 |
| --- | --- |
| 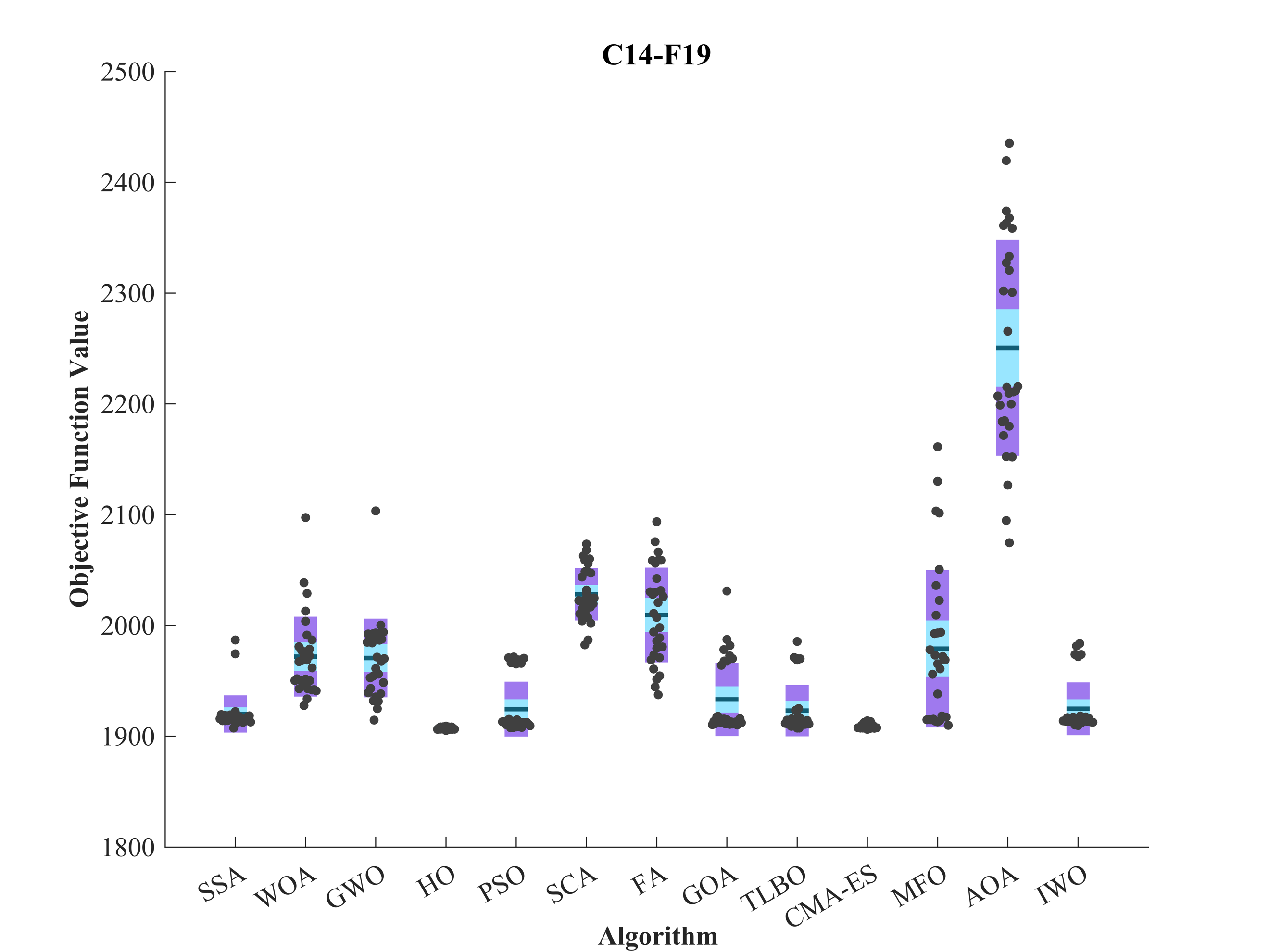 | 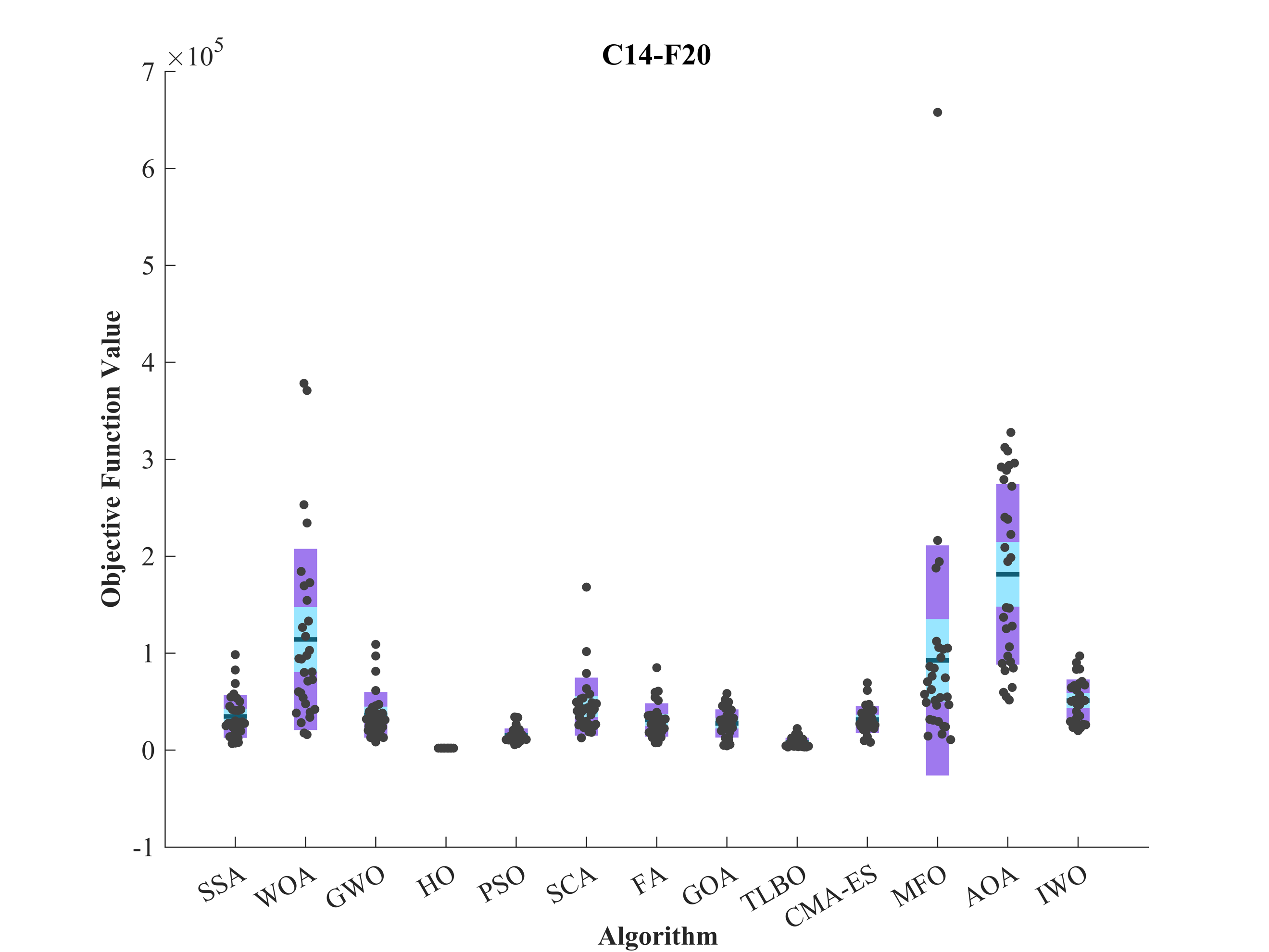 |
| 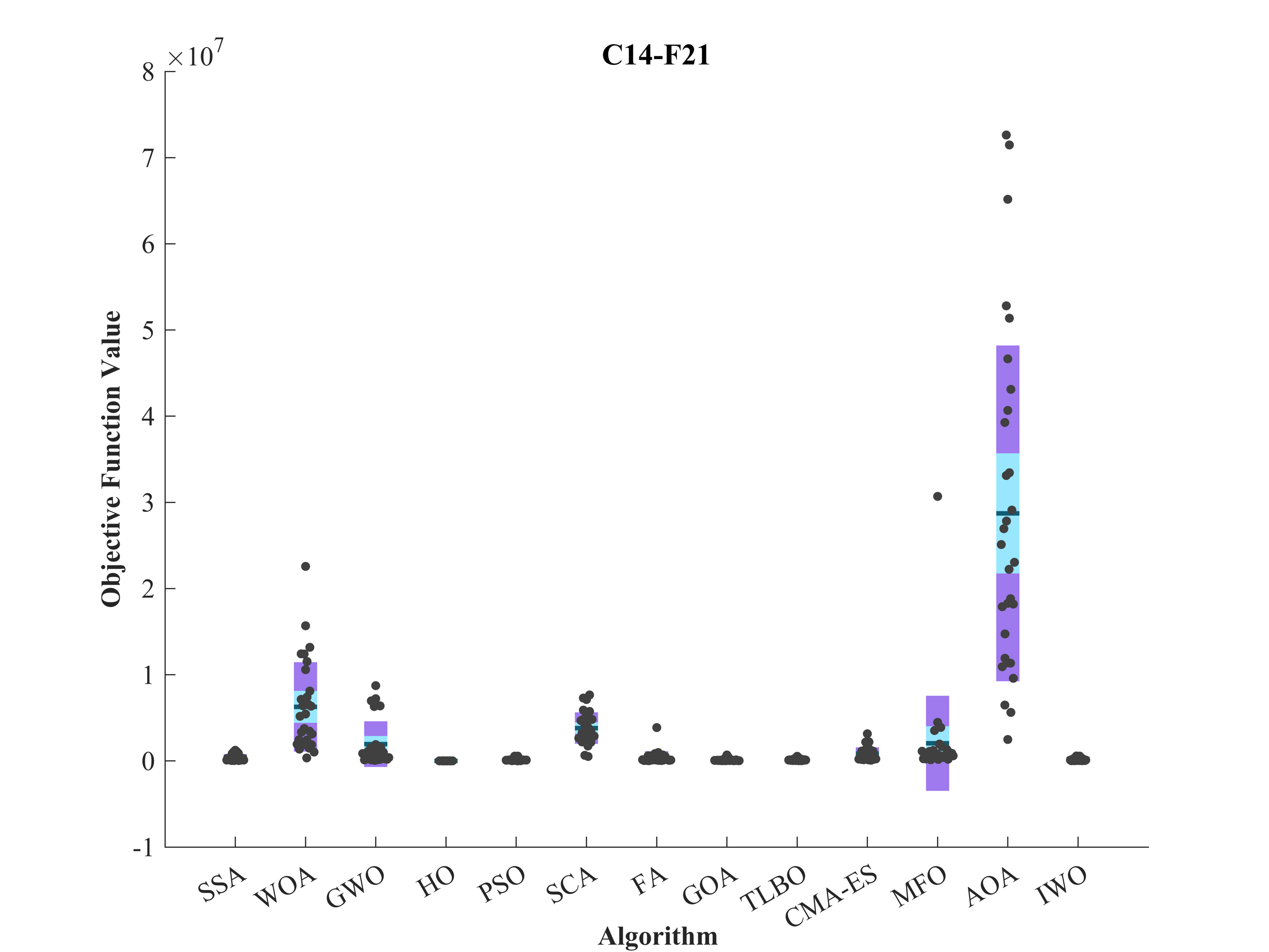 | 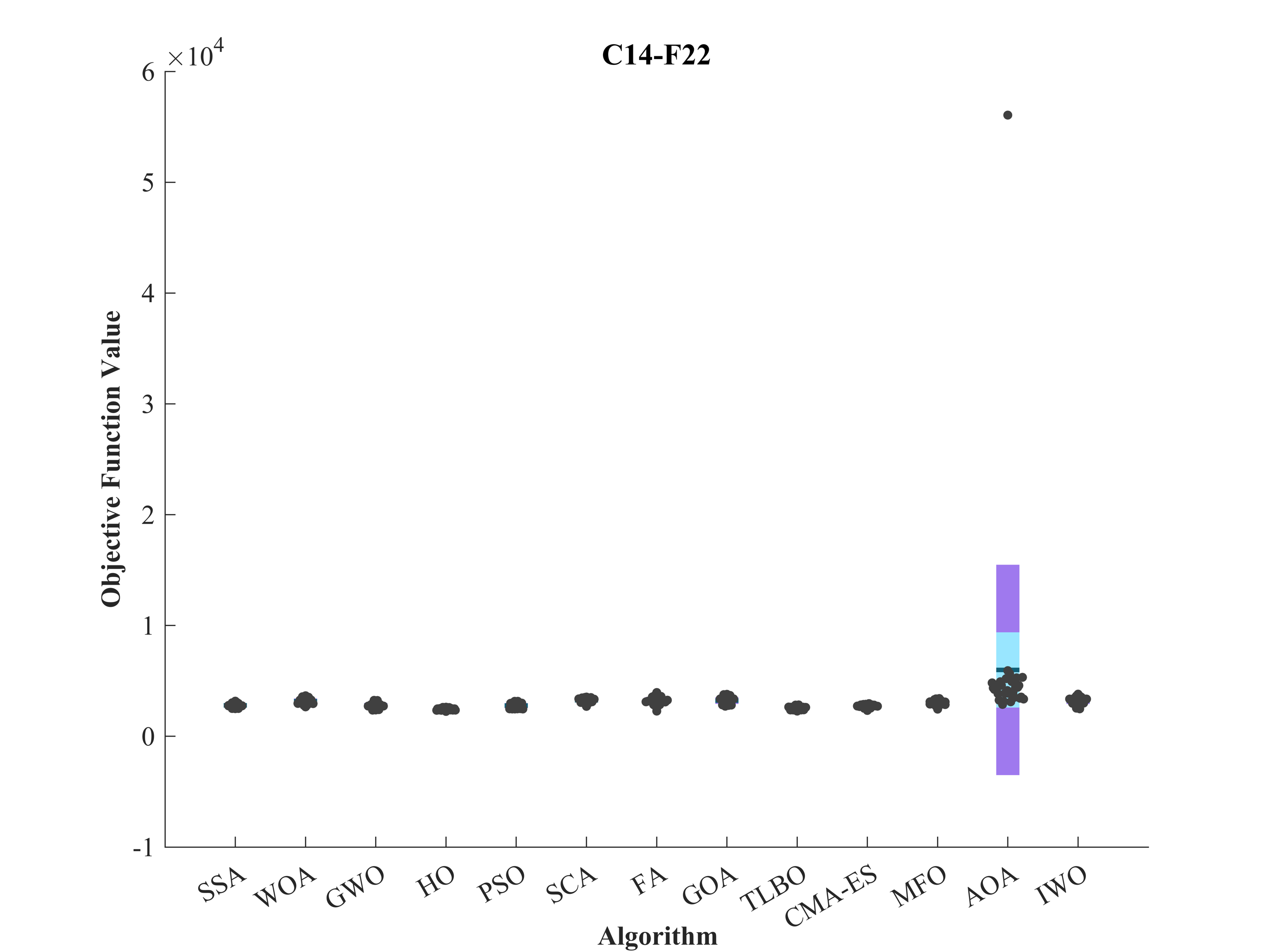 |
| 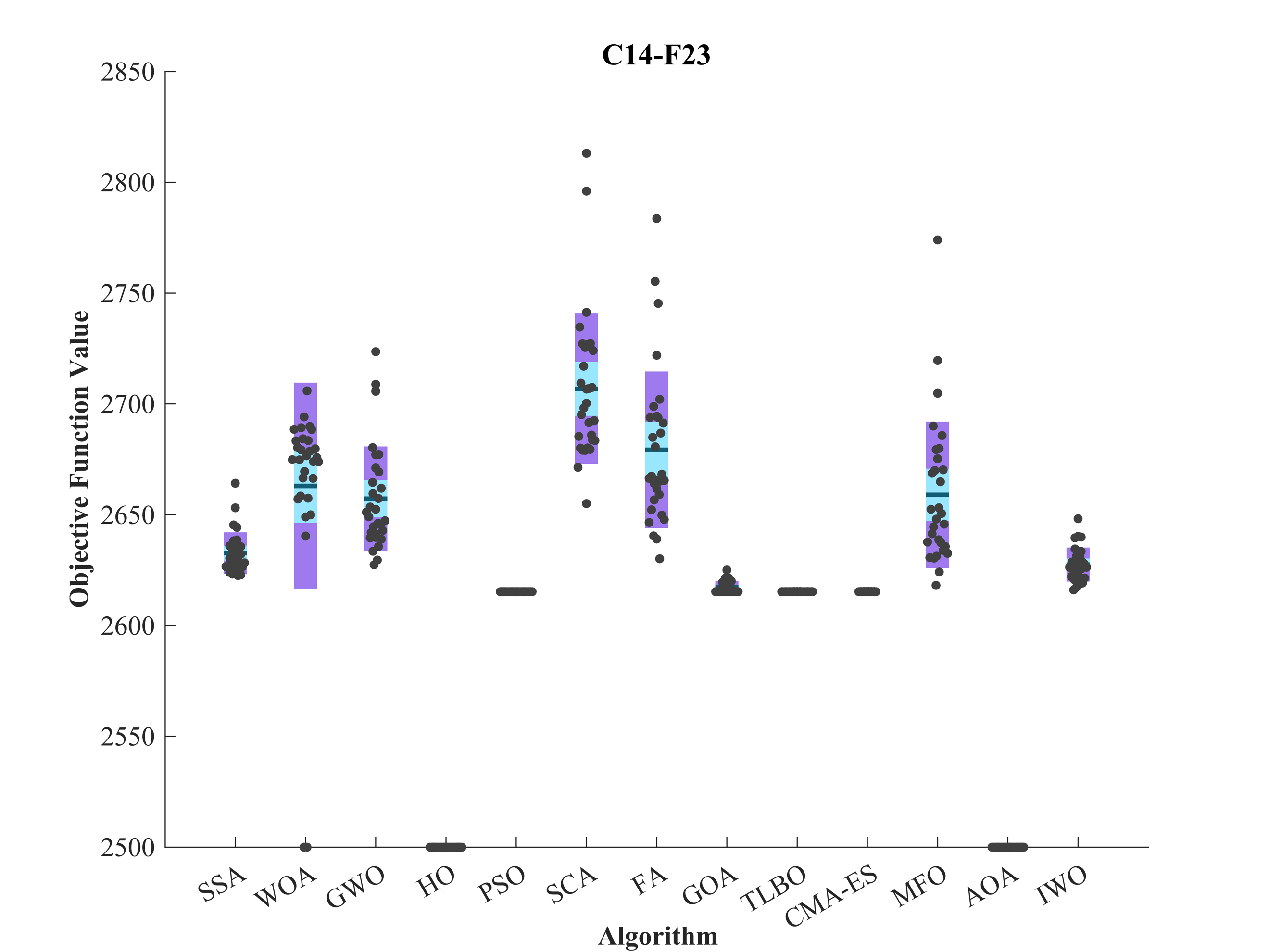 | 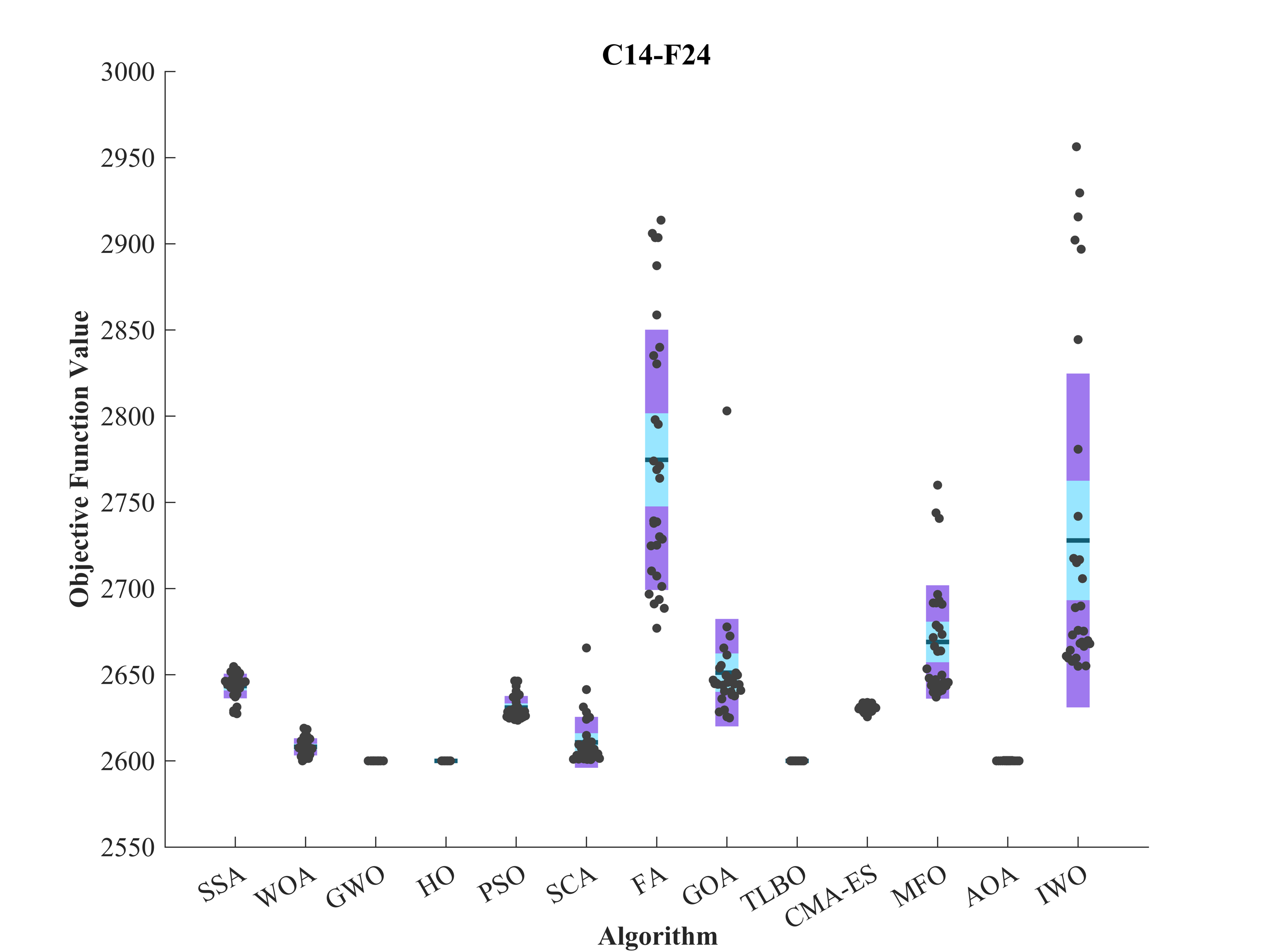 |

**Figure S4.** (continued)

| 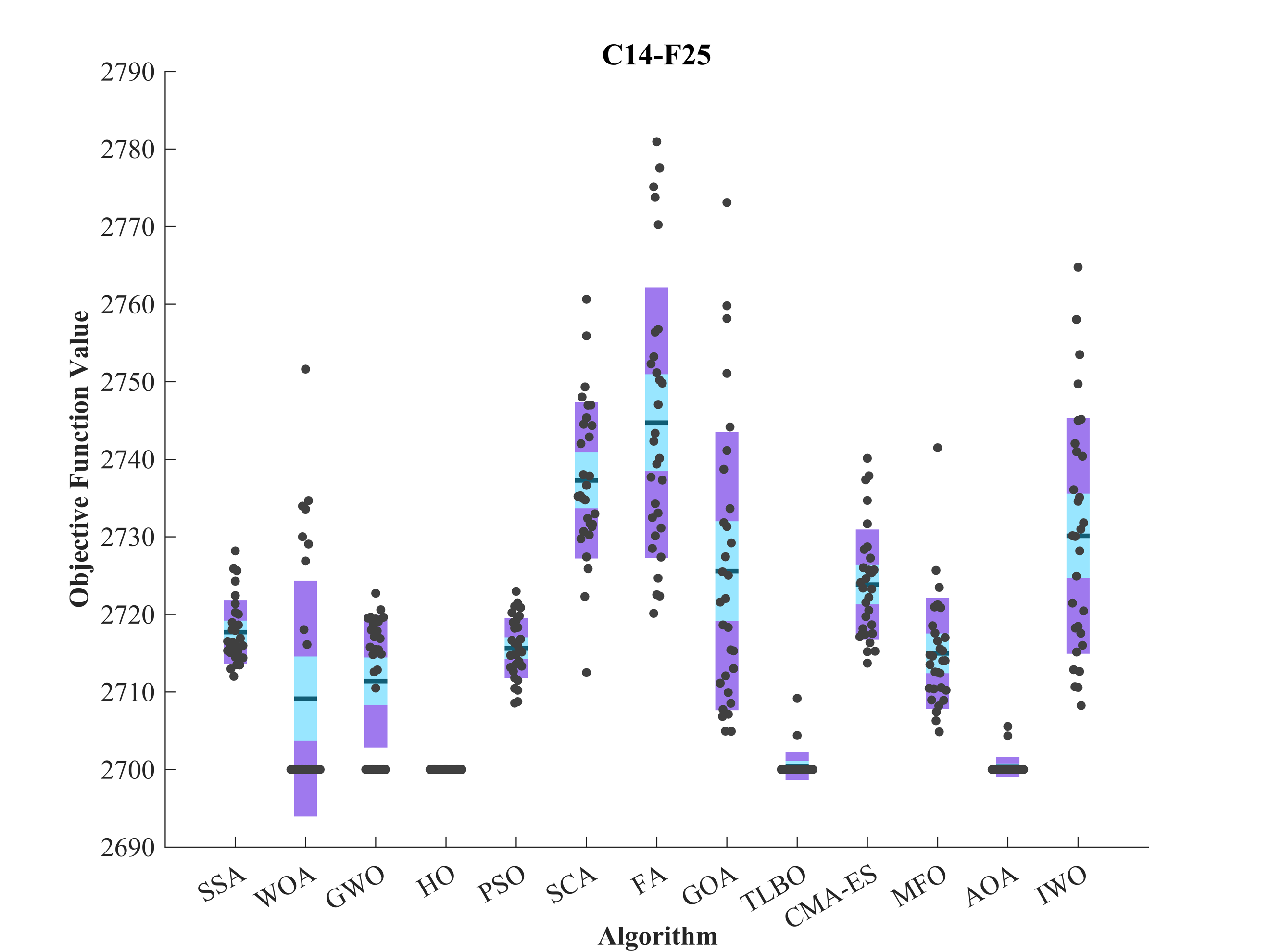 | 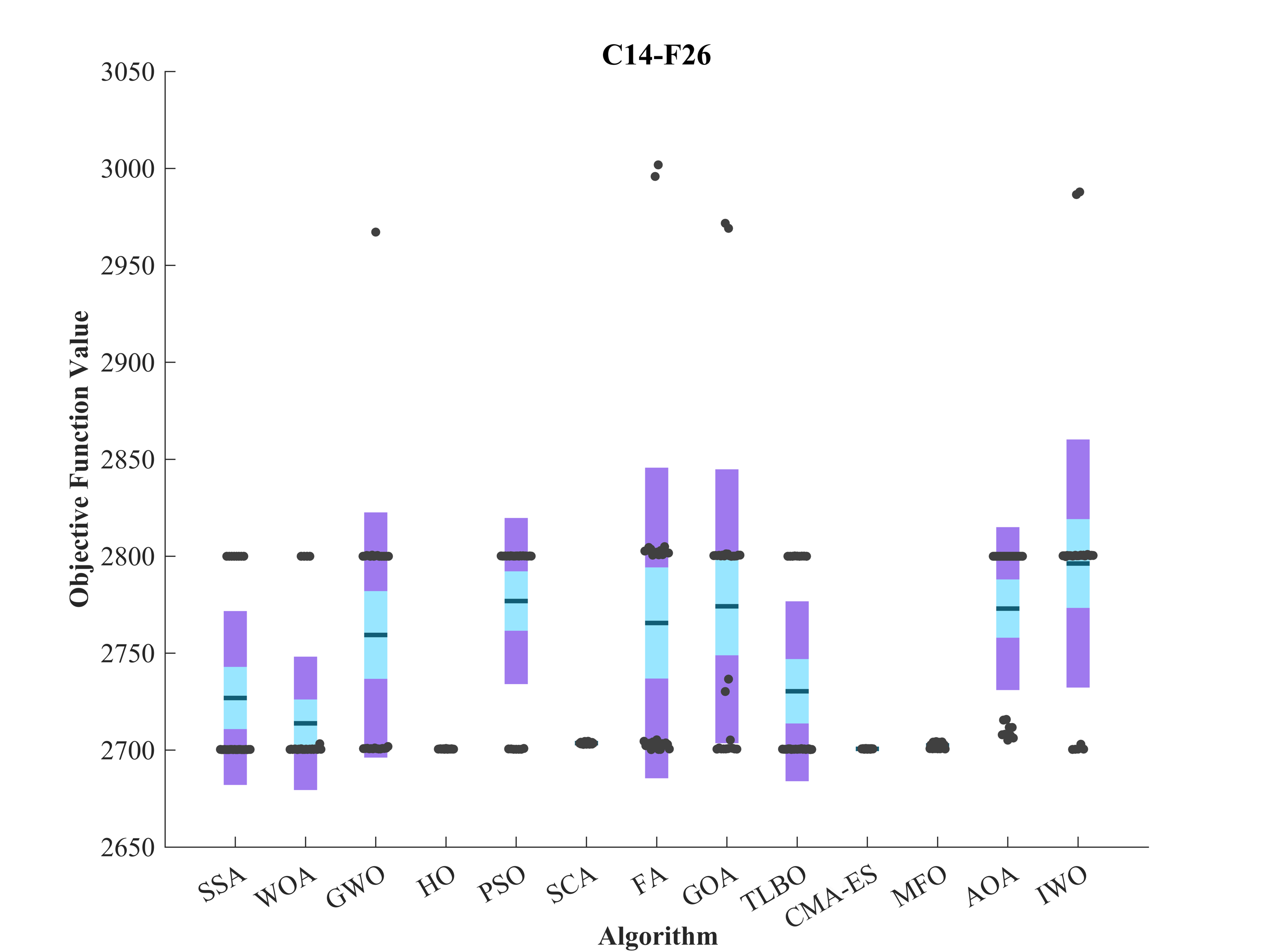 |
| --- | --- |
| 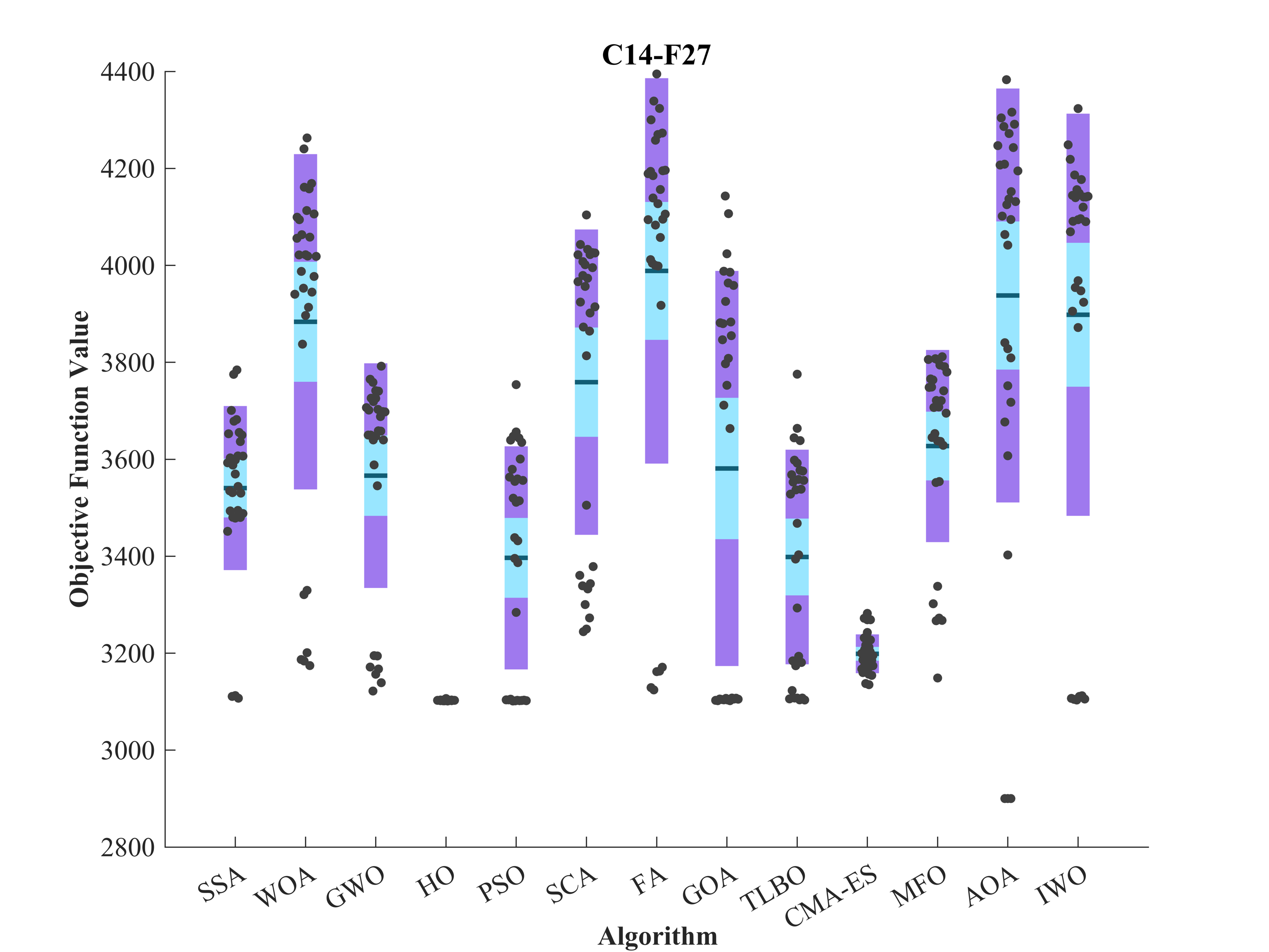 | 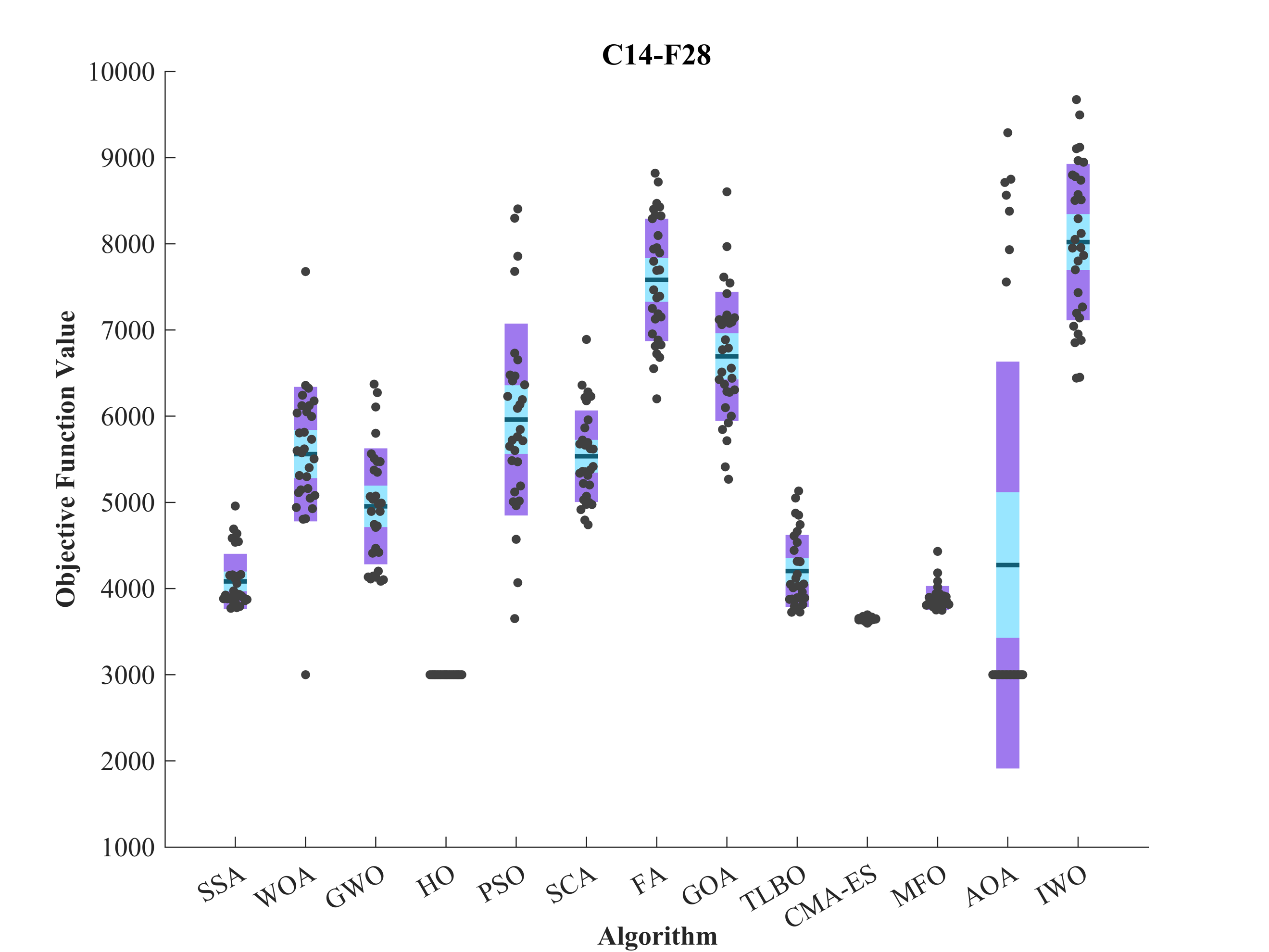 |
| 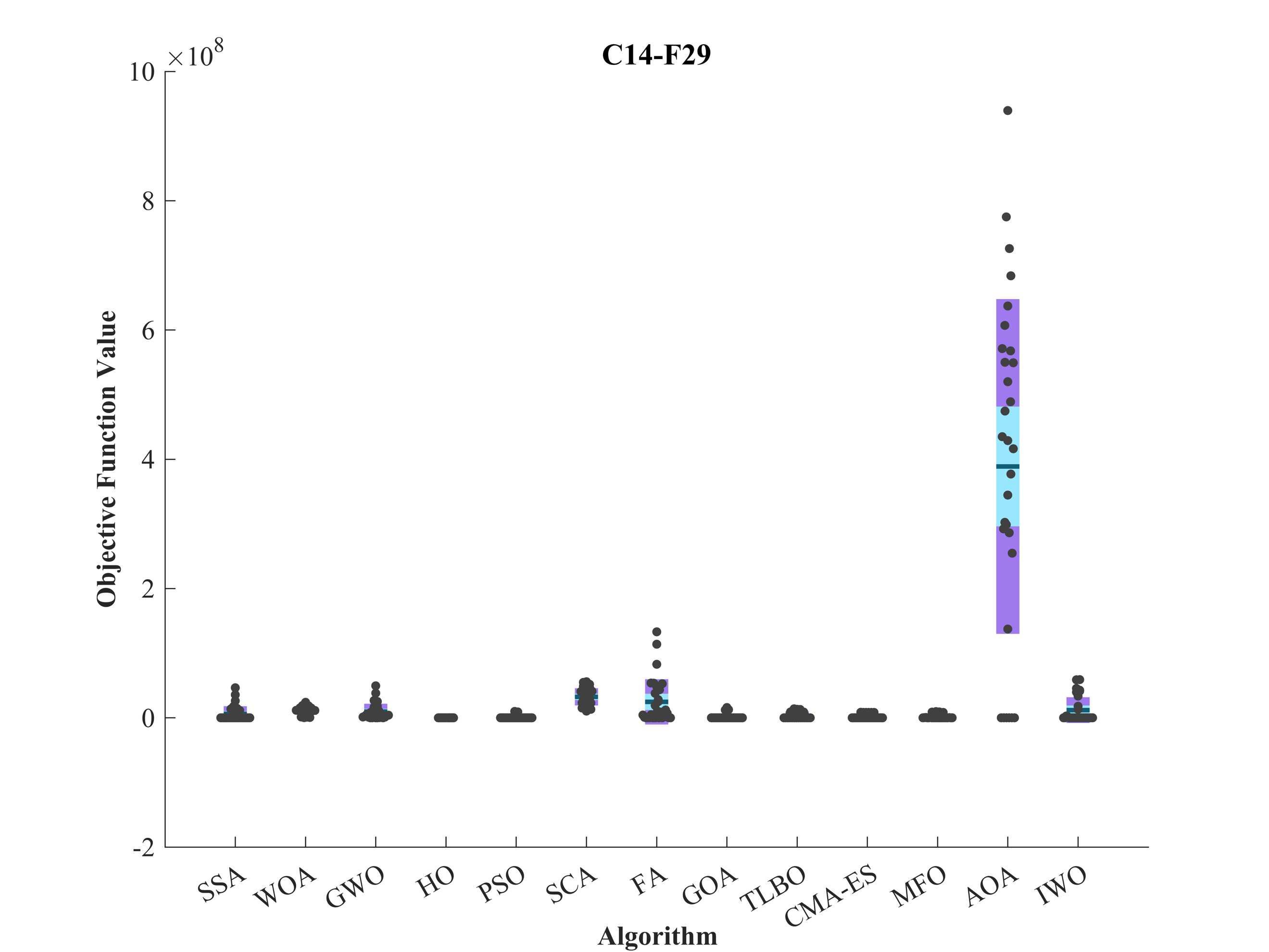 | 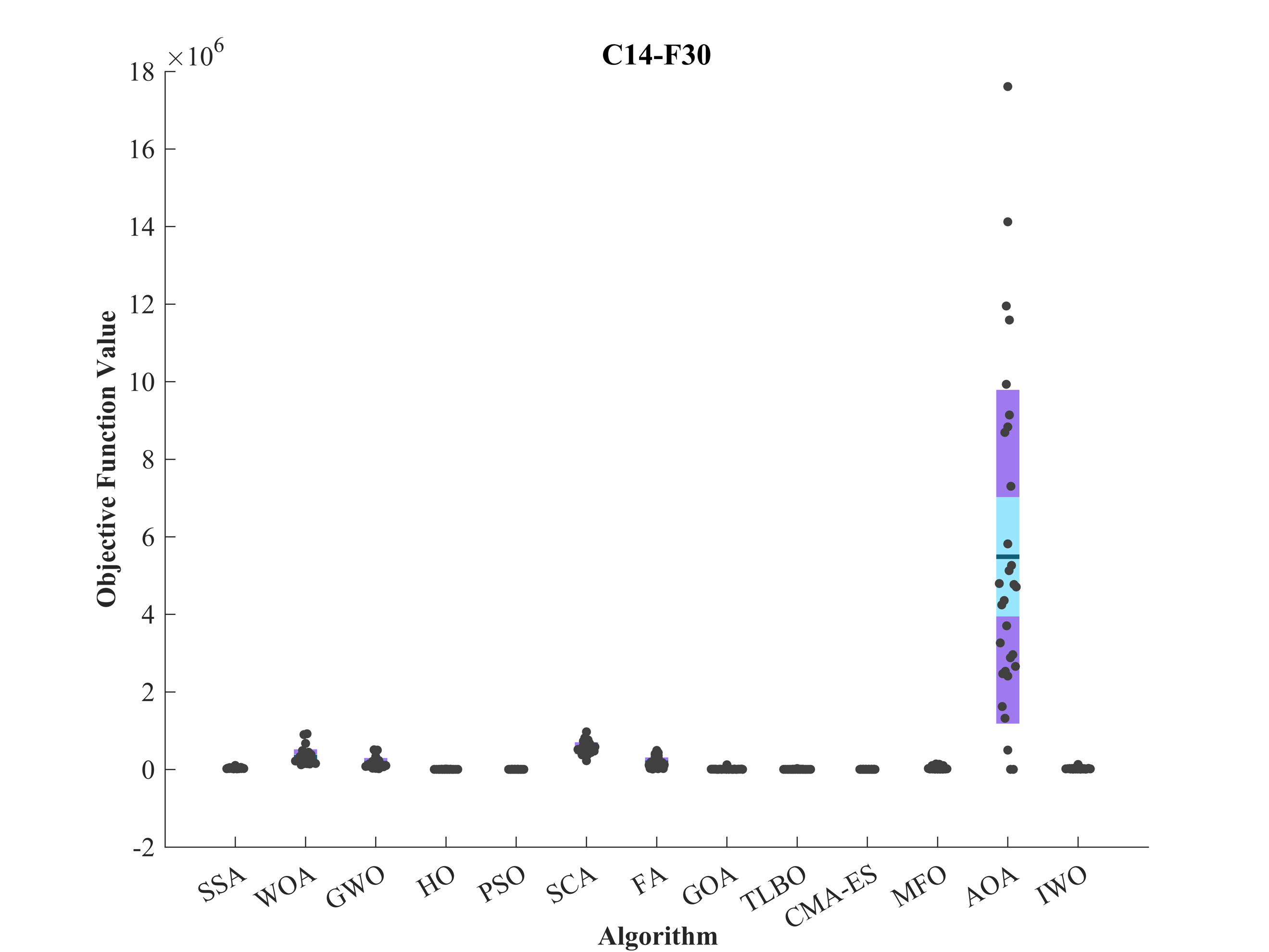 |

**Figure S4.** (continued)

| 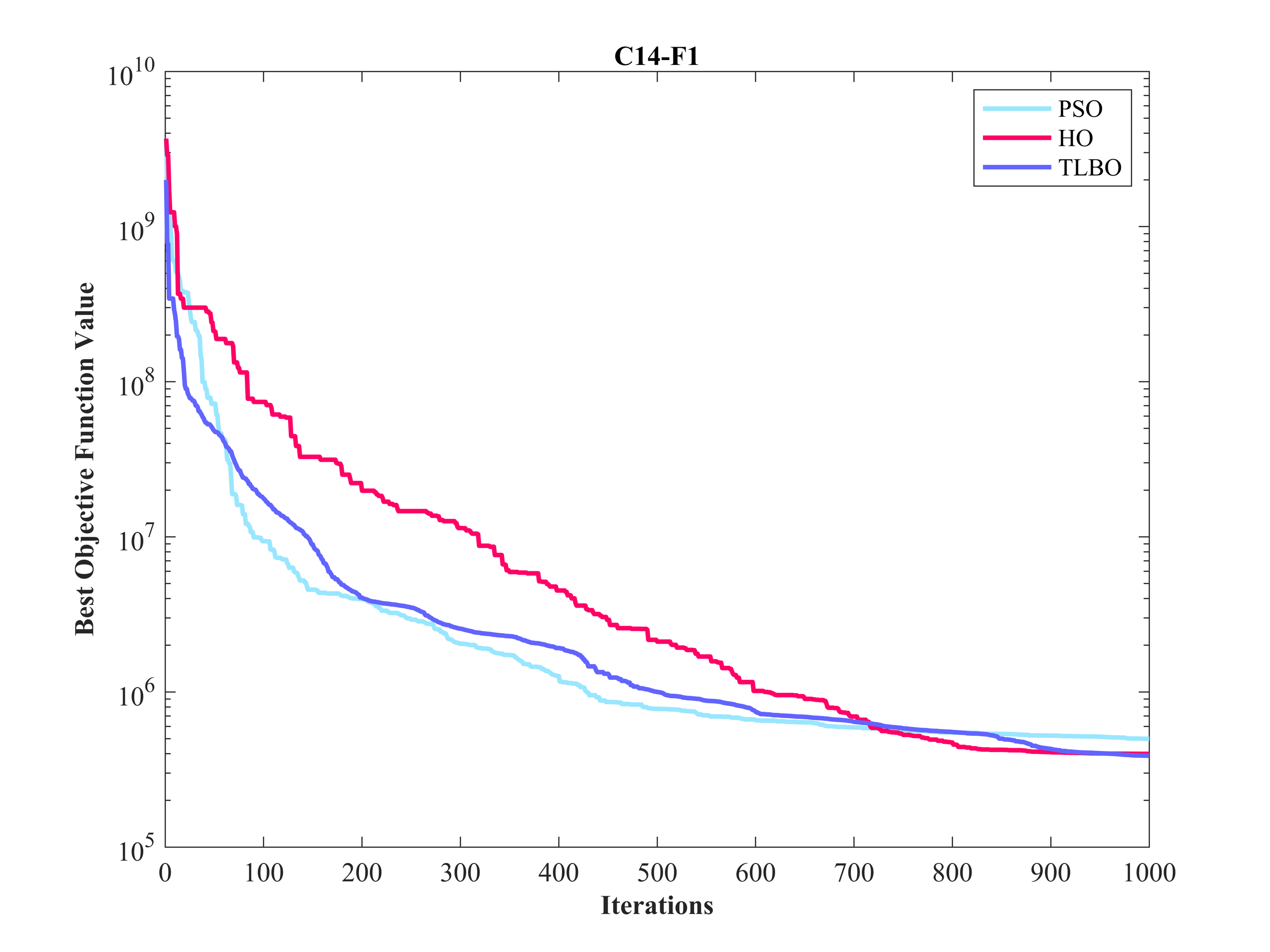 | 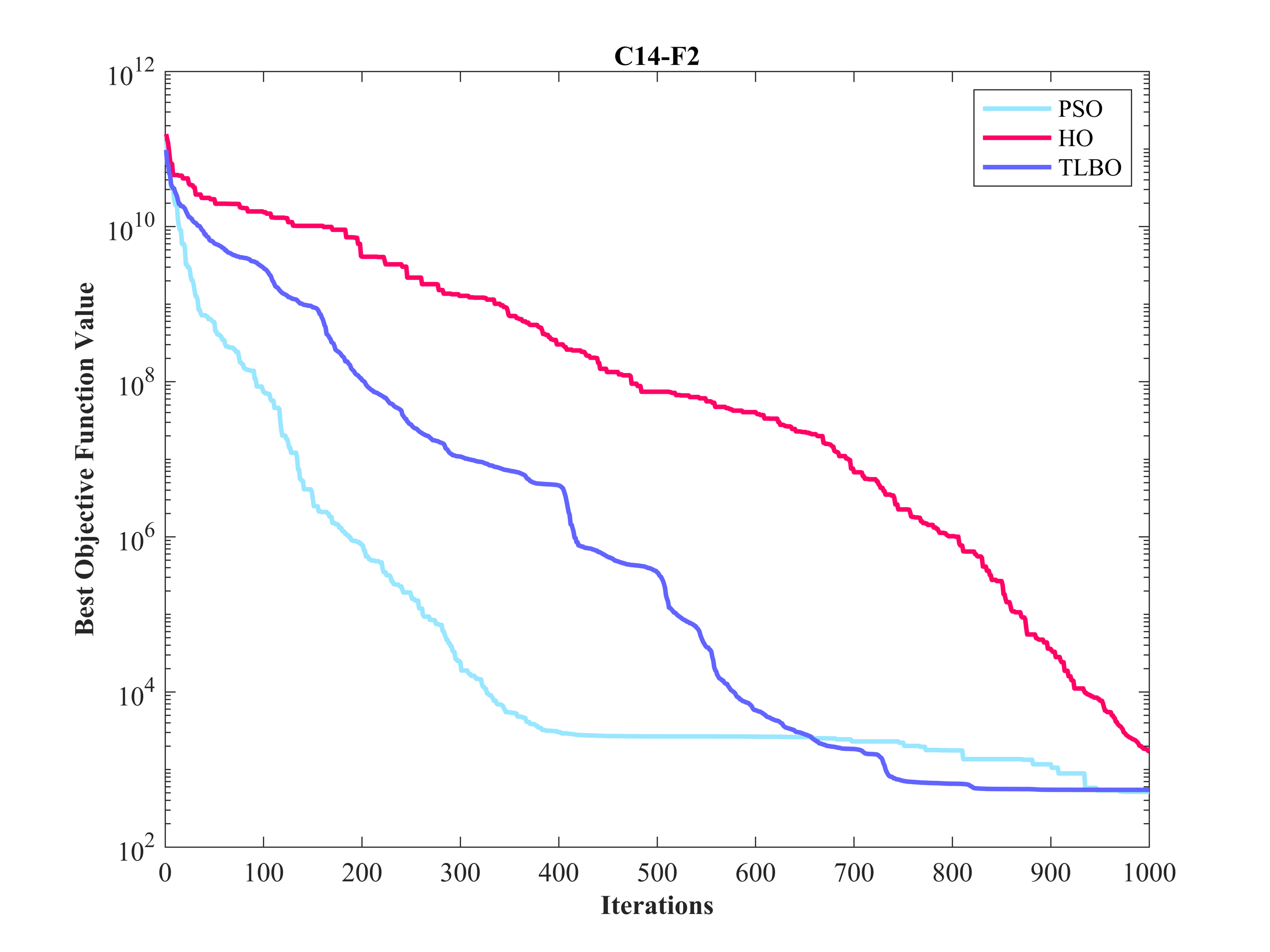 |
| --- | --- |
| 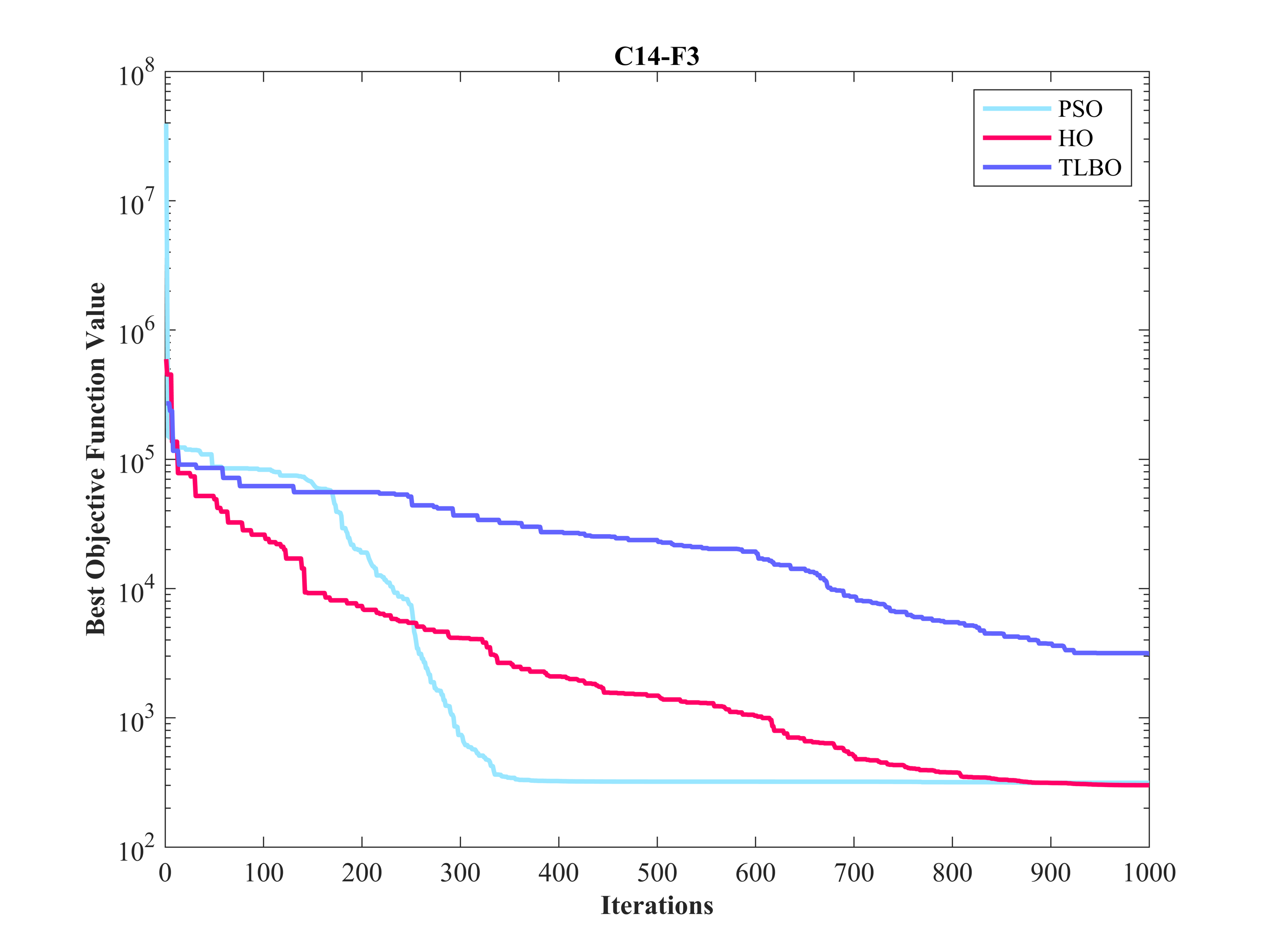 | 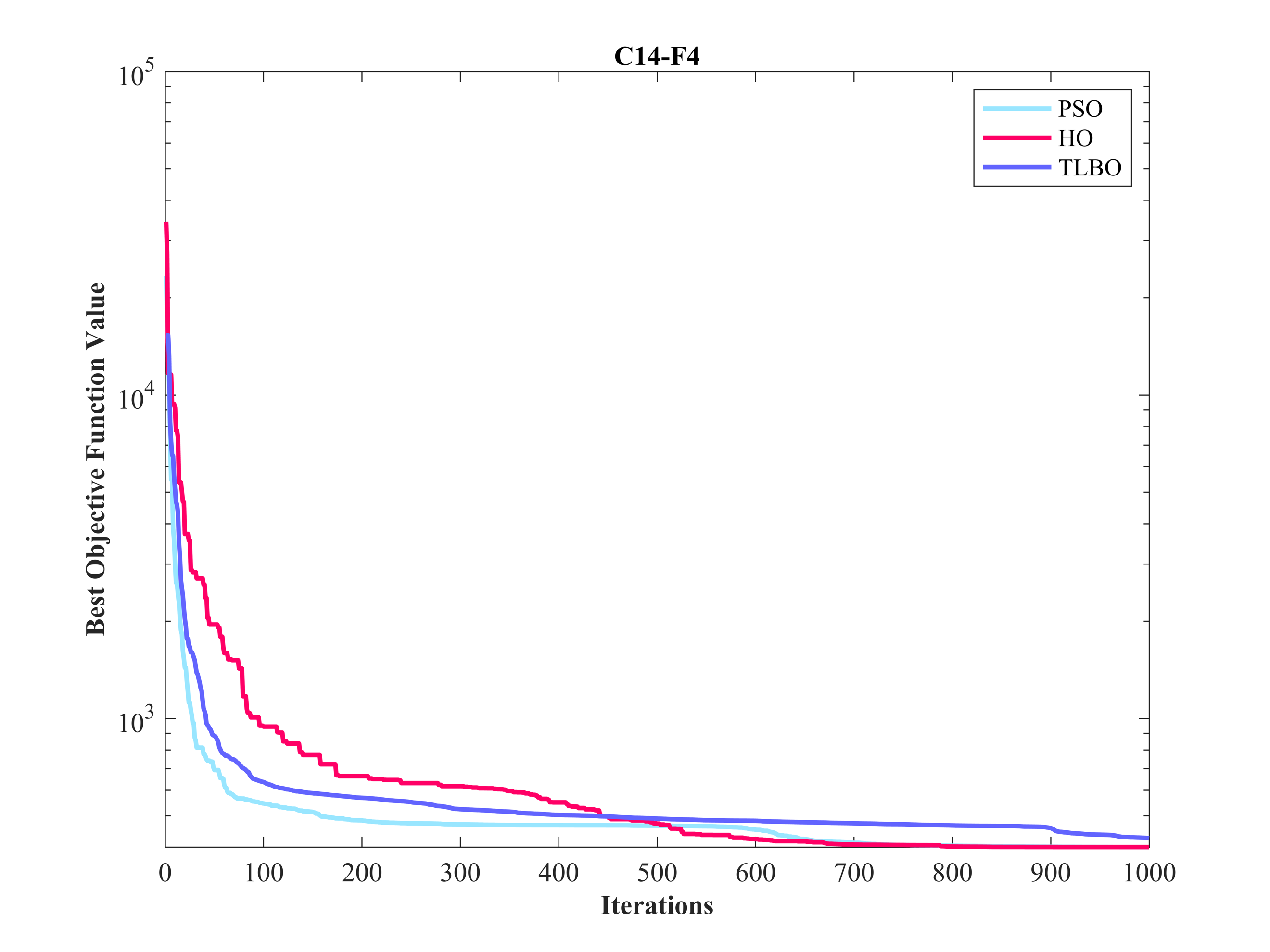 |
| 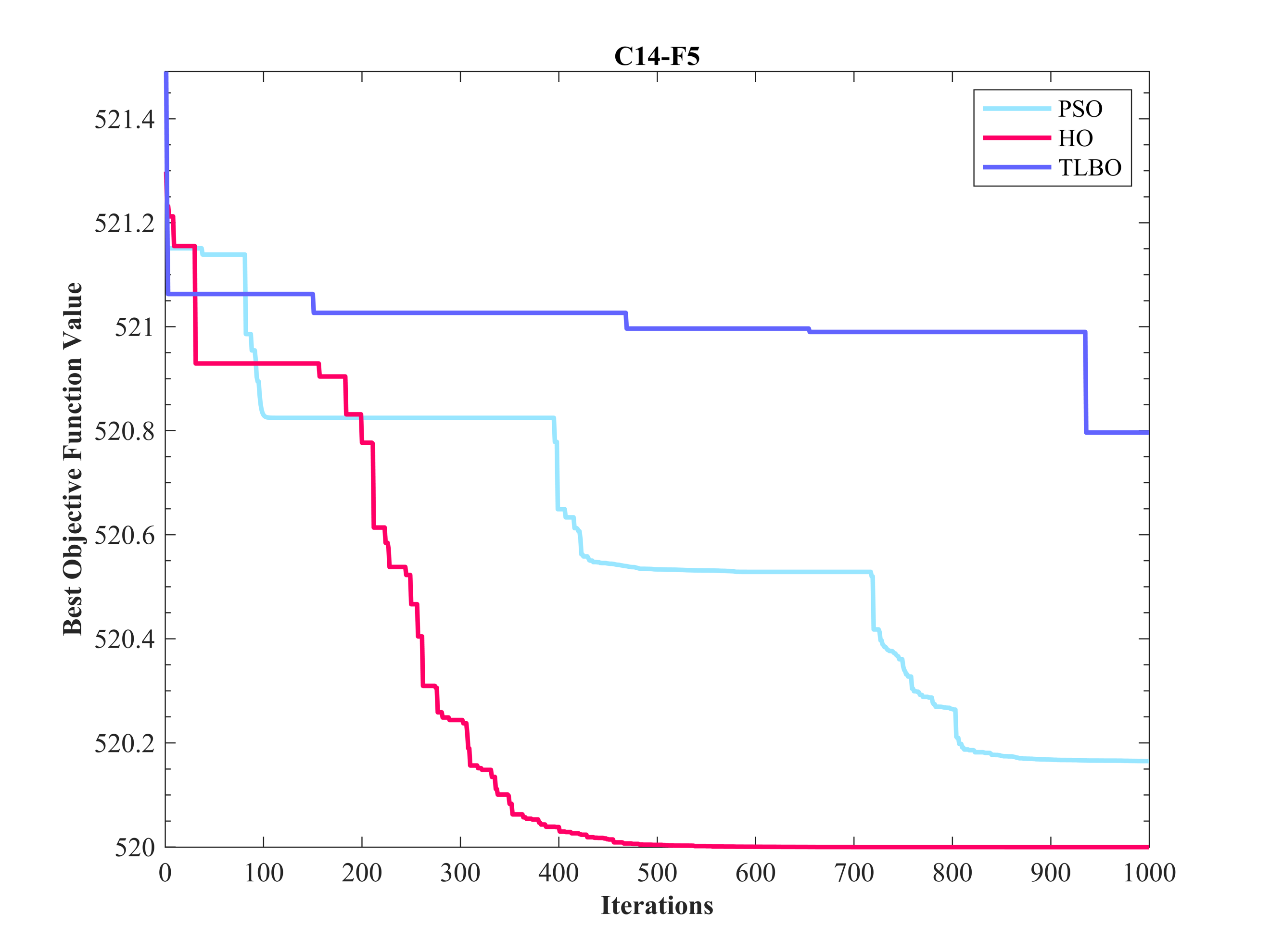 | 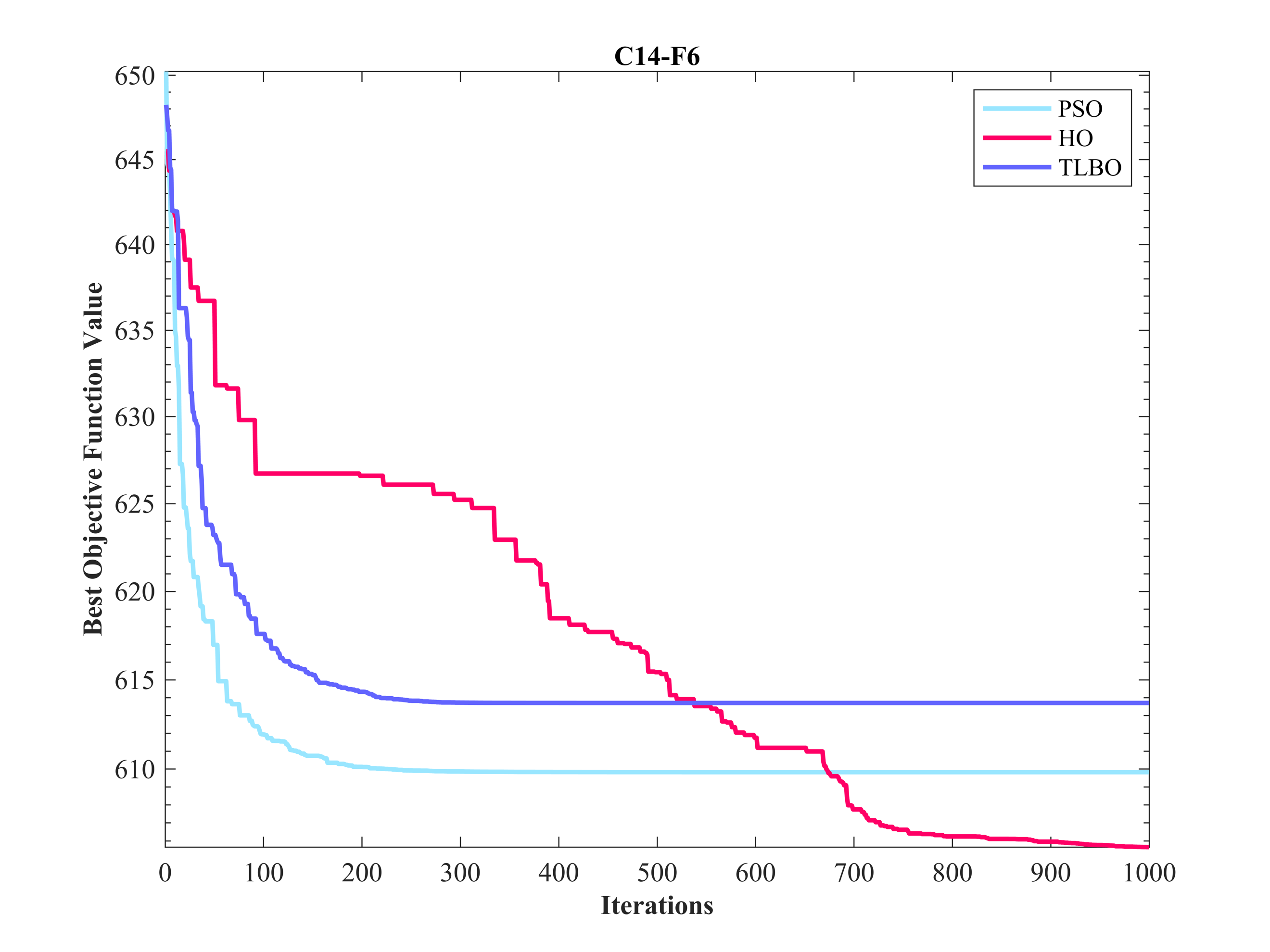 |
|  |  |

**Figure S5.** Convergence curves of the top three algorithms in each function in CEC 2014 (F1-F30) (D = 30).

|  |  |
| --- | --- |
|  |  |
|  |  |
|  |  |

**Figure S5.** (continued)

|  |  |
| --- | --- |
|  |  |
|  |  |
|  |  |

**Figure S5.** (continued)

|  |  |
| --- | --- |
|  |  |
|  |  |

**Figure S5.** (continued)

Evaluation outcomes for the objectives specified in the CEC 2014 (D = 50).

| **F** | **M** | **Optimization Algorithms** | | | | | | | | | | | | |
| --- | --- | --- | --- | --- | --- | --- | --- | --- | --- | --- | --- | --- | --- | --- |
|  |  | **HO** | **WOA** | **GWO** | **SSA** | **PSO** | **SCA** | **FA** | **GOA** | **TLBO** | **CMA-ES** | **MFO** | **AOA** | **IWO** |
| C14-F1 | Mean | 5.17E+06 | 2.38E+08 | 2.78E+08 | 4.28E+07 | 1.02E+07 | 1.19E+09 | 5.35E+08 | 2.18E+07 | 8.97E+06 | 7.17E+08 | 3.10E+08 | 3.48E+09 | 5.41E+07 |
|  | Best | 2.26E+06 | 8.84E+07 | 9.42E+07 | 2.23E+07 | 5.00E+06 | 8.29E+08 | 1.87E+08 | 2.32E+06 | 2.18E+06 | 4.10E+08 | 5.30E+07 | 1.44E+09 | 7.76E+06 |
|  | Worst | 1.13E+07 | 4.44E+08 | 5.49E+08 | 1.28E+08 | 1.97E+07 | 1.77E+09 | 1.10E+09 | 2.90E+08 | 2.25E+07 | 1.21E+09 | 9.00E+08 | 7.44E+09 | 1.57E+08 |
|  | Std. | 2.15E+06 | 8.31E+07 | 1.13E+08 | 2.08E+07 | 3.62E+06 | 2.42E+08 | 2.64E+08 | 5.20E+07 | 5.76E+06 | 2.04E+08 | 2.24E+08 | 1.55E+09 | 3.90E+07 |
|  | Median | 4.90E+06 | 2.37E+08 | 2.80E+08 | 3.81E+07 | 1.00E+07 | 1.14E+09 | 4.44E+08 | 8.29E+06 | 7.70E+06 | 7.08E+08 | 2.44E+08 | 2.86E+09 | 4.71E+07 |
|  | Rank | 1 | 7 | 8 | 5 | 3 | 12 | 10 | 4 | 2 | 11 | 9 | 13 | 6 |
| C14-F2 | Mean | 6.61E+06 | 9.25E+09 | 3.34E+10 | 7769.6 | 9162.1 | 7.38E+10 | 5.05E+10 | 1.50E+09 | 1.07E+08 | 5.15E+09 | 4.26E+10 | 1.57E+11 | 9.15E+08 |
|  | Best | 6.36E+05 | 4.99E+09 | 1.72E+10 | 203.16 | 284.64 | 5.33E+10 | 2.48E+10 | 216.35 | 37257 | 2.71E+09 | 1.67E+10 | 1.24E+11 | 986.94 |
|  | Worst | 3.35E+07 | 1.56E+10 | 5.25E+10 | 26932 | 27596 | 9.32E+10 | 7.29E+10 | 2.50E+10 | 1.64E+09 | 7.44E+09 | 8.42E+10 | 1.89E+11 | 3.54E+09 |
|  | Std. | 6.64E+06 | 2.63E+09 | 9.84E+09 | 9444.3 | 8388.9 | 7.88E+09 | 1.21E+10 | 4.93E+09 | 3.02E+08 | 1.18E+09 | 1.90E+10 | 1.73E+10 | 1.10E+09 |
|  | Median | 4.77E+06 | 8.97E+09 | 3.11E+10 | 3011.2 | 6506.9 | 7.32E+10 | 4.90E+10 | 2112.8 | 1.22E+07 | 5.04E+09 | 4.14E+10 | 1.63E+11 | 2.10E+08 |
|  | Rank | 3 | 8 | 9 | 1 | 2 | 12 | 11 | 6 | 4 | 7 | 10 | 13 | 5 |
| C14-F3 | Mean | 5829.9 | 1.46E+05 | 1.03E+05 | 1.27E+05 | 21987 | 1.34E+05 | 1.80E+05 | 46778 | 55158 | 2.28E+05 | 2.07E+05 | 1.42E+05 | 2.52E+05 |
|  | Best | 1791 | 1.21E+05 | 76765 | 89848 | 5292.8 | 94436 | 98983 | 18282 | 26863 | 1.78E+05 | 51045 | 1.26E+05 | 1.56E+05 |
|  | Worst | 10153 | 2.16E+05 | 1.30E+05 | 2.06E+05 | 43761 | 1.73E+05 | 2.94E+05 | 84415 | 87963 | 2.79E+05 | 3.75E+05 | 1.61E+05 | 3.52E+05 |
|  | Std. | 2262.3 | 23488 | 16751 | 25899 | 10891 | 18949 | 46817 | 19851 | 14912 | 25358 | 80900 | 9098.9 | 47441 |
|  | Median | 5449.4 | 1.40E+05 | 97111 | 1.18E+05 | 19651 | 1.33E+05 | 1.75E+05 | 37946 | 52810 | 2.27E+05 | 2.11E+05 | 1.41E+05 | 2.56E+05 |
|  | Rank | 1 | 9 | 5 | 6 | 2 | 7 | 10 | 3 | 4 | 12 | 11 | 8 | 13 |
| C14-F4 | Mean | 519.93 | 1782.9 | 4686.6 | 574.24 | 574.64 | 12677 | 7504 | 561.94 | 647.86 | 792.85 | 5060.3 | 41324 | 906.16 |
|  | Best | 468.59 | 929.93 | 2669.7 | 495.18 | 494.26 | 8481.7 | 4030.3 | 428.56 | 535.28 | 654.96 | 1355.2 | 22815 | 599.78 |
|  | Worst | 605.35 | 2740.5 | 8666.8 | 682.71 | 733 | 19735 | 12375 | 751.99 | 870.76 | 930.62 | 11432 | 58853 | 1419.3 |
|  | Std. | 34.445 | 436.5 | 1433.5 | 51.026 | 58.575 | 2613.4 | 2263.1 | 65.487 | 71.482 | 87.053 | 2556.2 | 9368.5 | 229.09 |
|  | Median | 503.23 | 1736.2 | 4522 | 569.91 | 562.6 | 12349 | 7297.3 | 551.81 | 637.48 | 775.55 | 4764.1 | 40045 | 814.42 |
|  | Rank | 1 | 8 | 9 | 3 | 4 | 12 | 11 | 2 | 5 | 6 | 10 | 13 | 7 |
| C14-F5 | Mean | 520 | 521.03 | 520.87 | 520.05 | 521 | 521.23 | 520.1 | 520.01 | 521.21 | 521.2 | 520.34 | 521.18 | 520 |
|  | Best | 520 | 520.85 | 520.57 | 520 | 520.05 | 521.17 | 520.01 | 520 | 521.16 | 521.12 | 520 | 521.12 | 520 |
|  | Worst | 520 | 521.23 | 521.08 | 520.25 | 521.27 | 521.28 | 520.19 | 520.09 | 521.27 | 521.27 | 520.72 | 521.27 | 520 |
|  | Std. | 0.00068024 | 0.095153 | 0.11494 | 0.080574 | 0.30494 | 0.034836 | 0.045359 | 0.02527 | 0.029157 | 0.037103 | 0.18564 | 0.037775 | 0.00031045 |
|  | Median | 520 | 521.04 | 520.9 | 520 | 521.13 | 521.24 | 520.1 | 520 | 521.22 | 521.21 | 520.38 | 521.18 | 520 |
|  | Rank | 1 | 9 | 7 | 4 | 8 | 13 | 5 | 3 | 12 | 11 | 6 | 10 | 2 |
| C14-F6 | Mean | 633.01 | 668.66 | 649.71 | 648.43 | 638.31 | 670.21 | 661.9 | 651.7 | 642.89 | 667.11 | 650.49 | 670.47 | 659.06 |
|  | Best | 625.51 | 660.94 | 642.01 | 635.93 | 630.53 | 664.64 | 652.26 | 639.83 | 635.79 | 654 | 640.49 | 666.02 | 650.89 |
|  | Worst | 641.25 | 678.11 | 658.13 | 657.79 | 653.17 | 676.11 | 669.47 | 659.74 | 649.41 | 675.84 | 663.1 | 676.43 | 666.04 |
|  | Std. | 4.2991 | 4.3185 | 3.5255 | 5.4288 | 5.1595 | 3.0665 | 3.8817 | 4.257 | 4.0463 | 4.856 | 4.2931 | 2.6087 | 4.313 |
|  | Median | 632.98 | 668.86 | 650.02 | 649.22 | 637.35 | 670.29 | 661.9 | 652.08 | 642.79 | 667.05 | 650.35 | 670.79 | 659.35 |
|  | Rank | 1 | 11 | 5 | 4 | 2 | 12 | 9 | 7 | 3 | 10 | 6 | 13 | 8 |

**Table S3.** Evaluation outcomes for the objectives specified in the CEC 2014 (D = 50).

| C14-F7 | Mean | 700.03 | 782.41 | 980.28 | 701.07 | 700.06 | 1384.9 | 1154.3 | 710.91 | 702.1 | 732.11 | 1142.3 | 2123.2 | 721.57 |
| --- | --- | --- | --- | --- | --- | --- | --- | --- | --- | --- | --- | --- | --- | --- |
|  | Best | 700 | 737.07 | 785.46 | 700.89 | 700 | 1251.8 | 851.61 | 700 | 700.46 | 723.8 | 730.56 | 1842 | 700.05 |
|  | Worst | 700.07 | 874.33 | 1151 | 701.2 | 700.56 | 1529.1 | 1361.9 | 790.81 | 714.6 | 745.06 | 1554.1 | 2435.5 | 810.01 |
|  | Std. | 0.017372 | 37.375 | 85.104 | 0.073119 | 0.11596 | 69.954 | 119.32 | 21.504 | 3.0198 | 5.9949 | 222.28 | 165.86 | 22.625 |
|  | Median | 700.03 | 769.12 | 979.69 | 701.08 | 700.02 | 1387.5 | 1147.6 | 700.02 | 701.16 | 730.57 | 1084.3 | 2120.2 | 714.23 |
|  | Rank | 1 | 8 | 9 | 3 | 2 | 12 | 11 | 5 | 4 | 7 | 10 | 13 | 6 |
| C14-F8 | Mean | 946.36 | 1233.8 | 1173.2 | 1113.4 | 1057.7 | 1376.9 | 1301.8 | 1249.6 | 995.84 | 1243.9 | 1123 | 1471.5 | 1378.5 |
|  | Best | 894.66 | 1124 | 1066.6 | 1017.7 | 977.1 | 1317.1 | 1147.2 | 1107.4 | 917.41 | 1205.9 | 964.01 | 1334.2 | 1244.7 |
|  | Worst | 1014 | 1369 | 1233.4 | 1228.8 | 1129.3 | 1437.7 | 1382 | 1347.1 | 1080.6 | 1282.2 | 1255.6 | 1530.6 | 1482.5 |
|  | Std. | 25.494 | 60.018 | 43.975 | 46.456 | 40.214 | 31.801 | 56.344 | 60.369 | 33.149 | 17.255 | 64.219 | 46.066 | 67.341 |
|  | Median | 946.21 | 1233.4 | 1187.9 | 1119.9 | 1057.2 | 1384.8 | 1300.9 | 1252.2 | 999.69 | 1242.8 | 1127.8 | 1480.1 | 1384.8 |
|  | Rank | 1 | 7 | 6 | 4 | 3 | 11 | 10 | 9 | 2 | 8 | 5 | 13 | 12 |
| C14-F9 | Mean | 1124.5 | 1471.8 | 1266.4 | 1242.7 | 1169.3 | 1502.5 | 1697.8 | 1594.9 | 1125.9 | 1408.2 | 1398.7 | 1596.4 | 1754.1 |
|  | Best | 1072.1 | 1345.1 | 1184.6 | 1119.9 | 1072.1 | 1404 | 1534 | 1362 | 1039.8 | 1362.2 | 1285.8 | 1486 | 1531.8 |
|  | Worst | 1181.4 | 1648.6 | 1369.4 | 1354.7 | 1316.9 | 1574.5 | 1848.3 | 1785.7 | 1264 | 1448.9 | 1546.9 | 1699.3 | 1986.5 |
|  | Std. | 29.983 | 69.896 | 45.108 | 62.057 | 47.732 | 35.123 | 99.931 | 107.82 | 43.98 | 21.518 | 65.927 | 49.743 | 105.75 |
|  | Median | 1127.7 | 1467.3 | 1259 | 1237.8 | 1161.7 | 1503.9 | 1692.1 | 1595.1 | 1114.9 | 1410.4 | 1389.7 | 1601.4 | 1748.7 |
|  | Rank | 1 | 8 | 5 | 4 | 3 | 9 | 12 | 10 | 2 | 7 | 6 | 11 | 13 |
| C14-F10 | Mean | 4021.3 | 10717 | 9068.1 | 8222.2 | 6186.1 | 14261 | 8760.3 | 6848.3 | 6273.2 | 13583 | 7656.3 | 13879 | 8728.9 |
|  | Best | 2196.9 | 8390.8 | 7158 | 5961.3 | 4804 | 13139 | 6908.9 | 5116 | 4374 | 12646 | 4552.1 | 12730 | 6684.6 |
|  | Worst | 5227.2 | 13485 | 11164 | 11032 | 7376.3 | 15366 | 10606 | 7974.2 | 8801.3 | 14495 | 9693.2 | 15086 | 10181 |
|  | Std. | 726.14 | 1267.7 | 981.34 | 1171.7 | 753.44 | 543.42 | 1041.4 | 708.32 | 1215.1 | 509.21 | 1426.3 | 606.71 | 838.39 |
|  | Median | 4095.9 | 10844 | 9088.9 | 8105.6 | 6162.4 | 14411 | 8927.8 | 6908.8 | 6118.6 | 13644 | 7718 | 13838 | 8856.4 |
|  | Rank | 1 | 10 | 9 | 6 | 2 | 13 | 8 | 4 | 3 | 11 | 5 | 12 | 7 |
| C14-F11 | Mean | 6883.7 | 12917 | 9473.9 | 7950.3 | 7552 | 15330 | 8228.3 | 8015 | 14428 | 15036 | 8685.7 | 14331 | 8477.6 |
|  | Best | 5320.3 | 10043 | 7127.4 | 5594 | 5411.9 | 14542 | 6013.2 | 6384.7 | 12590 | 13877 | 6297.9 | 13428 | 6333.2 |
|  | Worst | 7909.9 | 15584 | 12383 | 9417.8 | 9564.6 | 16105 | 11190 | 9471.7 | 15732 | 15754 | 10899 | 15179 | 10109 |
|  | Std. | 636.11 | 1166.8 | 1020.8 | 947.08 | 953.82 | 381.33 | 1067.6 | 830.86 | 679 | 395.59 | 1071.9 | 531.7 | 985.57 |
|  | Median | 6741 | 12939 | 9519.7 | 7970.8 | 7472.6 | 15358 | 8019.7 | 7961.9 | 14535 | 14991 | 8491.5 | 14312 | 8620.7 |
|  | Rank | 1 | 9 | 8 | 3 | 2 | 13 | 5 | 4 | 11 | 12 | 7 | 10 | 6 |
| C14-F12 | Mean | 1200.1 | 1203.2 | 1201.3 | 1201.1 | 1200.7 | 1204.2 | 1200.1 | 1200.2 | 1203.9 | 1204 | 1200.6 | 1203.1 | 1200.2 |
|  | Best | 1200 | 1201.6 | 1200.7 | 1200.5 | 1200.4 | 1203.4 | 1200 | 1200.1 | 1203 | 1203.1 | 1200.2 | 1202.2 | 1200.1 |
|  | Worst | 1200.1 | 1204.5 | 1202.8 | 1202.7 | 1202.3 | 1204.8 | 1200.3 | 1200.6 | 1204.6 | 1204.4 | 1201.7 | 1204.3 | 1200.5 |
|  | Std. | 0.021824 | 0.78599 | 0.54224 | 0.49764 | 0.3617 | 0.34135 | 0.062568 | 0.11939 | 0.31985 | 0.36529 | 0.32768 | 0.58445 | 0.082416 |
|  | Median | 1200 | 1203.3 | 1201.3 | 1201.1 | 1200.6 | 1204.2 | 1200.1 | 1200.2 | 1203.9 | 1204.1 | 1200.5 | 1202.9 | 1200.2 |
|  | Rank | 1 | 10 | 8 | 7 | 6 | 13 | 2 | 4 | 11 | 12 | 5 | 9 | 3 |
| C14-F13 | Mean | 1300.6 | 1300.6 | 1303.7 | 1300.5 | 1300.6 | 1305.6 | 1304.7 | 1300.7 | 1300.7 | 1301.1 | 1303.9 | 1308.2 | 1300.5 |
|  | Best | 1300.4 | 1300.4 | 1300.9 | 1300.3 | 1300.3 | 1304.8 | 1303.3 | 1300.5 | 1300.5 | 1300.9 | 1300.7 | 1307.1 | 1300.4 |
|  | Worst | 1300.8 | 1302.2 | 1304.7 | 1300.8 | 1300.8 | 1306.5 | 1305.9 | 1301.1 | 1301 | 1301.3 | 1306.3 | 1309.3 | 1300.7 |
|  | Std. | 0.10559 | 0.31188 | 0.69422 | 0.10227 | 0.105 | 0.36194 | 0.55546 | 0.13772 | 0.13009 | 0.11939 | 1.3013 | 0.64022 | 0.081491 |
|  | Median | 1300.6 | 1300.6 | 1303.8 | 1300.5 | 1300.6 | 1305.6 | 1304.7 | 1300.7 | 1300.7 | 1301.1 | 1304.2 | 1308.2 | 1300.5 |
|  | Rank | 4 | 5 | 9 | 2 | 3 | 12 | 11 | 7 | 6 | 8 | 10 | 13 | 1 |

**Table S3.** (continued)

| C14-F14 | Mean | 1400.4 | 1413 | 1486 | 1400.5 | 1400.4 | 1577 | 1520.4 | 1400.5 | 1400.4 | 1407.5 | 1524.9 | 1751.6 | 1401.2 |
| --- | --- | --- | --- | --- | --- | --- | --- | --- | --- | --- | --- | --- | --- | --- |
|  | Best | 1400.2 | 1400.3 | 1444.8 | 1400.3 | 1400.2 | 1541 | 1451.8 | 1400.2 | 1400.2 | 1401.4 | 1401 | 1659.2 | 1400.2 |
|  | Worst | 1401.1 | 1434.3 | 1546.6 | 1400.9 | 1401.1 | 1630.6 | 1607.7 | 1401.3 | 1401 | 1416.2 | 1641.7 | 1834.8 | 1427 |
|  | Std. | 0.18121 | 8.2185 | 23.945 | 0.20804 | 0.22194 | 20.092 | 32.558 | 0.31308 | 0.16096 | 4.2152 | 54.989 | 42.799 | 4.8659 |
|  | Median | 1400.3 | 1412.1 | 1489.4 | 1400.4 | 1400.3 | 1574 | 1525.2 | 1400.4 | 1400.3 | 1407.7 | 1529.2 | 1745.9 | 1400.3 |
|  | Rank | 1 | 8 | 9 | 4 | 3 | 12 | 10 | 5 | 2 | 7 | 11 | 13 | 6 |
| C14-F15 | Mean | 1525.5 | 19800 | 65381 | 1546.4 | 1540.7 | 4.43E+05 | 1.44E+05 | 1926 | 1897.8 | 7887 | 9.24E+05 | 3.45E+06 | 1707.8 |
|  | Best | 1513.8 | 5351.5 | 7527.9 | 1518.7 | 1526.2 | 1.53E+05 | 10357 | 1522.1 | 1590.9 | 2977.5 | 5583.3 | 1.14E+06 | 1551.6 |
|  | Worst | 1545.3 | 56007 | 3.10E+05 | 1600.5 | 1591.1 | 1.16E+06 | 4.49E+05 | 7595.8 | 3883.8 | 19072 | 4.68E+06 | 1.07E+07 | 2398.3 |
|  | Std. | 9.0063 | 13667 | 54048 | 16.493 | 14.36 | 2.23E+05 | 1.22E+05 | 1249.3 | 489.59 | 4513 | 1.19E+06 | 2.22E+06 | 190.57 |
|  | Median | 1523.7 | 16825 | 55728 | 1543.4 | 1536.6 | 4.28E+05 | 1.13E+05 | 1555.5 | 1703.3 | 6884.6 | 3.00E+05 | 2.76E+06 | 1639.2 |
|  | Rank | 1 | 8 | 9 | 3 | 2 | 11 | 10 | 6 | 5 | 7 | 12 | 13 | 4 |
| C14-F16 | Mean | 1620.6 | 1622.6 | 1621.8 | 1621.8 | 1621.6 | 1623 | 1622.5 | 1623.1 | 1622.1 | 1623.1 | 1622.4 | 1622.5 | 1623.1 |
|  | Best | 1619.3 | 1620.9 | 1620.5 | 1620.2 | 1620.4 | 1622.4 | 1620.6 | 1621.3 | 1621.2 | 1622.4 | 1621.3 | 1622 | 1622 |
|  | Worst | 1621.9 | 1623.4 | 1622.5 | 1623.4 | 1622.5 | 1623.4 | 1623.6 | 1624 | 1622.8 | 1623.3 | 1623.3 | 1622.9 | 1623.8 |
|  | Std. | 0.66243 | 0.65939 | 0.57284 | 0.68711 | 0.52358 | 0.2339 | 0.58282 | 0.56523 | 0.36805 | 0.20875 | 0.53318 | 0.28569 | 0.41388 |
|  | Median | 1620.7 | 1622.8 | 1621.9 | 1621.9 | 1621.7 | 1623 | 1622.7 | 1623.2 | 1622.1 | 1623.1 | 1622.4 | 1622.5 | 1623.2 |
|  | Rank | 1 | 9 | 3 | 4 | 2 | 10 | 8 | 13 | 5 | 11 | 6 | 7 | 12 |
| C14-F17 | Mean | 80275 | 1.44E+08 | 2.69E+07 | 3.45E+06 | 1.08E+06 | 9.46E+07 | 1.28E+07 | 1.36E+06 | 7.87E+05 | 4.69E+07 | 1.93E+07 | 3.69E+08 | 9.80E+05 |
|  | Best | 11581 | 5.08E+07 | 1.17E+06 | 1.04E+06 | 1.99E+05 | 2.56E+07 | 1.05E+06 | 1.75E+05 | 87451 | 1.57E+07 | 1.52E+06 | 1.30E+08 | 2.49E+05 |
|  | Worst | 3.00E+05 | 2.82E+08 | 1.04E+08 | 1.11E+07 | 3.17E+06 | 2.37E+08 | 4.86E+07 | 5.84E+06 | 4.91E+06 | 1.29E+08 | 8.71E+07 | 8.91E+08 | 2.86E+06 |
|  | Std. | 69582 | 5.94E+07 | 2.99E+07 | 2.29E+06 | 6.88E+05 | 4.46E+07 | 1.06E+07 | 1.31E+06 | 8.59E+05 | 2.42E+07 | 1.99E+07 | 1.83E+08 | 6.41E+05 |
|  | Median | 54089 | 1.41E+08 | 1.70E+07 | 2.72E+06 | 9.42E+05 | 8.01E+07 | 1.17E+07 | 9.82E+05 | 6.47E+05 | 4.90E+07 | 1.41E+07 | 3.36E+08 | 8.06E+05 |
|  | Rank | 1 | 12 | 9 | 6 | 4 | 11 | 7 | 5 | 2 | 10 | 8 | 13 | 3 |
| C14-F18 | Mean | 2868.3 | 4.83E+07 | 5.63E+08 | 4380.4 | 3502.7 | 2.50E+09 | 5.21E+07 | 4602.4 | 3829.2 | 4088.3 | 5.31E+08 | 1.62E+10 | 4114.8 |
|  | Best | 2229.8 | 9.45E+05 | 1.51E+05 | 2428.2 | 2010.1 | 1.43E+09 | 2937.8 | 2331.1 | 2049.7 | 2039.1 | 2896.8 | 7.01E+09 | 2403.5 |
|  | Worst | 8604.9 | 4.34E+08 | 2.02E+09 | 9057.9 | 7971.6 | 4.88E+09 | 7.88E+08 | 6148.5 | 8569.3 | 8624.5 | 3.01E+09 | 2.62E+10 | 6630.4 |
|  | Std. | 1181.3 | 9.66E+07 | 4.85E+08 | 2108.8 | 1562.3 | 7.69E+08 | 1.52E+08 | 1577.2 | 1546 | 1883.4 | 7.92E+08 | 5.12E+09 | 1533.7 |
|  | Median | 2483.3 | 5.97E+06 | 4.75E+08 | 3426.4 | 2815.2 | 2.32E+09 | 1.27E+06 | 5748.2 | 3601.4 | 3547.8 | 1.83E+08 | 1.56E+10 | 3653.8 |
|  | Rank | 1 | 8 | 11 | 6 | 2 | 12 | 9 | 7 | 3 | 4 | 10 | 13 | 5 |
| C14-F19 | Mean | 1945.3 | 2161.5 | 2091.2 | 1953.7 | 1945.7 | 2315.4 | 2182.2 | 1960.9 | 1963.9 | 1950.3 | 2154.3 | 3721.3 | 1969.5 |
|  | Best | 1915.5 | 2015.3 | 1983.9 | 1926.1 | 1916.5 | 2175 | 2040 | 1919.5 | 1921.8 | 1921.4 | 1940.8 | 2706.2 | 1927.8 |
|  | Worst | 1983.1 | 2333.6 | 2298.9 | 2001.6 | 1986.1 | 2526.7 | 2349.8 | 2114 | 1998.1 | 1980.5 | 2662.4 | 6126.4 | 2013.1 |
|  | Std. | 25.118 | 78.238 | 74.791 | 23.781 | 24.332 | 76.533 | 89.122 | 42.197 | 26.709 | 12.854 | 200.25 | 782.4 | 26.749 |
|  | Median | 1943 | 2155.9 | 2070 | 1949 | 1944.8 | 2303.6 | 2187.1 | 1949.3 | 1971.2 | 1948.9 | 2086.4 | 3742.1 | 1969.9 |
|  | Rank | 1 | 10 | 8 | 4 | 2 | 12 | 11 | 5 | 6 | 3 | 9 | 13 | 7 |
| C14-F20 | Mean | 2641 | 6.77E+05 | 44436 | 71106 | 22730 | 94469 | 71835 | 49348 | 15898 | 1.14E+05 | 1.76E+05 | 1.21E+05 | 95923 |
|  | Best | 2202.5 | 54776 | 21814 | 19458 | 8128.4 | 47401 | 35279 | 15211 | 6249.4 | 58206 | 30367 | 60018 | 41431 |
|  | Worst | 3713.5 | 2.42E+06 | 77257 | 1.65E+05 | 47330 | 2.07E+05 | 1.47E+05 | 93723 | 23834 | 1.79E+05 | 1.04E+06 | 2.29E+05 | 1.74E+05 |
|  | Std. | 344.72 | 6.64E+05 | 12766 | 35678 | 9371.6 | 37988 | 29064 | 20189 | 5318.1 | 29970 | 2.12E+05 | 43773 | 34447 |
|  | Median | 2545.3 | 3.89E+05 | 42816 | 62398 | 21706 | 82974 | 65796 | 48111 | 15713 | 1.16E+05 | 1.07E+05 | 1.24E+05 | 90454 |
|  | Rank | 1 | 13 | 4 | 6 | 3 | 8 | 7 | 5 | 2 | 10 | 12 | 11 | 9 |

**Table S3.** (continued)

| C14-F21 | Mean | 8915.6 | 2.16E+07 | 7.91E+06 | 2.63E+06 | 5.86E+05 | 2.65E+07 | 3.90E+06 | 1.02E+06 | 6.39E+05 | 2.33E+07 | 7.92E+06 | 6.50E+07 | 7.51E+05 |
| --- | --- | --- | --- | --- | --- | --- | --- | --- | --- | --- | --- | --- | --- | --- |
|  | Best | 4658.4 | 3.44E+06 | 1.13E+06 | 5.28E+05 | 94253 | 8.59E+06 | 2.25E+05 | 1.34E+05 | 94676 | 5.77E+06 | 5.41E+05 | 1.99E+07 | 1.34E+05 |
|  | Worst | 33439 | 5.43E+07 | 2.16E+07 | 5.81E+06 | 1.49E+06 | 6.86E+07 | 1.96E+07 | 7.89E+06 | 1.51E+06 | 6.00E+07 | 2.20E+07 | 1.82E+08 | 2.48E+06 |
|  | Std. | 5185.6 | 1.20E+07 | 5.42E+06 | 1.44E+06 | 2.89E+05 | 1.21E+07 | 4.83E+06 | 1.89E+06 | 3.65E+05 | 1.43E+07 | 6.78E+06 | 4.29E+07 | 5.23E+05 |
|  | Median | 7884.8 | 2.05E+07 | 7.38E+06 | 2.60E+06 | 5.32E+05 | 2.55E+07 | 1.73E+06 | 3.55E+05 | 6.08E+05 | 1.87E+07 | 4.49E+06 | 5.70E+07 | 7.19E+05 |
|  | Rank | 1 | 10 | 8 | 6 | 2 | 12 | 7 | 5 | 3 | 11 | 9 | 13 | 4 |
| C14-F22 | Mean | 2970.2 | 4656.9 | 3666.4 | 3499.8 | 3510 | 5023.4 | 4210.2 | 4089.9 | 3261.5 | 3921.3 | 3931 | 1.52E+05 | 4129.4 |
|  | Best | 2600 | 3385 | 2935.7 | 2816.4 | 2818.1 | 4594.9 | 3372.3 | 3210.5 | 2760.9 | 3589.1 | 3347.4 | 7536.8 | 3560.8 |
|  | Worst | 3265.5 | 6075.7 | 5200.1 | 4172.8 | 4072.7 | 5488.7 | 5295.6 | 5008.5 | 3940.5 | 4264 | 4598.6 | 1.53E+06 | 4987.6 |
|  | Std. | 188.87 | 632.04 | 452.25 | 381.08 | 313.5 | 245.64 | 505.26 | 424.5 | 279.1 | 145 | 322.99 | 2.83E+05 | 358.02 |
|  | Median | 2986.6 | 4577.1 | 3639 | 3550.9 | 3548.8 | 5080.6 | 4162.2 | 4094.3 | 3249.7 | 3939.3 | 3954.3 | 64233 | 4093.6 |
|  | Rank | 1 | 11 | 5 | 3 | 4 | 12 | 10 | 8 | 2 | 6 | 7 | 13 | 9 |
| C14-F23 | Mean | 2500 | 2688 | 2876 | 2688.8 | 2644 | 3199.8 | 2955 | 2648.8 | 2644.9 | 2647.1 | 2840.5 | 2500 | 2704.9 |
|  | Best | 2500 | 2500 | 2710.8 | 2663.1 | 2644 | 3058.8 | 2813.5 | 2644 | 2644 | 2645.2 | 2672.9 | 2500 | 2668.2 |
|  | Worst | 2500 | 2947 | 3082 | 2719.1 | 2644 | 3427.8 | 3122.3 | 2680.2 | 2649.3 | 2649.9 | 3151.4 | 2501 | 2803.1 |
|  | Std. | 0 | 181.52 | 95.798 | 15.56 | 0.0084457 | 93.243 | 79.501 | 8.969 | 1.4236 | 1.2627 | 112.58 | 0.18882 | 30.015 |
|  | Median | 2500 | 2783.7 | 2863.3 | 2690.5 | 2644 | 3203.6 | 2955.2 | 2645.9 | 2644.2 | 2647 | 2824.1 | 2500 | 2697.6 |
|  | Rank | 1 | 7 | 11 | 8 | 3 | 13 | 12 | 6 | 4 | 5 | 10 | 2 | 9 |
| C14-F24 | Mean | 2600 | 2601.3 | 2600 | 2700.6 | 2683.7 | 2753.5 | 2979.2 | 2760 | 2600 | 2740.8 | 2813.4 | 2600.5 | 2846.2 |
|  | Best | 2600 | 2600 | 2600 | 2686.6 | 2670.3 | 2666.9 | 2817.4 | 2689.1 | 2600 | 2723.1 | 2690.7 | 2600 | 2734.4 |
|  | Worst | 2600 | 2610.6 | 2600.1 | 2731.2 | 2695.1 | 2777.3 | 3164.4 | 3089.8 | 2600 | 2754.1 | 3014 | 2600.8 | 3201.4 |
|  | Std. | 0.0033213 | 2.6444 | 0.020694 | 8.9247 | 5.4232 | 24.116 | 97.434 | 114.2 | 0.0026951 | 7.5049 | 65.562 | 0.18556 | 129.54 |
|  | Median | 2600 | 2600.2 | 2600 | 2700.5 | 2682.6 | 2758.6 | 2957.3 | 2709.3 | 2600 | 2739.6 | 2803.4 | 2600.5 | 2794 |
|  | Rank | 1 | 5 | 3 | 7 | 6 | 9 | 13 | 10 | 2 | 8 | 11 | 4 | 12 |
| C14-F25 | Mean | 2700 | 2713.3 | 2700 | 2740.1 | 2734.2 | 2788 | 2784.6 | 2738.6 | 2700 | 2805.1 | 2739.1 | 2700 | 2766.8 |
|  | Best | 2700 | 2700 | 2700 | 2722.6 | 2724.3 | 2724 | 2749 | 2710.3 | 2700 | 2749.6 | 2718.2 | 2700 | 2732.2 |
|  | Worst | 2700 | 2745.9 | 2700 | 2756 | 2747.2 | 2837.6 | 2868.2 | 2815.6 | 2700 | 2934.8 | 2771.4 | 2700 | 2856.6 |
|  | Std. | 4.39E-13 | 19.262 | 3.11E-05 | 7.8687 | 5.8313 | 28.472 | 31.182 | 25.039 | 3.78E-13 | 42.085 | 13.965 | 0 | 28.002 |
|  | Median | 2700 | 2700 | 2700 | 2740.5 | 2733.7 | 2788.3 | 2774.5 | 2735.6 | 2700 | 2798.9 | 2737.1 | 2700 | 2761.2 |
|  | Rank | 1 | 5 | 4 | 9 | 6 | 12 | 11 | 7 | 3 | 13 | 8 | 2 | 10 |
| C14-F26 | Mean | 2700.6 | 2717.1 | 2815.6 | 2770.1 | 2780.4 | 2724.1 | 2833 | 2792.1 | 2780.2 | 2827.7 | 2753.4 | 2797.5 | 2798.5 |
|  | Best | 2700.5 | 2700.4 | 2702.7 | 2700.3 | 2700.4 | 2705.5 | 2806.8 | 2700.5 | 2700.5 | 2700.8 | 2704.1 | 2724.9 | 2700.4 |
|  | Worst | 2700.8 | 2800 | 3034.5 | 2800 | 2801.2 | 3071 | 3094.5 | 3062.8 | 2800.2 | 3060.4 | 3048.7 | 2800 | 2803.3 |
|  | Std. | 0.092847 | 37.72 | 73.288 | 46.401 | 40.619 | 71.879 | 65.948 | 85.358 | 40.465 | 169.42 | 115.67 | 13.706 | 18.523 |
|  | Median | 2700.6 | 2700.5 | 2800 | 2800 | 2800.3 | 2706.6 | 2813.5 | 2800.8 | 2800 | 2701.1 | 2705.5 | 2800 | 2801.8 |
|  | Rank | 1 | 2 | 11 | 5 | 7 | 3 | 13 | 8 | 6 | 12 | 4 | 9 | 10 |
| C14-F27 | Mean | 3726.6 | 4901.8 | 4361.3 | 4246.6 | 4067.3 | 4889.7 | 5152.7 | 4592.5 | 4281.1 | 4188.4 | 4279.5 | 5578.7 | 4779.2 |
|  | Best | 3117.5 | 3611.6 | 4159.6 | 4032.3 | 3112.1 | 4662 | 4818.7 | 4307.6 | 4086.3 | 3708.4 | 4050.7 | 4948.7 | 4476 |
|  | Worst | 3990.1 | 5179.8 | 4581.3 | 4622.8 | 4516.8 | 5069.8 | 5445.7 | 5140.8 | 4471.7 | 4619.5 | 4505.3 | 6382 | 4990.3 |
|  | Std. | 251.65 | 278.04 | 112.13 | 152.68 | 363.11 | 83.013 | 162.61 | 191.47 | 109.19 | 233.78 | 115.39 | 383.54 | 129.15 |
|  | Median | 3797.6 | 4933.8 | 4361.6 | 4221.8 | 4181.2 | 4883.6 | 5153.7 | 4549.5 | 4282.3 | 4184.8 | 4292.3 | 5452.1 | 4778.1 |
|  | Rank | 1 | 11 | 7 | 4 | 2 | 10 | 12 | 8 | 6 | 3 | 5 | 13 | 9 |

**Table S3.** (continued)

| C14-F28 | Mean | 3000 | 9211.9 | 8027.3 | 5649.6 | 8899.3 | 9990.9 | 13275 | 11409 | 5633.6 | 3978 | 4884.6 | 9547.1 | 12805 |
| --- | --- | --- | --- | --- | --- | --- | --- | --- | --- | --- | --- | --- | --- | --- |
|  | Best | 3000 | 5869.1 | 6143.5 | 4547.5 | 6821.5 | 8137 | 8793.4 | 8232.7 | 4222.3 | 3901 | 4211.9 | 3000 | 10030 |
|  | Worst | 3000 | 13046 | 11116 | 7750.6 | 11202 | 11705 | 16164 | 14129 | 7188.1 | 4144.4 | 6533.7 | 18926 | 17602 |
|  | Std. | 1.39E-12 | 1712.2 | 1231 | 890.51 | 1308.9 | 824.41 | 1652.1 | 1582.9 | 663.38 | 59.437 | 587.39 | 7170.1 | 1650.8 |
|  | Median | 3000 | 9450.6 | 7846.3 | 5493.3 | 9047.7 | 9976.5 | 13300 | 11258 | 5617.5 | 3959.5 | 4620.9 | 3000 | 12862 |
|  | Rank | 1 | 8 | 6 | 5 | 7 | 10 | 13 | 11 | 4 | 2 | 3 | 9 | 12 |
| C14-F29 | Mean | 1.31E+06 | 9.74E+07 | 9.52E+07 | 4.69E+07 | 5033.5 | 3.09E+08 | 2.37E+08 | 4.48E+06 | 5.88E+07 | 2.50E+07 | 3.71E+07 | 1.57E+09 | 9.19E+07 |
|  | Best | 9837.8 | 2.42E+06 | 32912 | 39068 | 3699.9 | 1.99E+08 | 40443 | 12396 | 8784.2 | 6523.9 | 1.23E+06 | 3100 | 13270 |
|  | Worst | 3.85E+07 | 1.86E+08 | 3.70E+08 | 2.89E+08 | 6319.4 | 4.00E+08 | 8.23E+08 | 6.45E+07 | 1.90E+08 | 3.84E+07 | 5.11E+07 | 2.51E+09 | 3.10E+08 |
|  | Std. | 7.02E+06 | 4.99E+07 | 8.55E+07 | 7.63E+07 | 640.29 | 5.28E+07 | 2.20E+08 | 1.54E+07 | 4.70E+07 | 1.66E+07 | 1.02E+07 | 6.25E+08 | 1.19E+08 |
|  | Median | 27552 | 1.08E+08 | 6.24E+07 | 1.22E+05 | 5054.2 | 2.98E+08 | 1.87E+08 | 47272 | 6.03E+07 | 3.52E+07 | 3.89E+07 | 1.65E+09 | 9.13E+05 |
|  | Rank | 2 | 10 | 9 | 6 | 1 | 12 | 11 | 3 | 7 | 4 | 5 | 13 | 8 |
| C14-F30 | Mean | 13632 | 1.21E+06 | 1.01E+06 | 99557 | 20659 | 4.09E+06 | 2.50E+06 | 24813 | 22984 | 13632 | 1.45E+05 | 3.42E+07 | 1.33E+05 |
|  | Best | 11341 | 1.94E+05 | 2.23E+05 | 26457 | 15089 | 1.57E+06 | 99710 | 15367 | 14221 | 11341 | 28848 | 3200 | 33997 |
|  | Worst | 17161 | 5.95E+06 | 2.73E+06 | 2.82E+05 | 27616 | 7.10E+06 | 7.61E+06 | 59951 | 73831 | 17161 | 5.41E+05 | 6.12E+07 | 8.10E+05 |
|  | Std. | 1710 | 1.17E+06 | 7.31E+05 | 52513 | 2944.6 | 1.50E+06 | 2.17E+06 | 13086 | 12026 | 1710 | 1.13E+05 | 1.63E+07 | 1.47E+05 |
|  | Median | 13248 | 7.45E+05 | 6.89E+05 | 85453 | 20601 | 3.77E+06 | 2.00E+06 | 18250 | 18487 | 13248 | 1.07E+05 | 3.32E+07 | 99292 |
|  | Rank | 1 | 10 | 9 | 6 | 3 | 12 | 11 | 5 | 4 | 2 | 8 | 13 | 7 |
| Sum rank | | 144 | 256 | 223 | 144 | 101 | 332 | 290 | 185 | 135 | 240 | 238 | 325 | 225 |
| Mean rank | | 1.2000 | 8.5333 | 7.4333 | 4.8000 | 3.3666 | 11.0666 | 9.6666 | 6.1666 | 4.50000 | 8 | 7.9333 | 10.8333 | 7.5000 |
| Total rank | | 1 | 10 | 6 | 4 | 2 | 13 | 11 | 5 | 3 | 9 | 8 | 12 | 7 |

**Table S3.** (continued)

|  |  |
| --- | --- |
|  |  |
|  |  |
|  |  |

**Figure S6.** Boxplot illustrating the performance of the HO in comparison to competing algorithms for optimizing CEC 2014 (D = 50).

|  |  |
| --- | --- |
|  |  |
|  |  |
|  |  |

**Figure S6.** (continued)

|  |  |
| --- | --- |
|  |  |
|  |  |
|  |  |

**Figure S6.** (continued)

|  |  |
| --- | --- |
|  |  |
|  |  |

**Figure S6.** (continued)

|  |  |
| --- | --- |
|  |  |
|  |  |
|  |  |

**Figure S7.** Convergence curves of the top three algorithms in each function in CEC 2014 (D = 50).

|  |  |
| --- | --- |
|  |  |
|  |  |
|  |  |

**Figure S7.** (continued)

|  |  |
| --- | --- |
|  |  |
|  |  |
|  |  |

**Figure S7.** (continued)

|  |  |
| --- | --- |
|  |  |
|  |  |

**Figure S7.** (continued)

Evaluation outcomes for the objectives specified in the CEC 2014 (D =100).

| **F** | **M** | **Optimization Algorithms** | | | | | | | | | | | | |
| --- | --- | --- | --- | --- | --- | --- | --- | --- | --- | --- | --- | --- | --- | --- |
|  |  | **HO** | **WOA** | **GWO** | **SSA** | **PSO** | **SCA** | **FA** | **GOA** | **TLBO** | **CMA-ES** | **MFO** | **AOA** | **IWO** |
| C14-F1 | Mean | 7.04E+07 | 1.11E+09 | 8.91E+08 | 2.74E+08 | 1.01E+08 | 4.60E+09 | 2.29E+09 | 8.75E+07 | 1.38E+08 | 6.02E+09 | 1.08E+09 | 7.32E+09 | 3.62E+08 |
|  | Best | 4.27E+07 | 7.03E+08 | 3.96E+08 | 1.67E+08 | 5.52E+07 | 3.10E+09 | 1.58E+09 | 3.09E+07 | 7.10E+07 | 3.64E+09 | 4.92E+08 | 4.64E+09 | 1.36E+08 |
|  | Worst | 1.17E+08 | 1.91E+09 | 1.52E+09 | 4.04E+08 | 1.70E+08 | 7.16E+09 | 4.12E+09 | 2.79E+08 | 2.91E+08 | 9.08E+09 | 2.47E+09 | 1.16E+10 | 6.94E+08 |
|  | Std. | 1.73E+07 | 2.66E+08 | 2.85E+08 | 6.10E+07 | 2.81E+07 | 1.10E+09 | 5.61E+08 | 4.77E+07 | 4.97E+07 | 1.38E+09 | 4.22E+08 | 1.67E+09 | 1.37E+08 |
|  | Median | 6.60E+07 | 1.10E+09 | 8.46E+08 | 2.80E+08 | 9.76E+07 | 4.60E+09 | 2.16E+09 | 7.30E+07 | 1.29E+08 | 5.86E+09 | 1.04E+09 | 7.10E+09 | 3.50E+08 |
|  | Rank | 1 | 9 | 7 | 5 | 3 | 11 | 10 | 2 | 4 | 12 | 8 | 13 | 6 |
| C14-F2 | Mean | 9.05E+07 | 5.69E+10 | 1.19E+11 | 6.14E+08 | 1.25E+08 | 2.19E+11 | 2.37E+11 | 1.28E+10 | 1.19E+10 | 9.89E+10 | 1.41E+11 | 3.07E+11 | 6.90E+10 |
|  | Best | 2.88E+07 | 3.94E+10 | 9.20E+10 | 2.53E+08 | 9.84E+06 | 1.88E+11 | 1.78E+11 | 1.59E+08 | 2.83E+09 | 7.72E+10 | 4.90E+10 | 2.74E+11 | 4.46E+10 |
|  | Worst | 1.94E+08 | 7.94E+10 | 1.45E+11 | 1.20E+09 | 1.16E+09 | 2.56E+11 | 2.72E+11 | 3.80E+10 | 2.47E+10 | 1.19E+11 | 2.62E+11 | 3.29E+11 | 1.33E+11 |
|  | Std. | 4.27E+07 | 8.97E+09 | 1.51E+10 | 2.55E+08 | 2.22E+08 | 1.64E+10 | 2.21E+10 | 1.02E+10 | 5.49E+09 | 9.71E+09 | 5.40E+10 | 1.44E+10 | 1.93E+10 |
|  | Median | 7.75E+07 | 5.55E+10 | 1.21E+11 | 5.47E+08 | 4.32E+07 | 2.15E+11 | 2.40E+11 | 1.00E+10 | 1.04E+10 | 1.00E+11 | 1.41E+11 | 3.09E+11 | 6.59E+10 |
|  | Rank | 1 | 6 | 9 | 3 | 2 | 11 | 12 | 5 | 4 | 8 | 10 | 3 | 7 |
| C14-F3 | Mean | 41040 | 4.64E+05 | 2.37E+05 | 3.29E+05 | 72663 | 3.53E+05 | 4.79E+05 | 98583 | 1.52E+05 | 6.12E+05 | 3.74E+05 | 2.97E+05 | 5.90E+05 |
|  | Best | 30656 | 2.90E+05 | 2.01E+05 | 2.16E+05 | 20345 | 2.98E+05 | 3.86E+05 | 53710 | 80512 | 4.67E+05 | 1.30E+05 | 2.68E+05 | 3.95E+05 |
|  | Worst | 49642 | 7.83E+05 | 2.80E+05 | 5.00E+05 | 1.56E+05 | 4.16E+05 | 6.88E+05 | 1.59E+05 | 2.22E+05 | 7.29E+05 | 7.18E+05 | 3.15E+05 | 7.89E+05 |
|  | Std. | 5484.7 | 1.38E+05 | 20897 | 58636 | 34161 | 31757 | 60127 | 31034 | 27700 | 58089 | 1.80E+05 | 11522 | 94635 |
|  | Median | 40705 | 4.42E+05 | 2.38E+05 | 3.37E+05 | 62330 | 3.56E+05 | 4.77E+05 | 96323 | 1.52E+05 | 6.09E+05 | 3.97E+05 | 2.99E+05 | 5.91E+05 |
|  | Rank | 1 | 10 | 5 | 7 | 2 | 8 | 11 | 3 | 4 | 13 | 9 | 6 | 12 |
| C14-F4 | Mean | 841.92 | 7681.1 | 13576 | 1006.9 | 1008.8 | 46784 | 51006 | 2097.5 | 1980.8 | 11257 | 25569 | 90128 | 7448.7 |
|  | Best | 709.69 | 4470.5 | 8370.8 | 833.24 | 876.14 | 32005 | 37227 | 883.42 | 1244.3 | 8006.4 | 8257.3 | 63313 | 3322.3 |
|  | Worst | 987.41 | 11197 | 23629 | 1231.5 | 1204.2 | 64000 | 70412 | 6797.1 | 3820.7 | 14491 | 67685 | 1.11E+05 | 12208 |
|  | Std. | 63.855 | 1786.4 | 3546.5 | 106.6 | 80.997 | 7289.5 | 9429.8 | 1416.5 | 571.32 | 1374 | 15775 | 13659 | 2561.3 |
|  | Median | 840.73 | 7102.7 | 13171 | 992.36 | 999.55 | 46926 | 51547 | 1558.7 | 1792.3 | 11141 | 20968 | 89489 | 7173 |
|  | Rank | 1 | 7 | 9 | 2 | 3 | 11 | 12 | 5 | 4 | 8 | 10 | 13 | 6 |
| C14-F5 | Mean | 520.31 | 521.23 | 521.15 | 520.17 | 521.27 | 521.38 | 520 | 520.09 | 521.37 | 521.37 | 520.4 | 521.38 | 520.06 |
|  | Best | 520.19 | 521.05 | 521.01 | 520.03 | 520.92 | 521.29 | 520 | 520.02 | 521.29 | 521.27 | 520.12 | 521.33 | 520.03 |
|  | Worst | 520.47 | 521.36 | 521.29 | 520.46 | 521.41 | 521.42 | 520.02 | 520.17 | 521.4 | 521.41 | 520.64 | 521.43 | 520.1 |
|  | Std. | 0.071693 | 0.076098 | 0.059427 | 0.10972 | 0.11598 | 0.031859 | 0.0042866 | 0.041352 | 0.027778 | 0.027505 | 0.11749 | 0.024565 | 0.020749 |
|  | Median | 520.3 | 521.24 | 521.14 | 520.13 | 521.31 | 521.39 | 520 | 520.08 | 521.37 | 521.38 | 520.4 | 521.38 | 520.06 |
|  | Rank | 5 | 8 | 7 | 4 | 9 | 12 | 1 | 3 | 10 | 11 | 6 | 13 | 2 |
| C14-F6 | Mean | 696.79 | 756.23 | 720.55 | 719.41 | 703.57 | 758.01 | 739.25 | 733.69 | 714.26 | 760.43 | 717.91 | 755.77 | 730.95 |
|  | Best | 682.75 | 741.07 | 708.79 | 695.4 | 683.77 | 748.81 | 729.58 | 722.68 | 703.41 | 748.48 | 703.57 | 750.08 | 713.4 |
|  | Worst | 710.09 | 767.26 | 731.68 | 756.86 | 724.9 | 766.46 | 752.39 | 743.13 | 724.63 | 767.13 | 731.91 | 763.4 | 749.64 |
|  | Std. | 6.4484 | 6.1071 | 6.5444 | 11.753 | 8.8118 | 4.2204 | 5.298 | 5.3488 | 5.0919 | 3.9655 | 6.7041 | 3.4413 | 7.7114 |
|  | Median | 696.66 | 756.6 | 720.51 | 720.01 | 704.73 | 758.82 | 739.96 | 734.02 | 714.07 | 761.18 | 718.56 | 755.9 | 730.84 |
|  | Rank | 1 | 11 | 6 | 5 | 2 | 12 | 9 | 8 | 3 | 13 | 4 | 10 | 7 |

**Table S4.** Evaluation outcomes for the objectives specified in the CEC 2014 (D = 100).

| C14-F7 | Mean | 701.64 | 1222.9 | 1797.2 | 701.75 | 707.13 | 2850.9 | 3052.4 | 804.39 | 812.25 | 1524.2 | 2193.6 | 3753.8 | 1338.1 |
| --- | --- | --- | --- | --- | --- | --- | --- | --- | --- | --- | --- | --- | --- | --- |
|  | Best | 701.04 | 1090.9 | 1474.6 | 701.25 | 703.51 | 2620 | 2243 | 702.91 | 746.15 | 1376.9 | 1455.6 | 3054.3 | 1077.9 |
|  | Worst | 703.87 | 1521.8 | 2171.6 | 703.37 | 715.55 | 3309.6 | 3470.8 | 949.74 | 885.93 | 1657.1 | 3687 | 4065.9 | 1642 |
|  | Std. | 0.63596 | 87.886 | 157.84 | 0.43412 | 2.579 | 156.13 | 248.92 | 80.616 | 39.25 | 61.575 | 526.92 | 216.88 | 121.47 |
|  | Median | 701.38 | 1220.8 | 1771.5 | 701.63 | 706.71 | 2828.6 | 3049.8 | 790.19 | 800.92 | 1528.4 | 2074.1 | 3780.6 | 1349.6 |
|  | Rank | 1 | 6 | 9 | 2 | 3 | 11 | 12 | 4 | 5 | 8 | 10 | 13 | 7 |
| C14-F8 | Mean | 1249.5 | 1815.1 | 1727.5 | 1503.3 | 1373.7 | 2148 | 1970.3 | 1921.2 | 1328.1 | 1951.4 | 1697 | 2296.1 | 2018.8 |
|  | Best | 1183.9 | 1610.4 | 1546.9 | 1389.9 | 1216.6 | 2007 | 1817.8 | 1756.1 | 1243.8 | 1888.2 | 1503.9 | 2207.8 | 1746.6 |
|  | Worst | 1362.6 | 2043 | 1868.2 | 1640.1 | 1494.3 | 2248 | 2146.3 | 2116.8 | 1470 | 1994.4 | 1886.6 | 2386.5 | 2288 |
|  | Std. | 39.727 | 97.095 | 61.451 | 52.158 | 72.99 | 63.874 | 93.866 | 105.74 | 47.675 | 25.487 | 102.39 | 41.475 | 134.62 |
|  | Median | 1237.9 | 1796.4 | 1728.4 | 1499.4 | 1377.7 | 2161.1 | 1970.5 | 1910 | 1328.6 | 1951.5 | 1682.6 | 2289.1 | 2047.3 |
|  | Rank | 1 | 7 | 6 | 4 | 3 | 12 | 10 | 8 | 2 | 9 | 5 | 13 | 11 |
| C14-F9 | Mean | 1563.9 | 2087.1 | 1831.9 | 1760.4 | 1578.5 | 2361.7 | 2504.2 | 2456 | 1572.2 | 2215.8 | 2238.5 | 2348.8 | 2566.7 |
|  | Best | 1424.3 | 1934.6 | 1696.9 | 1474.9 | 1497.8 | 2230.2 | 2302.1 | 2110.6 | 1409.9 | 2141 | 1905.7 | 2210.2 | 2215.7 |
|  | Worst | 1736.5 | 2362.4 | 2067 | 1983.1 | 1673 | 2512 | 2738.6 | 2658.2 | 1804.8 | 2285.3 | 2500 | 2435.9 | 2854.1 |
|  | Std. | 83.916 | 114.52 | 81.158 | 114.63 | 49.806 | 65.633 | 122.73 | 117.56 | 93.336 | 37.277 | 165.06 | 55.485 | 150.34 |
|  | Median | 1550.7 | 2075.5 | 1814 | 1770.5 | 1583.1 | 2356.3 | 2476.7 | 2455.5 | 1550.5 | 2217 | 2232.6 | 2355.9 | 2599.7 |
|  | Rank | 1 | 6 | 5 | 4 | 3 | 10 | 12 | 11 | 2 | 7 | 8 | 9 | 13 |
| C14-F10 | Mean | 10145 | 24381 | 21310 | 16483 | 13654 | 31494 | 16712 | 14955 | 15995 | 31030 | 15735 | 30479 | 16614 |
|  | Best | 7955 | 20678 | 17833 | 12832 | 12025 | 28649 | 13943 | 12697 | 12050 | 30082 | 10097 | 27959 | 13157 |
|  | Worst | 11957 | 28226 | 24872 | 20862 | 16521 | 33304 | 19808 | 18135 | 22460 | 32347 | 23281 | 32195 | 18929 |
|  | Std. | 930.57 | 1883 | 1797.4 | 1807.6 | 1201.4 | 1106.6 | 1486.8 | 1593.3 | 2432.7 | 527.65 | 2948.3 | 1134.6 | 1402.9 |
|  | Median | 10150 | 24537 | 21313 | 16125 | 13463 | 31776 | 16470 | 14982 | 15860 | 31106 | 15216 | 30707 | 16695 |
|  | Rank | 1 | 10 | 9 | 6 | 2 | 13 | 8 | 3 | 5 | 12 | 4 | 11 | 7 |
| C14-F11 | Mean | 15080 | 27962 | 20635 | 17432 | 15787 | 33147 | 16146 | 16282 | 31761 | 32774 | 18354 | 30061 | 16200 |
|  | Best | 13558 | 20350 | 18042 | 14613 | 13965 | 31305 | 12921 | 13852 | 29257 | 31479 | 14510 | 27661 | 13799 |
|  | Worst | 18302 | 33227 | 24750 | 20449 | 18481 | 34168 | 18051 | 18722 | 33458 | 33682 | 21000 | 31792 | 19219 |
|  | Std. | 1307.2 | 2579.5 | 1445.5 | 1746.3 | 1102.4 | 621.83 | 1153 | 1329 | 912.72 | 558.19 | 1727.6 | 1275 | 1260 |
|  | Median | 14690 | 27810 | 20689 | 17336 | 15575 | 33234 | 16300 | 16595 | 31944 | 32969 | 18439 | 30580 | 15978 |
|  | Rank | 1 | 9 | 8 | 6 | 2 | 13 | 3 | 5 | 11 | 12 | 7 | 10 | 4 |
| C14-F12 | Mean | 1200.2 | 1203.8 | 1202.2 | 1201.8 | 1201.4 | 1204.7 | 1200.3 | 1200.5 | 1204.6 | 1204.7 | 1200.9 | 1204.5 | 1200.4 |
|  | Best | 1200.1 | 1202.8 | 1201.4 | 1201 | 1200.8 | 1203.5 | 1200.1 | 1200.2 | 1204 | 1203.7 | 1200.5 | 1203.4 | 1200.2 |
|  | Worst | 1200.3 | 1204.7 | 1204.3 | 1203.2 | 1202.2 | 1205.4 | 1200.5 | 1200.9 | 1204.9 | 1205.2 | 1201.5 | 1205.1 | 1200.6 |
|  | Std. | 0.046223 | 0.53731 | 0.59877 | 0.43509 | 0.37963 | 0.36356 | 0.089485 | 0.19104 | 0.24805 | 0.30228 | 0.22461 | 0.38496 | 0.092041 |
|  | Median | 1200.1 | 1203.8 | 1202 | 1201.7 | 1201.4 | 1204.7 | 1200.3 | 1200.5 | 1204.6 | 1204.7 | 1200.9 | 1204.5 | 1200.4 |
|  | Rank | 1 | 9 | 8 | 7 | 6 | 13 | 2 | 4 | 11 | 12 | 5 | 10 | 3 |
| C14-F13 | Mean | 1300.6 | 1303.7 | 1305.6 | 1300.6 | 1300.6 | 1307.6 | 1308 | 1300.9 | 1300.7 | 1304.9 | 1306.3 | 1309.5 | 1304.3 |
|  | Best | 1300.4 | 1303 | 1304.9 | 1300.5 | 1300.4 | 1307.1 | 1306.8 | 1300.5 | 1300.5 | 1304.6 | 1304.2 | 1308.2 | 1303 |
|  | Worst | 1300.7 | 1304.2 | 1306.3 | 1300.8 | 1300.8 | 1308.1 | 1309 | 1303.8 | 1300.8 | 1305.4 | 1308.2 | 1310.1 | 1305 |
|  | Std. | 0.0642 | 0.27384 | 0.3739 | 0.077983 | 0.075127 | 0.27386 | 0.57639 | 0.72743 | 0.083142 | 0.22741 | 1.1314 | 0.44959 | 0.53243 |
|  | Median | 1300.6 | 1303.7 | 1305.5 | 1300.6 | 1300.6 | 1307.6 | 1308 | 1300.7 | 1300.7 | 1304.9 | 1306.4 | 1309.6 | 1304.4 |
|  | Rank | 1 | 6 | 9 | 3 | 2 | 11 | 12 | 5 | 4 | 8 | 10 | 13 | 7 |

**Table S4.** (continued)

| C14-F14 | Mean | 1400.3 | 1535.9 | 1713.9 | 1400.4 | 1400.4 | 2005.2 | 2067.1 | 1425.2 | 1422.6 | 1645.1 | 1823.6 | 2281.2 | 1604.2 |
| --- | --- | --- | --- | --- | --- | --- | --- | --- | --- | --- | --- | --- | --- | --- |
|  | Best | 1400.2 | 1503.1 | 1620.1 | 1400.3 | 1400.3 | 1932.8 | 1906.9 | 1400.2 | 1400.3 | 1594.5 | 1544.4 | 2114.8 | 1529.6 |
|  | Worst | 1401 | 1601 | 1784.2 | 1400.8 | 1401 | 2102.4 | 2236 | 1496.7 | 1484.1 | 1676 | 2056.9 | 2393.8 | 1669.9 |
|  | Std. | 0.12729 | 18.832 | 46.024 | 0.15871 | 0.18204 | 40.092 | 72.038 | 29.949 | 22.248 | 19.245 | 153.58 | 62.678 | 39.658 |
|  | Median | 1400.3 | 1534 | 1718.2 | 1400.3 | 1400.4 | 1995.5 | 2067.8 | 1411.6 | 1416.7 | 1645.7 | 1860.1 | 2289.2 | 1606.4 |
|  | Rank | 1 | 6 | 9 | 2 | 3 | 11 | 12 | 5 | 4 | 8 | 10 | 13 | 7 |
| C14-F15 | Mean | 1647.2 | 3.05E+05 | 5.78E+05 | 1985.7 | 1788.6 | 6.16E+06 | 7.79E+06 | 19598 | 4131.6 | 4.66E+06 | 6.57E+06 | 2.37E+07 | 1.39E+05 |
|  | Best | 1580 | 93475 | 1.38E+05 | 1695.9 | 1680.7 | 2.17E+06 | 2.33E+06 | 2532.7 | 2126 | 2.47E+06 | 8.16E+05 | 6.89E+06 | 23517 |
|  | Worst | 1828.5 | 7.24E+05 | 1.88E+06 | 2619.3 | 2026.9 | 1.00E+07 | 1.71E+07 | 1.15E+05 | 13999 | 9.50E+06 | 3.15E+07 | 4.53E+07 | 7.67E+05 |
|  | Std. | 57.439 | 1.56E+05 | 3.89E+05 | 217.97 | 79.207 | 2.04E+06 | 3.99E+06 | 27694 | 2234.6 | 1.80E+06 | 7.26E+06 | 1.02E+07 | 1.39E+05 |
|  | Median | 1632.3 | 2.72E+05 | 4.54E+05 | 1929.1 | 1777 | 5.83E+06 | 7.06E+06 | 6583.7 | 3777 | 4.23E+06 | 4.16E+06 | 2.00E+07 | 99189 |
|  | Rank | 1 | 7 | 8 | 3 | 2 | 10 | 12 | 5 | 4 | 9 | 11 | 13 | 6 |
| C14-F16 | Mean | 1643.9 | 1646.8 | 1645.3 | 1645.1 | 1645.2 | 1647.4 | 1645.8 | 1646.5 | 1646.5 | 1647.6 | 1646.1 | 1647 | 1646.3 |
|  | Best | 1641.9 | 1645.2 | 1643.8 | 1643 | 1641.7 | 1646.3 | 1643.8 | 1645.2 | 1645.4 | 1647 | 1644.6 | 1646.5 | 1645.2 |
|  | Worst | 1645.5 | 1647.7 | 1646.5 | 1647 | 1647 | 1647.9 | 1647 | 1647.5 | 1647.3 | 1647.9 | 1647.6 | 1647.5 | 1647.6 |
|  | Std. | 0.92041 | 0.69902 | 0.67686 | 0.99613 | 1.033 | 0.32758 | 0.71383 | 0.64568 | 0.39072 | 0.19876 | 0.79257 | 0.28216 | 0.6463 |
|  | Median | 1644.1 | 1647.1 | 1645.3 | 1644.9 | 1645.1 | 1647.5 | 1645.9 | 1646.5 | 1646.6 | 1647.6 | 1646 | 1647.1 | 1646.3 |
|  | Rank | 1 | 10 | 4 | 2 | 3 | 12 | 5 | 8 | 9 | 13 | 6 | 11 | 7 |
| C14-F17 | Mean | 5.02E+06 | 1.25E+08 | 9.22E+07 | 2.08E+07 | 5.86E+06 | 5.88E+08 | 1.88E+08 | 1.06E+07 | 6.93E+06 | 5.53E+08 | 8.01E+07 | 1.31E+09 | 1.50E+07 |
|  | Best | 1.69E+06 | 6.66E+07 | 2.05E+07 | 5.73E+06 | 2.70E+06 | 2.34E+08 | 4.91E+07 | 3.29E+06 | 2.95E+06 | 3.24E+08 | 2.66E+07 | 6.97E+08 | 4.50E+06 |
|  | Worst | 9.58E+06 | 2.54E+08 | 2.18E+08 | 3.97E+07 | 9.73E+06 | 9.50E+08 | 4.41E+08 | 3.26E+07 | 1.11E+07 | 7.82E+08 | 2.12E+08 | 2.07E+09 | 6.34E+07 |
|  | Std. | 2.11E+06 | 4.59E+07 | 4.28E+07 | 7.80E+06 | 1.99E+06 | 1.80E+08 | 8.73E+07 | 6.55E+06 | 2.46E+06 | 1.35E+08 | 4.38E+07 | 3.87E+08 | 1.22E+07 |
|  | Median | 4.85E+06 | 1.11E+08 | 8.48E+07 | 2.16E+07 | 5.80E+06 | 5.85E+08 | 1.79E+08 | 8.53E+06 | 6.86E+06 | 5.48E+08 | 6.35E+07 | 1.20E+09 | 1.27E+07 |
|  | Rank | 1 | 9 | 8 | 6 | 2 | 12 | 10 | 4 | 3 | 11 | 7 | 13 | 5 |
| C14-F18 | Mean | 3924.3 | 3.93E+08 | 4.88E+09 | 5675.9 | 21795 | 1.19E+10 | 3.82E+09 | 3.91E+07 | 9.89E+05 | 52849 | 6.77E+09 | 3.94E+10 | 7234.8 |
|  | Best | 2201.8 | 9.88E+07 | 1.16E+09 | 3117.4 | 4605.6 | 8.57E+09 | 4.38E+08 | 3477.6 | 2542.1 | 12543 | 1.45E+08 | 2.51E+10 | 3144.3 |
|  | Worst | 9836.7 | 9.51E+08 | 1.03E+10 | 14053 | 81047 | 1.72E+10 | 6.82E+09 | 6.81E+08 | 1.84E+07 | 2.16E+05 | 2.19E+10 | 5.17E+10 | 20115 |
|  | Std. | 2087.4 | 2.09E+08 | 2.55E+09 | 2756.5 | 17853 | 2.43E+09 | 1.72E+09 | 1.34E+08 | 3.78E+06 | 48619 | 5.14E+09 | 6.83E+09 | 3817.6 |
|  | Median | 3223.7 | 3.75E+08 | 4.61E+09 | 4911.9 | 15468 | 1.18E+10 | 3.51E+09 | 17521 | 4424.9 | 32937 | 4.93E+09 | 3.99E+10 | 6470.4 |
|  | Rank | 1 | 8 | 10 | 2 | 4 | 12 | 9 | 7 | 6 | 5 | 11 | 13 | 3 |
| C14-F19 | Mean | 2031.2 | 2686.6 | 2870.8 | 2053.3 | 2046.4 | 4145.6 | 3513.8 | 2156.4 | 2126.7 | 2082.1 | 3104.1 | 9923.1 | 2282.7 |
|  | Best | 1945.4 | 2451.4 | 2379 | 2006.2 | 1984.9 | 3311 | 2691.8 | 2007.6 | 2025.6 | 2057.6 | 2276.2 | 5441.8 | 2081 |
|  | Worst | 2097.1 | 3040 | 3606.8 | 2104.8 | 2116.5 | 5176.7 | 4321.8 | 2592.1 | 2310.1 | 2116.5 | 4179.5 | 13517 | 2613.8 |
|  | Std. | 34.04 | 143.17 | 281.9 | 23.24 | 33.07 | 464.91 | 448 | 150.4 | 71.353 | 14.242 | 458.03 | 1948.9 | 128.63 |
|  | Median | 2028.3 | 2668.4 | 2822.8 | 2051 | 2038.5 | 4107.1 | 3424.2 | 2111.1 | 2121 | 2081.6 | 3007.7 | 9861.1 | 2279.1 |
|  | Rank | 1 | 8 | 9 | 3 | 2 | 12 | 11 | 6 | 5 | 4 | 10 | 13 | 7 |
| C14-F20 | Mean | 25064 | 4.82E+05 | 1.64E+05 | 2.04E+05 | 77391 | 5.01E+05 | 3.18E+05 | 1.41E+05 | 71222 | 6.04E+05 | 5.70E+05 | 7.12E+05 | 2.78E+05 |
|  | Best | 7012.1 | 1.90E+05 | 71102 | 1.01E+05 | 37959 | 2.18E+05 | 1.37E+05 | 64976 | 42622 | 3.57E+05 | 1.51E+05 | 2.10E+05 | 1.78E+05 |
|  | Worst | 44303 | 1.67E+06 | 2.86E+05 | 3.19E+05 | 1.59E+05 | 1.17E+06 | 1.75E+06 | 2.43E+05 | 98785 | 9.40E+05 | 3.75E+06 | 1.17E+06 | 4.75E+05 |
|  | Std. | 9440.9 | 3.30E+05 | 55977 | 55511 | 26412 | 2.21E+05 | 2.81E+05 | 39945 | 16879 | 1.39E+05 | 7.71E+05 | 2.55E+05 | 68798 |
|  | Median | 25664 | 3.79E+05 | 1.51E+05 | 1.99E+05 | 71437 | 4.42E+05 | 2.51E+05 | 1.39E+05 | 70899 | 5.92E+05 | 3.22E+05 | 6.98E+05 | 2.57E+05 |
|  | Rank | 1 | 9 | 5 | 6 | 3 | 10 | 8 | 4 | 2 | 12 | 11 | 13 | 7 |

**Table S4.** (continued)

| C14-F21 | Mean | 1.86E+06 | 8.37E+07 | 4.14E+07 | 1.22E+07 | 3.78E+06 | 2.10E+08 | 6.29E+07 | 7.19E+06 | 3.54E+06 | 2.20E+08 | 4.06E+07 | 3.60E+08 | 4.51E+06 |
| --- | --- | --- | --- | --- | --- | --- | --- | --- | --- | --- | --- | --- | --- | --- |
|  | Best | 2.86E+05 | 2.70E+07 | 8.47E+06 | 2.96E+06 | 1.93E+06 | 1.15E+08 | 1.01E+07 | 1.85E+06 | 1.06E+06 | 7.95E+07 | 9.00E+06 | 1.40E+08 | 1.87E+06 |
|  | Worst | 4.79E+06 | 1.62E+08 | 1.25E+08 | 2.07E+07 | 7.95E+06 | 4.92E+08 | 2.66E+08 | 2.45E+07 | 1.32E+07 | 4.11E+08 | 1.46E+08 | 5.83E+08 | 9.84E+06 |
|  | Std. | 1.03E+06 | 3.10E+07 | 2.47E+07 | 5.36E+06 | 1.61E+06 | 8.27E+07 | 6.24E+07 | 4.47E+06 | 2.25E+06 | 7.89E+07 | 3.63E+07 | 1.27E+08 | 2.29E+06 |
|  | Median | 1.60E+06 | 8.94E+07 | 3.52E+07 | 1.25E+07 | 3.17E+06 | 1.97E+08 | 4.30E+07 | 6.74E+06 | 2.81E+06 | 2.02E+08 | 2.81E+07 | 3.25E+08 | 3.99E+06 |
|  | Rank | 1 | 10 | 8 | 6 | 3 | 11 | 9 | 5 | 2 | 12 | 7 | 13 | 4 |
| C14-F22 | Mean | 4324.6 | 8062.3 | 5639.7 | 4986.8 | 4584.7 | 9128.7 | 7563.7 | 5564.7 | 4852.2 | 7266.5 | 6535.2 | 1.54E+05 | 5495.6 |
|  | Best | 3249.3 | 6350.1 | 4116 | 3494.5 | 3597 | 8137.6 | 5190.7 | 4467.4 | 3487.4 | 6079.1 | 4862.1 | 11828 | 4501.7 |
|  | Worst | 4997.5 | 11257 | 15313 | 6224.5 | 5518.9 | 14676 | 13761 | 6622.1 | 6958.4 | 7885.9 | 10072 | 1.05E+06 | 6764.5 |
|  | Std. | 409.73 | 1262.6 | 1927.3 | 524.07 | 531.05 | 1183.9 | 1540.7 | 574.31 | 658.49 | 360.98 | 1002.6 | 1.97E+05 | 540.44 |
|  | Median | 4426.8 | 7745.9 | 5429.9 | 4998.9 | 4590 | 8940.6 | 7407.4 | 5571.4 | 4704.9 | 7319.4 | 6419.8 | 1.05E+05 | 5425.6 |
|  | Rank | 1 | 11 | 7 | 4 | 2 | 12 | 10 | 6 | 3 | 9 | 8 | 13 | 5 |
| C14-F23 | Mean | 2500 | 2569.2 | 3202.6 | 2815.4 | 2652 | 4156.4 | 3922.5 | 2693.8 | 2524 | 2898.1 | 3655 | 2503.9 | 2947.9 |
|  | Best | 2500 | 2500 | 2500 | 2764.1 | 2649.1 | 3833 | 3516 | 2656.6 | 2500 | 2799.3 | 2826.6 | 2502.4 | 2819.2 |
|  | Worst | 2500 | 3205.4 | 3545.4 | 2918.7 | 2657 | 4802.7 | 4481.6 | 2848.9 | 2696.1 | 2998.8 | 5378.4 | 2505.5 | 3127.9 |
|  | Std. | 0 | 211.18 | 303.35 | 35.014 | 1.9977 | 235.93 | 264.5 | 47.07 | 62.55 | 44.068 | 563.17 | 0.73387 | 87.242 |
|  | Median | 2500 | 2500 | 3271 | 2818 | 2651.9 | 4082.2 | 3892 | 2674.3 | 2500 | 2896.3 | 3534.7 | 2504 | 2939.8 |
|  | Rank | 1 | 4 | 10 | 7 | 5 | 13 | 12 | 6 | 3 | 8 | 11 | 2 | 9 |
| C14-F24 | Mean | 2600 | 2601.9 | 2600.1 | 2878.9 | 2817.7 | 3137.5 | 3749.9 | 2962.6 | 2600 | 3371.2 | 3325.7 | 2601.7 | 3347.8 |
|  | Best | 2600 | 2600 | 2600 | 2844.3 | 2795.4 | 3060.8 | 3471.1 | 2891.1 | 2600 | 3329.8 | 3113.3 | 2601 | 3126 |
|  | Worst | 2600 | 2609.5 | 2600.2 | 2927.7 | 2845.7 | 3290.1 | 4062.2 | 3068 | 2600 | 3421.1 | 3618.8 | 2602.2 | 4067.4 |
|  | Std. | 0.0043134 | 2.7947 | 0.047884 | 20.687 | 13.005 | 55.422 | 149.99 | 45.446 | 0.0028164 | 24.714 | 137.75 | 0.24813 | 190.9 |
|  | Median | 2600 | 2600.7 | 2600.1 | 2872 | 2817.4 | 3125 | 3752.1 | 2959.2 | 2600 | 3372.7 | 3290 | 2601.7 | 3295.6 |
|  | Rank | 1 | 5 | 3 | 7 | 6 | 9 | 13 | 8 | 2 | 12 | 10 | 4 | 11 |
| C14-F25 | Mean | 2700 | 2700 | 2700 | 2811.9 | 2803.1 | 2998.5 | 3020.9 | 2833.8 | 2700 | 3517.9 | 2845.2 | 2700.1 | 2892.1 |
|  | Best | 2700 | 2700 | 2700 | 2768.2 | 2779.9 | 2863.7 | 2919.2 | 2756.2 | 2700 | 3328.2 | 2755.9 | 2700.1 | 2818.4 |
|  | Worst | 2700 | 2700 | 2700 | 2872.1 | 2851.6 | 3185.2 | 3146.1 | 2936.7 | 2700 | 3781.2 | 2986.1 | 2700.2 | 2963.6 |
|  | Std. | 8.44E-14 | 4.22E-13 | 1.72E-12 | 23.225 | 14.486 | 91.279 | 61.488 | 46.674 | 4.55E-13 | 119.45 | 45.735 | 0.028103 | 37.867 |
|  | Median | 2700 | 2700 | 2700 | 2807.4 | 2800.9 | 2980.4 | 3003.7 | 2824.1 | 2700 | 3504.3 | 2836.8 | 2700.1 | 2888.7 |
|  | Rank | 2 | 1 | 4 | 7 | 6 | 11 | 12 | 8 | 3 | 13 | 9 | 5 | 10 |
| C14-F26 | Mean | 2784.8 | 2800 | 2800 | 2800 | 2801.8 | 3042.5 | 2917.6 | 2805.9 | 2800 | 3070.1 | 2901.8 | 2800 | 2811.1 |
|  | Best | 2700.6 | 2800 | 2800 | 2800 | 2800.7 | 2712.2 | 2842.1 | 2802.7 | 2800 | 2704.3 | 2706.8 | 2800 | 2806.1 |
|  | Worst | 2808.7 | 2800 | 2800 | 2800 | 2803 | 3367 | 3061 | 2815.1 | 2801.2 | 3697.2 | 3195.2 | 2800 | 2819.3 |
|  | Std. | 42.817 | 4.05E-13 | 1.43E-11 | 0 | 0.55756 | 276.67 | 41.357 | 2.9004 | 0.21804 | 319.04 | 139.32 | 0.00030347 | 3.4186 |
|  | Median | 2805.2 | 2800 | 2800 | 2800 | 2801.8 | 3017.9 | 2915.3 | 2804.7 | 2800 | 3272.1 | 2869 | 2800 | 2810.5 |
|  | Rank | 1 | 3 | 4 | 2 | 7 | 12 | 11 | 8 | 6 | 13 | 10 | 5 | 9 |
| C14-F27 | Mean | 5173.6 | 7377.5 | 6200.4 | 6109.1 | 5877.7 | 7364.4 | 7864 | 6784.6 | 6216.2 | 6826.5 | 6162.9 | 9206.2 | 6857.1 |
|  | Best | 4831.9 | 6928 | 5893.1 | 5643.7 | 5456.3 | 7138.1 | 6918.9 | 6234 | 5916.1 | 6559.6 | 5777.9 | 7805.9 | 6356.5 |
|  | Worst | 5499.6 | 7788.4 | 6468.6 | 6571.8 | 6430.9 | 7619.7 | 8653 | 7194.5 | 6476.5 | 7048.6 | 6827.4 | 10615 | 7201.1 |
|  | Std. | 174.56 | 224.45 | 166.85 | 239.59 | 194.18 | 117.03 | 380.94 | 225.98 | 145.57 | 112.15 | 210.08 | 814.19 | 229.2 |
|  | Median | 5181.7 | 7421.6 | 6196.8 | 6108.7 | 5890.1 | 7363.9 | 7862.2 | 6772.7 | 6221.5 | 6823.5 | 6148.7 | 9180 | 6848 |
|  | Rank | 1 | 11 | 5 | 3 | 2 | 10 | 12 | 7 | 6 | 8 | 4 | 13 | 9 |

| C14-F28 | Mean | 3000 | 21429 | 18354 | 11369 | 15201 | 25017 | 28294 | 25321 | 10602 | 5621.6 | 8499.9 | 30691 | 29145 |
| --- | --- | --- | --- | --- | --- | --- | --- | --- | --- | --- | --- | --- | --- | --- |
|  | Best | 3000 | 14654 | 15104 | 9129.1 | 9233.4 | 22959 | 19286 | 16319 | 8739.9 | 5481.5 | 6841.4 | 3003.1 | 23345 |
|  | Worst | 3000 | 29255 | 22662 | 13936 | 20701 | 28719 | 33490 | 31038 | 12684 | 5823.2 | 10084 | 43393 | 33793 |
|  | Std. | 2.31E-12 | 4363.5 | 2014.1 | 1337.8 | 2618.9 | 1322.4 | 3228.7 | 2837.6 | 1026 | 83.434 | 894.19 | 15760 | 2569.4 |
|  | Median | 3000 | 20506 | 18435 | 11312 | 14928 | 24680 | 28493 | 25328 | 10429 | 5624.4 | 8420.6 | 37994 | 29145 |
|  | Rank | 1 | 8 | 7 | 5 | 6 | 9 | 11 | 10 | 4 | 2 | 3 | 13 | 12 |
| C14-F29 | Mean | 6819.3 | 7.25E+08 | 6.52E+08 | 3.32E+08 | 5.84E+07 | 1.78E+09 | 1.78E+09 | 3.22E+06 | 2.70E+08 | 5.65E+07 | 1.07E+08 | 2.07E+09 | 3.11E+08 |
|  | Best | 4491.3 | 3100 | 1.96E+08 | 36048 | 34638 | 1.33E+09 | 3.84E+08 | 43056 | 6.46E+07 | 97275 | 6.18E+07 | 3165.3 | 29243 |
|  | Worst | 10506 | 1.37E+09 | 1.14E+09 | 1.92E+09 | 2.48E+08 | 2.43E+09 | 3.09E+09 | 8.54E+07 | 8.41E+08 | 8.71E+07 | 1.71E+08 | 5.81E+09 | 1.58E+09 |
|  | Std. | 1578 | 3.50E+08 | 2.55E+08 | 5.94E+08 | 8.10E+07 | 2.64E+08 | 6.53E+08 | 1.56E+07 | 1.69E+08 | 4.05E+07 | 1.70E+07 | 2.45E+09 | 4.86E+08 |
|  | Median | 6506.9 | 6.96E+08 | 6.33E+08 | 87712 | 1.56E+05 | 1.72E+09 | 1.80E+09 | 1.01E+05 | 2.19E+08 | 8.38E+07 | 1.03E+08 | 1.21E+07 | 5.12E+07 |
|  | Rank | 1 | 10 | 9 | 8 | 4 | 12 | 11 | 2 | 6 | 3 | 5 | 13 | 7 |
| C14-F30 | Mean | 55253 | 1.64E+07 | 1.37E+07 | 1.32E+06 | 75063 | 4.42E+07 | 2.36E+07 | 1.12E+05 | 77582 | 3.74E+05 | 4.89E+06 | 2.32E+08 | 1.59E+06 |
|  | Best | 4491.3 | 3100 | 1.96E+08 | 36048 | 34638 | 1.33E+09 | 3.84E+08 | 43056 | 6.46E+07 | 97275 | 6.18E+07 | 3165.3 | 29243 |
|  | Worst | 91777 | 4.33E+07 | 3.17E+07 | 2.59E+06 | 2.21E+05 | 7.54E+07 | 6.41E+07 | 3.88E+05 | 1.88E+05 | 6.75E+05 | 2.86E+07 | 5.51E+08 | 5.13E+06 |
|  | Std. | 14884 | 9.89E+06 | 7.20E+06 | 5.98E+05 | 47985 | 1.41E+07 | 1.64E+07 |  | 39655 | 1.04E+05 | 5.54E+06 | 1.47E+08 | 1.11E+06 |
|  | Median | 53257 | 1.39E+07 | 1.33E+07 | 1.21E+06 | 63135 | 4.07E+07 | 1.84E+07 | 67419 | 68984 | 3.63E+05 | 2.91E+06 | 2.63E+08 | 1.15E+06 |
|  | Rank | 1 | 10 | 9 | 6 | 2 | 12 | 11 | 4 | 3 | 5 | 8 | 13 | 7 |
| Sum rank | | 35 | 234 | 216 | 137 | 104 | 338 | 293 | 169 | 140 | 280 | 237 | 320 | 217 |
| Mean rank | | 1.1666 | 7.8000 | 7.2000 | 4.5666 | 3.4666 | 11.2666 | 9.7666 | 5.6333 | 4.6666 | 9.3333 | 7.9000 | 10.6666 | 7.2333 |
| Total rank | | 1 | 8 | 6 | 3 | 2 | 13 | 11 | 5 | 4 | 10 | 9 | 12 | 7 |

**Table S4.** (continued)

|  |  |
| --- | --- |
|  |  |
|  |  |
|  |  |

**Figure S8.** Boxplot illustrating the performance of the HO in comparison to competing algorithms for optimizing CEC 2014 (D = 100).

|  |  |
| --- | --- |
|  |  |
|  |  |
|  |  |

**Figure S8.** (continued)

|  |  |
| --- | --- |
|  |  |
|  |  |
|  |  |

**Figure S8.** (continued)

|  |  |
| --- | --- |
|  |  |
|  |  |

**Figure S8.** (continued)

|  |  |
| --- | --- |
|  |  |
|  |  |
|  |  |

**Figure S9.** Convergence curves of the top three algorithms in each function in CEC 2014 (D = 100).

|  |  |
| --- | --- |
|  |  |
|  |  |
|  |  |

**Figure S9.** (continued)

|  |  |
| --- | --- |
|  |  |
|  |  |
|  |  |

**Figure S9.** (continued)

|  |  |
| --- | --- |
|  |  |
|  |  |

**Figure S9.** (continued)

|  |  |
| --- | --- |
|  |  |

**Figure S10.** Lévy flight (a) distribution, (b) 2D trajectory, (c) 3D trajectory

**The real-world problems**

The engineering design problems are formulated as follows:

**TCS**

**------------------------------------------------------------------------------------------------------------------------------------------------------**

Consider: $\mathcal{Z=}\left[ \mathcal{z}_{1},\mathcal{z}_{2},\mathcal{z}_{3} \right]\mathcal{=[d,D,P]}$

Minimize: $\mathcal{F}\left( \mathcal{Z} \right)=\left( \mathcal{z}_{3}+2 \right)\mathcal{z}_{2}\mathcal{z}_{1}^{2}$

Subject to: $\left\{ \begin{aligned} \mathcal{g}_{1}\left( \mathcal{Z} \right)=1-\frac{\mathcal{z}_{3}\mathcal{z}_{2}^{3}}{71785\mathcal{z}_{1}^{4}}\leq0 \\ \mathcal{g}_{2}\left( \mathcal{Z} \right)=\frac{4\mathcal{z}_{2}^{2}-\mathcal{z}_{2}\mathcal{z}_{1}}{12566\left( \mathcal{z}_{2}\mathcal{z}_{1}^{3} \right)}+\frac{1}{5108\mathcal{z}_{1}^{2}}-1\leq0 \\ \mathcal{g}_{3}\left( \mathcal{Z} \right)=1-\frac{140.45\mathcal{z}_{1}}{\mathcal{z}_{3}\mathcal{z}_{2}^{2}}\leq0 \\ \mathcal{g}_{4}\left( \mathcal{Z} \right)=\frac{\mathcal{z}_{1}+\mathcal{z}_{2}}{1.5}-1\leq0 \end{aligned} \right.$

With. $0.05\leq\mathcal{z}_{1}\leq2, 0.25\leq\mathcal{z}_{2}\leq1.3, 2\leq\mathcal{z}_{3}<15$

**WB**

**------------------------------------------------------------------------------------------------------------------------------------------------------**Consider: $\mathcal{Z=}\left[ \mathcal{z}_{1},\mathcal{z}_{2},\mathcal{z}_{3},\mathcal{z}_{4} \right]\mathcal{=[h,l,t,b]}$

Minimize: $\mathcal{F}\left( \mathcal{Z} \right)=1.1047\mathcal{z}_{2}\mathcal{z}_{1}^{2}+0.0481\mathcal{z}_{3}\mathcal{z}_{4}\left( 14+\mathcal{z}_{2} \right)$

Subject to: $\left\{ \begin{aligned} \mathcal{g}_{1}\left( \mathcal{Z} \right)=\tau\left( \mathcal{Z} \right)-13600\leq0 \\ \mathcal{g}_{2}\left( \mathcal{Z} \right)=\sigma\left( \mathcal{Z} \right)-30000\leq0 \\ \mathcal{g}_{3}\left( \mathcal{Z} \right)=\mathcal{z}_{1}-\mathcal{z}_{4}\leq0 \\ \mathcal{g}_{4}\left( \mathcal{Z} \right)=0.10471\mathcal{z}_{1}^{2}+0.0481\mathcal{z}_{3}\mathcal{z}_{4}\left( 14+\mathcal{z}_{2} \right)-5\leq0 \\ \mathcal{g}_{5}\left( \mathcal{Z} \right)=0.125-\mathcal{z}_{1}\leq0 \\ \mathcal{g}_{6}\left( \mathcal{Z} \right)=\delta\left( \mathcal{Z} \right)-0.25\leq0 \\ \mathcal{g}_{7}\left( \mathcal{Z} \right)=6000-\mathcal{P}_{c}\left( \mathcal{Z} \right)\leq0 \end{aligned} \right.$

Where: $\left\{ \begin{aligned} \tau\left( \mathcal{Z} \right)=\sqrt{\left( \tau^{¤} \right)^{2}+\left( 2\tau\tau^{¤} \right)\frac{\mathcal{z}_{2}}{2R}+\left( \tau^{¤¤} \right)^{2}}, \tau^{¤}=\frac{6000}{\sqrt{2}\mathcal{z}_{1}\mathcal{z}_{2}}, \tau^{¤¤}=\frac{MR}{J} \\ M=6000(14+\frac{\mathcal{z}_{2}}{2}) \\ R=\sqrt{\frac{\mathcal{z}_{2}^{2}}{4}+\left( \frac{\mathcal{z}_{1}+\mathcal{z}_{3}}{2} \right)^{2}} \\ J=2\sqrt{2}\mathcal{z}_{1}\mathcal{z}_{2}\left( \frac{\mathcal{z}_{2}^{2}}{12}+\left( \frac{\mathcal{z}_{1}+\mathcal{z}_{3}}{2} \right) \right)^{2} \\ \sigma\left( \mathcal{Z} \right)=\frac{504000}{\mathcal{z}_{4}\mathcal{z}_{3}^{2}} \\ \sigma\left( \mathcal{Z} \right)=\frac{65856000}{\left( {30.10}^{6} \right)\mathcal{z}_{4}\mathcal{z}_{3}^{3}} \\ \mathcal{P}_{c}\left( \mathcal{Z} \right)=\frac{4.013\left( {30.10}^{6} \right)\mathcal{z}_{3}\mathcal{z}_{4}^{3}}{6\times196}\left( 1-\frac{\mathcal{z}_{3}}{28}\sqrt{\frac{{30\times10}^{6}}{4\left( {12\times10}^{6} \right)}} \right) \end{aligned} \right.$

With. $0.1\leq\mathcal{z}_{1},\mathcal{z}_{4}\leq2, 0.1\leq\mathcal{z}_{2},\mathcal{z}_{3}\leq10$

**PV**

**------------------------------------------------------------------------------------------------------------------------------------------------------**Consider: $\mathcal{Z=}\left[ \mathcal{z}_{1},\mathcal{z}_{2},\mathcal{z}_{3},\mathcal{z}_{4} \right]=[\mathcal{T}_{\mathcal{s}},\mathcal{T}_{\mathcal{k}}\mathcal{,R,L]}$

Minimize: $\mathcal{F}\left( \mathcal{Z} \right)=0.6224\mathcal{z}_{1}\mathcal{z}_{3}\mathcal{z}_{4}+1.778\mathcal{z}_{2}\mathcal{z}_{3}^{2}+3.1661\mathcal{z}_{1}^{2}\mathcal{z}_{4}+19.84\mathcal{z}_{1}^{2}\mathcal{z}_{3}$

Subject to: $\left\{ \begin{aligned} \mathcal{g}_{1}\left( \mathcal{Z} \right)=-\mathcal{z}_{1}+0.0193\mathcal{z}_{3}\leq0 \\ \mathcal{g}_{2}\left( \mathcal{Z} \right)=-\mathcal{z}_{2}+0.00954\mathcal{z}_{3}\leq0 \\ \mathcal{g}_{3}\left( \mathcal{Z} \right)=-\pi\mathcal{z}_{3}^{2}\mathcal{z}_{4}-\frac{4}{3}\pi\mathcal{z}_{3}^{3}+1296000\leq0 \\ \mathcal{g}_{4}\left( \mathcal{Z} \right)=\mathcal{z}_{4}-240\leq0 \end{aligned} \right.$

With. $0\leq\mathcal{z}_{1},\mathcal{z}_{2}\leq100, 10\leq\mathcal{z}_{3},\mathcal{z}_{4}\leq200$
